# Chromosome-length genome assembly and linkage map of a critically endangered Australian bird: the helmeted honeyeater

**DOI:** 10.1093/gigascience/giac025

**Published:** 2022-03-29

**Authors:** Diana A Robledo-Ruiz, Han Ming Gan, Parwinder Kaur, Olga Dudchenko, David Weisz, Ruqayya Khan, Erez Lieberman Aiden, Ekaterina Osipova, Michael Hiller, Hernán E Morales, Michael J L Magrath, Rohan H Clarke, Paul Sunnucks, Alexandra Pavlova

**Affiliations:** School of Biological Sciences, Monash University, Clayton, VIC 3800, Australia; Deakin Genomics Centre, Deakin University, Geelong, VIC 3220, Australia; GeneSEQ Sdn Bhd, 48300 Rawang, Selangor, Malaysia; UWA School of Agriculture and Environment, The University of Western Australia, Perth WA 6009,Australia; The Center for Genome Architecture, Department of Molecular and Human Genetics, Baylor College of Medicine, Houston, TX 77030, USA; Center for Theoretical Biological Physics and Department of Computer Science, Rice University, Houston, TX 77030, USA; The Center for Genome Architecture, Department of Molecular and Human Genetics, Baylor College of Medicine, Houston, TX 77030, USA; The Center for Genome Architecture, Department of Molecular and Human Genetics, Baylor College of Medicine, Houston, TX 77030, USA; UWA School of Agriculture and Environment, The University of Western Australia, Perth WA 6009,Australia; The Center for Genome Architecture, Department of Molecular and Human Genetics, Baylor College of Medicine, Houston, TX 77030, USA; Center for Theoretical Biological Physics and Department of Computer Science, Rice University, Houston, TX 77030, USA; Broad Institute of MIT and Harvard, Cambridge, MA 02139, USA; Shanghai Institute for Advanced Immunochemical Studies, ShanghaiTech, Pudong 201210, China; Max Planck Institute of Molecular Cell Biology and Genetics, Pfotenhauerstr 108, 101307 Dresden, Germany; LOEWE Centre for Translational Biodiversity Genomics, Senckenberganlage 25, 60325 Frankfurt, Germany; Senckenberg Research Institute, Senckenberganlage 25, 60325 Frankfurt, Germany; Goethe-University, Faculty of Biosciences, Max-von-Laue-Str. 9, 60438 Frankfurt, Germany; Max Planck Institute of Molecular Cell Biology and Genetics, Pfotenhauerstr 108, 101307 Dresden, Germany; LOEWE Centre for Translational Biodiversity Genomics, Senckenberganlage 25, 60325 Frankfurt, Germany; Senckenberg Research Institute, Senckenberganlage 25, 60325 Frankfurt, Germany; Goethe-University, Faculty of Biosciences, Max-von-Laue-Str. 9, 60438 Frankfurt, Germany; Section for Evolutionary Genomics, GLOBE Institute, University of Copenhagen, Denmark; Department of Wildlife Conservation and Science, Zoos Victoria, Parkville, VIC 3052, Australia; School of Biological Sciences, Monash University, Clayton, VIC 3800, Australia; School of Biological Sciences, Monash University, Clayton, VIC 3800, Australia; School of Biological Sciences, Monash University, Clayton, VIC 3800, Australia

## Abstract

**Background:**

The helmeted honeyeater (*Lichenostomus melanops cassidix*) is a Critically Endangered bird endemic to Victoria, Australia. To aid its conservation, the population is the subject of genetic rescue. To understand, monitor, and modulate the effects of genetic rescue on the helmeted honeyeater genome, a chromosome-length genome and a high-density linkage map are required.

**Results:**

We used a combination of Illumina, Oxford Nanopore, and Hi-C sequencing technologies to assemble a chromosome-length genome of the helmeted honeyeater, comprising 906 scaffolds, with length of 1.1 Gb and scaffold N50 of 63.8 Mb. Annotation comprised 57,181 gene models. Using a pedigree of 257 birds and 53,111 single-nucleotide polymorphisms, we obtained high-density linkage and recombination maps for 25 autosomes and Z chromosome. The total sex-averaged linkage map was 1,347 cM long, with the male map being 6.7% longer than the female map. Recombination maps revealed sexually dimorphic recombination rates (overall higher in males), with average recombination rate of 1.8 cM/Mb. Comparative analyses revealed high synteny of the helmeted honeyeater genome with that of 3 passerine species (e.g., 32 Hi-C scaffolds mapped to 30 zebra finch autosomes and Z chromosome). The genome assembly and linkage map suggest that the helmeted honeyeater exhibits a fission of chromosome 1A into 2 chromosomes relative to zebra finch. PSMC analysis showed a ∼15-fold decline in effective population size to ∼60,000 from mid- to late Pleistocene.

**Conclusions:**

The annotated chromosome-length genome and high-density linkage map provide rich resources for evolutionary studies and will be fundamental in guiding conservation efforts for the helmeted honeyeater.

## Background

Despite advances in sequencing technologies in recent years, high-quality genomes at the chromosome scale for non-model species remain rare. For example, as of 12 July 2021, for the Class Aves, there are only 83 genome assemblies classified as chromosome-length available in the NCBI GenBank. Chromosome-length assemblies have several advantages over scaffold-level assemblies. They facilitate identification of large-scale rearrangements and syntenic relationships among related organisms. Once annotated, they provide a platform that informs of the position of genes relative to each other and with respect to chromosomal structures (e.g., centromeres, telomeres, repeat elements, and regulatory regions) and enable more complete gene models, which contributes to understanding the organization and function of the genome [1]. Chromosome-length assemblies also provide a template for estimating linkage disequilibrium over long genomic regions, enabling reconstruction of very recent demographic history, precise quantification of relatedness and inbreeding (e.g., identity-by-descent and runs of homozygosity, respectively), and detection of genomic regions under natural selection [2–5].

Some genomic methods require pairing a chromosome-length genome assembly with its high-density linkage map. For example, linkage maps allow the study of variation in recombination rates along the genome, between sexes, individuals, populations, and species [6–8]. Incorporating recombination rates into genomic analyses facilitates the identification of evolutionary processes, such as genetic drift, natural selection, and gene flow [5, 9]. It also contributes to our understanding of the influence of structural variants and chromosomal rearrangements on these processes [9]. Thus, in combination with chromosomal-length assembly, linkage and recombination maps provide a powerful resource for answering important questions in ecology, and evolutionary and conservation biology. However, obtaining linkage maps requires genotypic data from multiple known families, which represents an important limiting factor for many species. Currently, few bird species have both a chromosome-length genome assembly and an associated high-density linkage map (e.g., domestic chicken *Gallus gallus*; great tit *Parus major*; zebra finch *Taeniopygia guttata*; collared flycatcher *Ficedula albicolis*; house sparrow *Passer domesticus*; rock pigeon *Columba livia*; superb fairy-wren *Malurus cyaneus*) [1,10–15].

**Figure 1:**
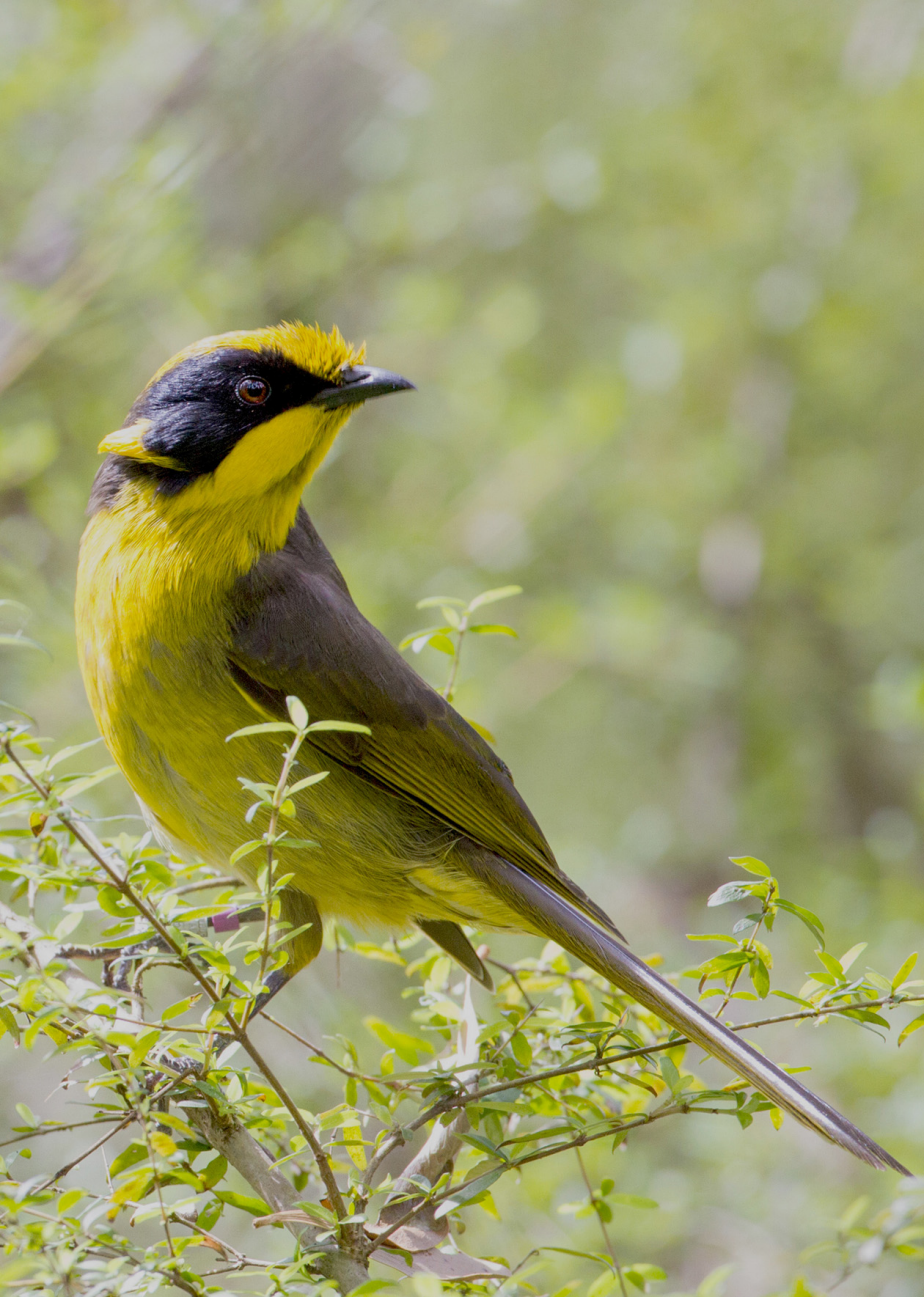
Helena, the helmeted honeyeater (*Lichenostomus melanops cassidix*) whose genome was sequenced, at Yellingbo Nature Conservation Reserve (Victoria, Australia). Photo by Nick Bradsworth.

The helmeted honeyeater, *Lichenostomus melanops cassidix* (NCBI:txid1497555), is a member of the superfamily Meliphagoidea. Distinguished by its characteristic “helmet” of crown feathers, it is 1 of 4 subspecies of yellow-tufted honeyeater (*L. melanops*) (Fig. 1). Endemic to the state of Victoria, Australia, it was declared Victoria's bird emblem in 1971. It has been classified as Critically Endangered, and its sole population consists of just ∼250 individuals inhabiting the Yellingbo Nature Conservation Reserve (YNCR) [16]. The helmeted honeyeater has been subject to intensive conservation management, including captive breeding [17]. Ecological data and genetic samples have been collected for >3 decades, which enabled the recent construction of a multigenerational pedigree spanning 257 helmeted honeyeaters [18].

Despite intensive and comprehensive conservation efforts, the population of the helmeted honeyeater exhibits a small effective population size, low genetic variation, and strong inbreeding depression [19–20]. After projections showed that without intervention the population's genetic health would continue to decline, a genetic rescue trial commenced in 2018 to facilitate gene flow from its closest relative and neighbour subspecies *L. melanops gippslandicus* [19, 21]. Genetic rescue aims to reduce inbreeding levels and increase the genetic diversity of a population to avoid extinction and restore evolutionary potential [22–23]. However, limited understanding of the genome-wide consequences of genetic rescue hinders efficient genetic monitoring [24]. Here, we present an annotated chromosome-length assembly of the ∼1.1 Gb genome of the helmeted honeyeater, and a high-density linkage map for 25 autosomes and Z chromosome. These resources will provide the basis for studies that seek to understand, monitor, and modulate the effects of genetic rescue on the genome of the helmeted honeyeater and will contribute to developing management approaches for other threatened species.

## Data Description

We sequenced and assembled the nuclear and mitochondrial genomes of a wild-born adult (>10 year old) female helmeted honeyeater, banded on 26 October 2010 (ABBBS metal band 043-00510, colour bands pm:uk, Healesville accession B80296; nicknamed “Helena” by the Helmeted Honeyeater Recovery Team). This female successfully bred for ≥7 breeding seasons at YNCR and was included in 3 genetic studies [20, 21, 18], which revealed that it was genetically diverse and had longer than average life span and higher than average number of fledglings. After presenting symptoms of periarticular gout and nephropathy, this bird was humanely euthanized for animal welfare reasons at Healesville Sanctuary's Australian Wildlife Health Centre on 27 February 2018 under the authority of Zoos Victoria Research and Animal Ethics Committee (approval ZV16010). A combined sequencing strategy was applied to obtain the helmeted honeyeater genome and linkage map. A summary of all genomic resources, sample IDs, and accession numbers can be found in Table 1.

**Table 1:** Summary of the genomic resources produced in this study

Genomic Resource	Value
**Draft genome sequencing**	
NCBI BioProject	PRJNA554936
Sample ID genome	B80296
BioSample DNAseq	SAMN12287370
Short-read Illumina NovaSeq data (Gb)	220
Short-read NCBI-SRA accession Illumina NovaSeq	SRX6469119
Long-read Oxford Nanopore MinION data (Gb)	19.9
Long-read NCBI-SRA accessions Nanopore	SRX6458354, SRX6458355, SRX6458356
**Hi-C sequencing**	
NCBI BioProject	PRJNA512907
Sample ID genome	Sample2749A
BioSample DNAseq	SAMN16895762
Hi-C Illumina NovaSeq data (Gb)	41.6
Hi-C NCBI-SRA accession HiC	SRX9606522
**Draft genome assembly (HeHo_1.0)**	
Assembled genome size (Gb)	1.1
Scaffold N50 (bp)	7,973,128
No. of scaffolds	1,912
Contig N50 (bp)	7,673,876
No. of contigs	1,929
NCBI GenBank assembly accession	GCA_008360975.1
Whole-genome shotgun accession	VLJF00000000.1
BUSCO completeness	97.1% Complete, 0.7% fragmented, 2.2% missing
**Chromosome-length assembly (HeHo_2.0)**	
Assembled genome size (Gb)	1.103
Scaffold N50 (bp)	63,800,663
No. of scaffolds	906
Contig N50 (bp)	6,736,108
No. of contigs	2,239
NCBI GenBank assembly accession	GCA_008360975.2
Whole-genome shotgun accession	VLJF00000000.2
BUSCO completeness	97.1% Complete, 0.7% fragmented, 2.2% missing
**Mitochondrial genome assembly**	
NCBI BioProject	PRJNA554936
Sample ID genome	B80296
Short-read NCBI-SRA accession Illumina NovaSeq	SRX6469119
NCBI GenBank assembly accession	OK189508
Assembled genome size (bp)	16,851
**Genome annotation**	
No. of predicted protein-coding genes	29,454
No. of functionally annotated protein-coding genes	18,058
No. of genes with GO annotations	12,710
BUSCO completeness	99.4% Complete, 0.2% fragmented, 0.4% missing
DOI for annotations	doi.org/10.26180/16695607
**DArT sequencing**	
DArT sequencing NCBI-SRA accessions	SAMN25688276-SAMN25688532
**Linkage and recombination map**	
DOI for linkage and recombination maps	doi.org/10.26180/16695607

### Draft genome assembly

**Short-read sequencing**. For short-read DNA libraries, DNA was extracted from muscle tissue preserved in ethanol, using Qiagen DNeasy Blood & Tissue kits. A total of 100 ng genomic DNA was fragmented to 350 bp using QSonica and processed with a New England Biolabs (NEB) Next Ultra DNA Library Prep Kit for Illumina®. The library was pooled with libraries for other projects and sequenced on all 4 lanes of S4 flowcell of a NovaSeq 6000 Sequencing System (Illumina) at the Deakin Genomics Centre using 2 × 151 bp run configuration. In total, we obtained 220 Gb of raw sequence data (GenBank accession SRX6469119).

**Long-read sequencing**. A total of 1 Nanopore LSK108 and 2 Nanopore LSK109 libraries were run on 3 individual MinION revD flowcells, generating a total of 19.9 Gb data. The first LSK108 library was constructed using the same DNA source as the Illumina run and generated only 2.9 Gb data (GenBank accession SRX6458354). Higher run output was obtained after switching to the LSK109 library preparation kit. For the second run, aiming for more output (9.9 Gb) but associated with shorter reads, DNA was extracted from frozen liver tissue using Zymo Quick DNA miniprep kit (GenBank accession SRX6458355). For the third run, aiming for longer reads but less output (7.1 Gb), DNA was extracted from muscle tissue frozen without Zymo RNA/DNA Shield buffer using conventional salting out/ethanol precipitation approach [25] (GenBank accession SRX6458356). Base-calling used Guppy 3.1.5+781ed57 high-accuracy model (dna_r9.4.1_450bps_hac.cfg).

**De novo assembly**. To generate a draft genome (GenBank accession GCA_008360975.1) we assembled Illumina reads, adapter-trimmed using fastp v0.19.5 (fastp, RRID:SCR_016962) [26], and Nanopore long reads*de novo* using MaSuRCA v3.3.3 (MaSuRCA, RRID:SCR_010691) [27]. The MaSuRCA pipeline error-corrected the short Illumina reads and used them to construct contigs by the de Bruijn graph approach. These contigs were used to error-correct the Nanopore long reads, generating “mega read” contigs for Overlap-Layout-Consensus assembly. This draft genome of the helmeted honeyeater contained 1,929 contigs with a contig N50 length of 7,673,876 and a total length of 1,102,302,466 bp (Table 1). Genome completeness was assessed using BUSCO v5.2.1 (BUSCO, RRID:SCR_015008) [28] with the aves_odb10 lineage and default settings, which revealed a complete recall of 97.1% of genes, 0.7% fragmented, and 2.2% missing.

### Chromosome-length genome assembly

**Hi-C sequencing**. To produce a chromosome-length genome assembly, a frozen liver sample was used to construct *in situ* a Hi-C library as described in [29]. A total of 138,592,561 paired-end (150 bp) Hi-C reads were generated using NovaSeq 6000 (Illumina). The Hi-C library and reads were generated by the DNA Zoo Consortium [30].

**Chromosome-length assembly**. The draft genome was scaffolded to chromosome-length by the DNA Zoo Consortium following the methods described in [30]. The Hi-C data were processed using Juicer (Juicer, RRID:SCR_017226) [31] and used as input into the 3D-DNA pipeline (3D DNA pipeline, RRID:SCR_017227) [32] to produce a candidate chromosome-length genome assembly. We performed additional finishing on the scaffolds using Juicebox Assembly Tools (Juicebox, RRID:SCR_021172) [33, 34]. The percent of unmapped sequenced Hi-C read pairs was very low (0.81%), with 75.50% of the library representing unique Hi-C contacts. The contact matrices generated by aligning the Hi-C data to the genome assembly before and after the Hi-C scaffolding are available for browsing interactively at multiple resolutions at [35] visualized using Juicebox.js, a cloud-based visualization system for Hi-C data [36].

A total of 1,102,960,466 bp were assembled into the chromosome-length genome (GenBank accession GCA_008360975.2) with scaffold N50 of 63.8 Mb and longest scaffold of 152.7 Mb (Table 1). BUSCO assessment of the chromosome-length assembly (conducted as explained above) revealed a level of genome completeness similar to that of the draft genome (complete recall of 97.1% of genes, 0.7% fragmented, and 2.2% missing).

### Mitochondrial genome assembly

MITObim v1.6 (MITObim, RRID:SCR_015056) [37] was used to assemble the whole mitogenome from Illumina short-read sequencing data, using the *ND2* gene sequence of another helmeted honeyeater (GenBank accession KJ586920) [19] as the bait for iterative mapping assembly. The assembled genome was circularized, re-oriented, and annotated using MITOS [38, 39]. The homology of the helmeted honeyeater mitogenome to mitogenomes of other members of family Meliphagidae available in the NCBI nucleotide database was validated by BLASTn analysis (BLASTN, RRID:SCR_001598) (best match to noisy miner *Manorina melanocephala*, GenBank accession KY994587; 90.25% identity; Supplementary Material S1). Geneious v6.1 (Geneious, RRID:SCR_010519) [40] was used to manually check mitogenome annotations for absence of premature stop codons and consistency of coding gene annotations with those of noisy miner (KY994587); a start codon was added to *ND6* gene to rectify a single discrepancy.

The helmeted honeyeater mitogenome is 16,849 bp long, encoding 13 protein-coding genes, 2 ribosomal RNA genes (12S rRNA and 16S rRNA), and 22 transfer RNA genes (Supplementary Material S2; GenBank accession OK189508). LASTZ v1.04.03 (LASTZ, RRID:SCR_018556) [41] was used to align the mitogenome to the chromosome-length genome using default parameters except for disabled seed transitions (--notransition), K = 4500, L = 300, and enabled chaining (--chain). In total, 22 Hi-C scaffolds mapped to the mitochondrial sequence. These included 12 short Hi-C scaffolds comprising 16,090 bp of the mitogenome, fragments of 9 long Hi-C scaffolds corresponding to nuclear chromosomes 1, 2, 3, 5, 8, 11, 24, Z, and W (which indicated the presence of nuclear copies of mitochondrial DNA on these chromosomes), and a short Hi-C scaffold that was not assembled to other chromosomes (Supplementary Material S3). Alignment of the mitogenome to the draft genome did not reveal additional findings.

### Annotations

We identified repeat families in the helmeted honeyeater Hi-C genome using RepeatModeler v1.0.9 (RepeatModeler, RRID:SCR_015027) [42] with “-engine ncbi” option, and soft-masked repeats using RepeatMasker v4.1.2 (RepeatMasker, RRID:SCR_012954) [43]. We then combined orthology predictions, protein data from birds, and *ab initio* gene predictions to produce a high-quality protein-coding gene annotation for the helmeted honeyeater chromosome-length assembly.

First, we generated pairwise alignment chains between the helmeted honeyeater and the reference genomes of chicken, zebra finch, and great tit (GeneBank accessions GCA_000002315.5, GCA_003957565.2, and GCA_001522545.3, respectively) using LASTZ v1.04.03 with parameters K = 2,400, L = 3,000, Y = 9,400, H = 2,000 and the default scoring matrix, axtChain [44], chainCleaner [45], and RepeatFiller (RepeatFiller, RRID:SCR_017414) [46]. Potential orthologous genes were inferred by projecting transcripts annotated for the 3 reference species to the helmeted honeyeater genome using the generated alignment chains and TOGA [47]. NCBI annotations of zebra finch and great tit (46,022 and 41,530 gene models, respectively) and combined chicken NCBI annotation with APPRIS principal isoforms (total of 64,081 gene models) were used as reference annotations.

We prepared the protein library combining proteomes of 23 avian species and 7 species outside of the avian clade available on NCBI (Supplementary Material S4) and aligned the library to the helmeted honeyeater genome using GenomeThreader v1.7.1 [48], applying the Bayesian Splice Site Model (BSSM) trained for chicken. For protein GenomeThreader alignments, a seed and minimum match length of 20 amino acids (preseedlength 20, prminmatchlen 20) and a Hamming distance of 2 (prhdist 2) were used. For the transcript alignments, a seed length and minimum match length of 32 nucleotides (seedlength 32, minmatchlen 32) were used. At least 70% of the protein or mRNA sequence was required to be covered by the alignment (-gcmincoverage 70), and potential paralogous genes were also computed (-paralogs).

Next, we used Augustus v3.3.3 (Augustus, RRID:SCR_008417) [49] to obtain *de novo* gene predictions, providing TOGA projections and mapped protein data as hints. Prediction of additional splice sites was enabled (--allow_hinted_splicesites = gcag,atac), and prediction of untranslated regions was disabled (--UTR = off). The resulting set of gene models was filtered to exclude models with >10% overlap with a repeat region using bedtools intersect. The remaining gene models were converted to protein and queried against the Swissprot database using blastp (BLASTP, RRID:SCR_001010) with an E-value cut-off 1e−10. Only hits matching a sequence in the vertebrate database or hits >200 amino acids long were retained. *De novo* gene prediction resulted in 33,844 gene models.

Finally, we used EVidenceModeler v1.1.1 (EVidenceModeler, RRID:SCR_014659) [50] to combine TOGA projections, aligned protein data, and *de novo* gene models (with respective weights of 8, 2, and 1) into a consensus set of 18,280 gene models, each represented by a single transcript. This set of transcripts was extended by adding TOGA transcript projections that were identical for ≥2 of the 3 reference species. The final annotation comprised 57,181 gene models.

**Figure 2:**
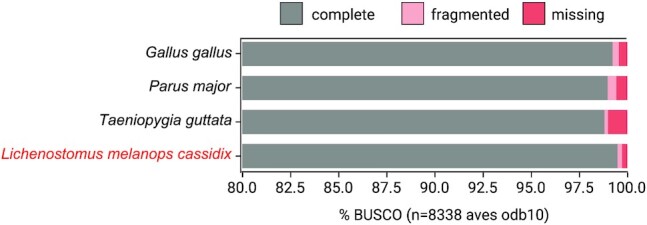
Comparison of the completeness of gene annotations of reference NCBI annotations and the newly produced helmeted honeyeater annotation, as a percentage of 8,338 avian genes from BUSCO (odb10).

To assess annotation completeness, we used BUSCO v5.2.1 and the set of 8,338 conserved single-copy avian genes (aves_odb10). Our final annotation showed a high level of completeness, with 99.4% of the BUSCO genes being complete, 0.2% fragmented, and 0.4% missing. This level of completeness is higher than for the genomes of chicken, zebra finch, and great tit used for alignment (Fig. 2).

### Synteny analysis

**Figure 3:**
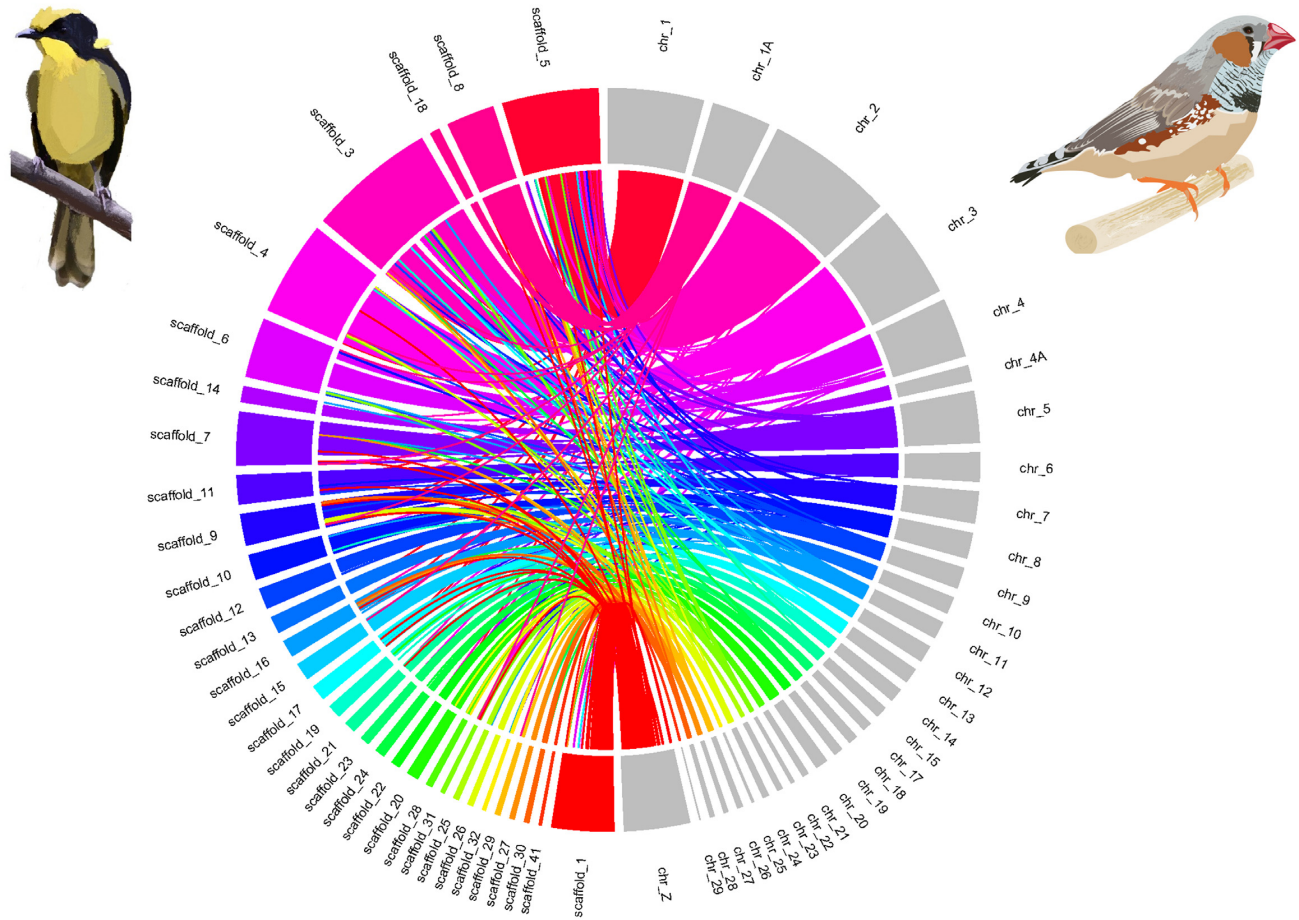
Synteny between the helmeted honeyeater Hi-C scaffolds (left) and the chromosomes of the zebra finch assembly (right).

To validate the genome assembly and do a preliminary assignment of each Hi-C scaffold to a putative chromosome, we aligned it to the most recent female zebra finch genome assembly available in March 2021 (bTaeGut2.pat.W.v2, GenBank accession GCA_008822105.2). The zebra finch was the second avian genome to ever be sequenced [51] and is therefore a model genome from a passerine species in NCBI. Owing to the lack of chromosome 16 in this assembly, we used chromosome 16 of the most recent male assembly (bTaeGut1.pri.v2, GenBank accession CM012098.1). Using LASTZ v1.04.03 we aligned all Hi-C scaffolds to the 29 autosomes and both sex chromosomes of the zebra finch genome with the same parameters as for the alignment of the mitogenome (see section “Mitochondrial genome assembly”). Initial inspection of the alignment for each chromosome revealed good alignments of 32 of the largest Hi-C scaffolds to all zebra finch chromosomes except for 16 and W (Supplementary Material S5). Chromosome 16 aligned to 49 short fragments (length 5–20 kb) of Hi-C scaffold 36 but also to up to 6 smaller fragments (5–10 kb) of 21 additional Hi-C scaffolds (Supplementary Material S6). Owing to the lack of unambiguous alignment to a single Hi-C scaffold, chromosome 16 was excluded from downstream linkage map analyses. Hi-C scaffold 2 did not align to the zebra finch genome but was inferred to represent the helmeted honeyeater W chromosome, based on (i) haploid read depth coverage, (ii) the presence of the chromo-helicase DNA-binding protein gene (*CHD1-W*, zebra finch NCBI Gene ID 778443) that is used for avian molecular sexing [52] (Supplementary Material S7), and (iii) the lack of heterozygous markers called by Lep-MAP3 module ParentCall2, consistent with these markers being hemizygous (see section “Construction of linkage map with Lep-MAP3”). Hi-C scaffold 1 was inferred to be the Z chromosome, based on (i) haploid read depth coverage, (ii) alignment to zebra finch Z chromosome, and (iii) presence of the *CHD1-Z*gene (zebra finch NCBI Gene ID 778444) (Supplementary Material S7). Large-scale synteny was represented with a CIRCOS plot to qualitatively show the assignment of putative chromosomes and assess chromosomal rearrangements (Fig. 3). The CIRCOS plot was built with the R package circlize v0.4.12 (circlize, RRID:SCR_002141) [53] using aligned sequences of length ≥5,000 bp. We also analysed the synteny between the helmeted honeyeater assembly and 2 other passerine genomes: the collared flycatcher and superb fairy-wren (FicAlb1.5, GenBank accession GCA_000247815.2; mCya_1.0, GenBank accession GCA_009741485.1; respectively).

We observed largely conserved synteny between the helmeted honeyeater scaffolds and the zebra finch genome (Fig. 3). The synteny was mostly captured by 32 of the largest Hi-C scaffolds that mapped to 30 zebra finch autosomes, plus its Z chromosome. A probable fission of chromosome 1A into 2 putative chromosomes in helmeted honeyeater relative to zebra finch was apparent because Hi-C scaffolds 8 and 18 both mapped to zebra finch 1A (Fig. 3): the larger Hi-C scaffold 8 mainly mapped to the first ∼50 Mb of zebra finch 1A chromosome, and the smaller Hi-C scaffold 18 to the last ∼20 Mb. Consistent with the observed trend in the class Aves [54], the same pattern of overall highly conserved synteny was found with the collared flycatcher and superb fairy-wren genomes (Supplementary Material S8), with Hi-C scaffolds 8 and 18 mapping to chromosome 1A in both.

### Linkage and recombination maps

**Preparing input for Lep-MAP3**. The helmeted honeyeater genetic map was constructed using a pedigree and the genotype posterior probabilities (obtained from DArT sequencing) of all the individuals in the pedigree with the software Lep-MAP3 v0.2 [55].

*Pedigree*. Using the results of a previous parentage analysis [18], we selected the 36 full-sibling families (father-mother-offspring) that had ≥3 full-siblings (range = 3–14, mean = 5.69 [SD 3.22]; 206 offspring in total). When possible, grandparents and half-siblings of these families were included. Some birds were present more than once in the pedigree (e.g., either as offspring, parent, or grandparent), yielding 257 unique individuals in total (Supplementary Material S9).

*Genotype posterior probabilities*. We used raw sequencing data obtained using DArTseq [56] from a previous study [18] for the selected 257 related individuals. Briefly, DArTseq is a reduced-representation sequencing method that uses a combination of PstI and SphI restriction enzymes for DNA digestion, sequencing fragments with both PstI- and SphI-compatible adapters on Illumina HiSeq2500 using single-read configuration (for details see [20]). We trimmed Illumina adapters from the raw DArTseq reads with fastp v0.20.0 [26], demultiplexed them, and removed barcodes with process_radtags v2.41 (Stacks, RRID:SCR_003184) [57]. Trimmed reads were mapped to the Hi-C genome using BWA v0.7.17 (BWA, RRID:SCR_010910) [58]. Individual sam files were converted to bam files and sorted with SAMtools v1.11 (SAMtools/BCFtools, RRID:SCR_005227) [59] excluding reads with MAPQ < 20 (option -q 20). Genotype posterior probabilities (likelihoods) were obtained using the pipeline based on SAMtools mpileup [60] provided by Lep-MAP3. This pipeline used as input a list of the 257 individuals and their respective bam files, yielding a file of the genotype likelihoods for each individual and marker.

**Construction of linkage map with Lep-MAP3**. The following Lep-MAP3 modules were used to construct the helmeted honeyeater linkage map for 28 autosomes and the Z chromosome:

ParentCall2 module was used to call individual genotypes from genotype posterior probabilities taking into account the genotypic information of the pedigree. Monomorphic loci were filtered out (removeNonInformative = 1). Information from half-siblings was used (halfSibs = 1) to call single-nucleotide polymorphisms (SNPs) on autosomes (default parameters) and the Z chromosome (ZLimit = 2). Genotype calling identified 83,628 informative markers (including 2,988 Z markers).

Filtering2 module was used to remove SNPs with high distortion from Mendelian segregation by setting the dataTolerance parameter to 0.001. No SNPs were removed.

SeparateChromosomes2 module was used to calculate pairwise logarithm of odds (LOD) scores for each pair of SNPs (i.e., statistical estimate of whether 2 genes are likely to be located near each other [61]) and split them into linkage groups (LGs, likely chromosomes) according to the user-specified LOD score limit. Following [1], we did independent runs of this module with different LOD score limits (lodLimit = 11–23) to find the LOD that grouped SNPs in LGs that better recovered the putative chromosomes found from the synteny analysis with the zebra finch genome (Fig. 3). We selected a LOD score limit of 21 as the most conservative score where few SNPs from different putative chromosomes were assigned to the same LG, but SNPs from the same putative chromosome were not split into different LGs (Supplementary Material S10, black arrows). We also specified a minimum LG size of 100 markers (sizeLimit = 100). SeparateChromosomes2 was used to assign 41,542 markers to 29 LGs. Putative chromosomes 22, 25, and 29 were not recovered as LGs owing to the limited number of markers available for them (111, 68, and 68 SNPs were present in scaffolds 31, 32, and 41, respectively; but <100 SNPs from scaffold 31 were grouped in the corresponding LG for chromosome 22).

JoinSingles2All module was used to iteratively assign “singles” (i.e., SNPs not assigned to any LG by SeparateChromosomes2) to the existing LGs using a lower LOD limit. Similar to the previous step, we did multiple iterations with different LOD score limits (lodLimit = 10–19) to select the score that retrieved as many singles as possible without misassigning them to a different putative chromosome based on synteny as above (Supplementary Material S11). We chose LOD score 13, which assigned 16,845 singles, giving a total of 58,387 SNPs.

OrderMarkers2 module was then used to find the most likely order of SNPs in each LG and calculate sex-specific genetic distances in centimorgans (cM). This module was run with default parameters 10 independent times for each LG and the map with the highest likelihood was selected. For the LG that corresponded to the putative Z chromosome, we set female recombination to zero (recombination2 = 0).

**Linkage map curation and recombination map with MareyMap**.The post-processing of the genetic map for each LG was done with the online software MareyMap (MAREYMAP, RRID:SCR_009066) [62].

*Manual curation*. We built Marey maps [63] by plotting SNP genetic distance against SNP physical distance for each LG and sex. The 4,766 aberrant SNPs that disrupted the monotonically increasing trends of the Marey maps (i.e., their genetic position disagreed with their physical position) were manually removed (Supplementary Material S12). These aberrant SNPs could have resulted from the limited size of the mapping population (257 individuals), low allelic frequency, or polymorphic structural variation within the mapping population [1,64]. Marey maps for putative chromosomes Z, 13, and 26 contained large regions that were not consistent with the pattern of monotonic increase, which suggested possible misassemblies during Hi-C scaffolding (Supplementary Material S12). To further examine this, we mapped adapter-trimmed Oxford Nanopore reads with minimum base quality score of 7 and longer than 10 kb to these chromosomes to visually inspect the read coverage on the predicted breakpoints (GenBank accession SRX6458354, SRX6458355, SRX6458356) (Porechop, RRID:SCR_016967; NanoFilt, RRID:SCR_016966; Minimap2, RRID:SCR_018550; Integrative Genomics Viewer, RRID:SCR_011793) [65–67]. For all 3 chromosomes, we found no mapped reads on a 500-bp stretch at the possible breakpoint, which gave support to the possibility of a misassembly (Supplementary Material S13). Thus, SNPs located within these regions were excluded from downstream analyses (502, 858, and 143 SNPs, respectively). We advise consideration of the aforementioned information when using chromosomes Z, 13, and 26 of this assembly.

**Figure 4:**
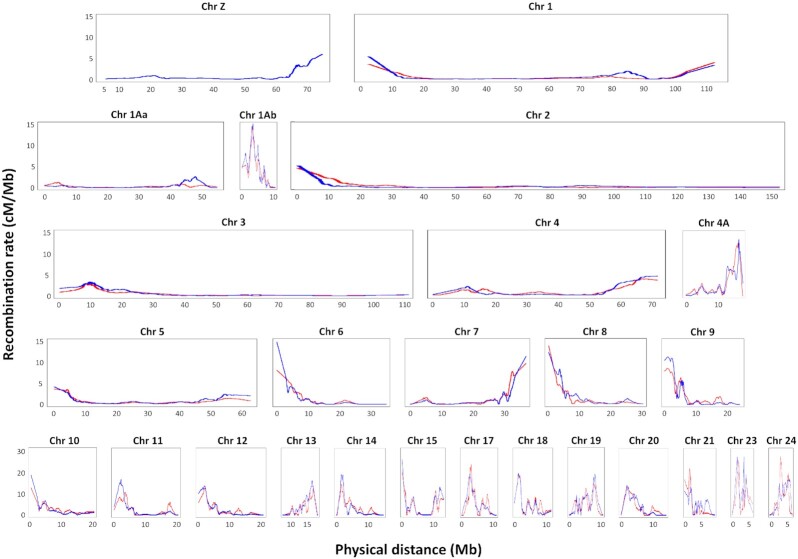
Comparison of sex-specific recombination maps. The recombination rates for all chromosomes are compared between female (red) and male (blue) maps. Note the change in scale of the y-axis in the bottom row.

*Recombination map*. A final set of 53,225 curated informative SNPs was used to calculate sex-specific local recombination rates using a locally weighted regression model (LOESS) with span parameter of 0.2 in MareyMap online. This method estimates the local recombination rates (cM/Mb) as the slope of the curve describing the relationship between the physical (Mb) and genetic (cM) positions. Probably owing to very low SNP density in some regions of their genetic maps, we obtained large negative local recombination rates (range: −0.57 to −8.74) in some regions of the LGs corresponding to putative chromosomes 26, 27, and 28 (Supplementary Material S14). We considered these linkage and recombination maps unreliable and discarded them. For the remaining 25 putative chromosomes, there were some regions with small negative local recombination rate values (range: −0.01 to −0.56) that coincided with flat regions in their Marey maps and are likely mathematical artefacts of the smoothing method with no biological meaning (L. Guéguen, personal communication). Given that the slope of those flat regions in the Marey maps is zero, we converted the small negative recombination values to zero. We plotted sex-specific recombination rates against physical position (Mb) (Fig. 4; see Supplementary Material S15 for individual plots). We made available the maps with the original values and with the zero-converted values.

In total, we obtained linkage and recombination maps for 25 of the 31 putative autosomes for which we found a syntenic relationship to the zebra finch genome. The complete linkage map was obtained from a total of 53,111 curated SNPs, from which 2,070 were used for the linkage map of the Z chromosome (Table 2).

**Table 2:** Summary of nuclear chromosome metrics for helmeted honeyeater assembly and linkage map

Chromosome*	Hi-C scaffold	Chromosome physical size (Mb)	No. of markers	Genetic distance (cM)		
				Female	Male	Average
Z	1	74.88	2,070		60.21	
W	2	24.15				
1	5	115.34	6,686	87.963	100.914	94.44
1Aa	8	57.70	2,415	21.60	26.56	24.32
1Ab	18	11.65	1,118	47.18	54.10	49.892
2	3	152.68	5,937	68.91	64.24	65.52
3	4	113.35	6,048	53.49	65.78	59.63
4	6	71.40	4,242	67.88	68.67	68.53
4A	14	19.12	1,618	55.17	47.14	52.21
5	7	63.80	3,823	42.70	52.04	47.36
6	11	35.01	2,122	58.45	49.91	53.16
7	9	37.86	2,250	60.62	54.80	60.13
8	10	30.39	1,755	47.59	63.53	56.54
9	12	24.91	1,684	47.73	50.72	51.84
10	13	20.33	1,518	56.82	57.95	57.02
11	16	20.49	943	48.26	59.30	53.78
12	15	20.79	1,503	48.10	52.27	50.24
13	17	18.71	862	39.23	45.27	42.25
14	19	16.12	1,165	49.68	43.88	46.53
15	21	13.55	678	53.32	69.36	61.34
17	23	11.06	829	54.52	55.78	55.15
18	24	11.99	849	59.35	51.89	55.62
19	22	10.88	785	52.09	58.42	54.71
20	20	14.36	1,149	52.46	52.15	51.42
21	28	7.78	320	26.90	30.17	27.96
22	31	5.22	111			
23	25	6.87	286	55.62	65.399	59.95
24	26	6.87	456	46.31	48.55	47.43
25	32	4.45	68			
26	29	6.47	231			
27	27	6.16	126			
28	30	6.31	144			
29	41	3.58	68			
**Total**		1,022.15		1,680.84	1,924.17	1,738.28

*Chromosomes are assigned on the basis of synteny with zebra finch.

Confirming the findings of the synteny analysis between helmeted honeyeater and zebra finch, we found that markers that mapped to zebra finch chromosome 1A are split into 2 different helmeted honeyeater LGs. This phenomenon was not unique to the selected LOD = 21, as it occurred during the process of LG discovery (Lep-MAP3 module SeparateChromosomes2) as early as with LOD = 13 (see Supplementary Material S10, asterisks). For this reason, we infer a fission of chromosome 1A into 2 chromosomes in helmeted honeyeater relative to zebra finch, and refer to them as putative chromosomes 1Aa and 1Ab hereafter. This fission was not observed in the superb fairy-wren, the sole other species from the Meliphagoidea superfamily with a chromosome-length genome that allows large-scale synteny analyses, and the high-density linkage map necessary to confirm within-chromosome linkage [1]. Neither has it been reported for other passerine birds with both resources available (i.e., great tit, collared flycatcher, and house sparrow) [11, 13, 14].

**Figure 5:**
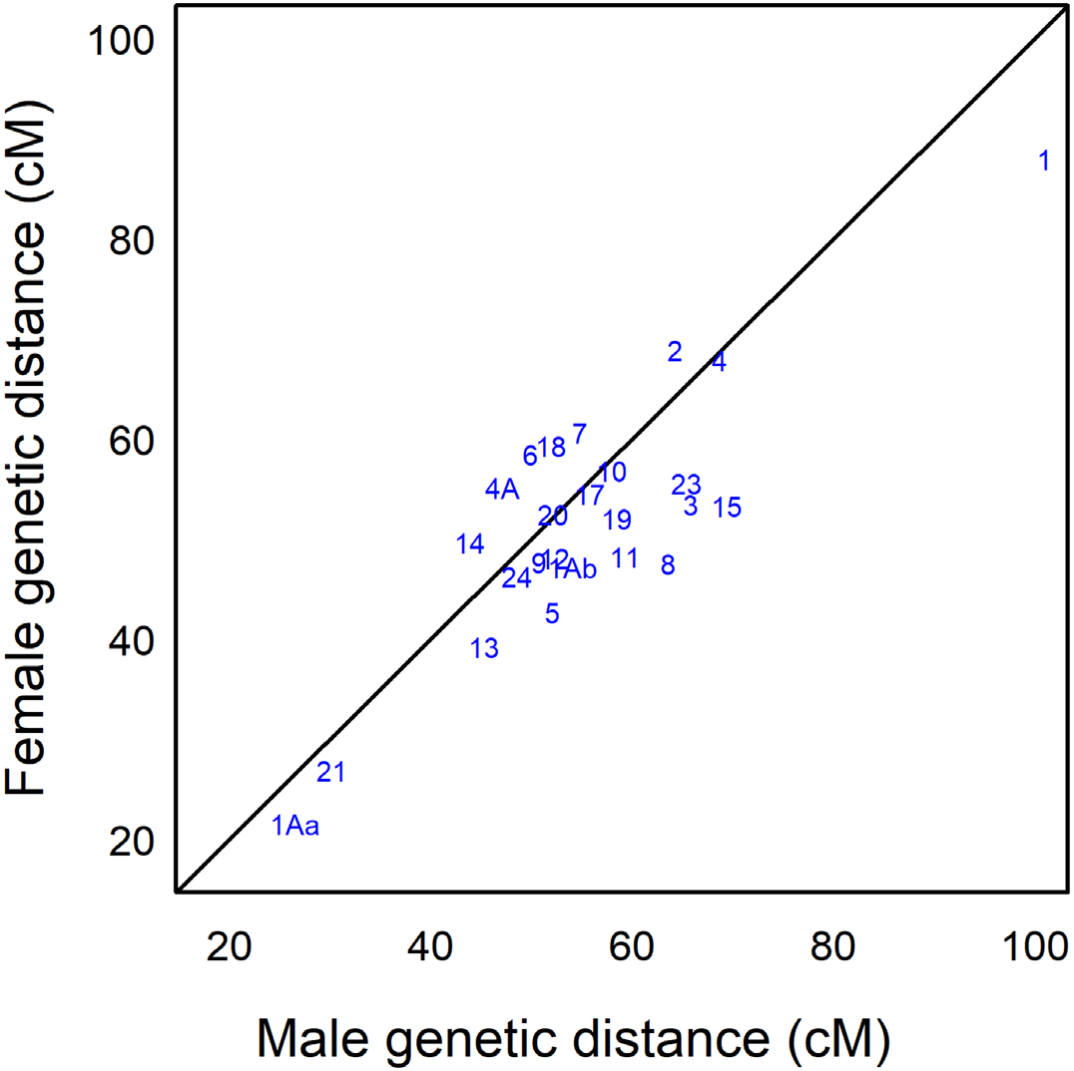
Comparison of genetic map length (measured in centimorgans) between male and female helmeted honeyeater for each chromosome. Chromosomes on the black diagonal line have approximately the same genetic distance in both sexes, below the line are longer in male, and above the line are longer in female.

The total sex-averaged linkage map length was 1,347 cM (Table 2). The male genetic map was 6.7% longer than that of females (1,389 cM vs 1,302 cM, excluding Z chromosome). This is consistent with results for superb fairy-wren, where the male-specific map was 8% longer than the female-specific one [1]. A larger difference in genetic map length between sexes has been found in collared flycatcher (10% longer in males), and a small difference in the opposite direction in zebra finch (2% longer in females) [12, 13]. The difference in helmeted honeyeater genetic map length between males and females varied across chromosomes: 15 chromosome maps were longer in males, 6 in females, and 4 were similar (Table 2, Fig. 5).

As found in other passerine genomes [1, 12, 13], a large proportion of the helmeted honeyeater genome showed sexually dimorphic recombination rates. The helmeted honeyeater map presents overall higher mean recombination rates in males (male: 1.86 cM/Mb [SD 3.08], female: 1.71 cM/Mb [SD 2.78]; Fig. 4). The overall mean difference in male to female recombination rate was 0.19 cM/Mb (SD 1.58), with the largest mean difference found on putative chromosome 23 (3.81 cM/Mb [SD 6.31]), and the smallest on putative chromosome 18 (−0.94 cM/Mb [SD 1.26]). Consistent with genomic observations in birds [54], the highest recombination rates were found in the shortest chromosomes (because ≥1 recombination event per chromosome is necessary for adequate chromosomal segregation during meiosis) and around the chromosome ends, except for the smallest chromosomes 1Ab, 23, and 24. The average recombination rate found for helmeted honeyeater (1.83 cM/Mb [SD 2.9]) is similar to that in zebra finch (1.3 cM/Mb [SD 2.2]) but lower than in collared flycatcher (3.1 cM/Mb [SD 4.1]) [12, 13].

### Reconstructing demographic history

We illustrate the usefulness of our high-quality genomic resources by estimating the historical effective population size (*N*_e_) of the helmeted honeyeater population using Pairwise sequentially Markovian coalescent (PSMC, RRID:SCR_017229) [68]. Raw Illumina reads (GenBank accession SRX6469119) were processed for alignment against the helmeted honeyeater chromosome-length genome by removing adapters and trimming poly-G tails with fastp v0.20.0 (fastp, RRID:SCR_016962) [26]. Trimmed reads were mapped to the autosomes of the genome (i.e., excluding Hi-C scaffolds 1 and 2) with BWA v0.7.17 (BWA, RRID:SCR_010910) [58], and mapped reads were transformed and sorted with SAMtools v1.11 (SAMtools/BCFtools, RRID:SCR_005227) [59]. We produced genotype likelihoods from reads with minimum base and mapping quality score of 30 with BCFtools mpileup (BCFtools v1.9-80, RRID:SCR_005227) [59], and called a consensus sequence with BCFtools call (option -c). The consensus sequence was transformed to fastq format with vcfutils.pl vcf2fq keeping loci with read depth between 66× and 400× (average depth was 200×). We ran PSMC v0.6.5-r67 with parameter –p 4+30*2+4+6+10 and 100 bootstraps based on previous studies done for birds [69, 70]. Results were plotted assuming a generation time of 3.17 years [71] and mutation rate of 3.44 × 10^–9^ per generation (estimated for another passerine, the medium ground finch, *Geospiza fortis* [69, 72]).

**Figure 6:**
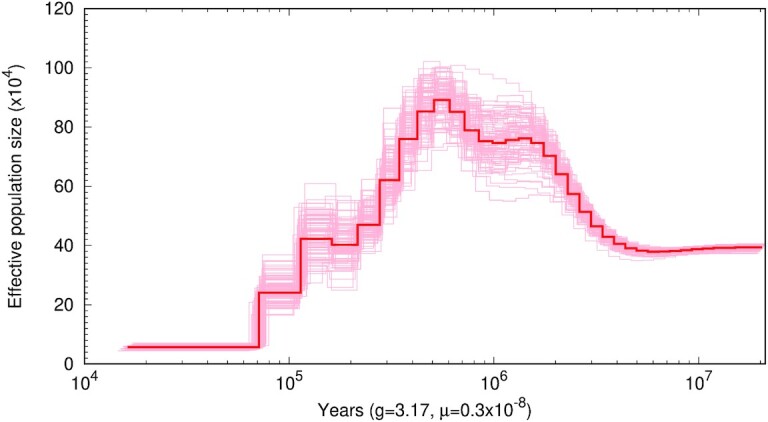
Pairwise sequentially Markovian coalescent (PSMC) reconstruction of the demographic history of the helmeted honeyeater. The red line represents the PSMC estimate and the pink lines the estimates for 100 bootstrapped sequences. The plot was constructed assuming a generation time of 3.17 years and mutation rate of 3.44 × 10^–9^ per generation.

The PSMC analysis revealed the demographic history of helmeted honeyeaters from ∼20 million years ago (Mya) to ∼20 thousand years ago (kya; Fig. 6). It suggests that the ancestral Pliocene population of *N*_e_ ∼400,000 individuals doubled from the beginning of the Pleistocene (∼2.5 Mya) until mid-Pleistocene (∼500 kya), then gradually declined, reaching ∼60,000 individuals by the Late Pleistocene (∼50 kya). The latter is generally consistent with historical *N*_e_ of 11,000 (HPD 4,000–77,000) estimated for the helmeted honeyeater based on nuclear introns [19], despite higher mutation rates used here. In combination with the previous estimate of divergence time between helmeted honeyeater and its closest relative *L. m. gippslandicus* of 56 kya (range 4–281 kya) [19], our PSMC analysis suggests that *N*_e_ decline of the helmeted honeyeater population may have started since its divergence.

## Conclusion

The helmeted honeyeater is one of few bird species for which both an annotated chromosome-length genome assembly and its associated high-density linkage map have been produced. The chromosome-length assembly and linkage map suggest a fission of the ancestral chromosome 1A into 2 chromosomes in helmeted honeyeater (chromosomes 1Aa and 1Ab), providing an insight into the evolution of the avian genome. The exceptionally high-quality genomic resources here allowed us to reconstruct the demographic history of this population, and provide an invaluable opportunity for future studies to use state-of-the-art tools to reconstruct genome-wide genealogies in order to infer mutational ages, split times, and positive selection (e.g., Relate [5]), and enable genomic monitoring of the ongoing genetic rescue of helmeted honeyeater. Future research based on these resources will also help to develop a genomic toolbox for other threatened species.

## Availability of Source Code

All scripts used in this article have been archived in Bridges Monash University research repository [73].

## Data Availability

Table 1 is a summary of all genomic resources, sample IDs, and accession numbers used in this study. The draft genome is available in NCBI GenBank under accession GCA_008360975.1, and the chromosome-length genome under accession GCA_008360975.2. Raw sequence data have been deposited in NCBI SRA under NCBI BioProject PRJNA554936 accessions SRX6469119 (Illumina NovaSeq), SRX6458354–SRX6458356 (Oxford Nanopore MinION), SAMN25688276–SAMN25688532 (DArT sequencing), and NCBI BioProject PRJNA512907 accession SRX9606522 (HiC). The contact matrices generated by aligning the Hi-C data to the genome assembly before and after the Hi-C scaffolding are available for browsing at multiple resolutions at [35]. The pedigree, annotation data, and final linkage and recombination maps have been archived in Bridges Monash University research repository [73].

## Additional Files

**Supplementary Material S1**. Neighbour-joining tree of complete mitogenomes closely matching the helmeted honeyeater mitogenome (*Lichenostomus melanops* cassidix B80296) based on BLASTn analysis of the NCBI nucleotide database. A subtree of only Meliphagidae and its sister clade comprising Pardalotidae and Acanthizidae is shown. The scale on the figure measures divergence in substitutions per site.

**Supplementary Material S2**. Annotated mitochondrial genome of the helmeted honeyeater (GenBank accession OK189508).

**Supplementary Material S3**. Alignment of helmeted honeyeater mitochondrial genome (B80296) to the draft and chromosome-length (Hi-C) genomes using LASTZ v1.04.03.

**Supplementary Material S4**. List of proteomes used for protein library preparations for the annotation of the helmeted honeyeater chromosome-length genome.

**Supplementary Material S5**. Alignment of helmeted honeyeater Hi-C scaffolds to female zebra finch chromosomes (assembly bTaeGut2.pat.W.v2, GenBank accession GCA_008822105.2) using LASTZ v1.04.03. Forward alignments are shown in black and reverse alignments in red.

**Supplementary Material S6**. LASTZ v1.04.03 output and pivot table of the alignment of helmeted honeyeater Hi-C scaffolds to male zebra finch chromosome 16 (assembly bTaeGut1.pri.v2, GenBank accession CM012098.1).

**Supplementary Material S7**. Alignment using LASTZ v1.04.03 of helmeted honeyeater. (A) Hi-C scaffold 1 to zebra finch *CHD1-Z* gene and (B) Hi-C scaffold 2 to zebra finch *CHD1-W* gene. Forward alignments are shown in black and reverse alignments in red.

**Supplementary Material S8**. Synteny between the helmeted honeyeater Hi-C scaffolds (left) and the chromosomes of the (A) collared flycatcher genome (right) and (B) superb fairy-wren genome (right).

**Supplementary Material S9**. Pedigree of the population used to build the linkage map. Females are represented as circles and males as squares. Lines stretching across the pedigree link the presence of the individual in multiple locations indicating extra-pair mating. The pedigree consists of 1 large cluster and 5 smaller unrelated ones.

**Supplementary Material S10**. Trials of different LOD score limits to split markers into linkage groups (putative chromosomes). At LOD = 21 markers tend to be placed in a linkage group that corresponds to a homologous zebra finch chromosome. Black arrows indicate that high LOD scores were inadequate because they split markers that mapped to 1 zebra finch chromosome into different linkage groups. Red asterisks denote that markers that mapped to zebra finch Chr 1A are split into 2 different linkage groups as early as LOD = 13.

**Supplementary Material S11**. Trials of different LOD score limits, joining single markers into the 29 linkage groups (putative chromosomes) of the helmeted honeyeater. LOD = 13 retrieved as many singles as possible without assigning them to another putative chromosome that corresponds to a different homologous zebra finch chromosome.

**Supplementary Material S12**. Marey maps of the markers for each helmeted honeyeater putative chromosome per sex. Aberrant markers that disrupted the monotonically increasing trends of the linkage maps are shown in red and were removed from the final linkage maps.

**Supplementary Material S13**. Oxford Nanopore read coverage for problematic regions in (A) the Z chromosome, (B) chromosome 13, and (C) chromosome 26. In all cases, we found a possible breakpoint of 500 bp with no mapped reads, which gave support to the possibility of a misassembly in these chromosomes.

**Supplementary Material S14**. Recombination maps of helmeted honeyeater chromosomes 26, 27, and 28 per sex. Some regions present large negative local recombination rates. Recombination rates were calculated using a LOESS regression with span parameter of 0.2.

**Supplementary Material S15**. Recombination maps for 25 autosomes and Z chromosome of the helmeted honeyeater. Male and female maps are shown in blue and red, respectively. Recombination rates were calculated using a LOESS regression with span parameter of 0.2.

giac025_GIGA-D-21-00337_Original_Submission

giac025_GIGA-D-21-00337_Revision_1

giac025_Response_to_Reviewer_Comments_Revision_1

giac025_Reviewer_1_Report_Original_Submission Martien Groenen -- 11/18/2021 Reviewed

giac025_Reviewer_1_Report_Revision_1 Martien Groenen -- 1/25/2022 Reviewed

giac025_Reviewer_2_Report_Original_Submission Taras K Oleksyk, Ph.D. -- 11/24/2021 Reviewed

giac025_Supplemental_Files

## Abbreviations

APPRIS: annotating principal splice isoforms; BLAST: Basic Local Alignment Search Tool; bp: base pairs; BUSCO: Benchmarking Universal Single-Copy Orthologs; BWA: Burrows-Wheeler Aligner; cM: centimorgans; DArTseq: DArT sequencing; Gb: gigabase pairs; kb: kilobase pairs; kya: thousand years ago; LG: linkage group; LOD: logarithm of odds; Mb: megabase pairs; MAPQ: mapping quality; MITObim: mitochondrial baiting and iterative mapping; mRNA: messenger RNA; Mya: million years ago; NCBI: National Center for Biotechnology Information; NEB: New England Biolabs; NIH: National Institutes of Health; NSF: National Science Foundation; SNP: single-nucleotide polymorphism; SRA: Sequence Read Archive; TOGA: Tool to infer Orthologs from Genome Alignments; YNCR: Yellingbo Nature Conservation Reserve.

## Competing Interests

The authors declare that they have no competing interests.

## Funding

This work was supported by Australian Research Council Linkage Grant LP160100482 to Monash University and La Trobe University, with Partner Organizations University of Canberra, Department of Environment, Land, Water and Planning (DELWP, Victoria), Diversity Arrays Technology, Zoos Victoria, Environment, Planning & Sustainable Development Directorate (ACT Government), and Department of Biodiversity, Conservation and Attractions (Western Australia). Hi-C data for the helmeted honeyeater were created by the DNA Zoo Consortium (www.dnazoo.org). DNA Zoo is supported by Illumina, Inc.; IBM; and the Pawsey Supercomputing Center. Additional support was provided by the Helen Macpherson Smith Trust, Zoos Victoria, and the Faculty of Science (Monash University), The University of Western Australia (UWA), DNA Zoo Australia, and Holsworth Wildlife Research Endowment (Ecological Society of Australia). D.A.R. was supported by the Monash Faculty of Science Dean's Postgraduate Research Scholarship (DPRS) and Monash Faculty of Science Dean's International Postgraduate Research Scholarship (DIPRS). H.E.M. was funded by the European Union's Horizon 2020 research and innovation program under Marie Skłodowska‐Curie (grant 840519). A.P. was supported by LP160100482 and Revive & Restore (Catalyst Science Fund). P.K. is supported by the University of Western Australia. E.L.A. was supported by the Welch Foundation (Q-1866), a McNair Medical Institute Scholar Award, an NIH Encyclopedia of DNA Elements Mapping Center Award (UM1HG009375), a US-Israel Binational Science Foundation Award (2019276), the Behavioral Plasticity Research Institute (NSF DBI-2021795), NSF Physics Frontiers Center Award (NSF PHY-2019745), and an NIH Centers of Excellence in Genomic Science grant (RM1HG011016-01A1).

## Authors’ Contributions

A.P., H.M.G., and P.S. were involved in the initial project conceptualization and design. A.P., P.S., and M.J.L.M. coordinated the collection of genetic samples. H.M.G. performed short-read and long-read sequencing and the *de novo* assembly of the draft genome. P.K., O.D., R.K., D.W., and E.L.A. performed the Hi-C sequencing and assembled the genome to chromosome length. E.O. and M.H. performed genome annotation. D.A.R. did the synteny analysis and constructed the linkage and recombination maps with guidance from H.E.M., A.P., P.S., R.H.C., and M.J.L.M. D.A.R. and A.P. drafted the manuscript, and all authors contributed to writing. All authors approved the final version of this manuscript for publication. P.S., A.P., M.J.L.M., and D.A.R. secured the direct funding for the project.
