# Supplementary material for: Chromosome-length genome assembly and linkage map of a critically endangered Australian bird: the helmeted honeyeater
Source: Gigascience. 2022 Mar 29;11:giac025. doi: 10.1093/gigascience/giac025 (PMC8963300; doi:10.1093/gigascience/giac025)
Supplement: giac025_Supplemental_Files [file giac025_supplemental_files.zip › Supplementary_Material_S12.pdf]

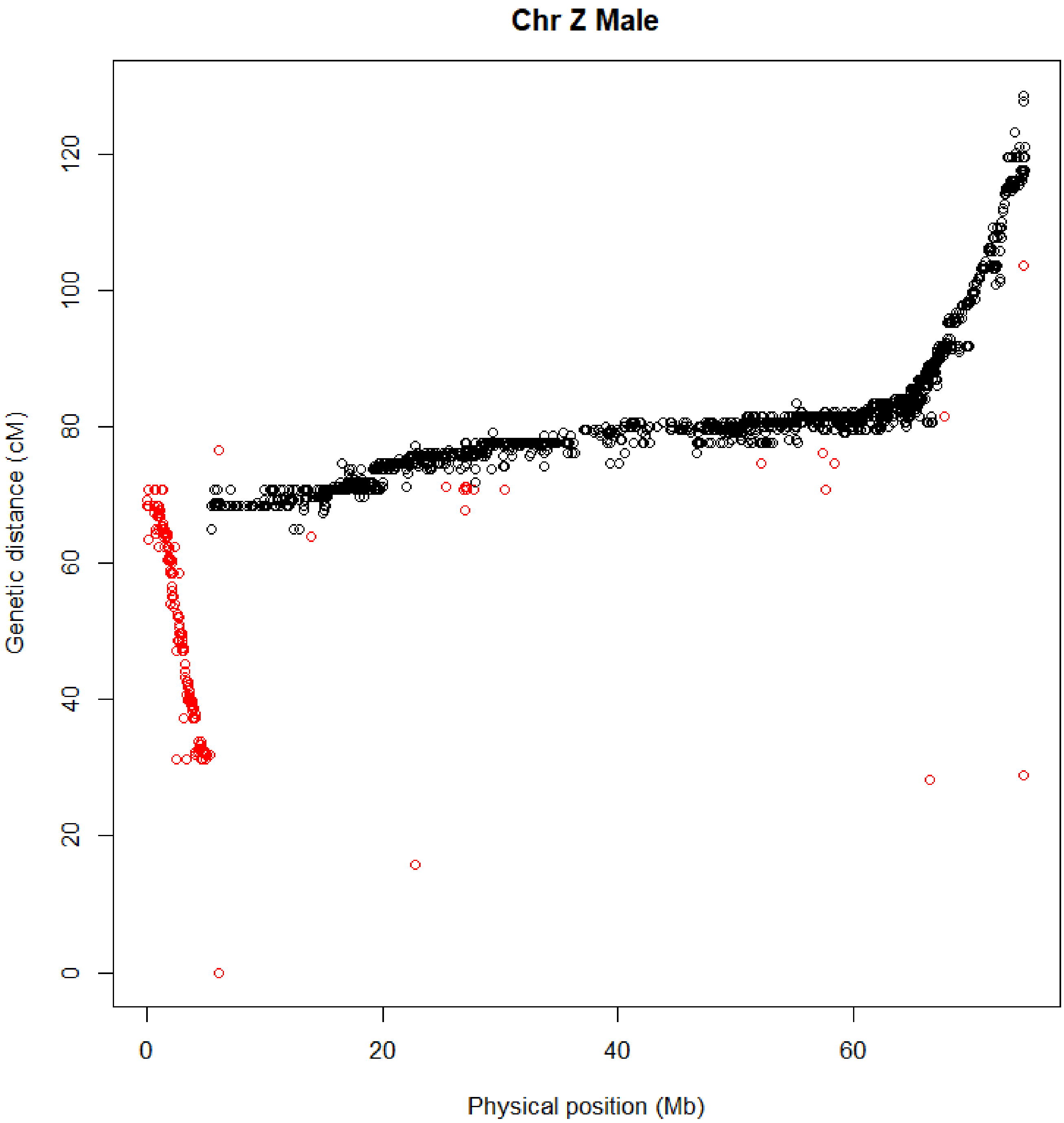

Chr Z Male
Genetic distance (cM)
0
20
40
60
80
100
120
Physical position (Mb)
0
20
40
60

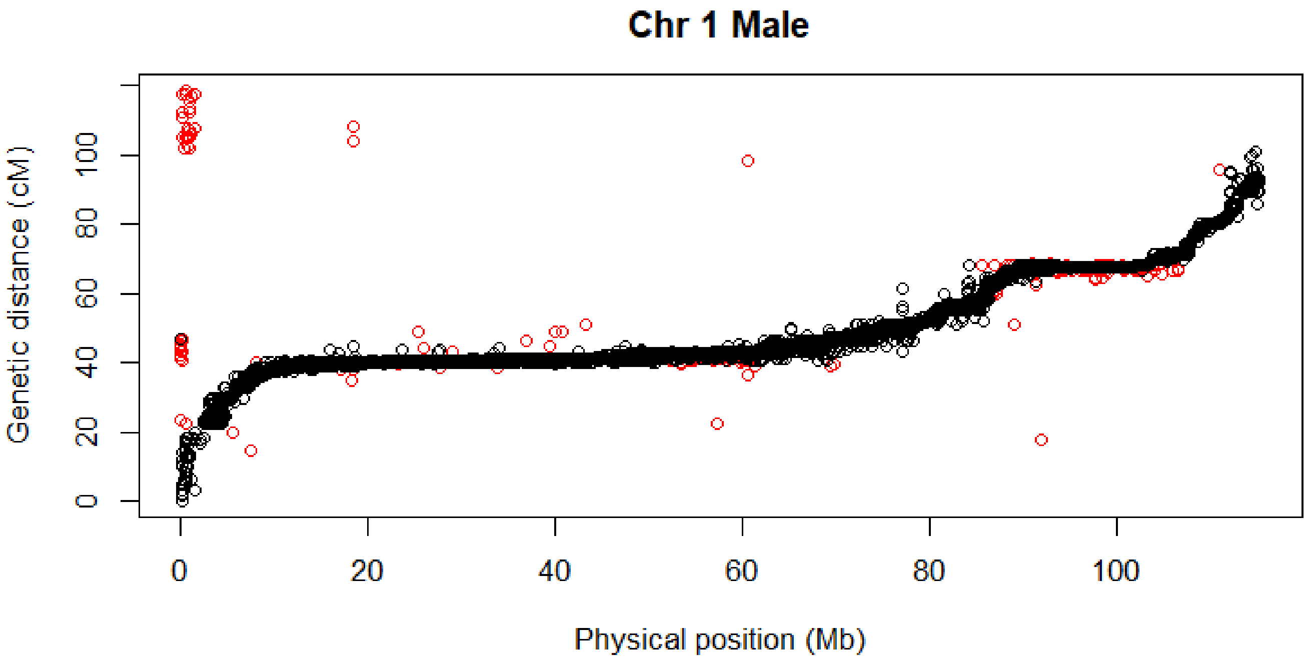
Chr 1 Male
Genetic distance (cM)
0
20
40
60
80
100
Physical position (Mb)

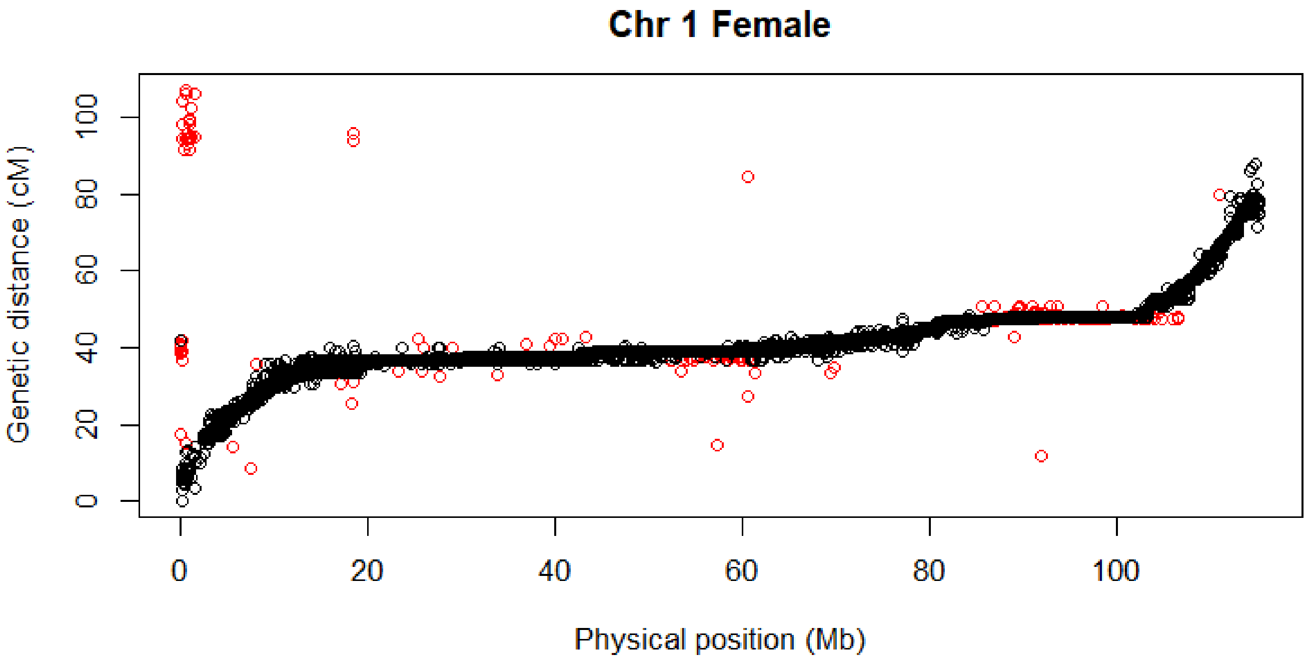
Chr 1 Female
Genetic distance (cM)
0
20
40
60
80
100
Physical position (Mb)

Chr 1Aa Male

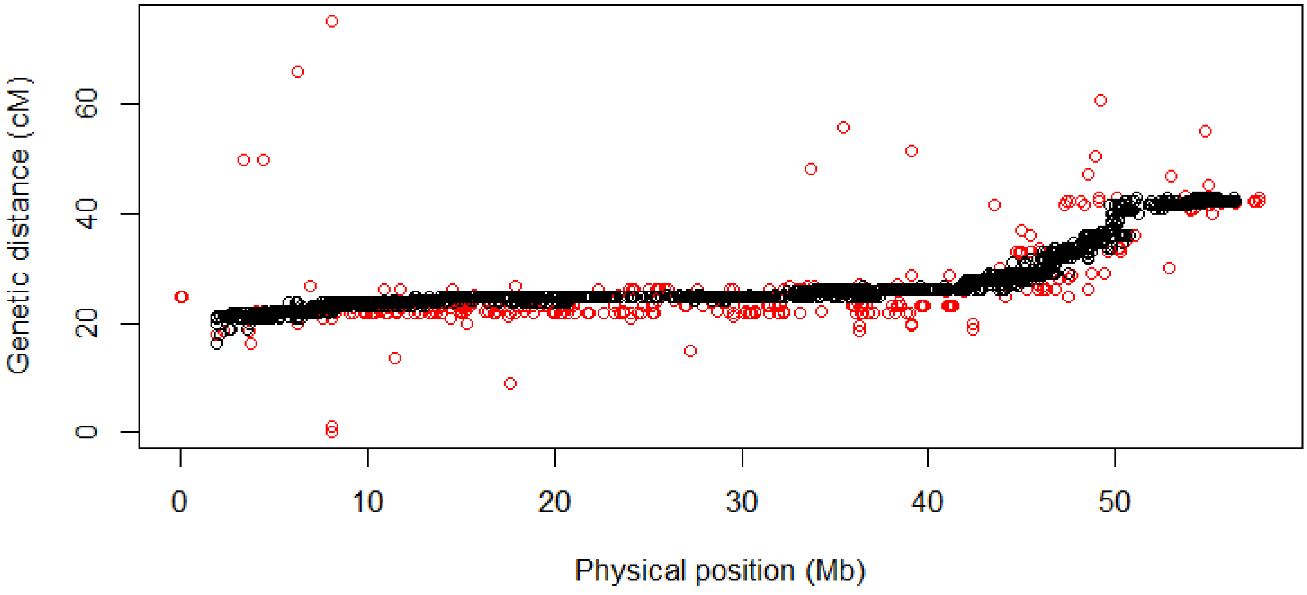

Chr 1Aa Female

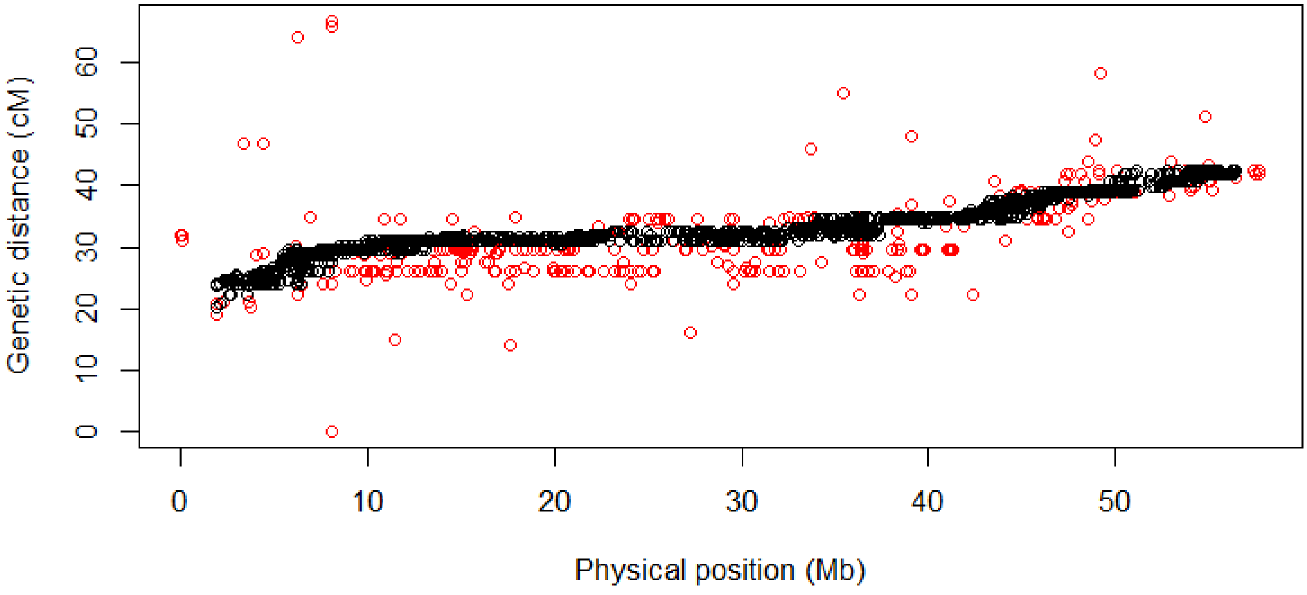

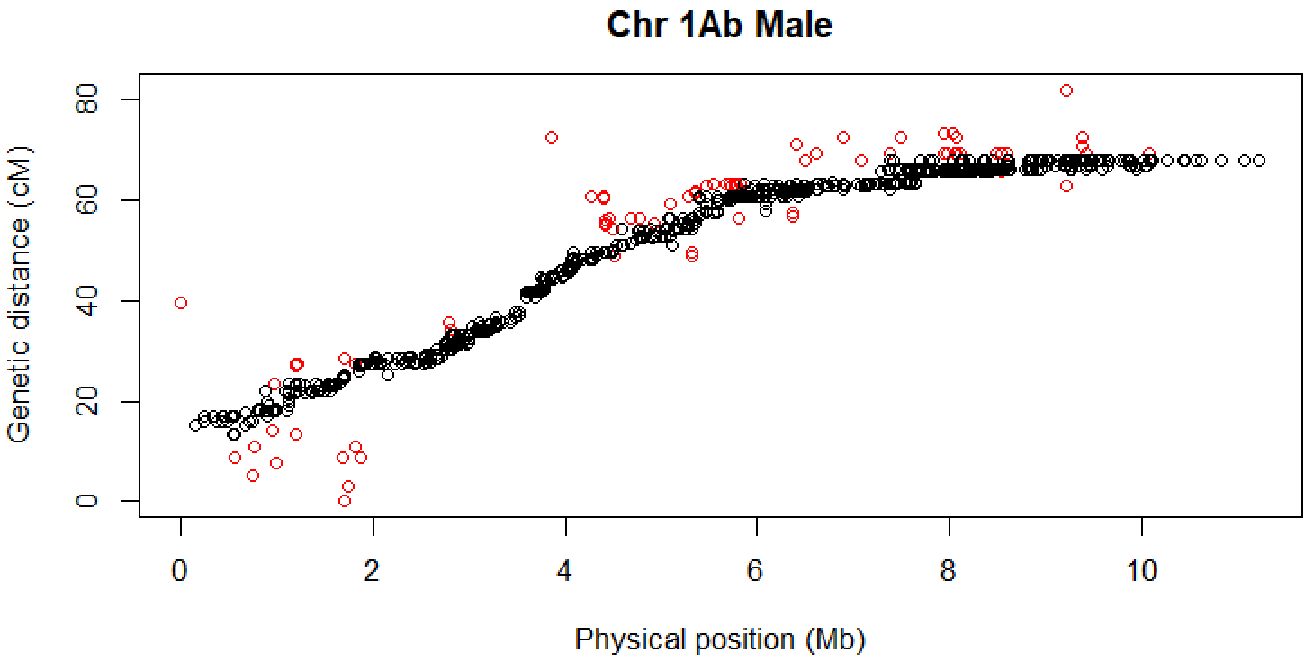

Chr 1Ab Male
Genetic distance (cM)
0
20
40
60
80
0
2
4
6
8
10
Physical position (Mb)

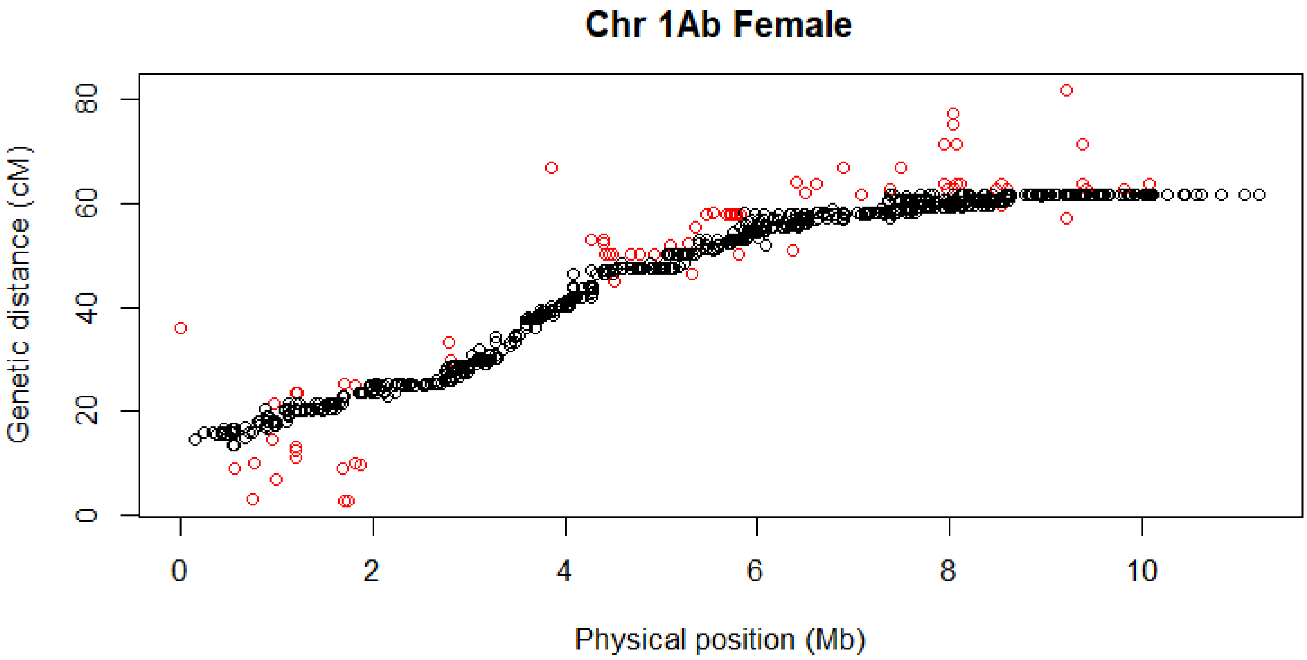

Chr 1Ab Female
Genetic distance (cM)
0
20
40
60
80
0
2
4
6
8
10
Physical position (Mb)

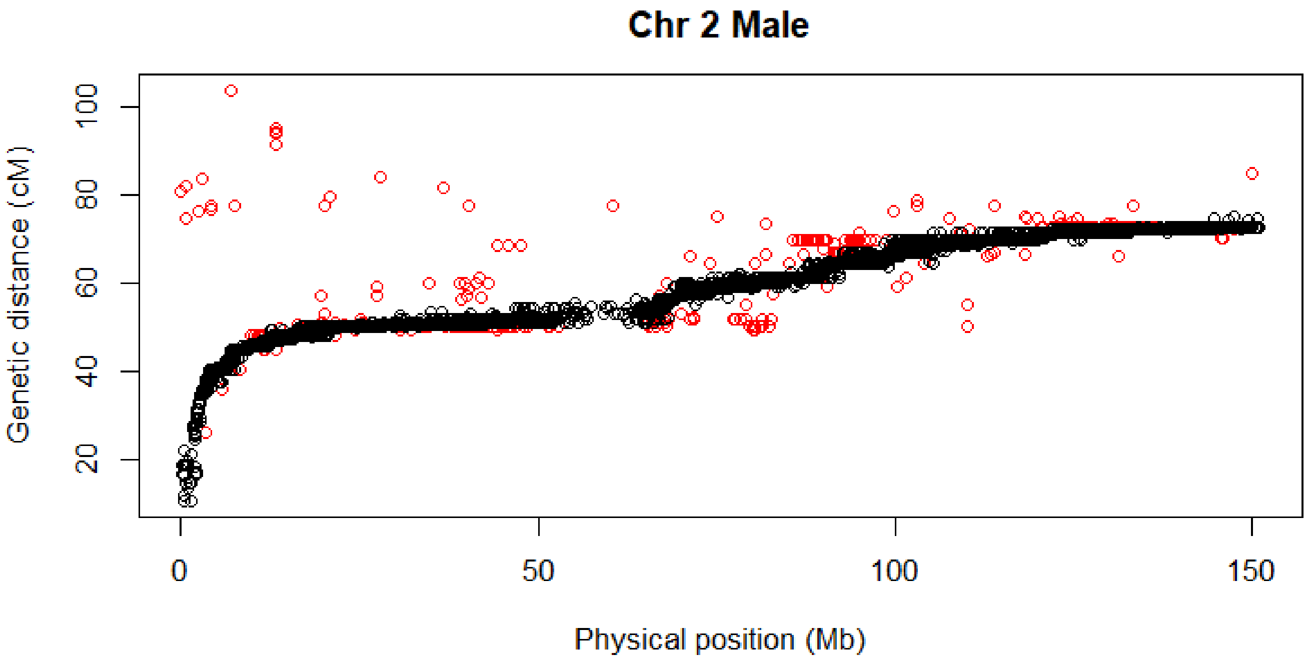
Chr 2 Male
Genetic distance (cM)
20
40
60
80
100
0
50
100
150
Physical position (Mb)

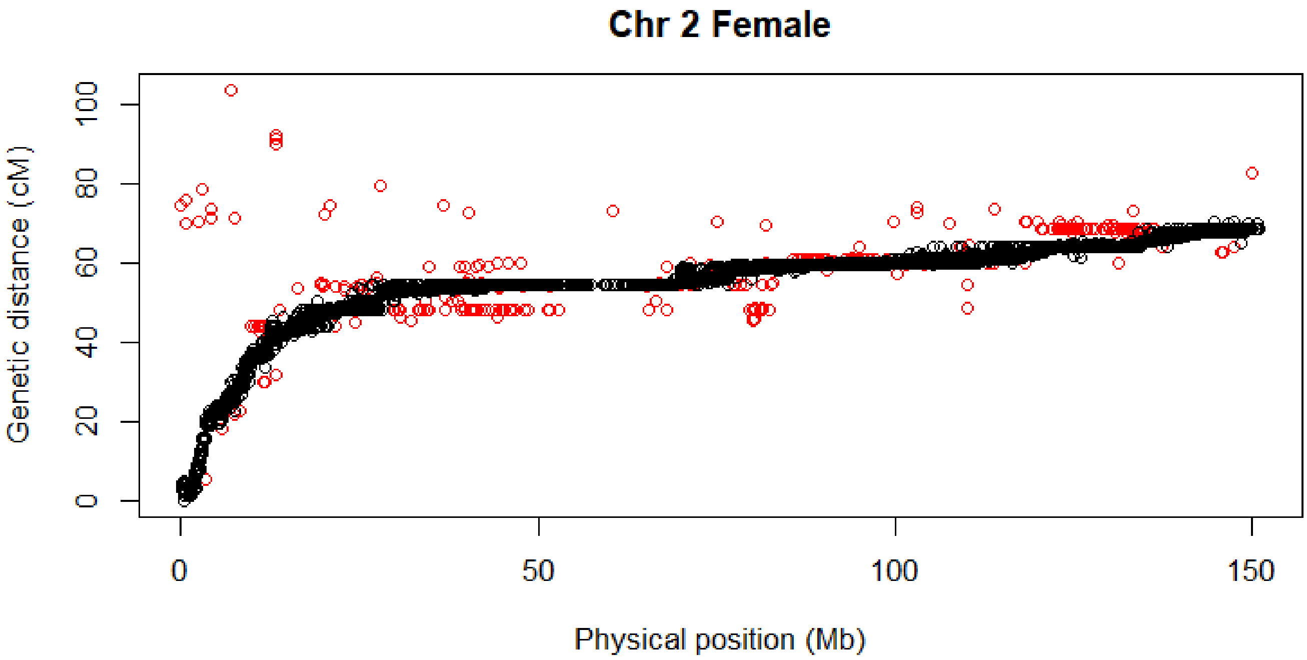
Chr 2 Female
Genetic distance (cM)
0
20
40
60
80
100
0
50
100
150
Physical position (Mb)

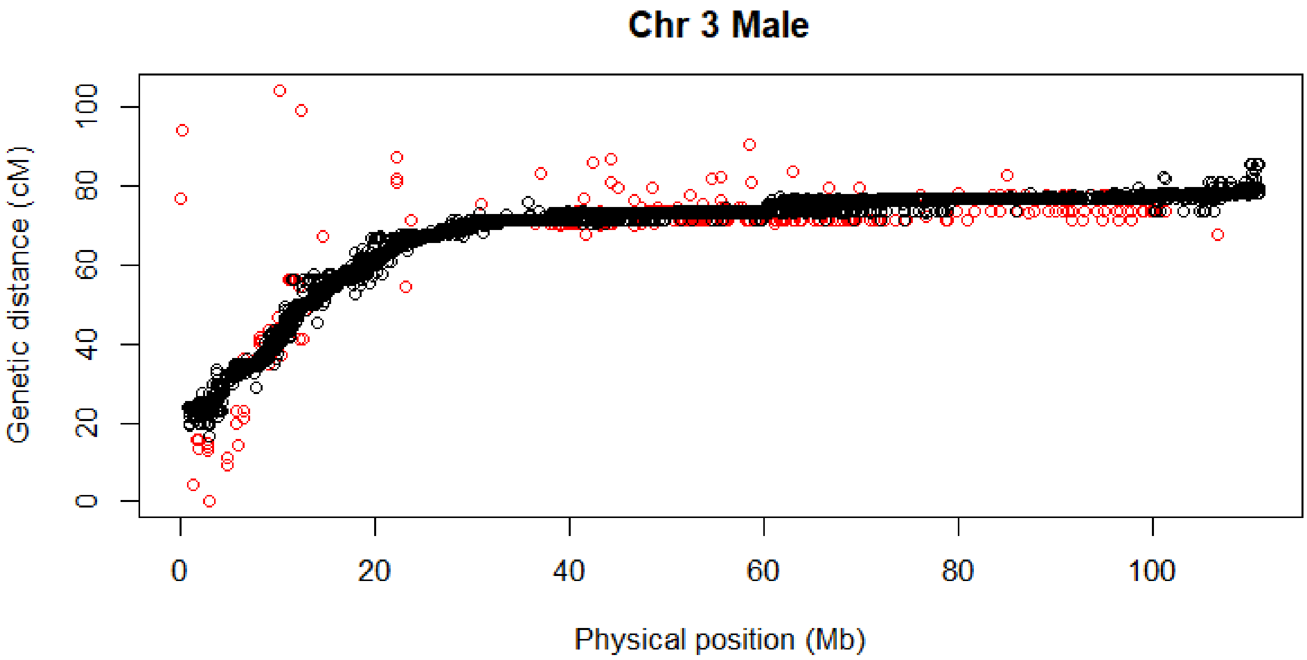

Chr 3 Male
Genetic distance (cM)
0
20
40
60
80
100
Physical position (Mb)

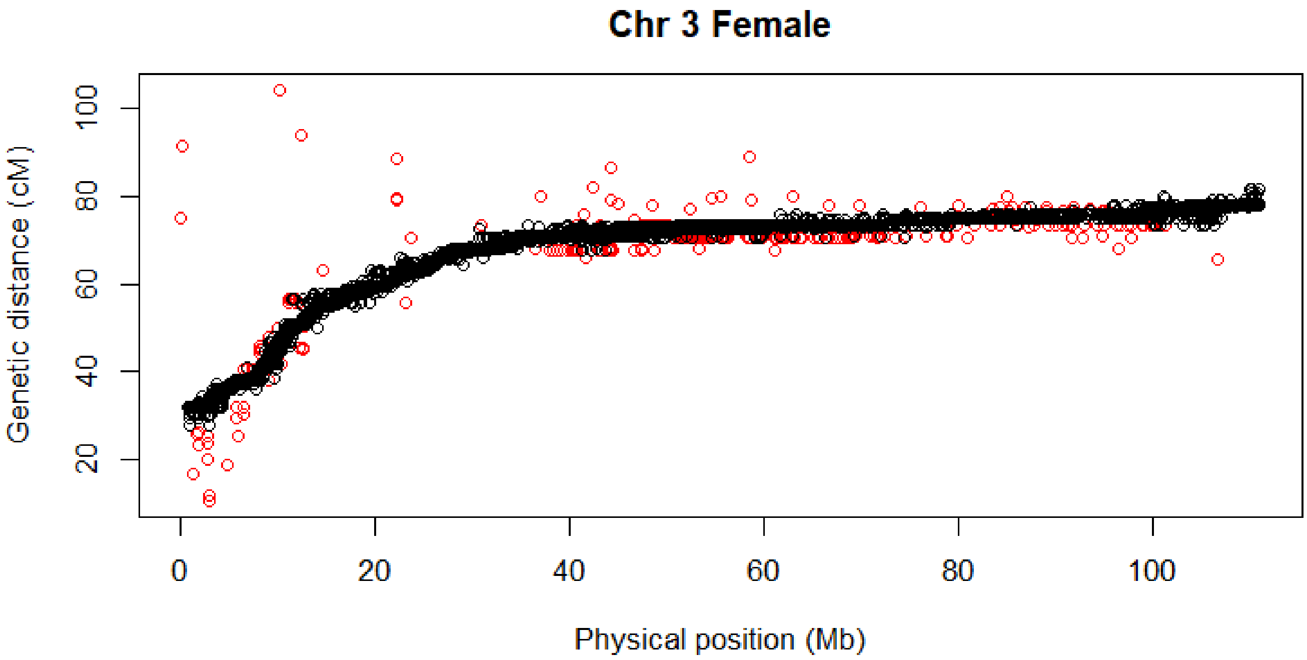

Chr 3 Female
Genetic distance (cM)
20
40
60
80
100
0
Physical position (Mb)

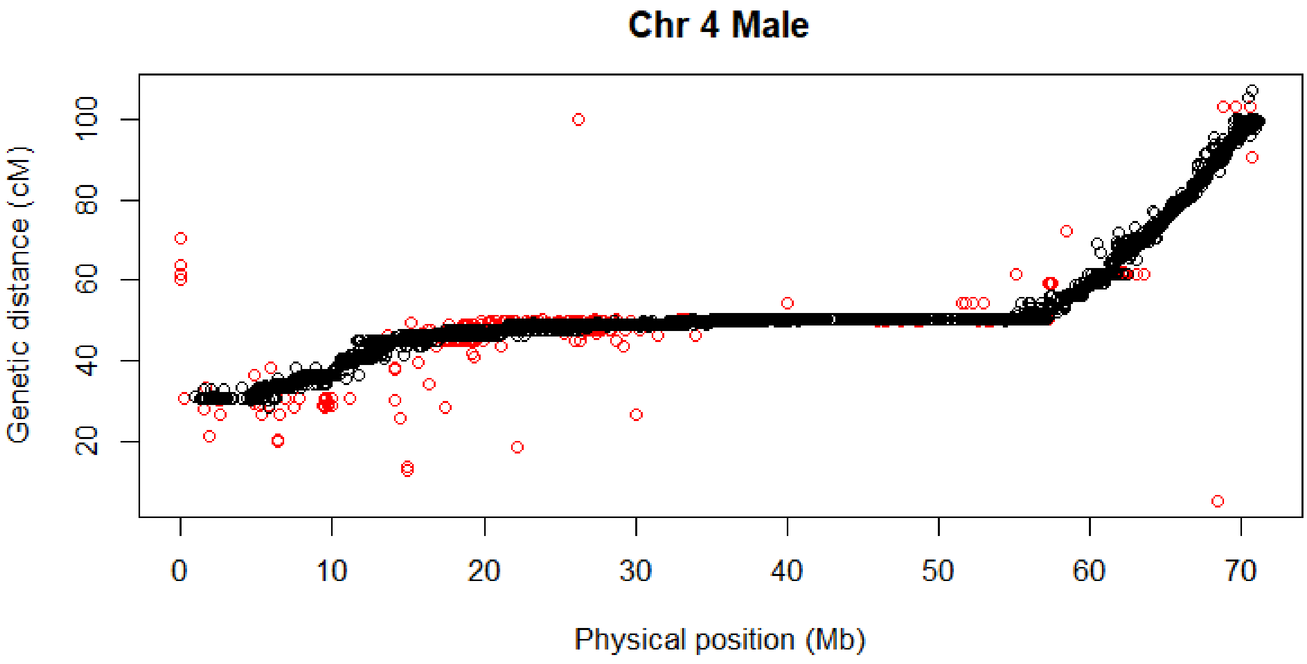

Chr 4 Male
Genetic distance (cM)
20
40
60
80
100
0
10
20
30
40
50
60
70
Physical position (Mb)

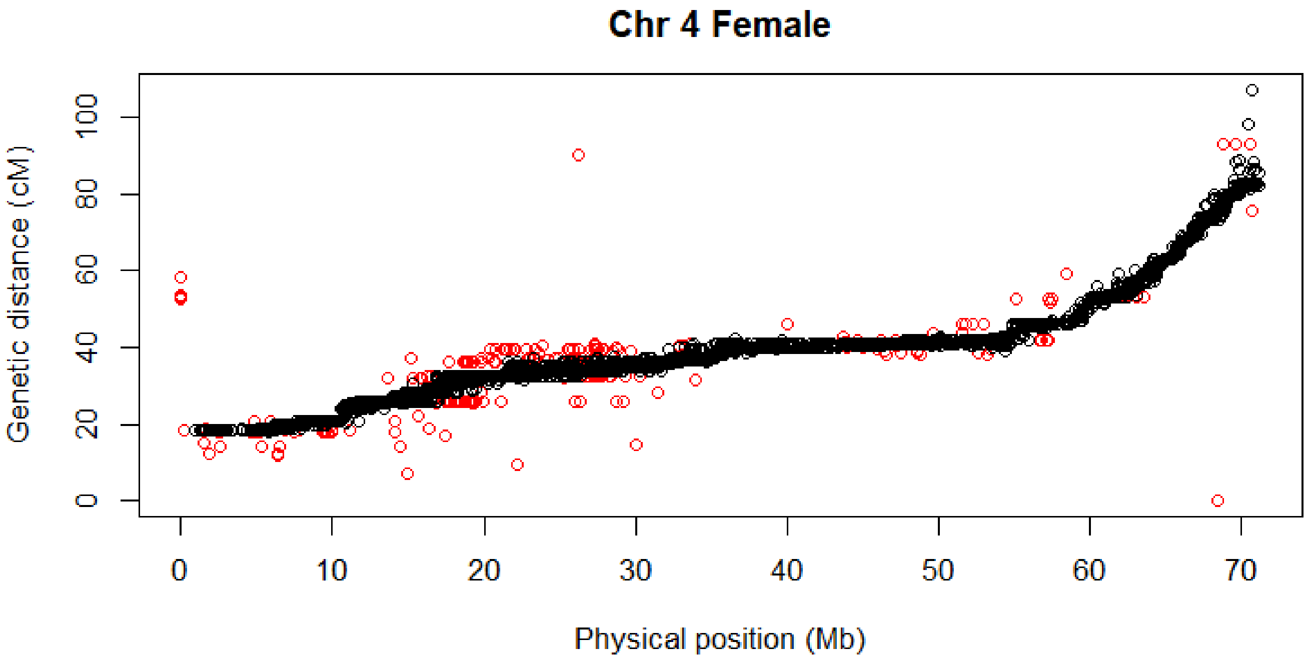

Chr 4 Female
Genetic distance (cM)
0
20
40
60
80
100
0
10
20
30
40
50
60
70
Physical position (Mb)

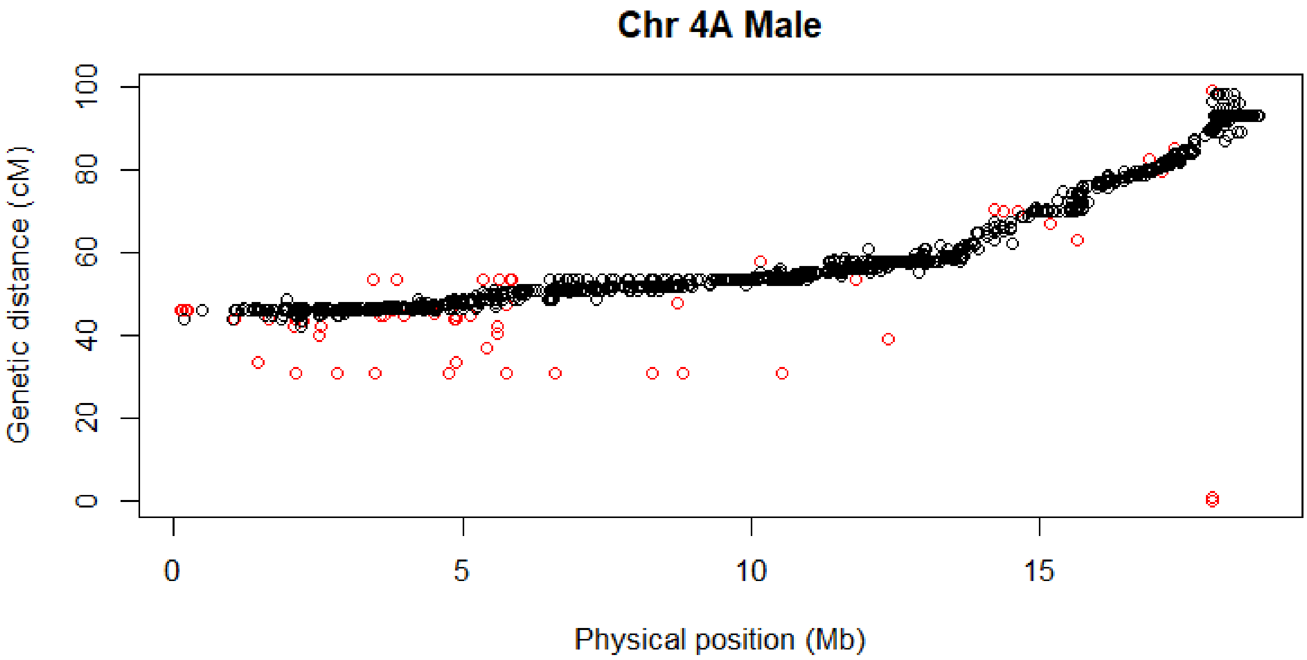
Chr 4A Male
Genetic distance (cM)
0
20
40
60
80
100
0
5
10
15
Physical position (Mb)

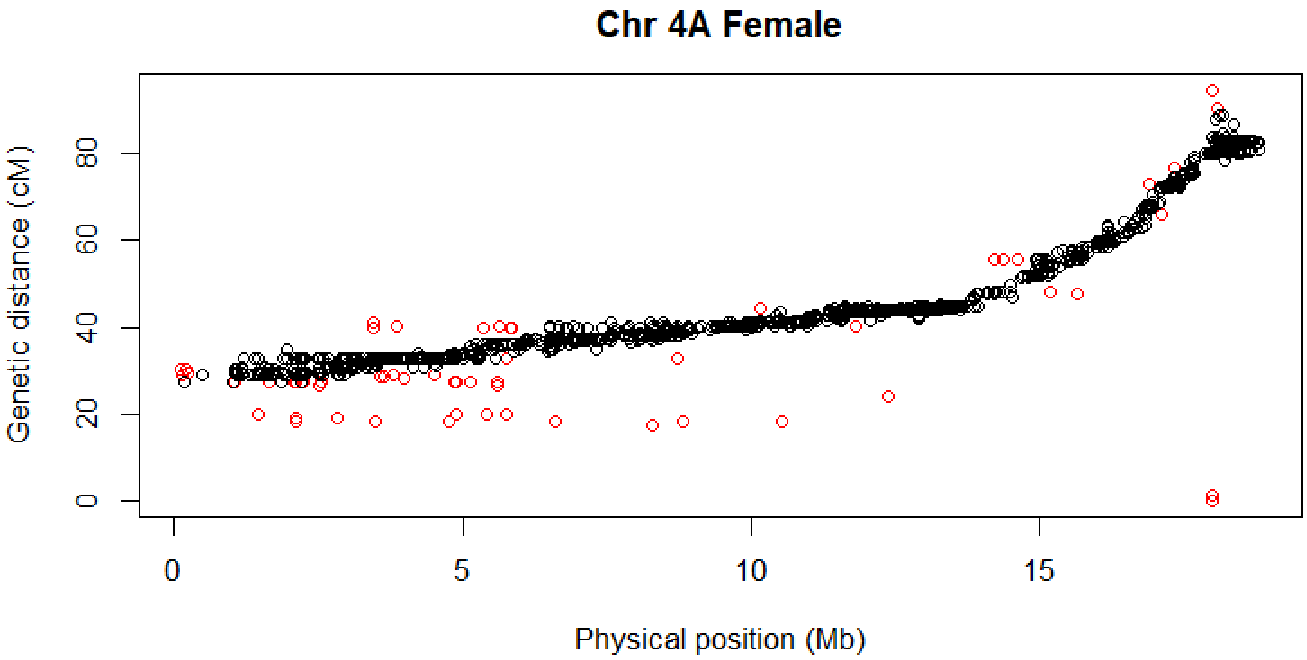
Chr 4A Female
Genetic distance (cM)
0
20
40
60
80
0
5
10
15
Physical position (Mb)

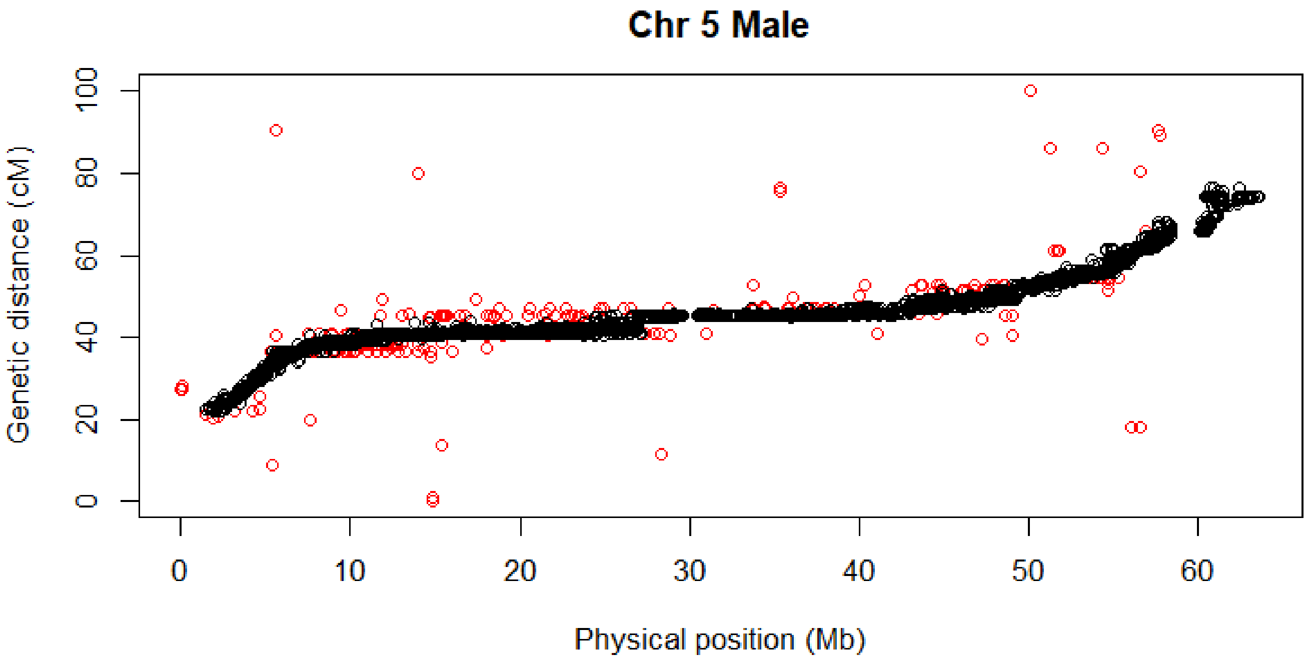
Chr 5 Male
Genetic distance (cM)
0
20
40
60
80
100
0
10
20
30
40
50
60
Physical position (Mb)

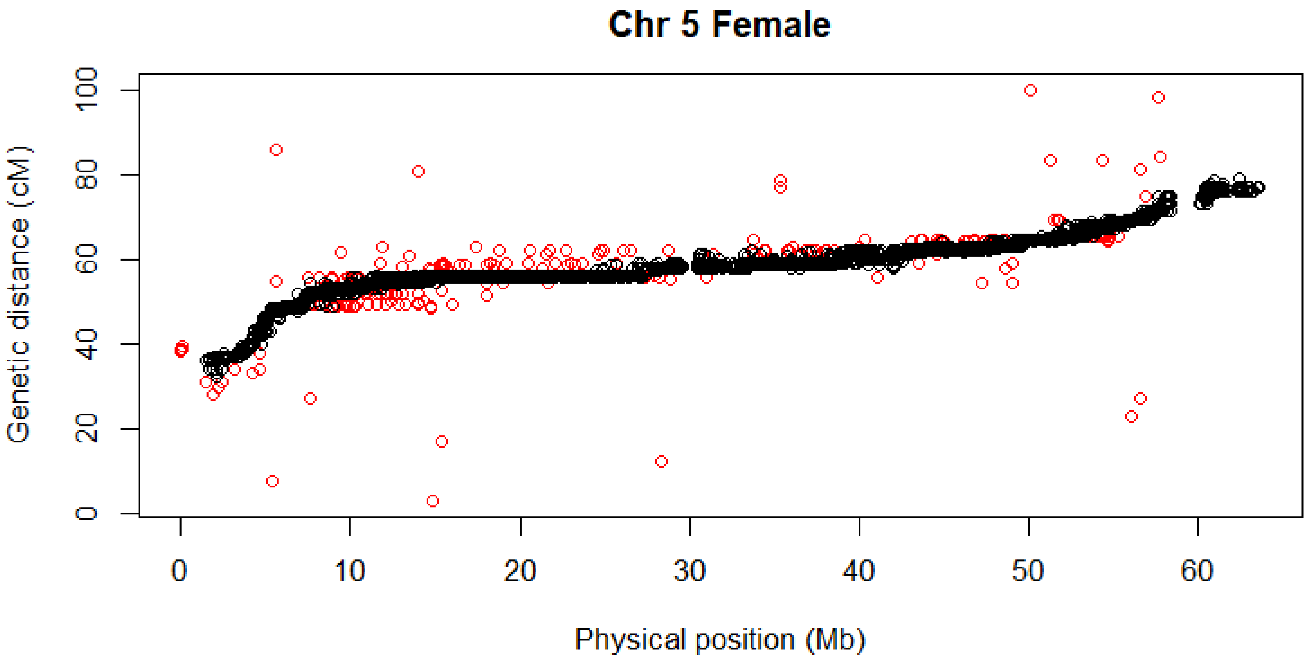
Chr 5 Female
Genetic distance (cM)
0
20
40
60
80
100
0
10
20
30
40
50
60
Physical position (Mb)

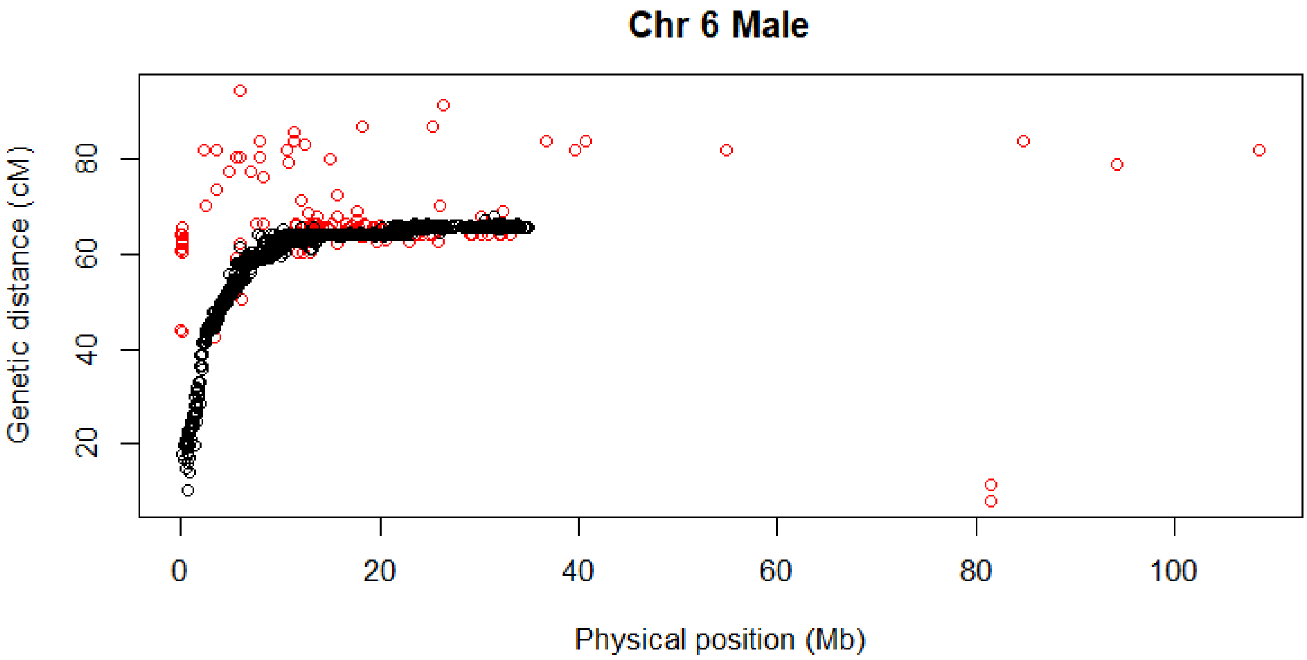
Chr 6 Male
Genetic distance (cM)
20
40
60
80
0
20
40
60
80
100
Physical position (Mb)

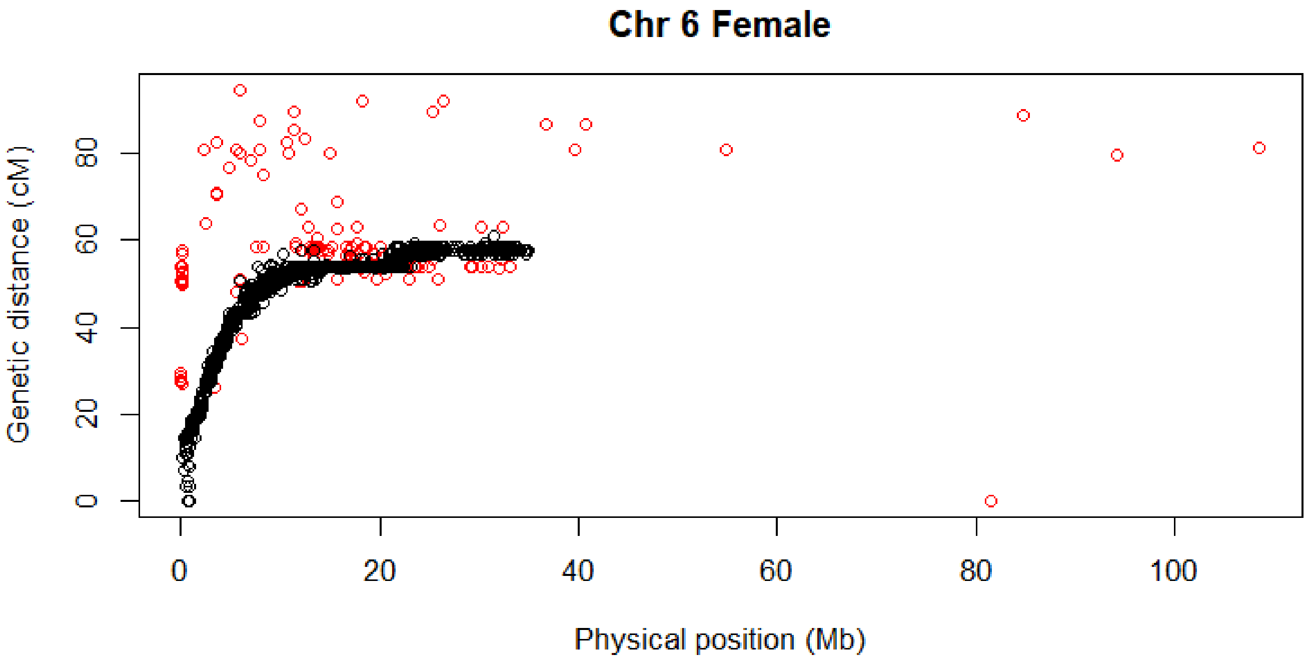
Chr 6 Female
Genetic distance (cM)
0
20
40
60
80
0
20
40
60
80
100
Physical position (Mb)

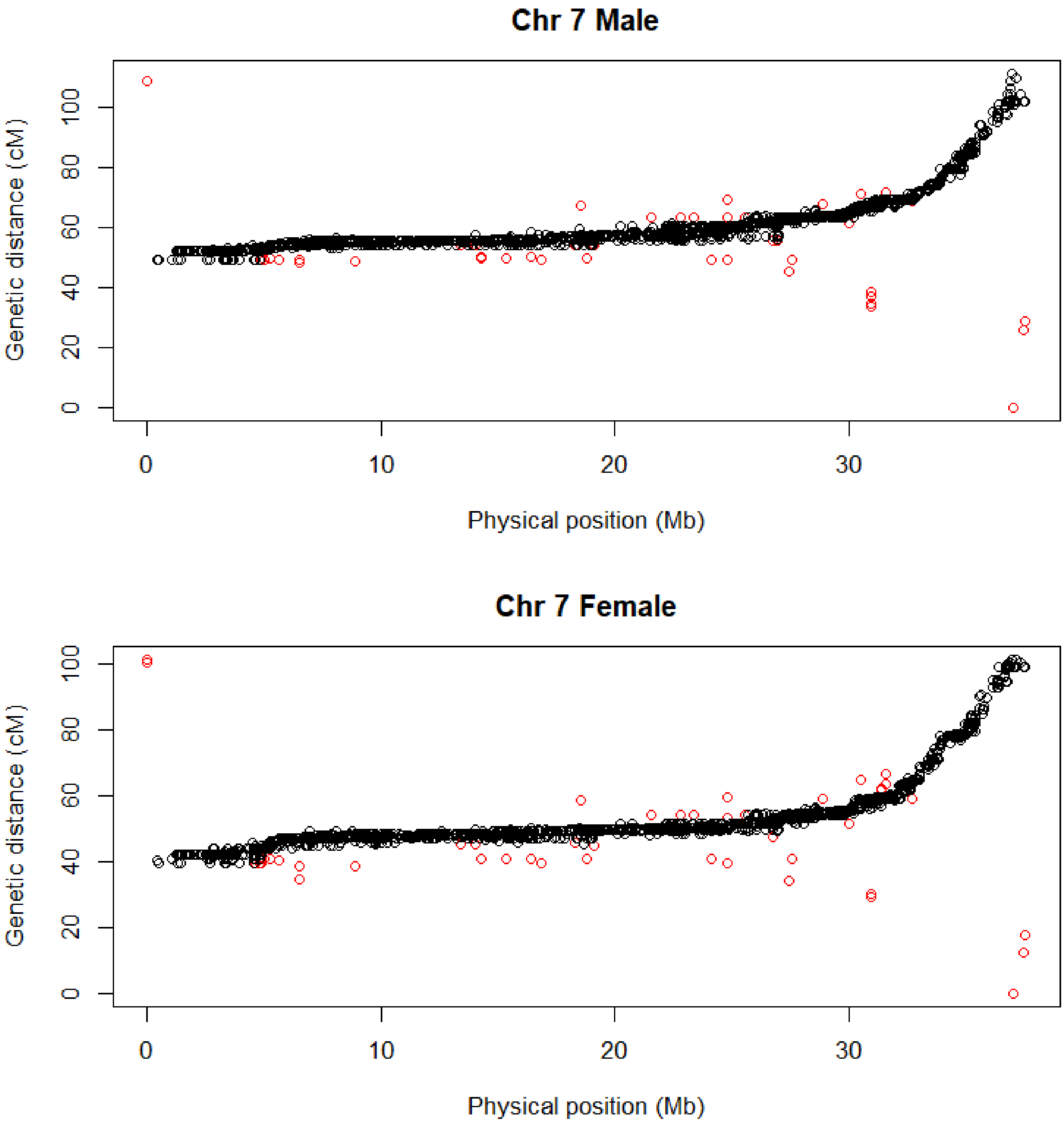
Chr 7 Male
Genetic distance (cM)
Physical position (Mb)
Chr 7 Female
Genetic distance (cM)
Physical position (Mb)

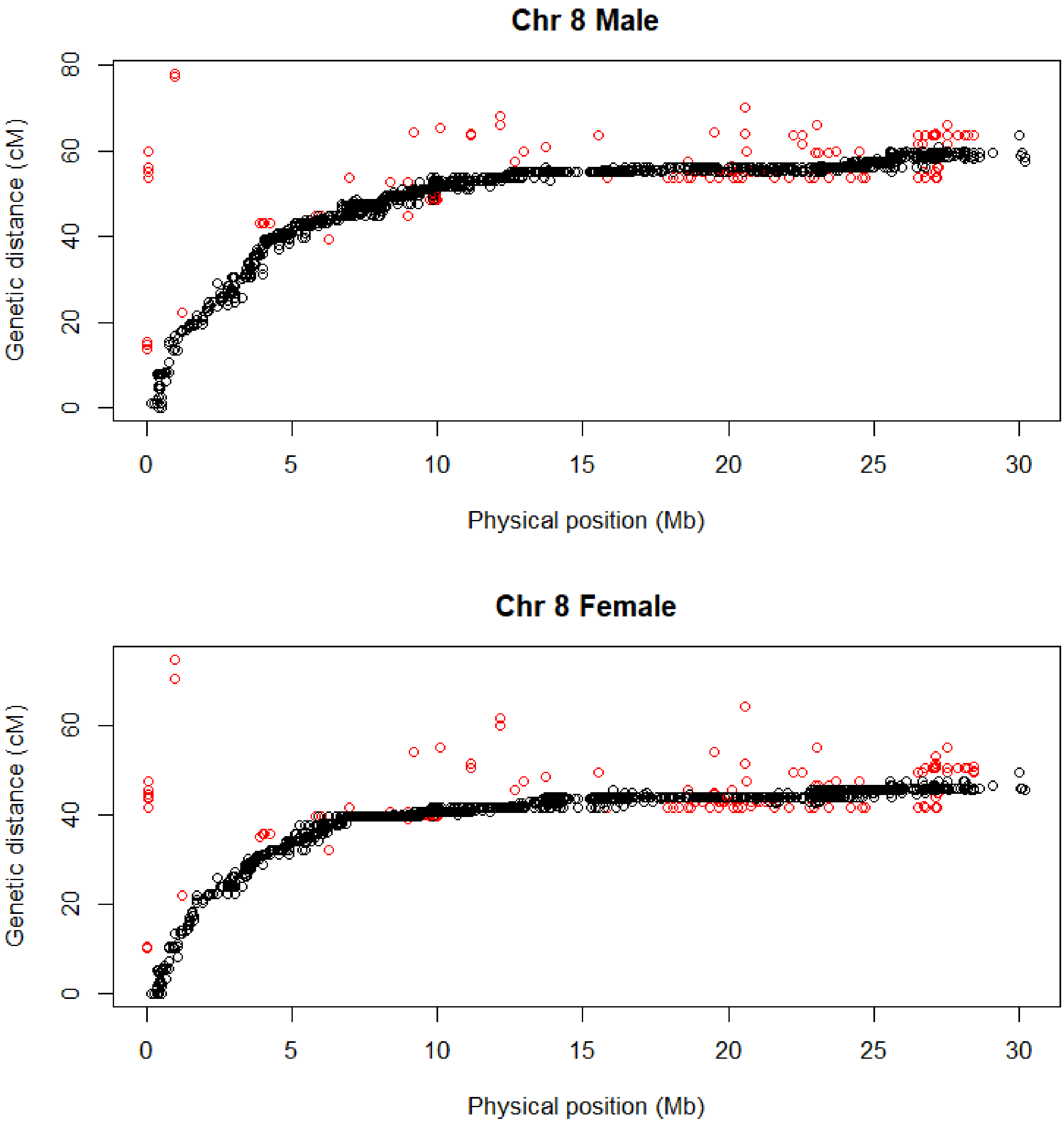

Chr 8 Male
Genetic distance (cM)
0
20
40
60
80
0
5
10
15
20
25
30
Physical position (Mb)
Chr 8 Female
Genetic distance (cM)
0
20
40
60
0
5
10
15
20
25
30
Physical position (Mb)

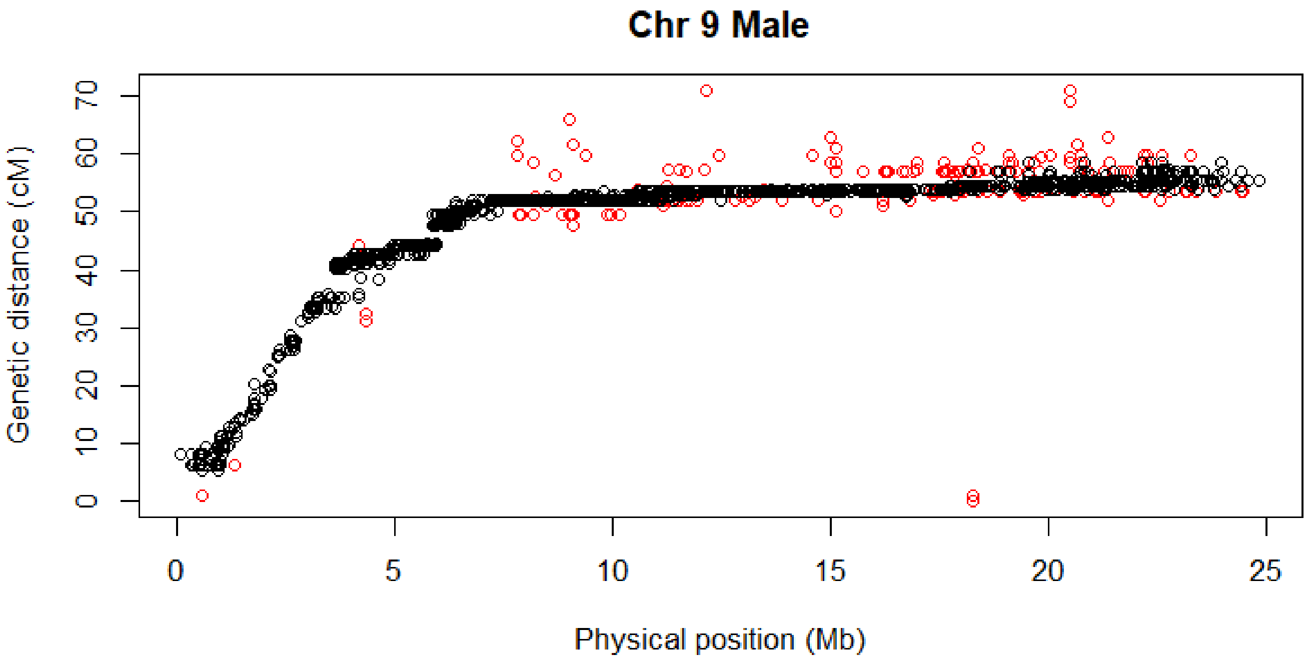
Chr 9 Male
Genetic distance (cM)
0
10
20
30
40
50
60
70
Physical position (Mb)
0
5
10
15
20
25

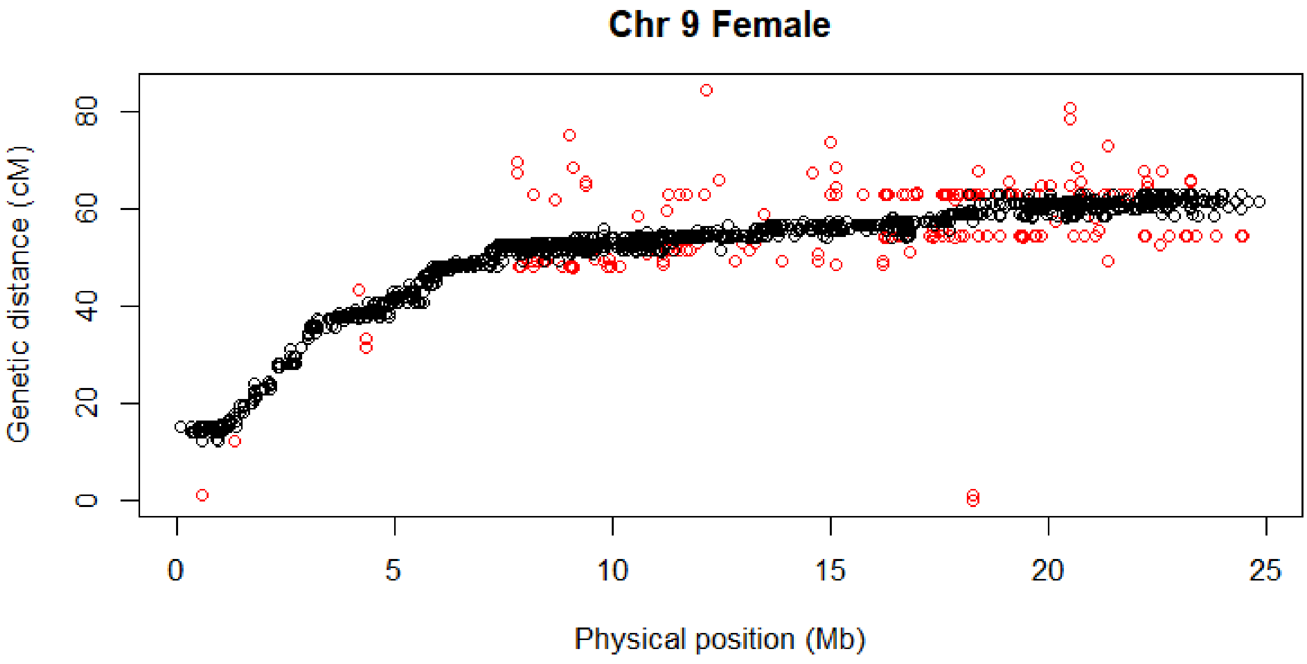
Chr 9 Female
Genetic distance (cM)
0
20
40
60
80
Physical position (Mb)
0
5
10
15
20
25

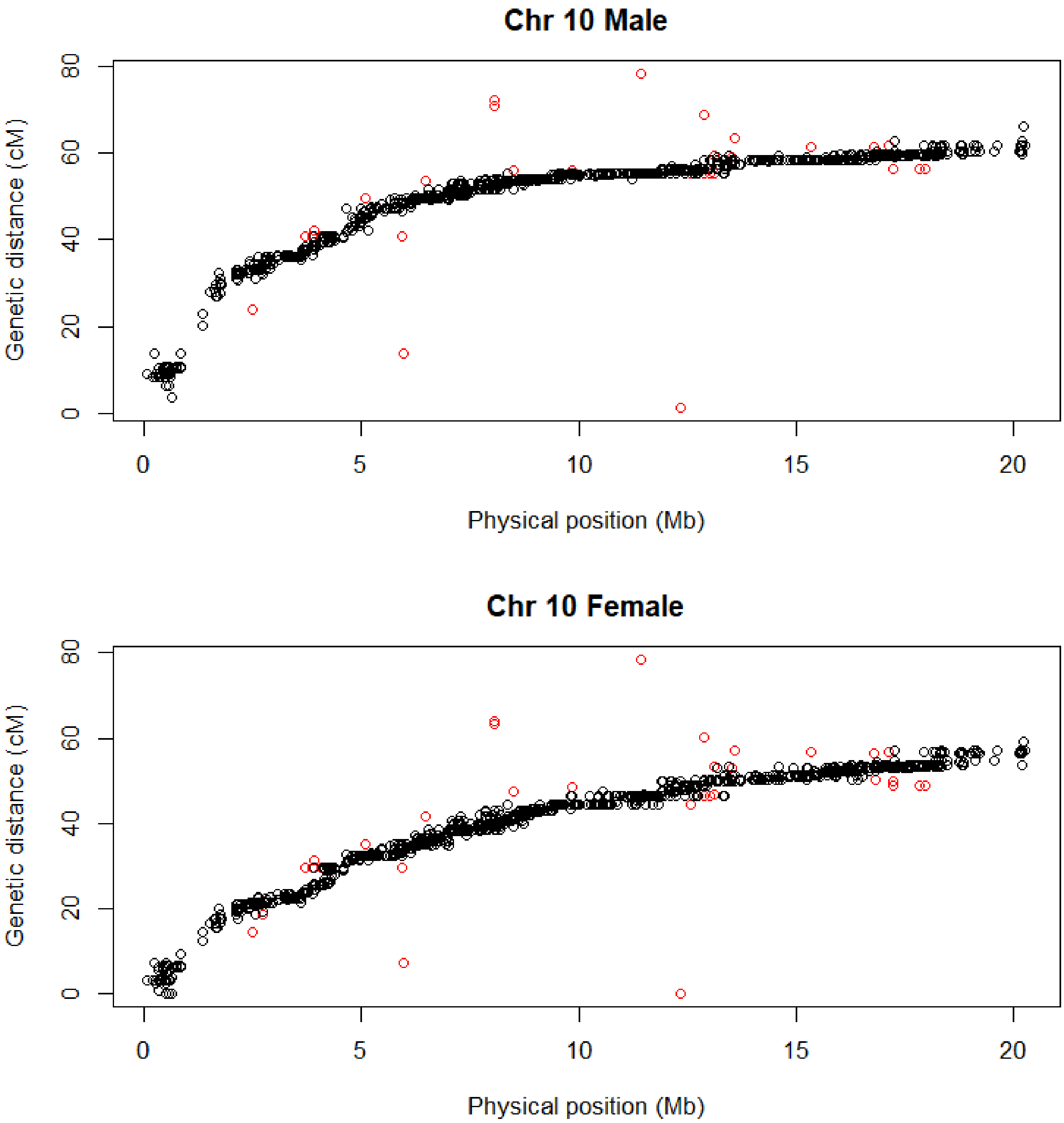
Chr 10 Male
Genetic distance (cM)
0
20
40
60
80
0
5
10
15
20
Physical position (Mb)
Chr 10 Female
Genetic distance (cM)
0
20
40
60
80
0
5
10
15
20
Physical position (Mb)

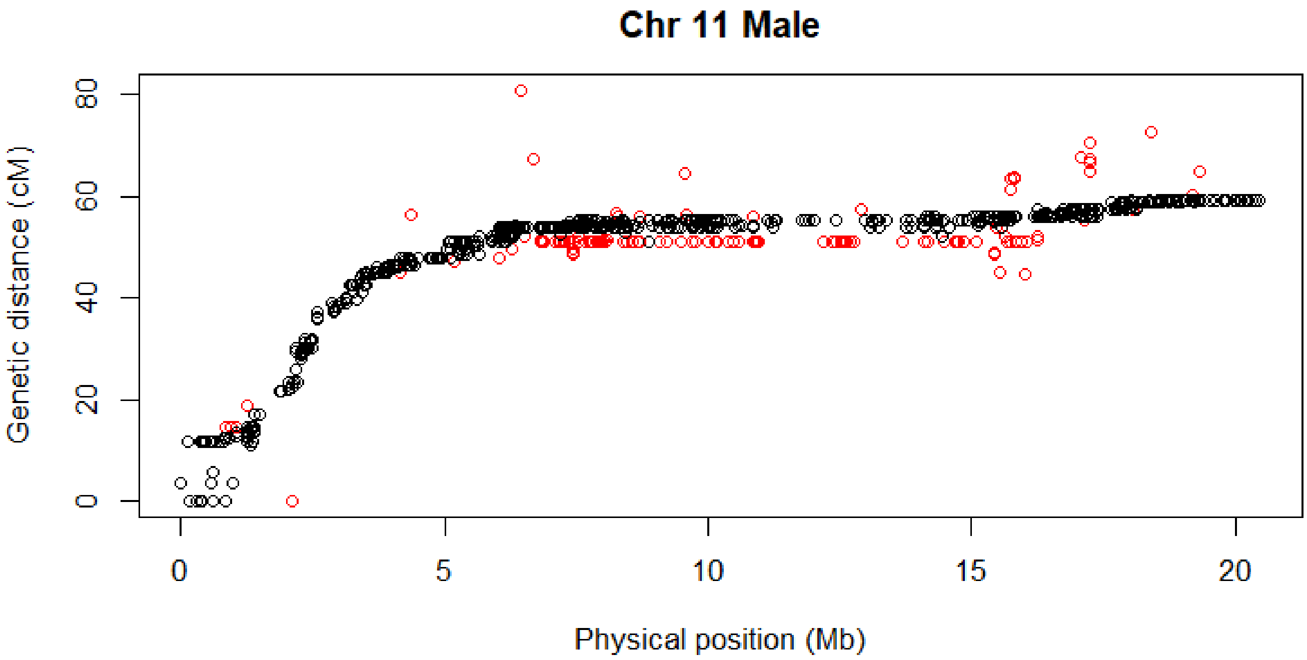

Chr 11 Male
Genetic distance (cM)
0
20
40
60
80
0
5
10
15
20
Physical position (Mb)

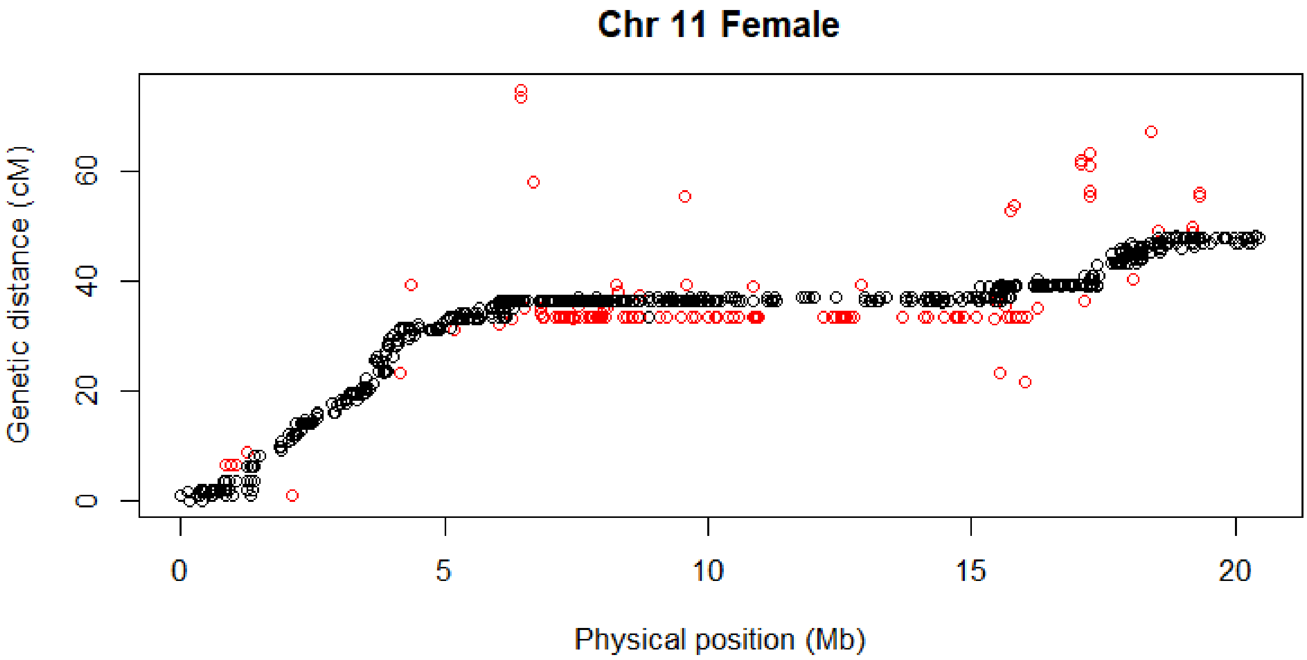

Chr 11 Female
Genetic distance (cM)
0
20
40
60
0
5
10
15
20
Physical position (Mb)

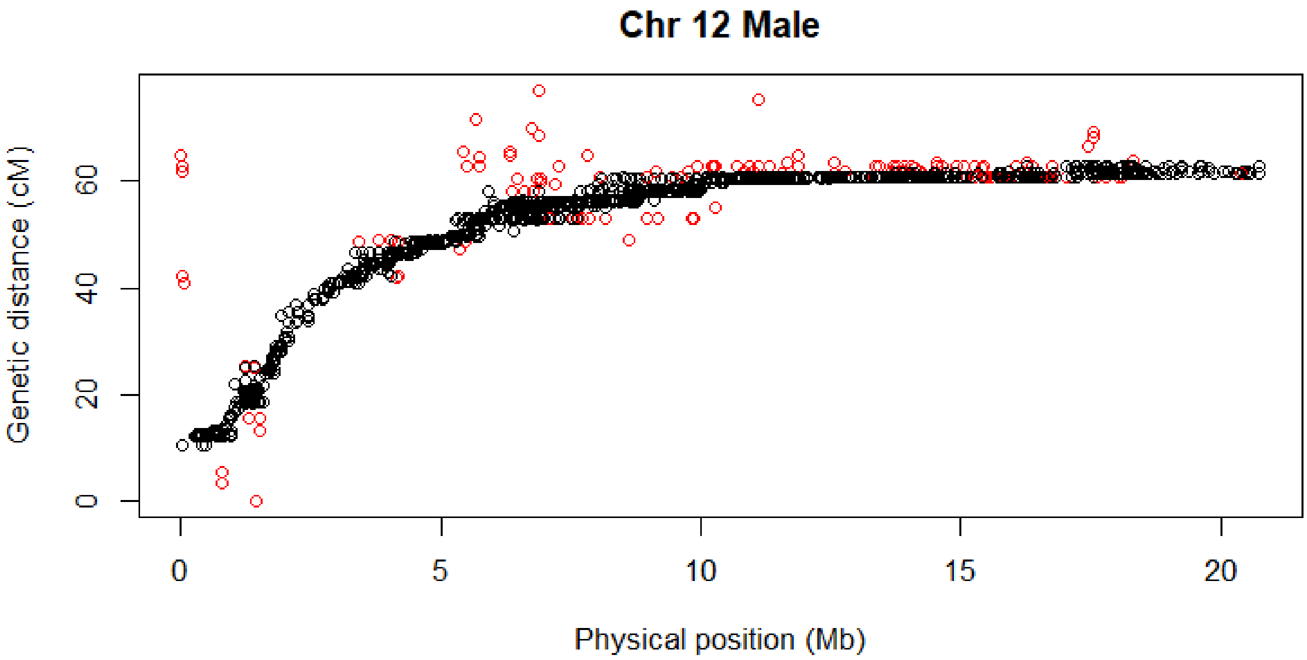
Chr 12 Male
Genetic distance (cM)
0
20
40
60
0
5
10
15
20
Physical position (Mb)

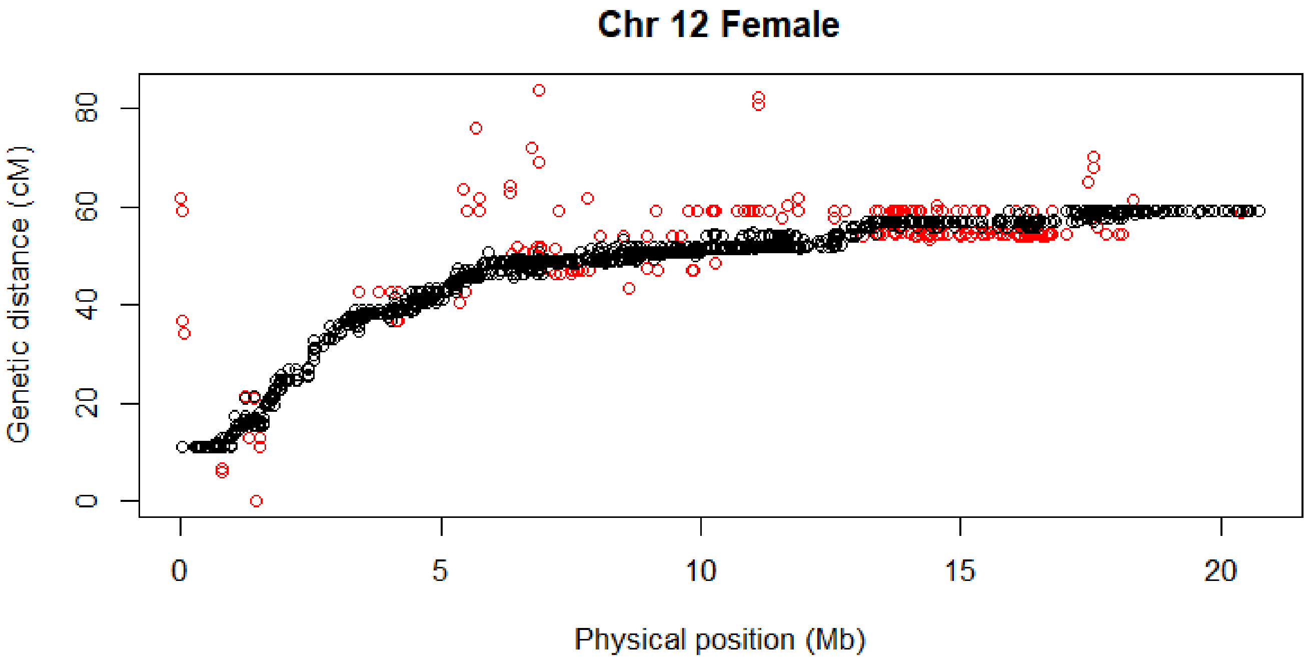
Chr 12 Female
Genetic distance (cM)
0
20
40
60
80
0
5
10
15
20
Physical position (Mb)

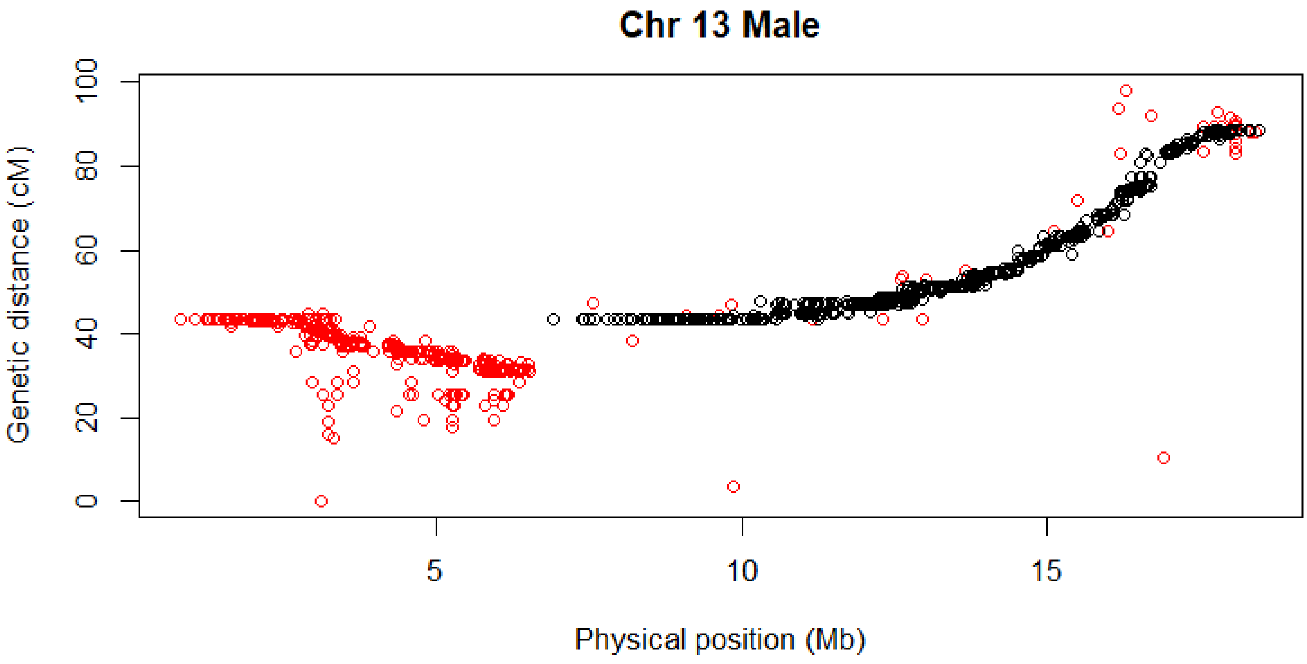

Chr 13 Male
Genetic distance (cM)
0
20
40
60
80
100
5
10
15
Physical position (Mb)

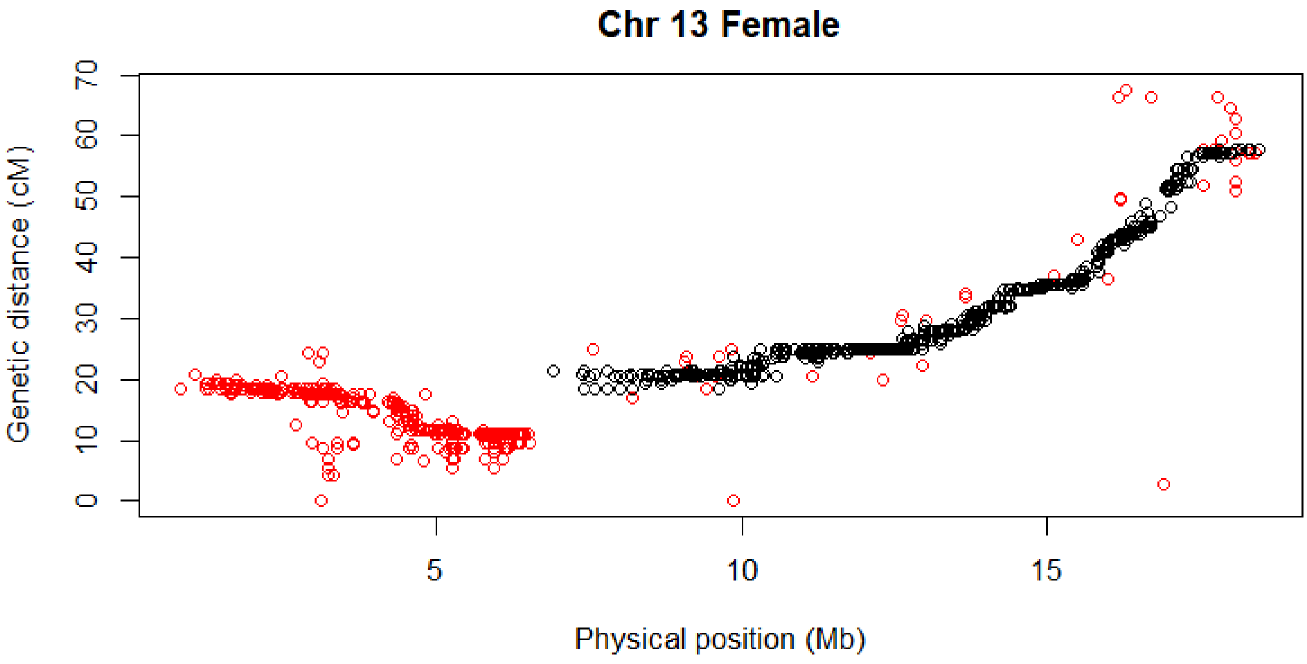

Chr 13 Female
Genetic distance (cM)
0
10
20
30
40
50
60
70
5
10
15
Physical position (Mb)

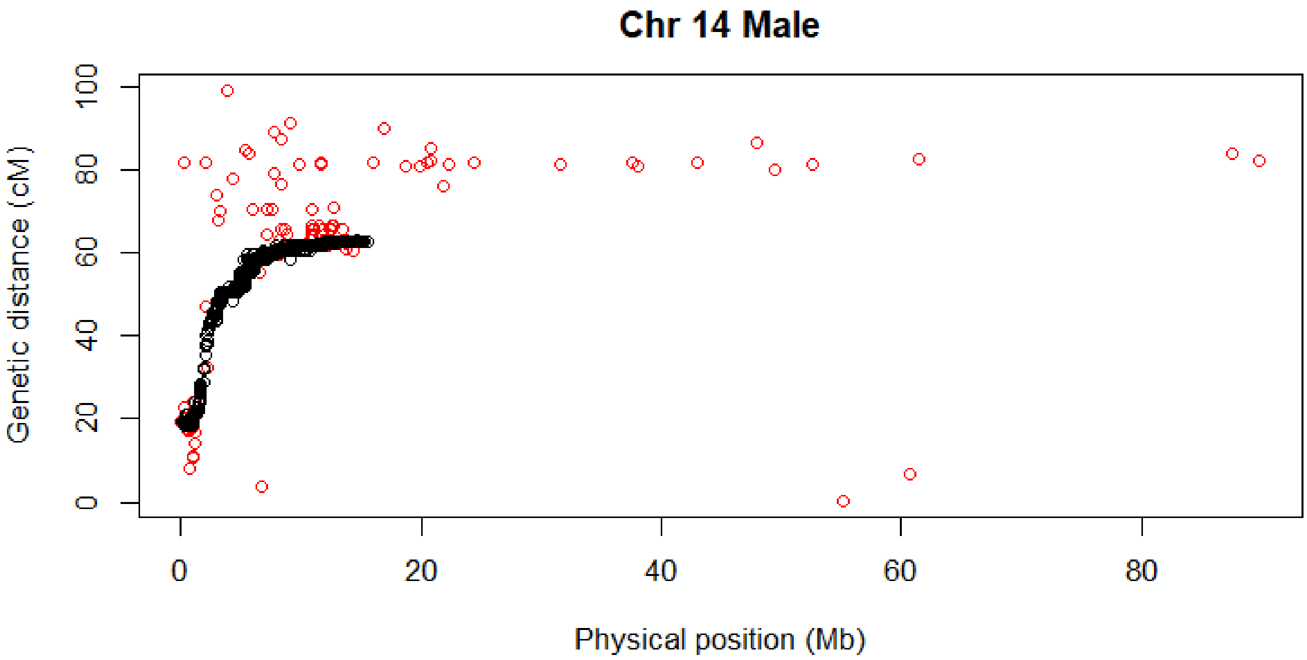
Chr 14 Male
Genetic distance (cM)
0
20
40
60
80
100
0
20
40
60
80
Physical position (Mb)

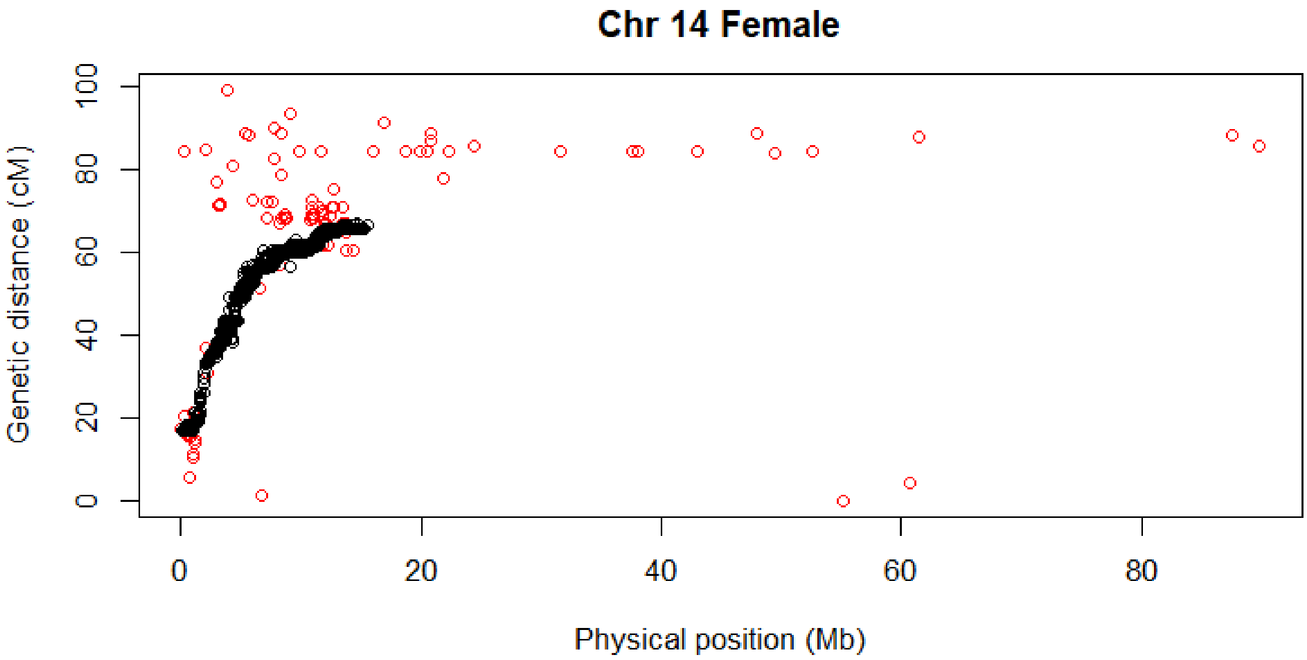
Chr 14 Female
Genetic distance (cM)
0
20
40
60
80
100
0
20
40
60
80
Physical position (Mb)

Chr 15 Male

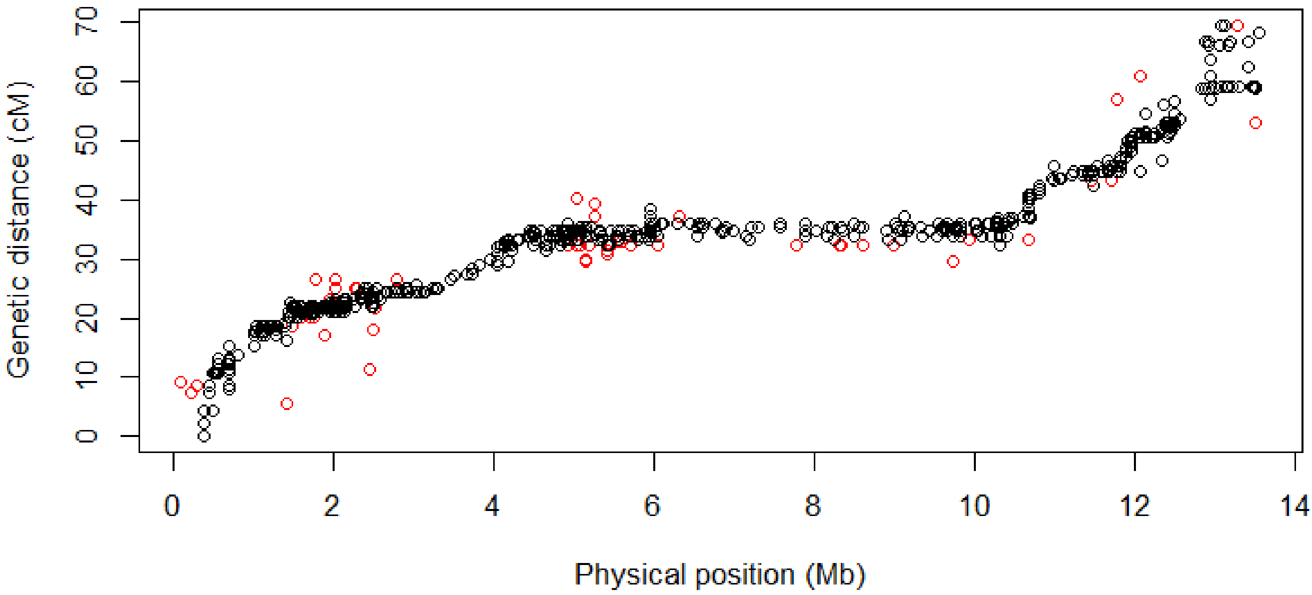

Chr 15 Female

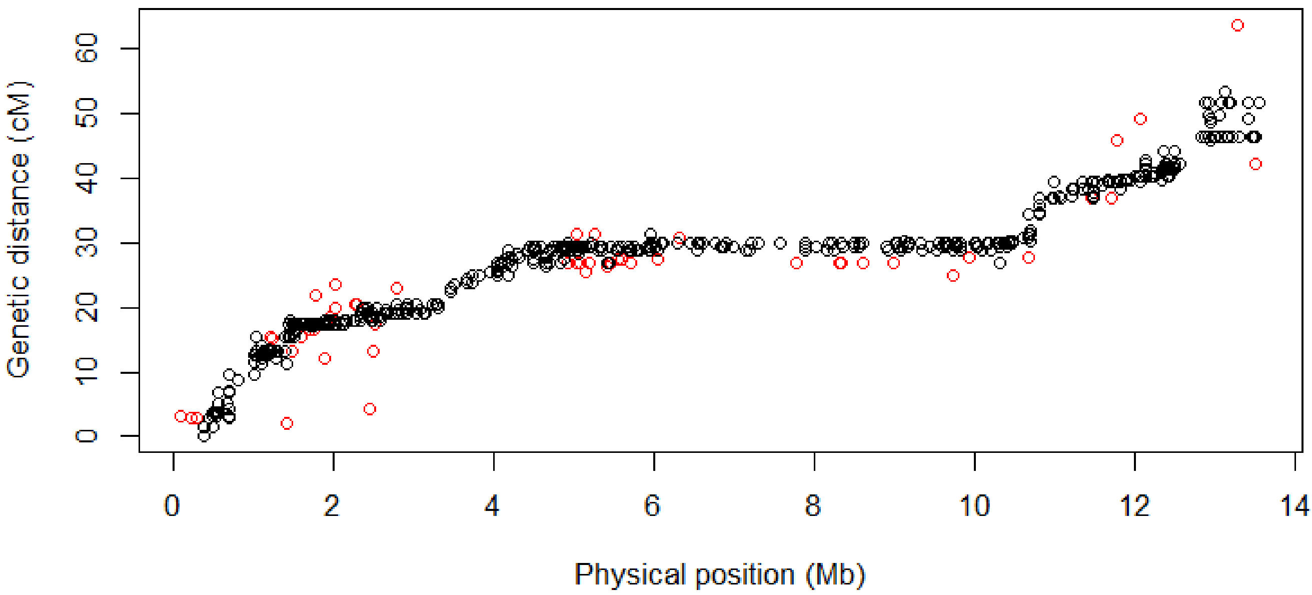

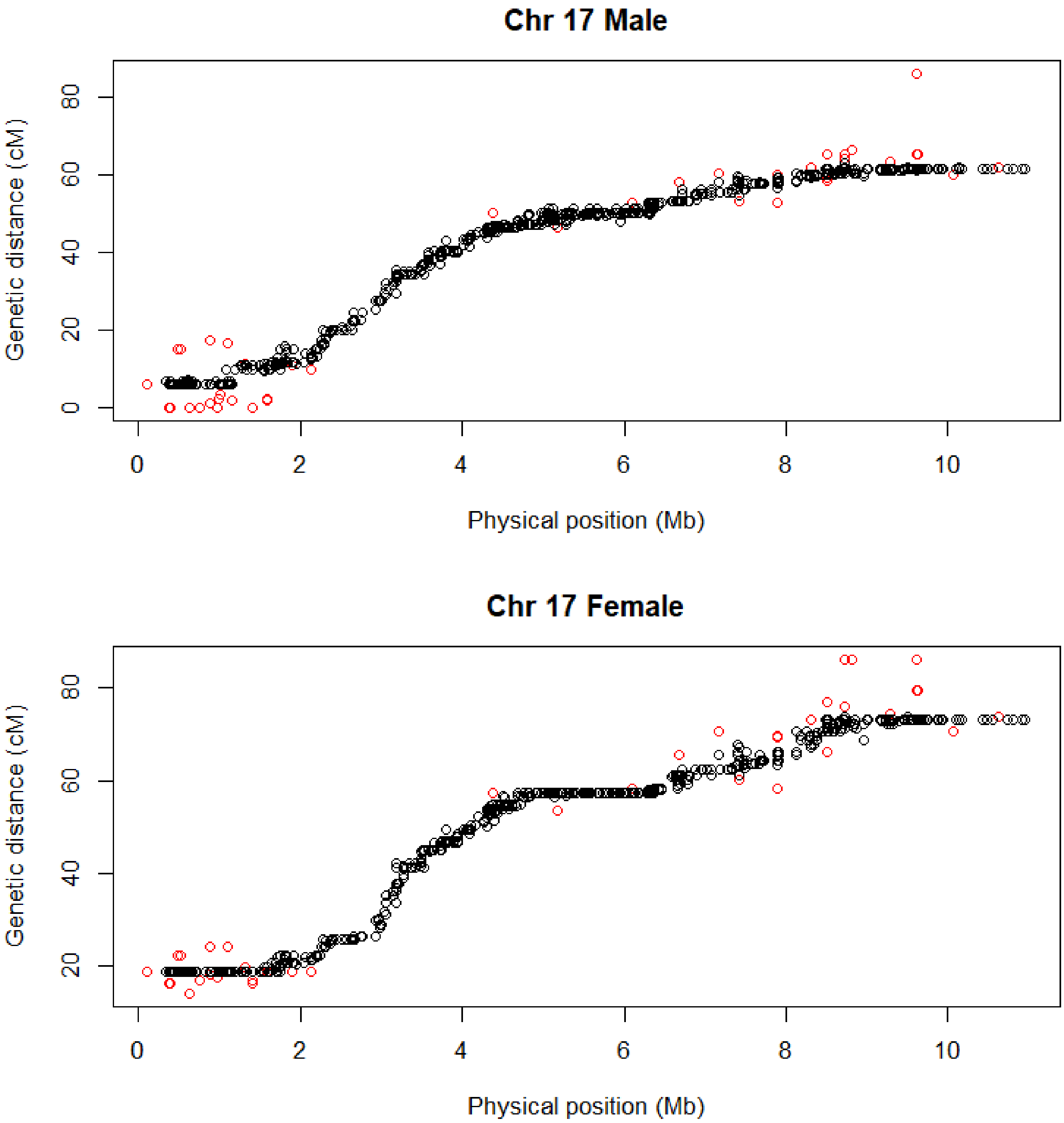
Chr 17 Male
Genetic distance (cM)
0
20
40
60
80
0
2
4
6
8
10
Physical position (Mb)
Chr 17 Female
Genetic distance (cM)
20
40
60
80
0
2
4
6
8
10
Physical position (Mb)

Chr 18 Male

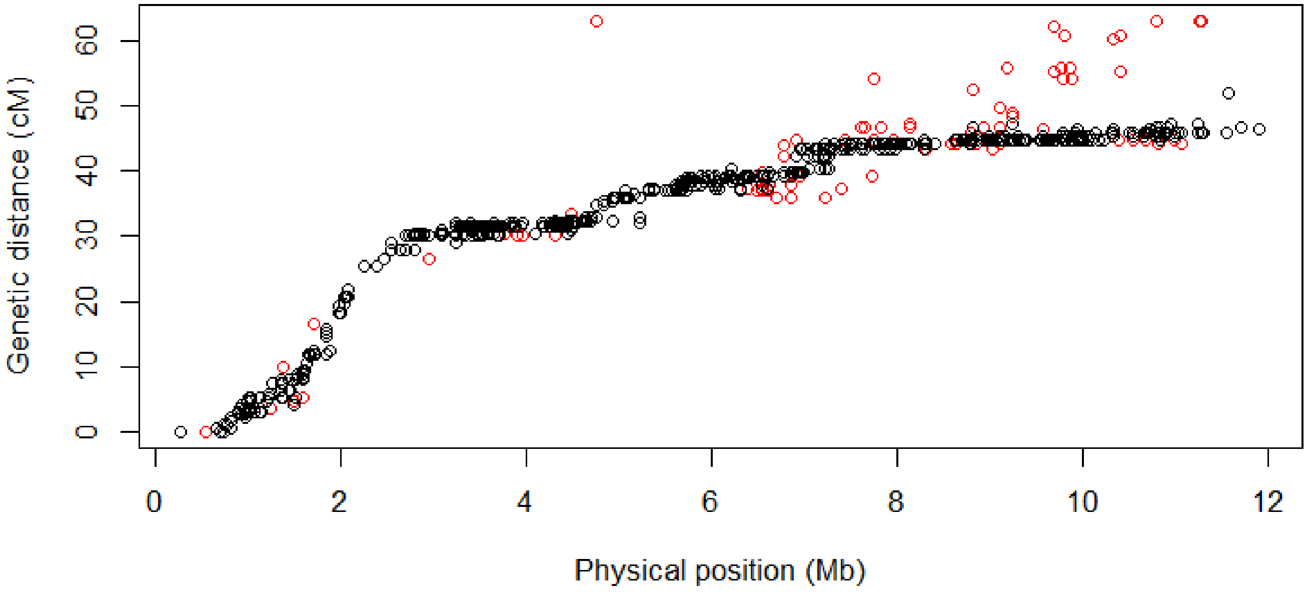

Chr 18 Female

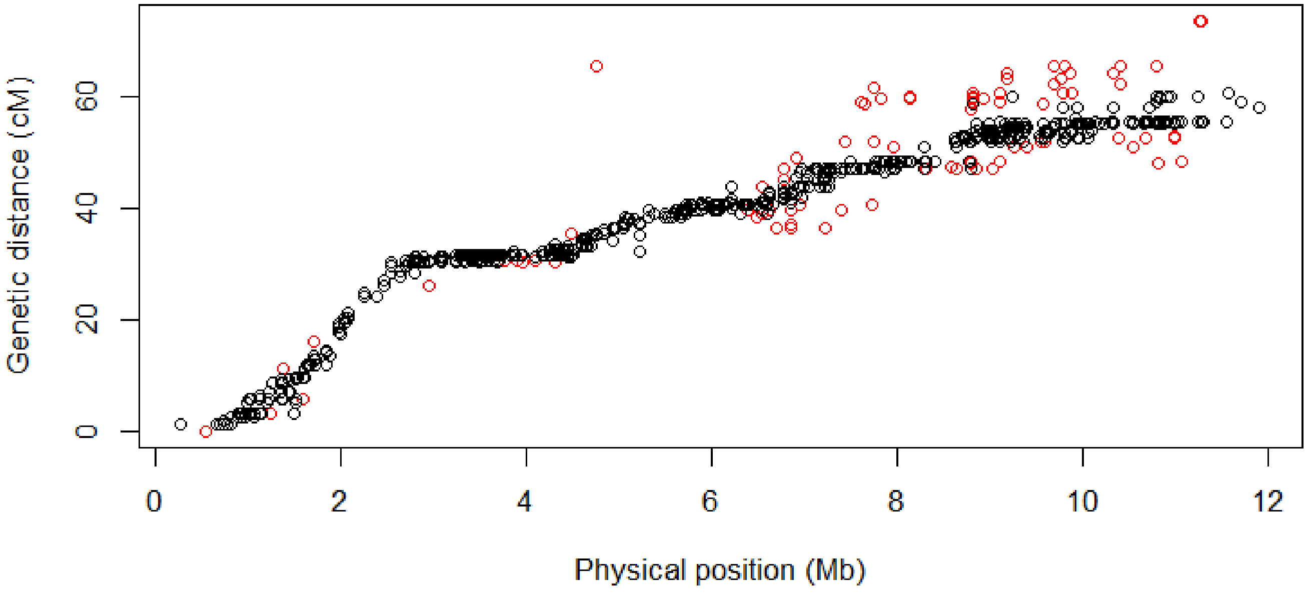

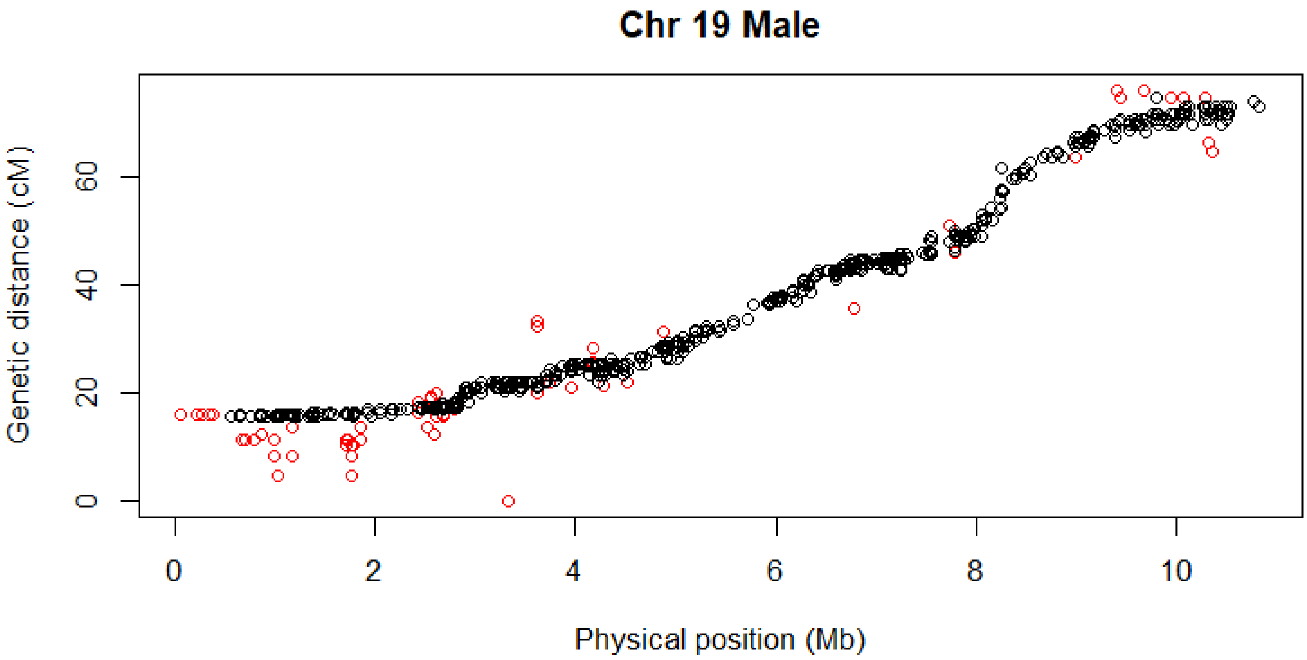

Chr 19 Male
Genetic distance (cM)
0
20
40
60
0
2
4
6
8
10
Physical position (Mb)

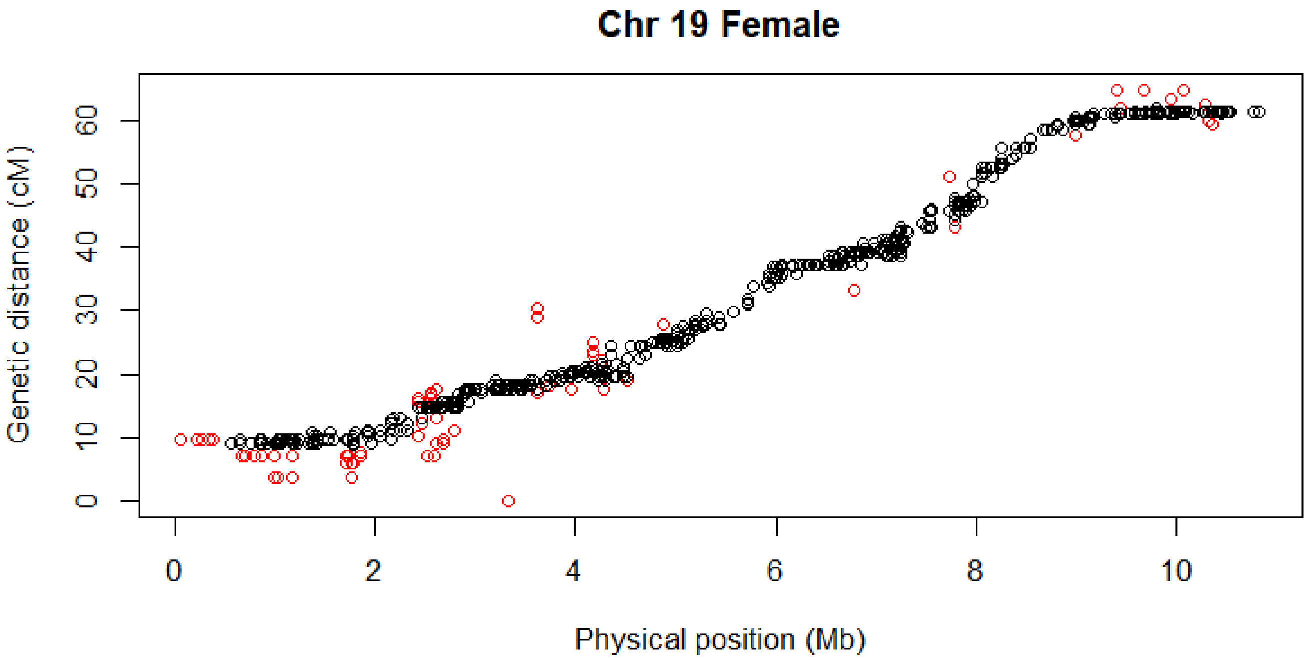

Chr 19 Female
Genetic distance (cM)
0
10
20
30
40
50
60
0
2
4
6
8
10
Physical position (Mb)

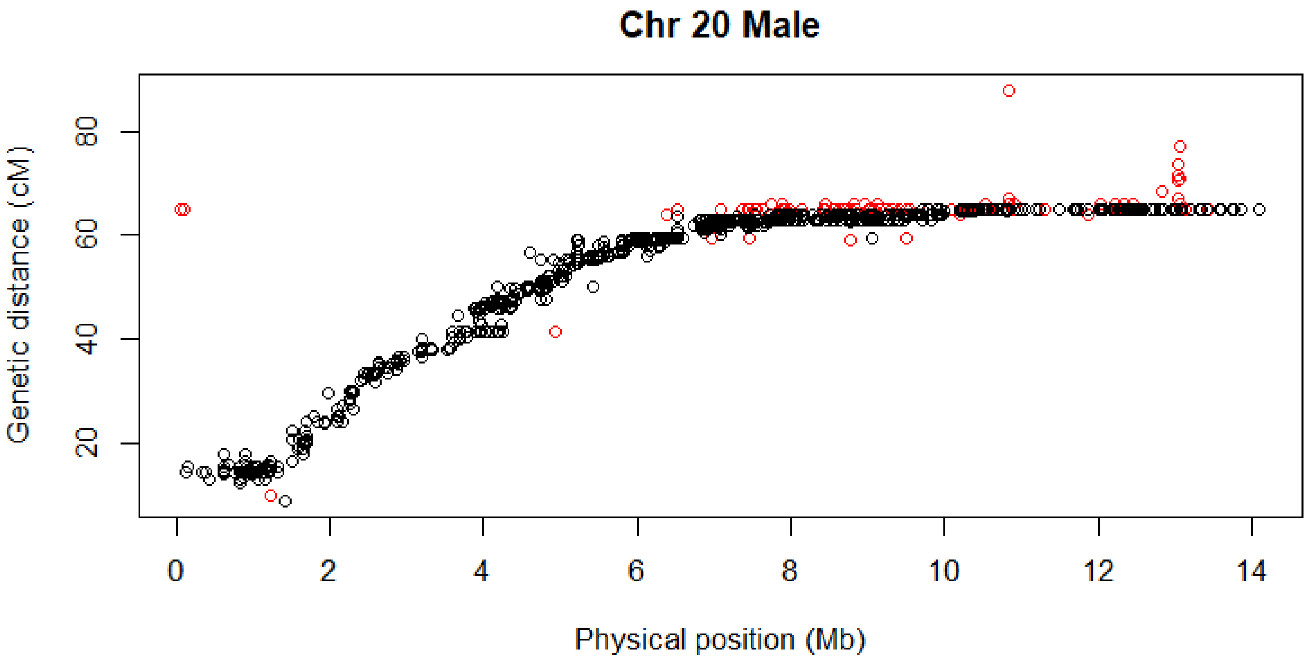

Chr 20 Male
Genetic distance (cM)
20
40
60
80
0
2
4
6
8
10
12
14
Physical position (Mb)

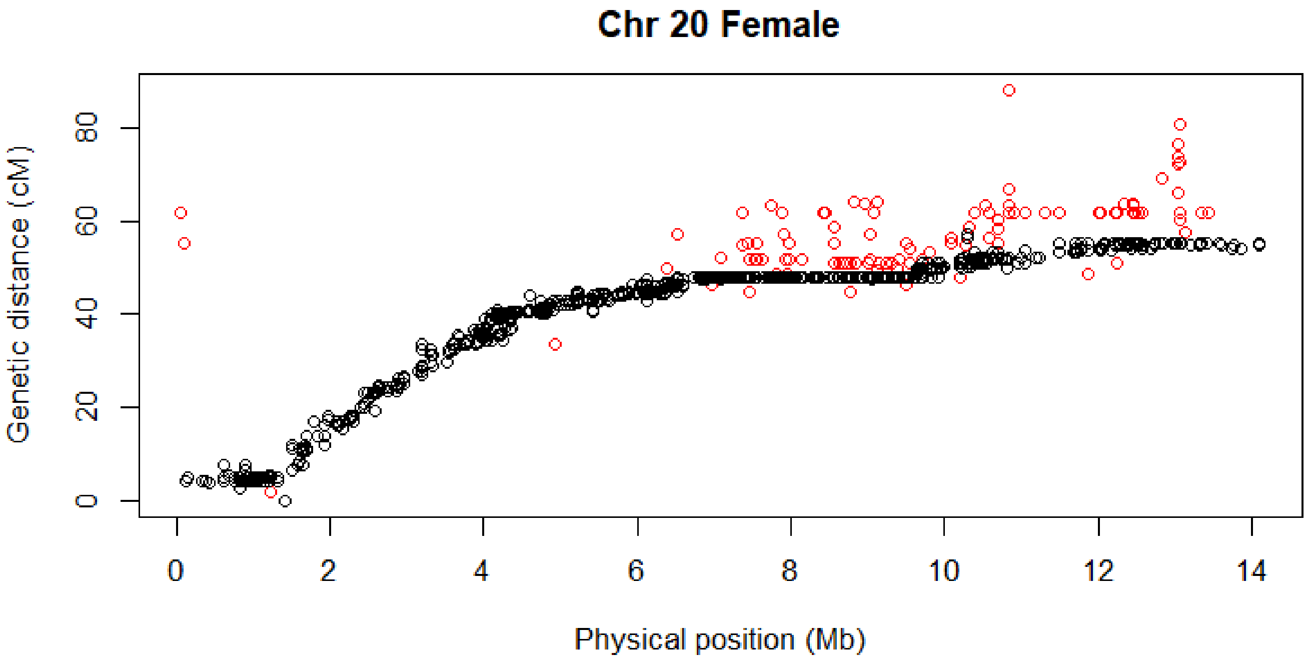

Chr 20 Female
Genetic distance (cM)
0
20
40
60
80
0
2
4
6
8
10
12
14
Physical position (Mb)

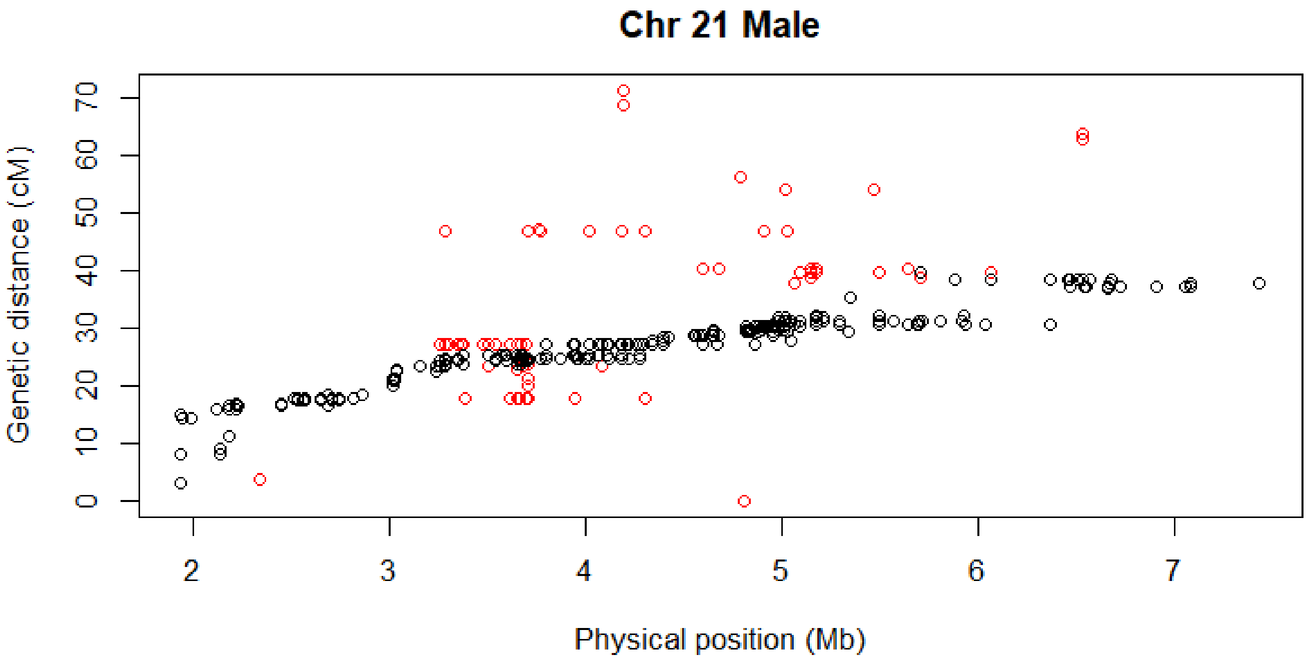
Chr 21 Male
Genetic distance (cM)
0
10
20
30
40
50
60
70
2
3
4
5
6
7
Physical position (Mb)

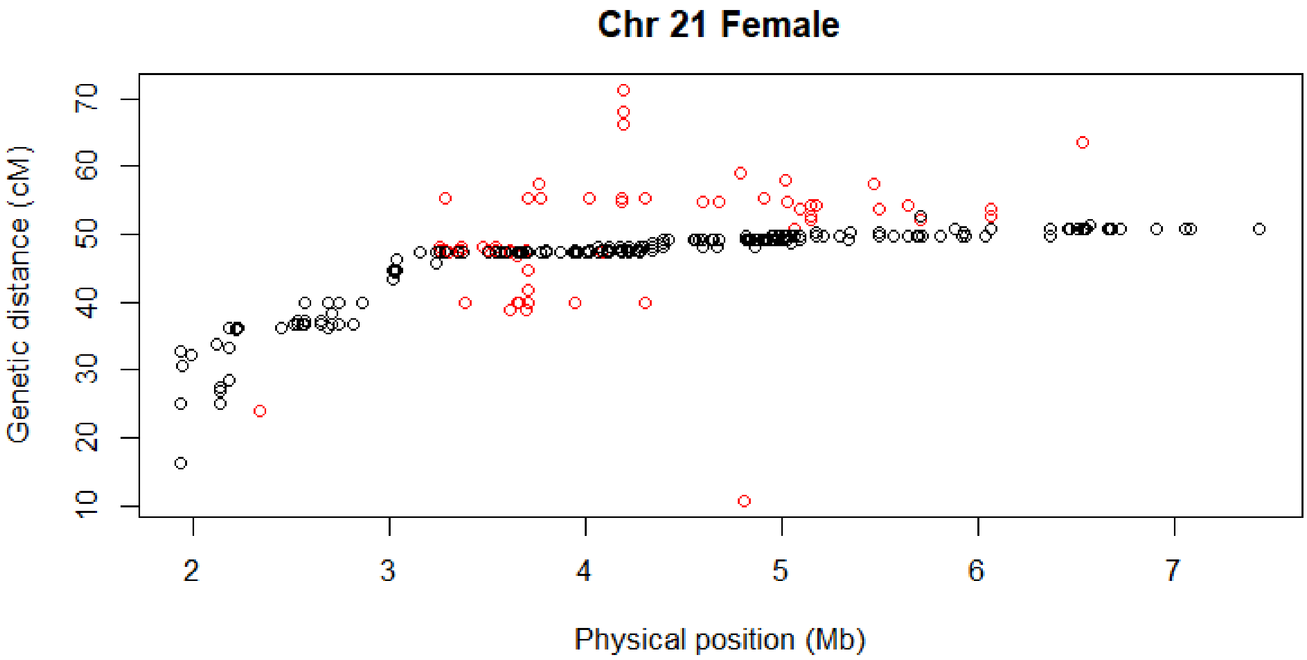
Chr 21 Female
Genetic distance (cM)
10
20
30
40
50
60
70
2
3
4
5
6
7
Physical position (Mb)

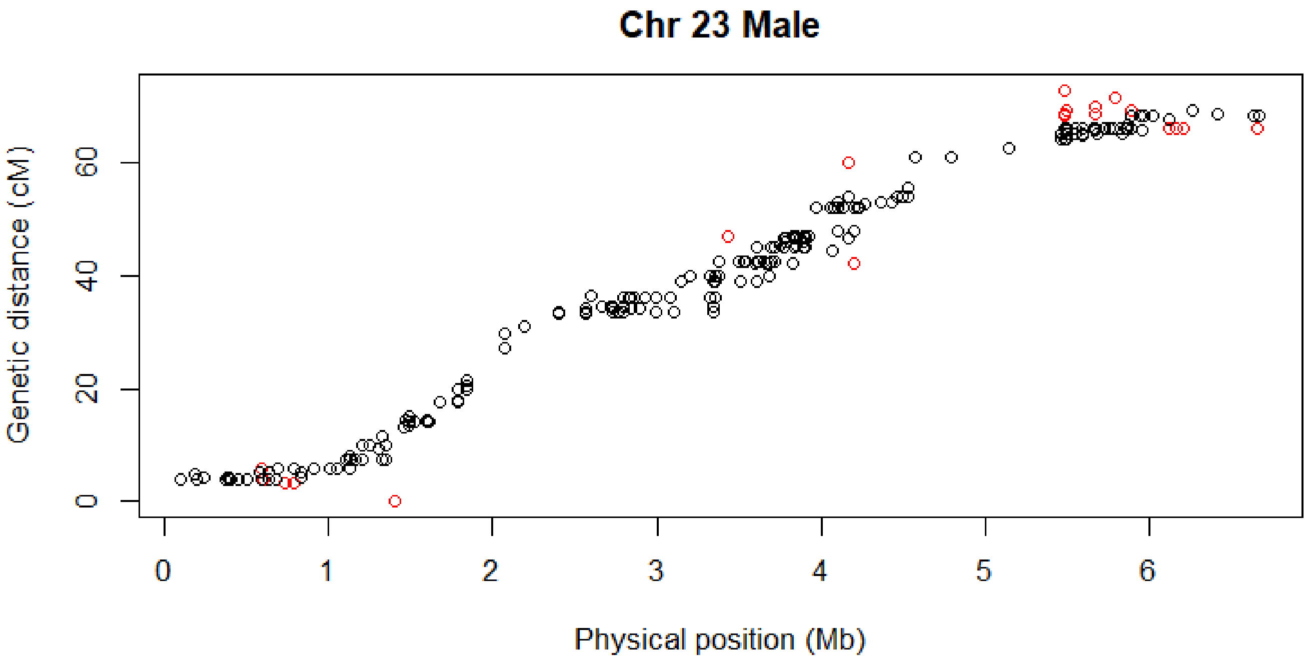
Chr 23 Male
Genetic distance (cM)
0
20
40
60
0
1
2
3
4
5
6
Physical position (Mb)

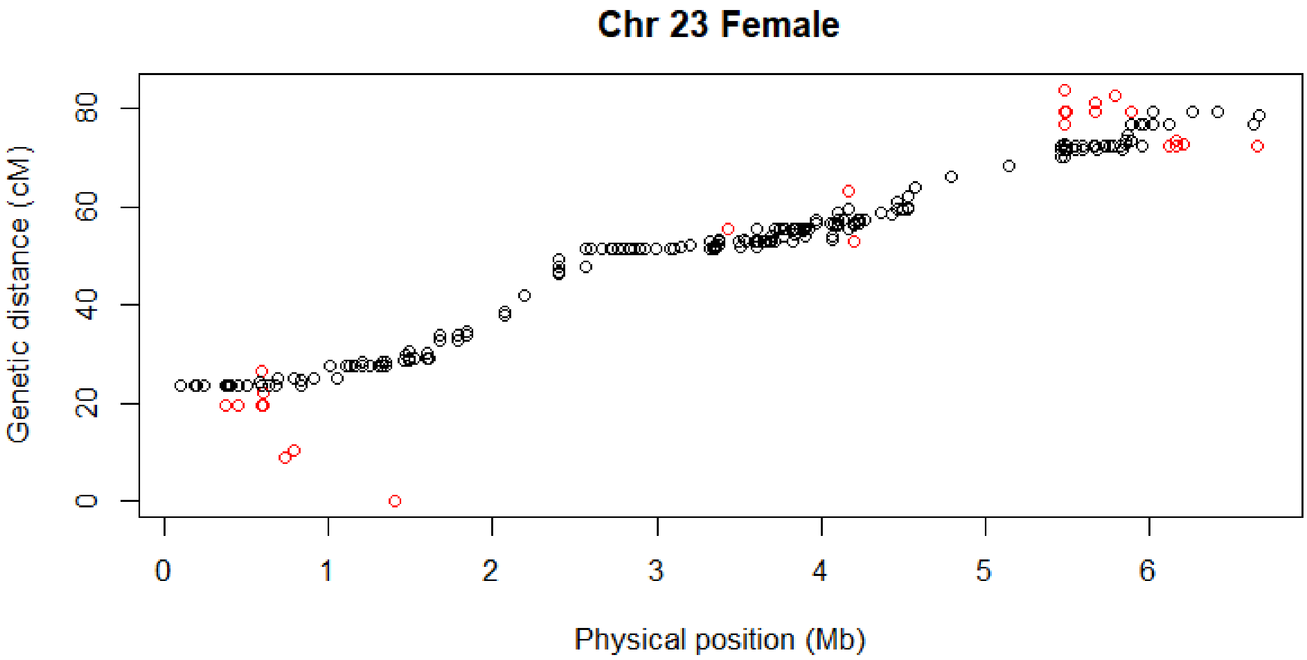
Chr 23 Female
Genetic distance (cM)
0
20
40
60
80
0
1
2
3
4
5
6
Physical position (Mb)

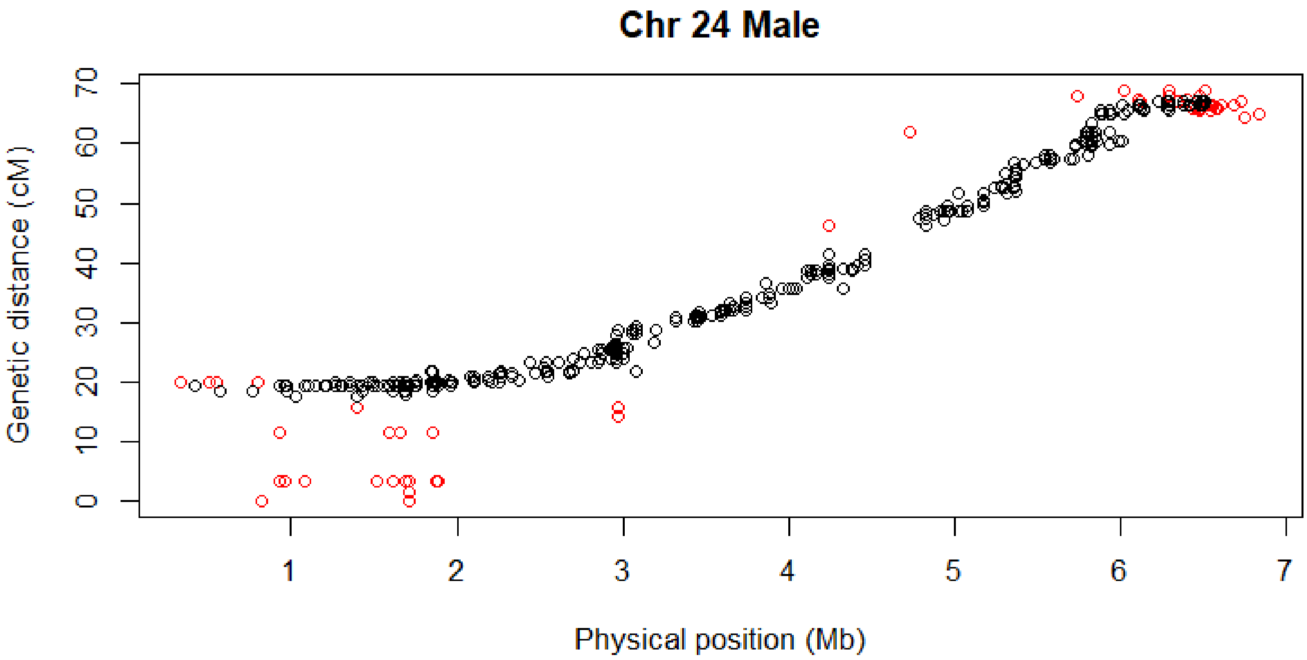

Chr 24 Male
Genetic distance (cM)
0
10
20
30
40
50
60
70
1
2
3
4
5
6
7
Physical position (Mb)

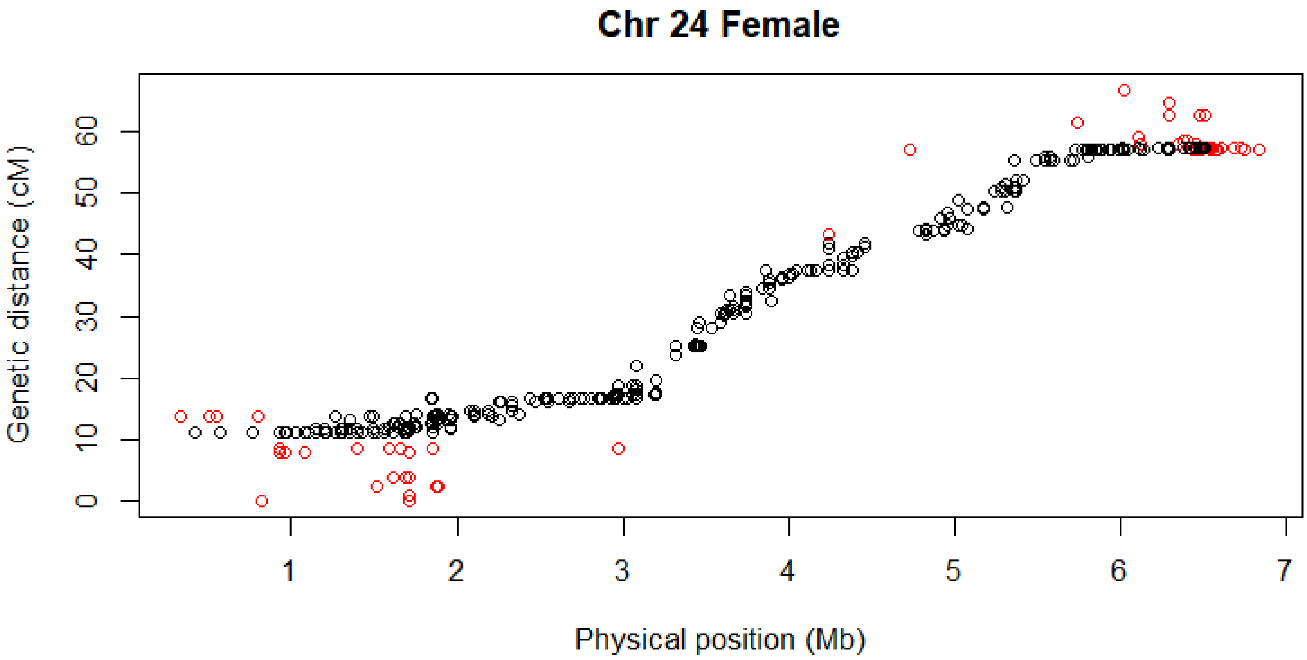

Chr 24 Female
Genetic distance (cM)
0
10
20
30
40
50
60
1
2
3
4
5
6
7
Physical position (Mb)

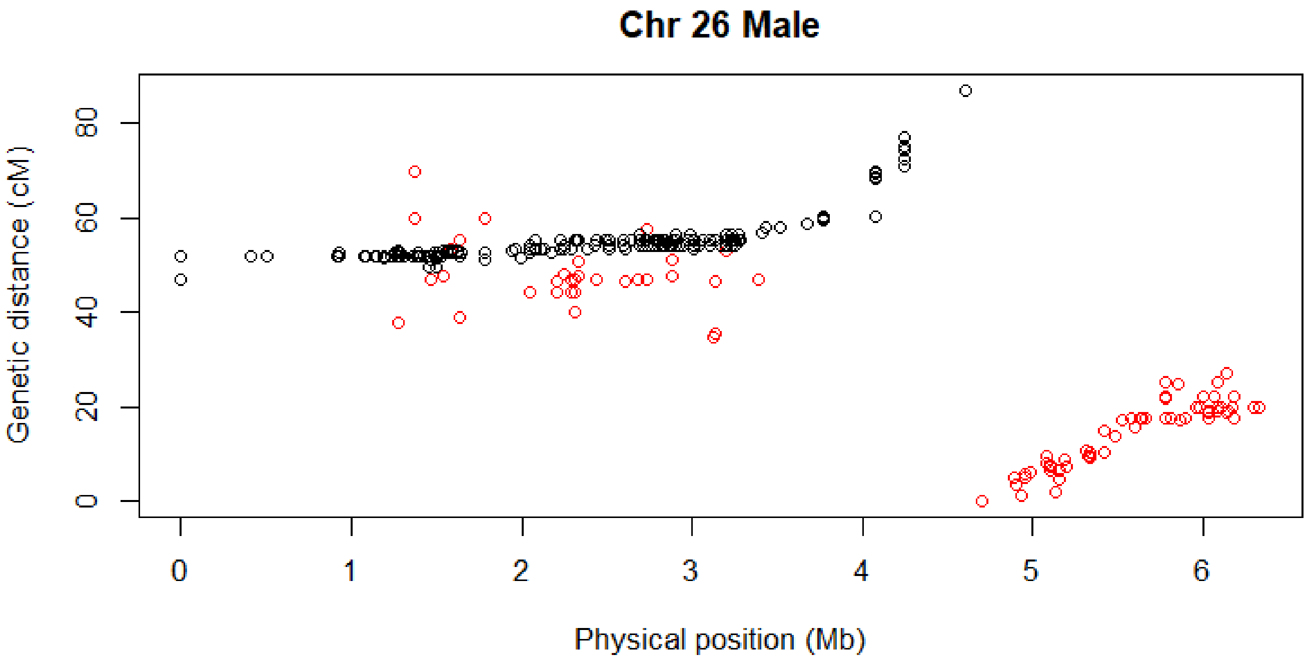
Chr 26 Male
Genetic distance (cM)
0
20
40
60
80
0
1
2
3
4
5
6
Physical position (Mb)

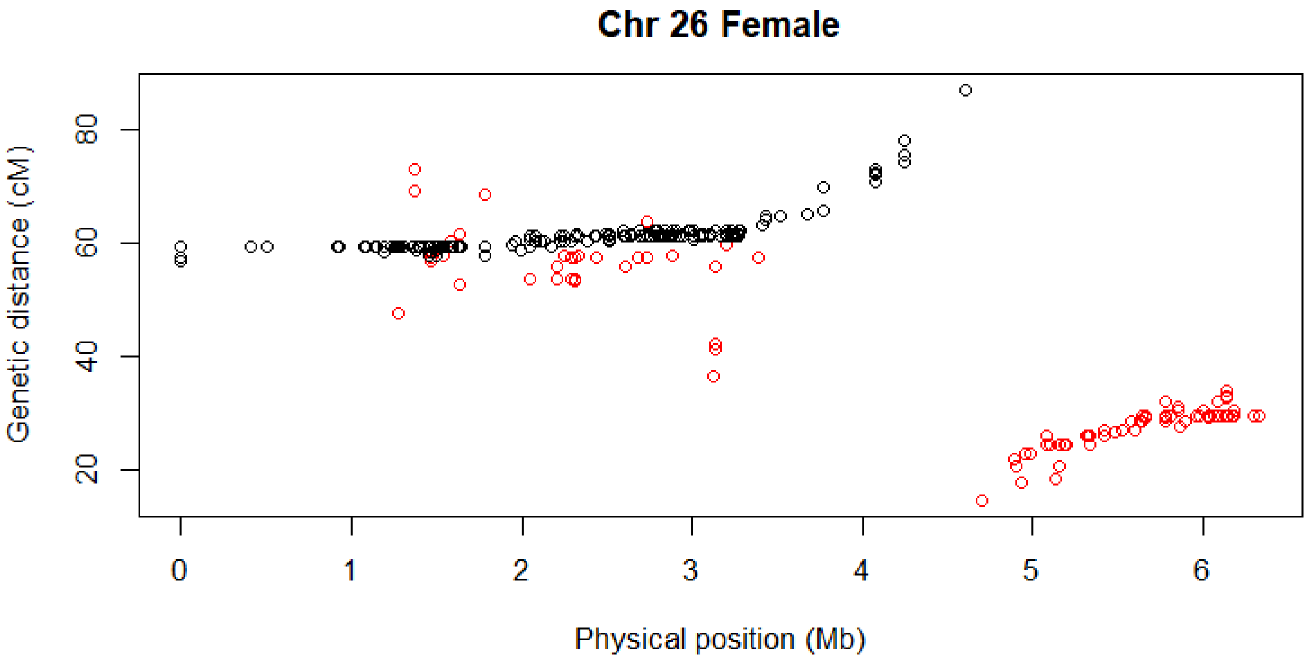
Chr 26 Female
Genetic distance (cM)
20
40
60
80
0
1
2
3
4
5
6
Physical position (Mb)

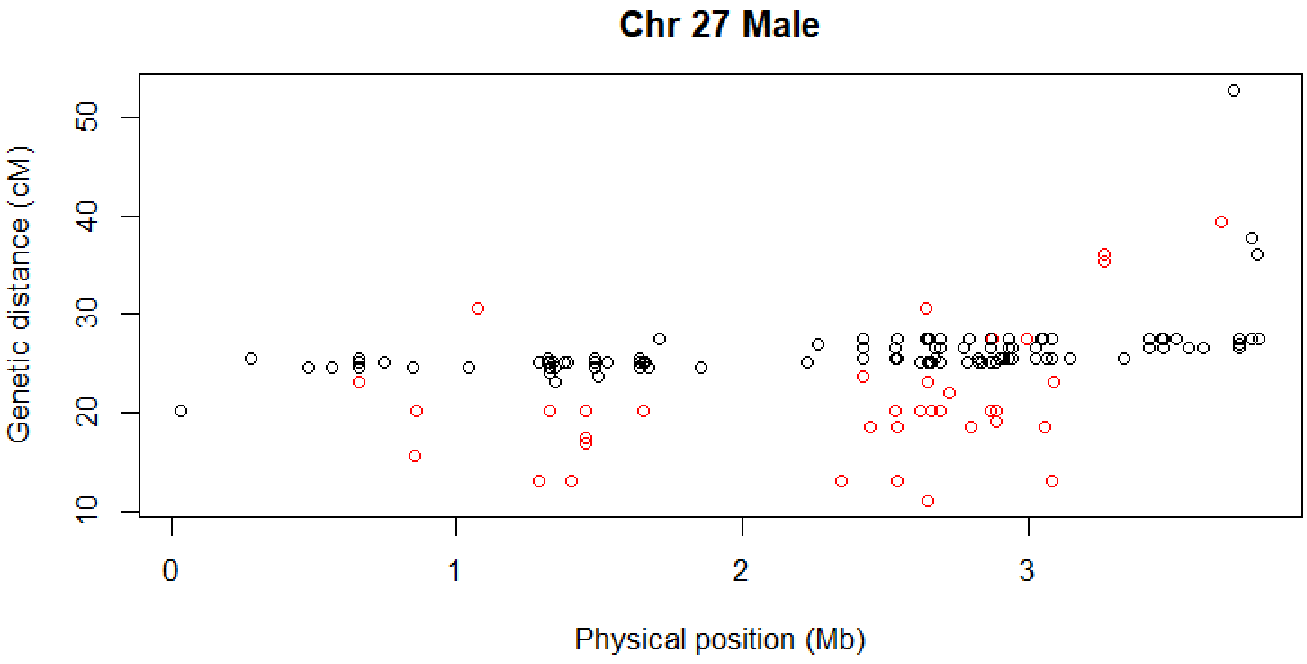
Chr 27 Male
Genetic distance (cM)
10
20
30
40
50
0
1
2
3
Physical position (Mb)

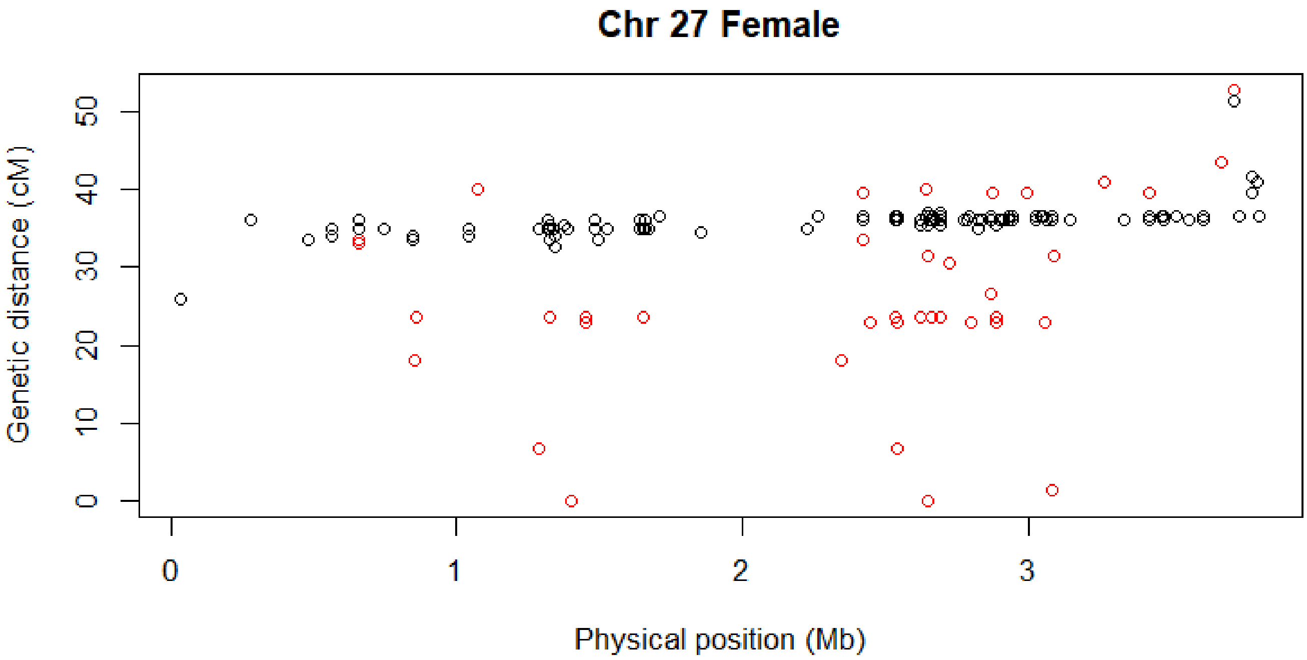
Chr 27 Female
Genetic distance (cM)
0
10
20
30
40
50
0
1
2
3
Physical position (Mb)

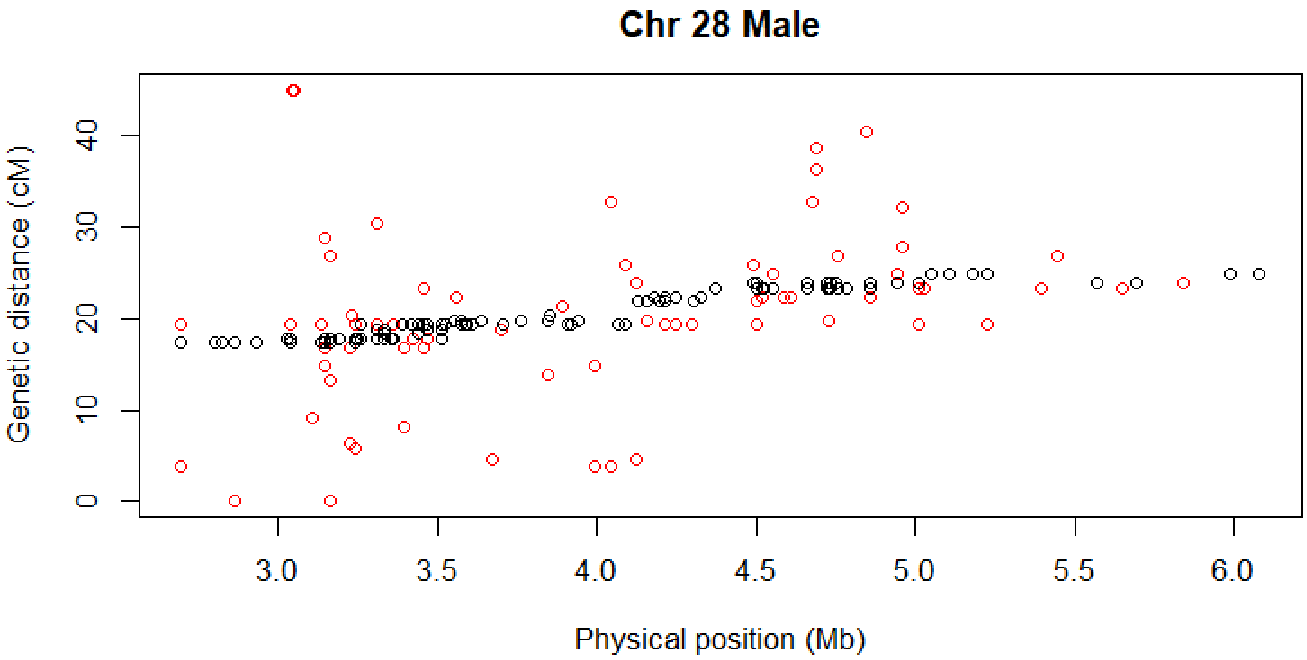
Chr 28 Male
Genetic distance (cM)
0
10
20
30
40
3.0
3.5
4.0
4.5
5.0
5.5
6.0
Physical position (Mb)

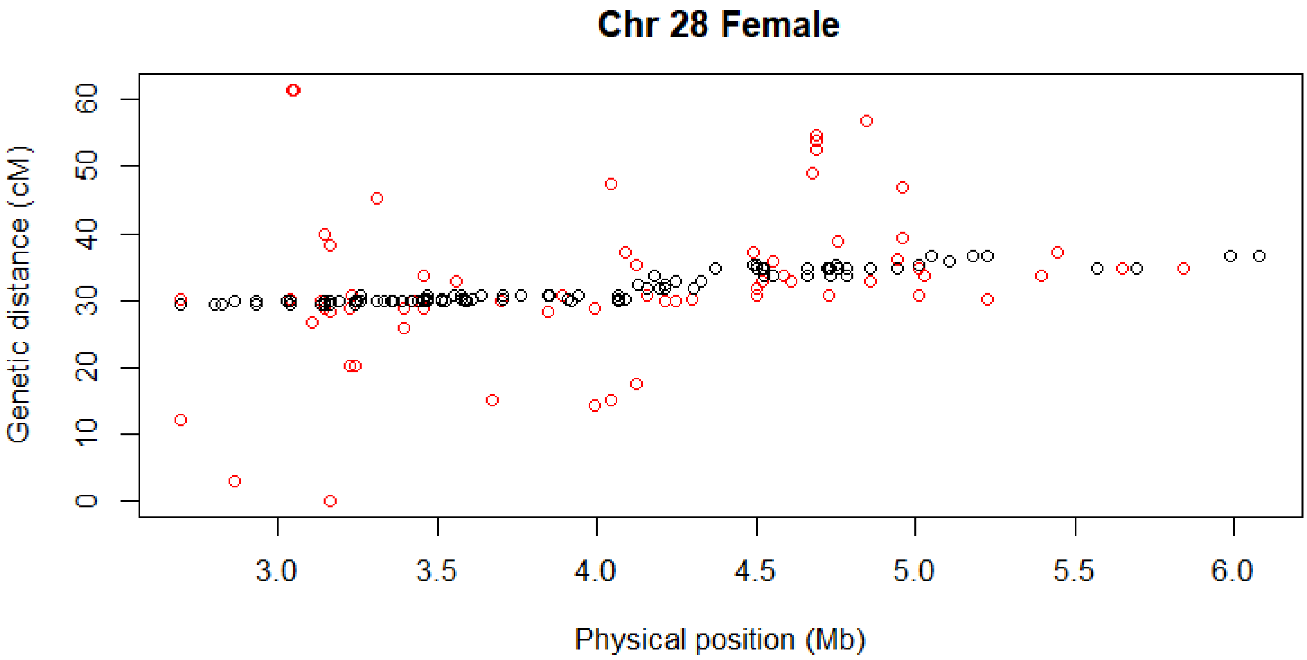
Chr 28 Female
Genetic distance (cM)
0
10
20
30
40
50
60
3.0
3.5
4.0
4.5
5.0
5.5
6.0
Physical position (Mb)
