# Supplementary material for: Chromosome-length genome assembly and linkage map of a critically endangered Australian bird: the helmeted honeyeater
Source: Gigascience. 2022 Mar 29;11:giac025. doi: 10.1093/gigascience/giac025 (PMC8963300; doi:10.1093/gigascience/giac025)
Supplement: giac025_Supplemental_Files [file giac025_supplemental_files.zip › Supplementary_Material_S15.pdf]

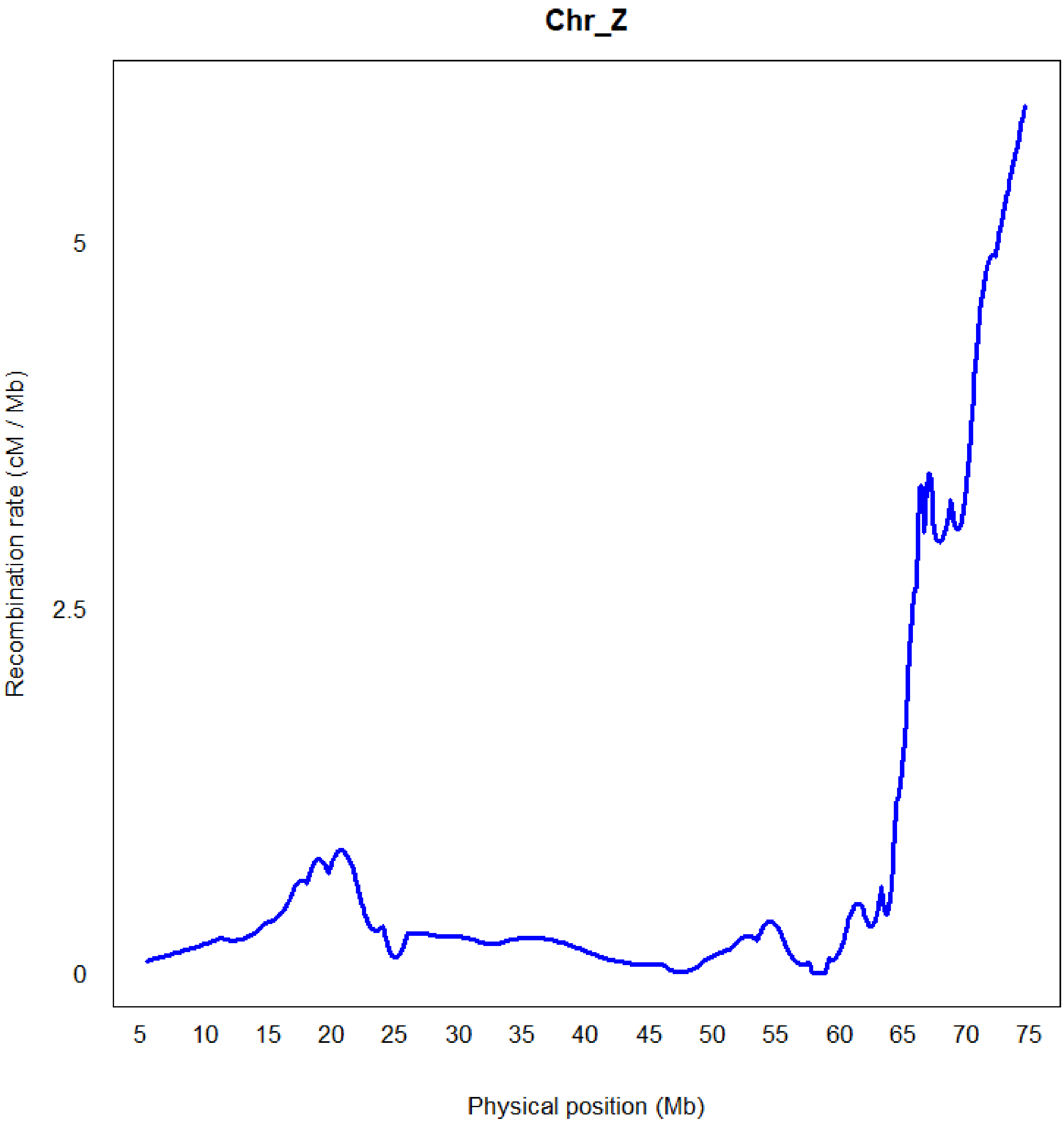

Chr_Z
Recombination rate (cM / Mb)
5
2.5
0
5
10
15
20
25
30
35
40
45
50
55
60
65
70
75
Physical position (Mb)

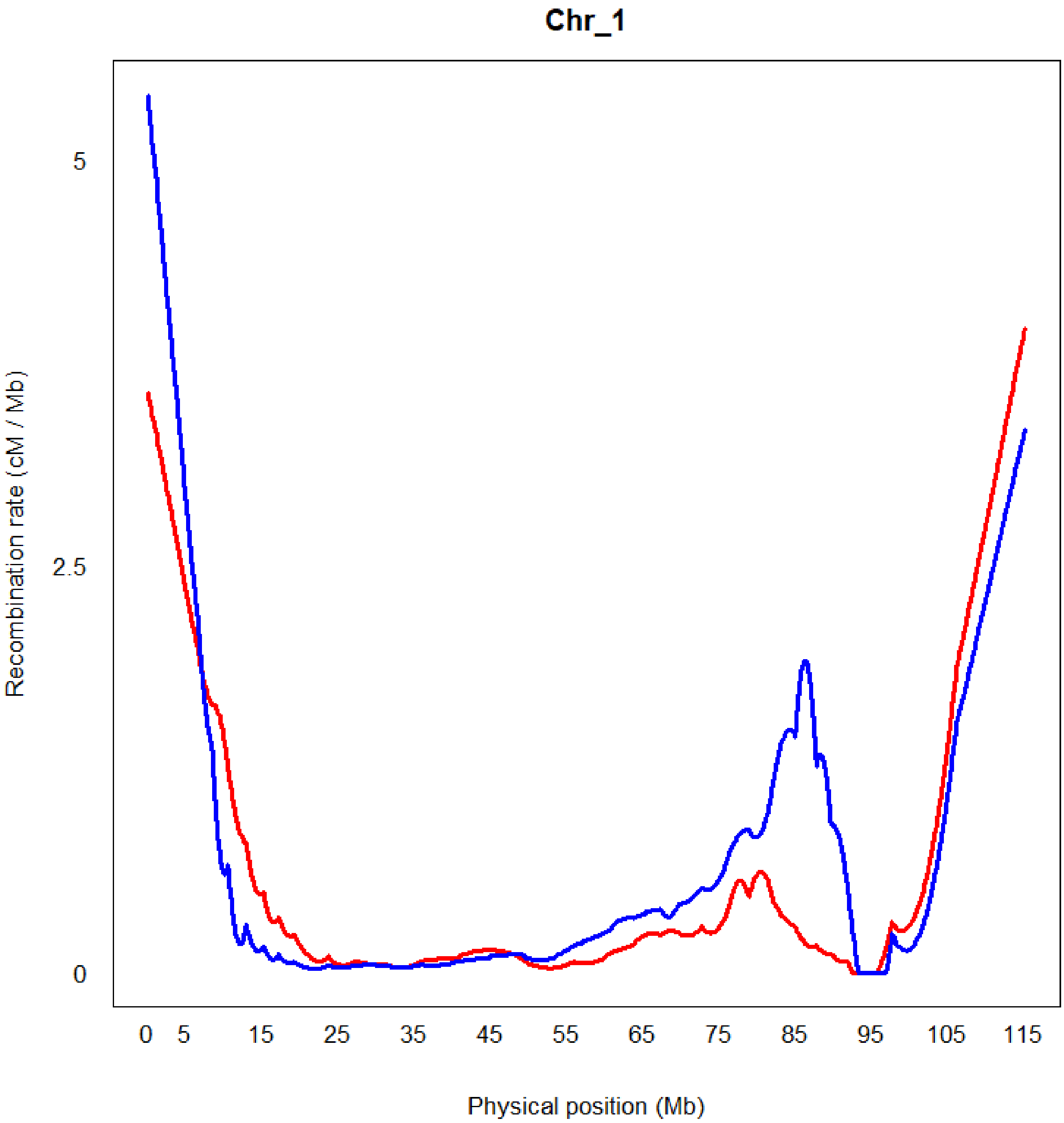

Chr_1
Recombination rate (cM / Mb)
5
2.5
0
0
5
15
25
35
45
55
65
75
85
95
105
115
Physical position (Mb)

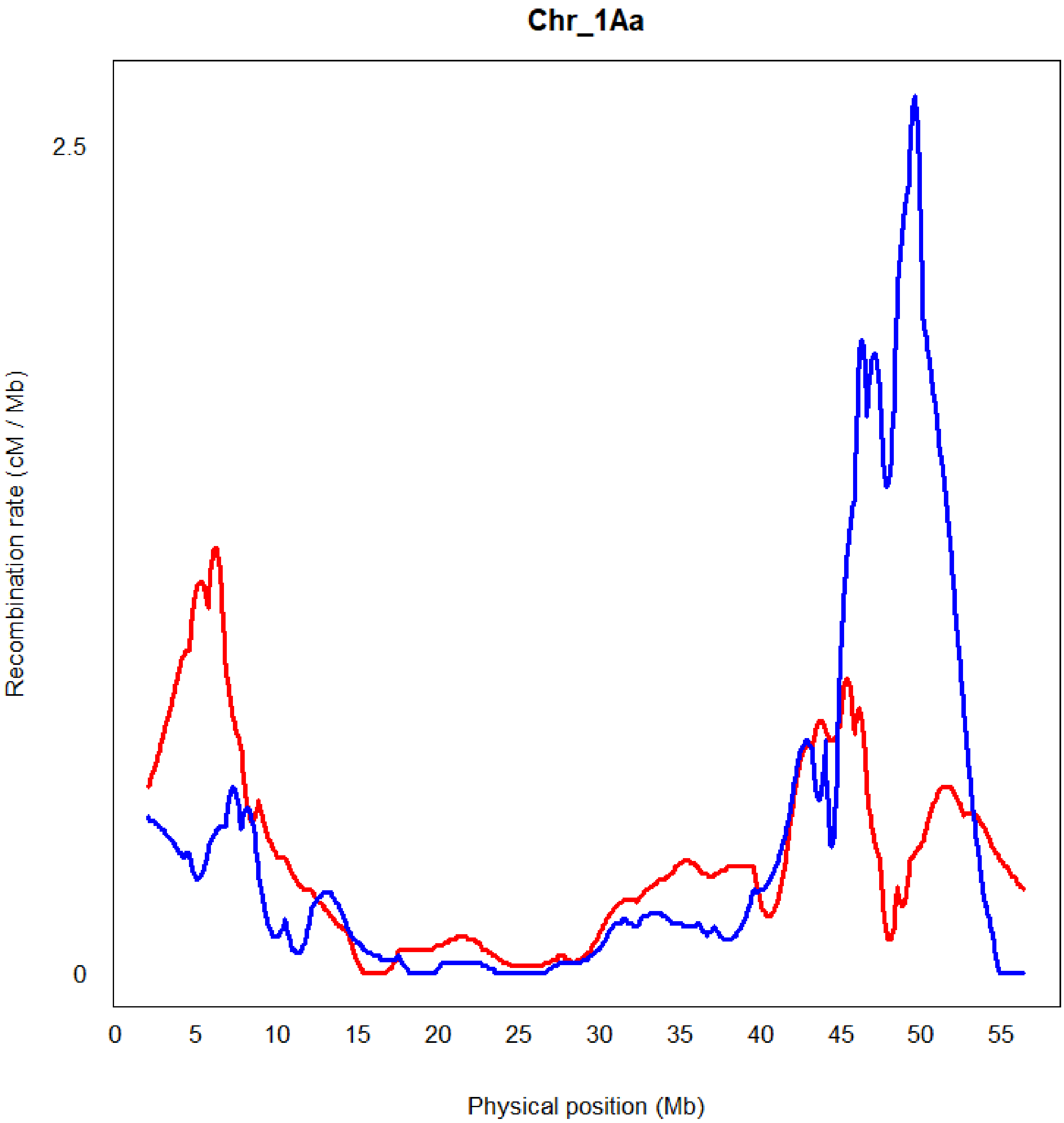

Chr_1Aa
Recombination rate (cM / Mb)
2.5
0
0
5
10
15
20
25
30
35
40
45
50
55
Physical position (Mb)

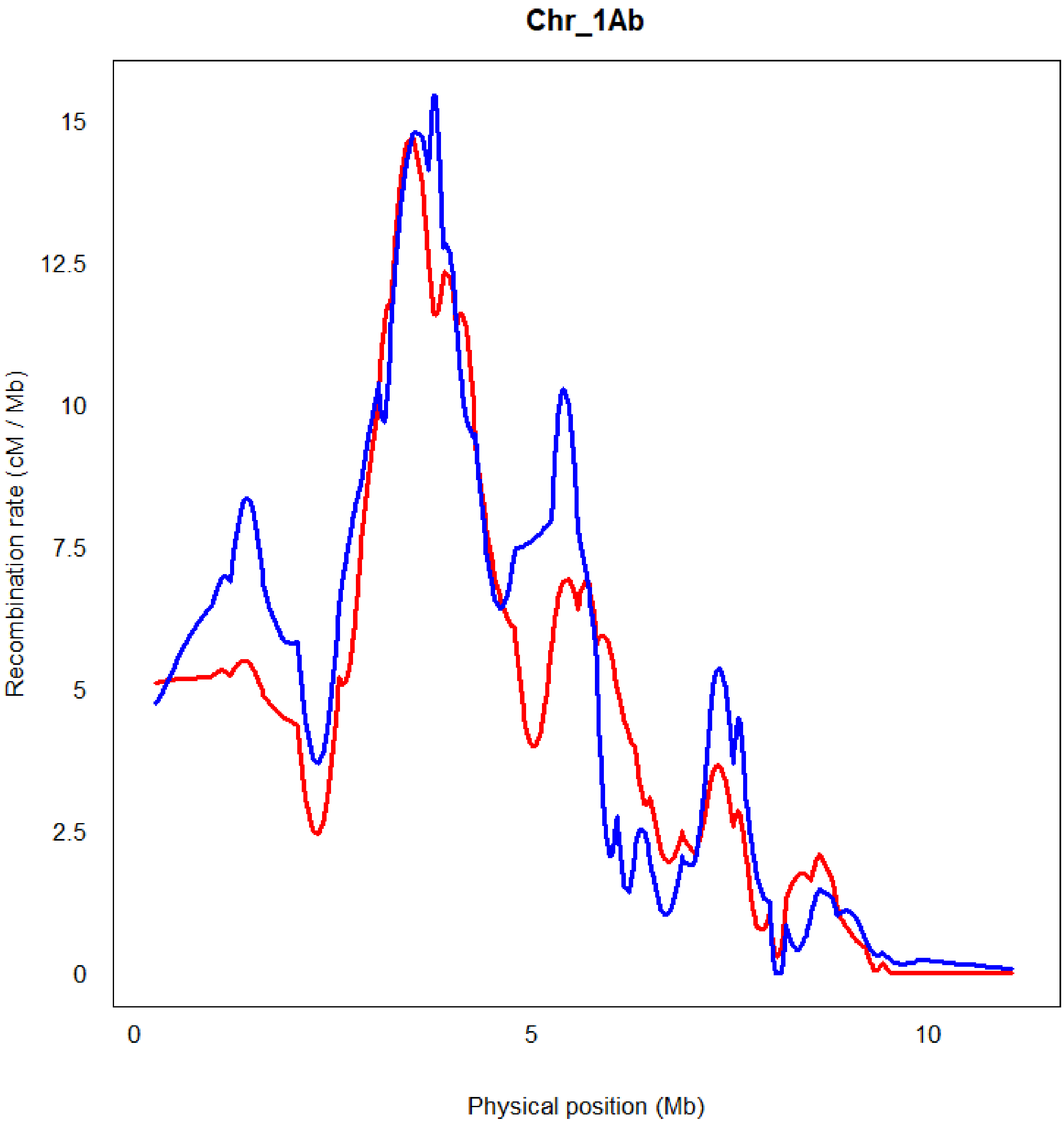
Chr_1Ab
Recombination rate (cM / Mb)
15
12.5
10
7.5
5
2.5
0
0
5
10
Physical position (Mb)

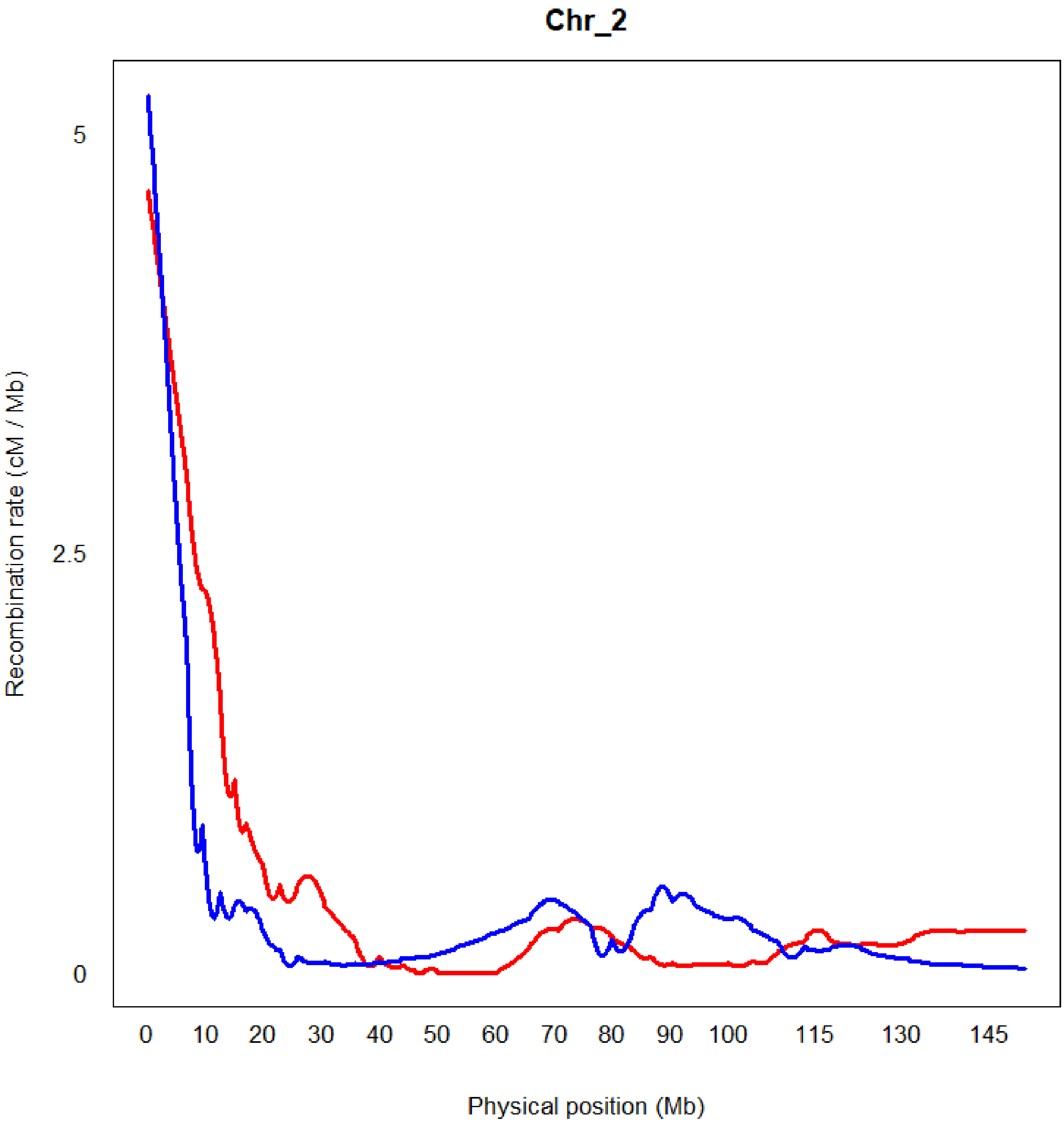
Chr_2
Recombination rate (cM / Mb)
5
2.5
0
0
10
20
30
40
50
60
70
80
90
100
115
130
145
Physical position (Mb)

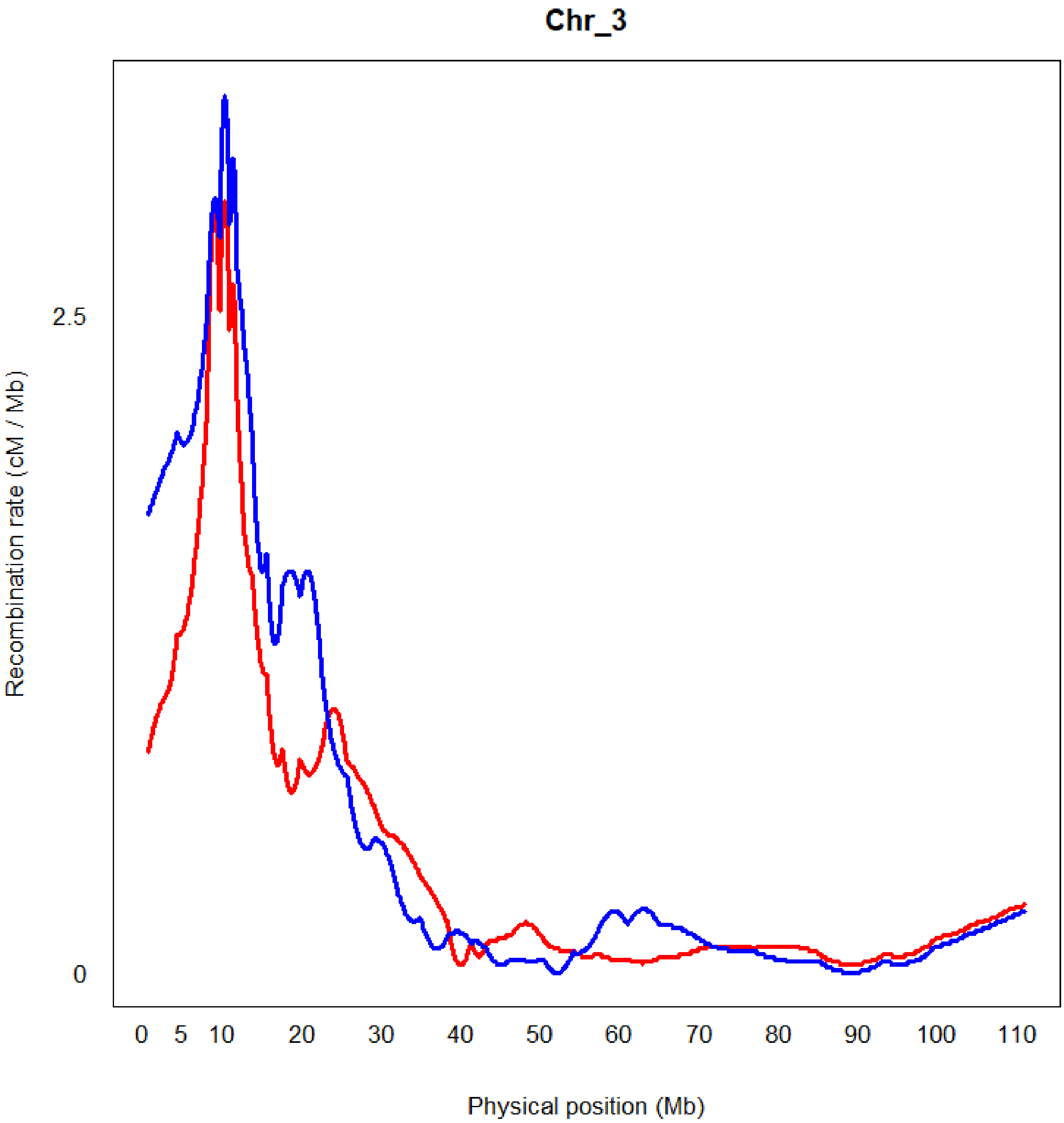

Chr_3
Recombination rate (cM / Mb)
2.5
0
0
5
10
20
30
40
50
60
70
80
90
100
110
Physical position (Mb)

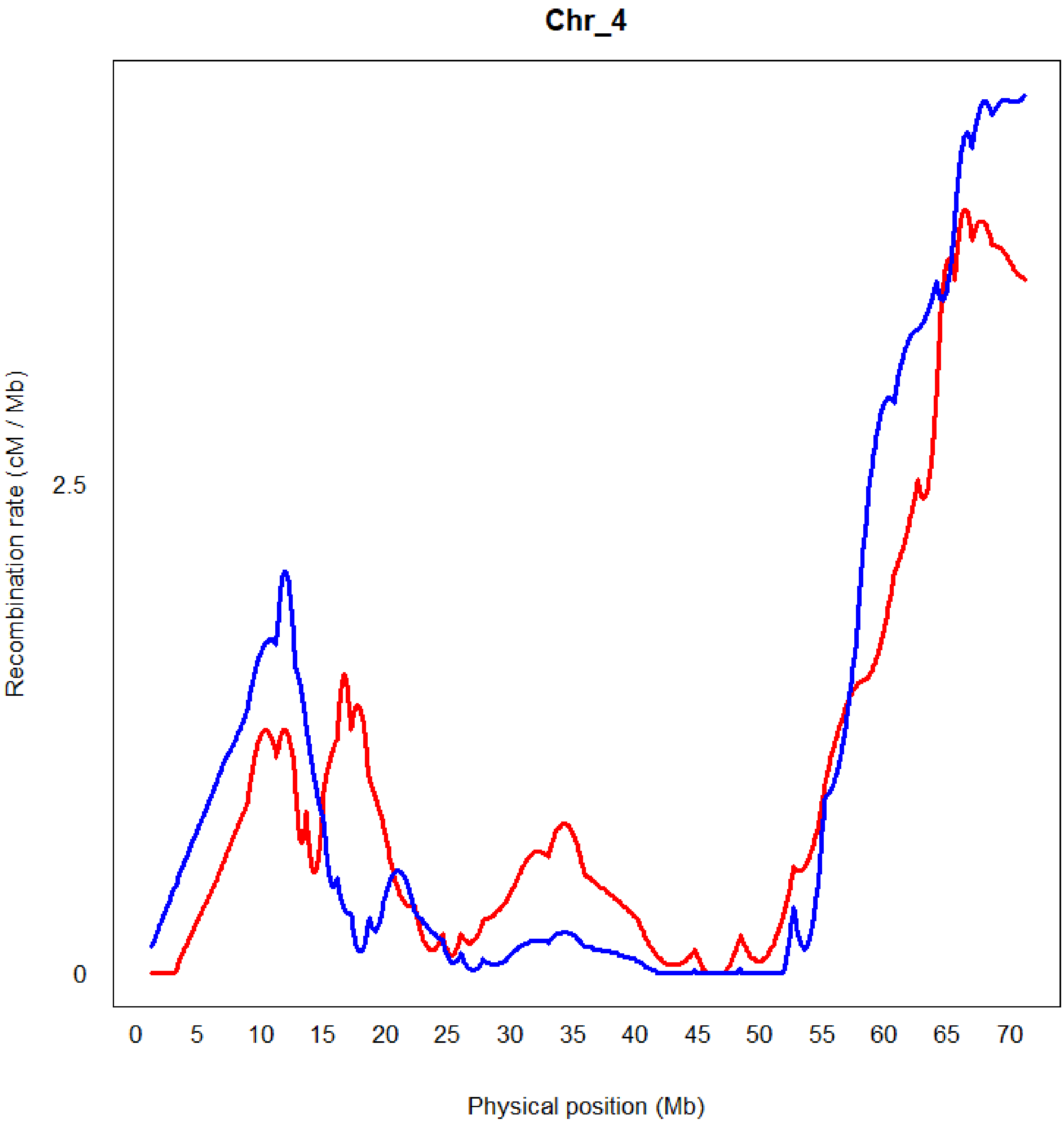

Chr_4
Recombination rate (cM / Mb)
2.5
0
0
5
10
15
20
25
30
35
40
45
50
55
60
65
70
Physical position (Mb)

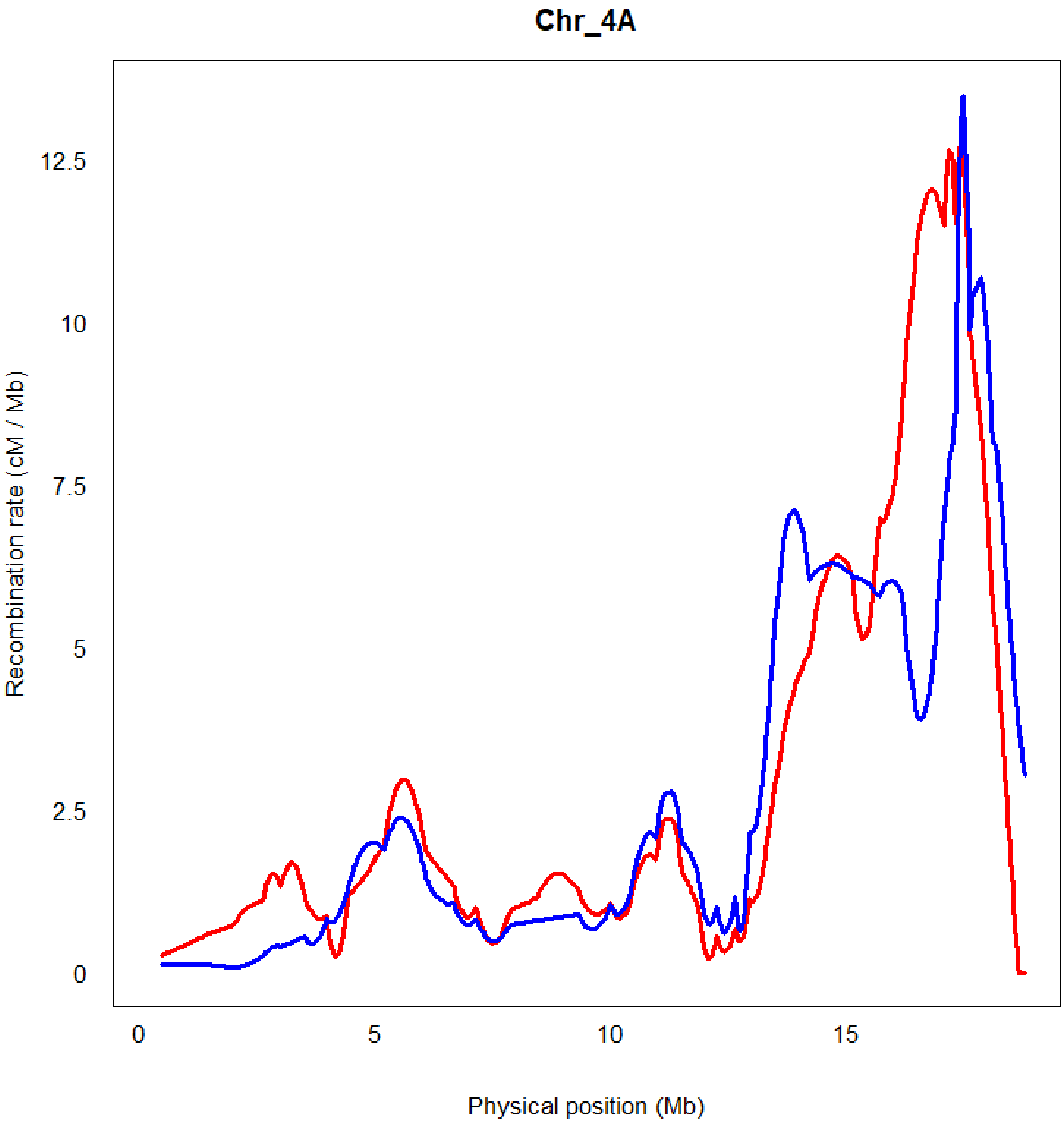
Chr_4A
Recombination rate (cM / Mb)
Physical position (Mb)
0
2.5
5
7.5
10
12.5
0
5
10
15

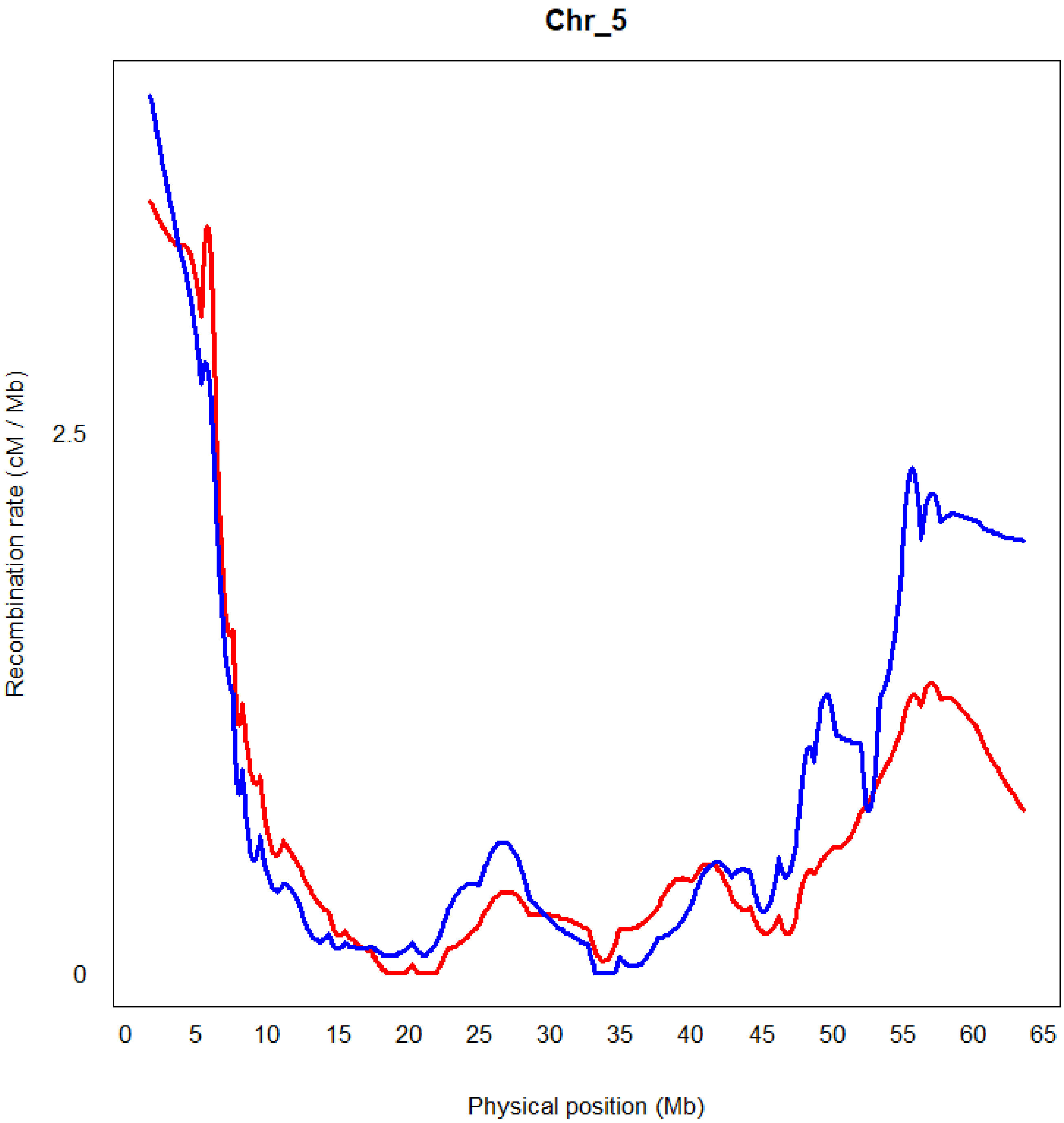
Chr_5
Recombination rate (cM / Mb)
2.5
0
0
5
10
15
20
25
30
35
40
45
50
55
60
65
Physical position (Mb)

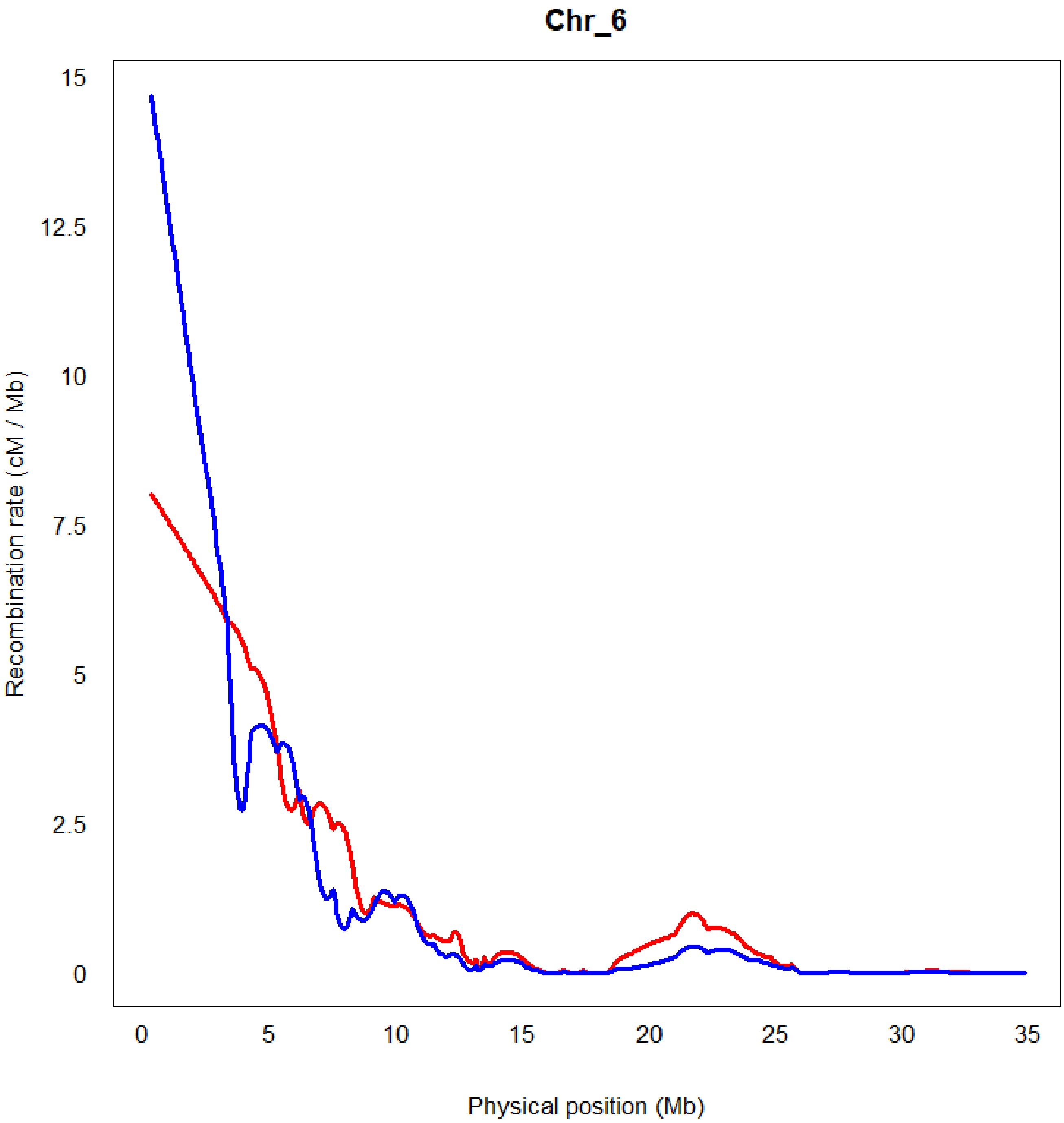
Chr_6
Recombination rate (cM / Mb)
15
12.5
10
7.5
5
2.5
0
0
5
10
15
20
25
30
35
Physical position (Mb)

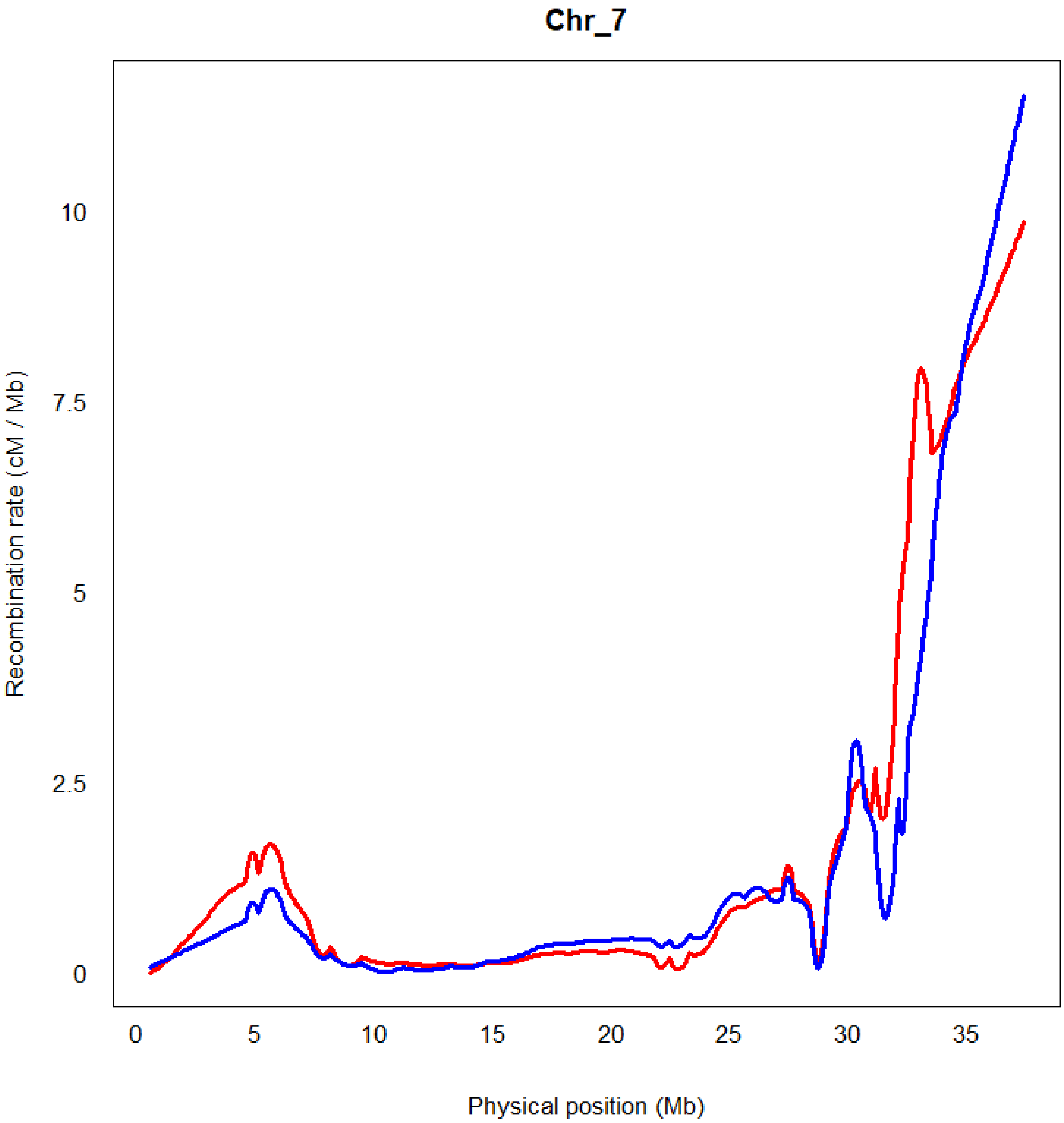

Chr_7
Recombination rate (cM / Mb)
0
2.5
5
7.5
10
0
5
10
15
20
25
30
35
Physical position (Mb)

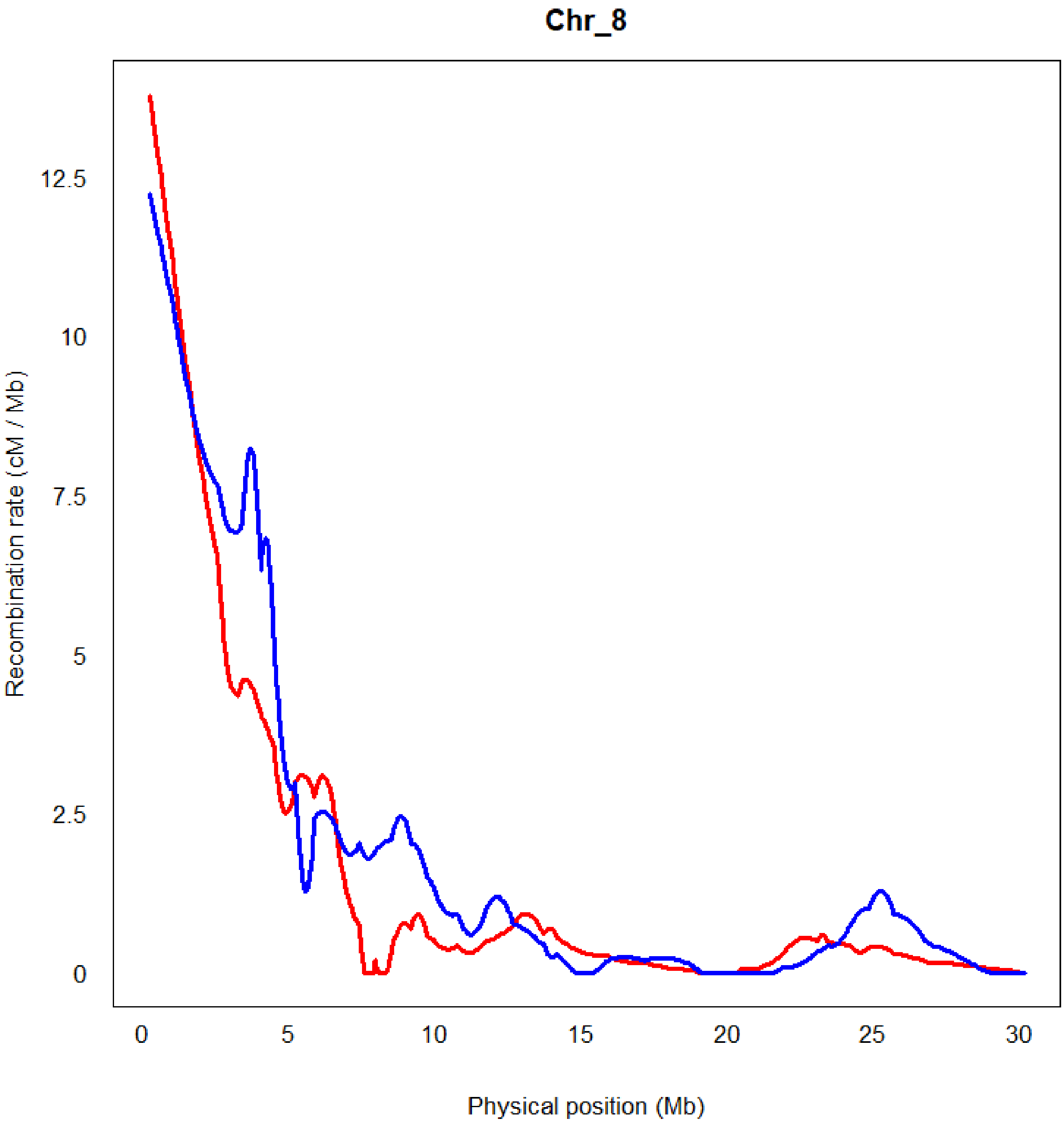
Chr_8
Recombination rate (cM / Mb)
12.5
10
7.5
5
2.5
0
0
5
10
15
20
25
30
Physical position (Mb)

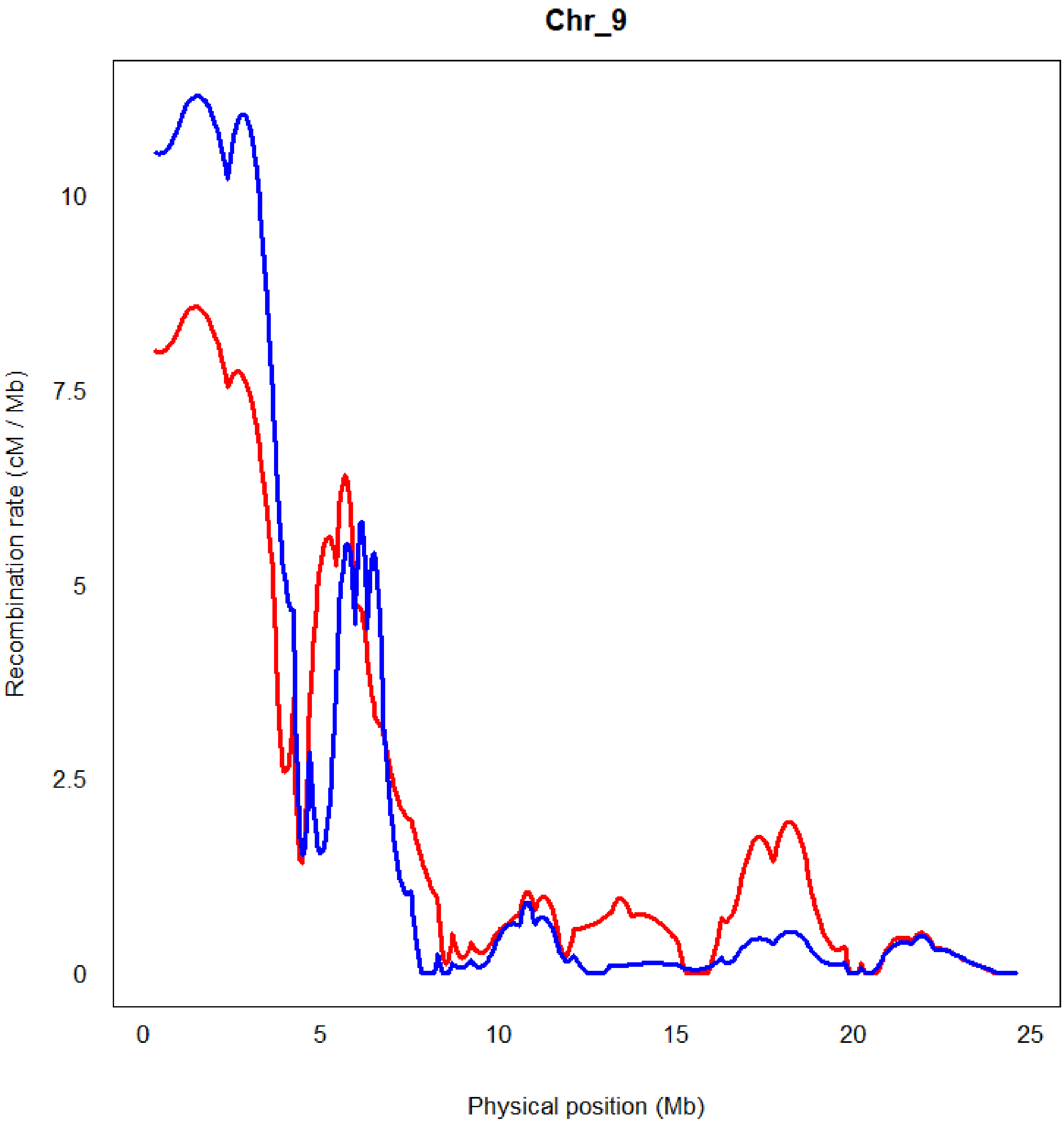
Chr_9
Recombination rate (cM / Mb)
10
7.5
5
2.5
0
0
5
10
15
20
25
Physical position (Mb)

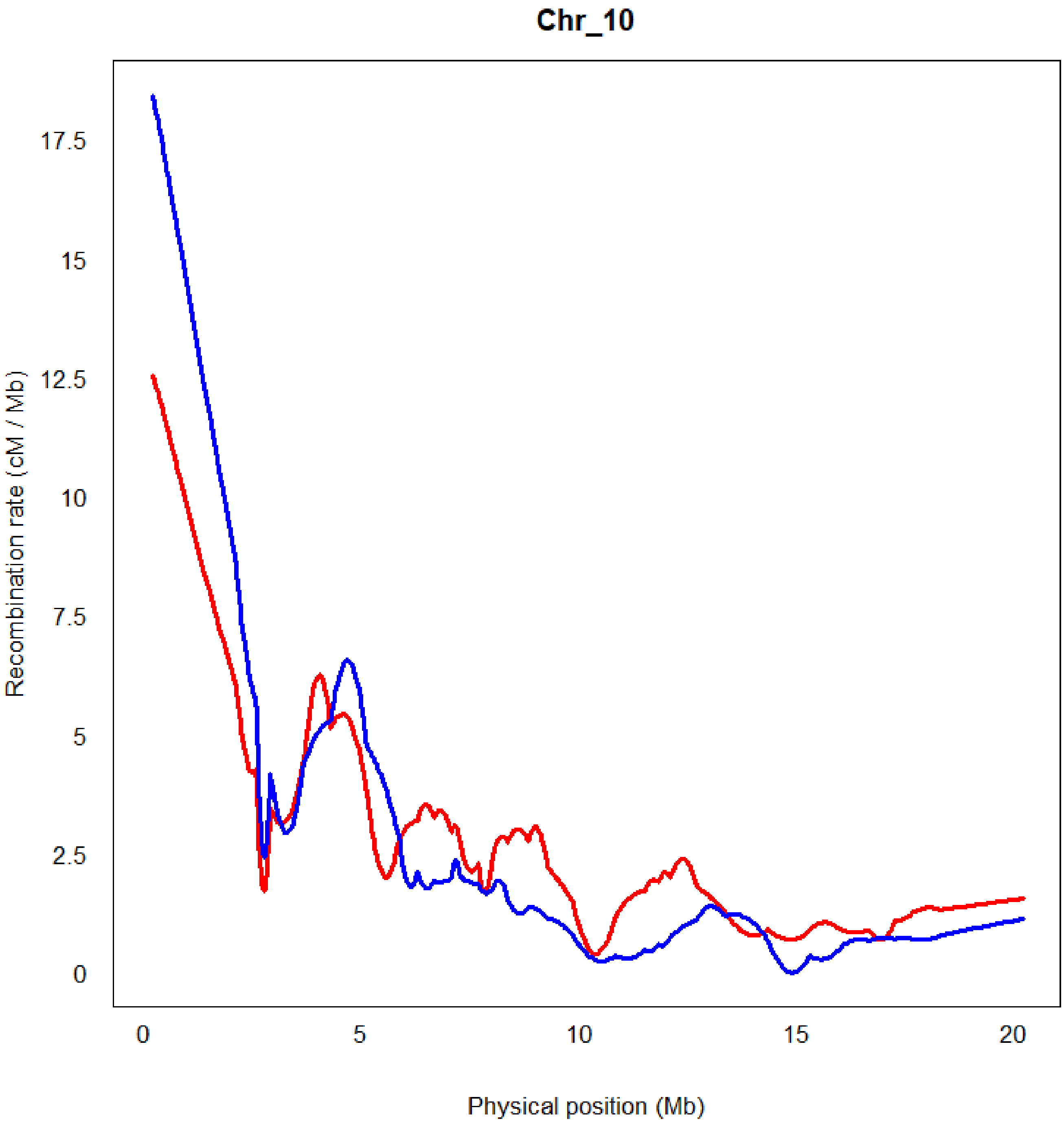
Chr_10
Recombination rate (cM / Mb)
17.5
15
12.5
10
7.5
5
2.5
0
0
5
10
15
20
Physical position (Mb)

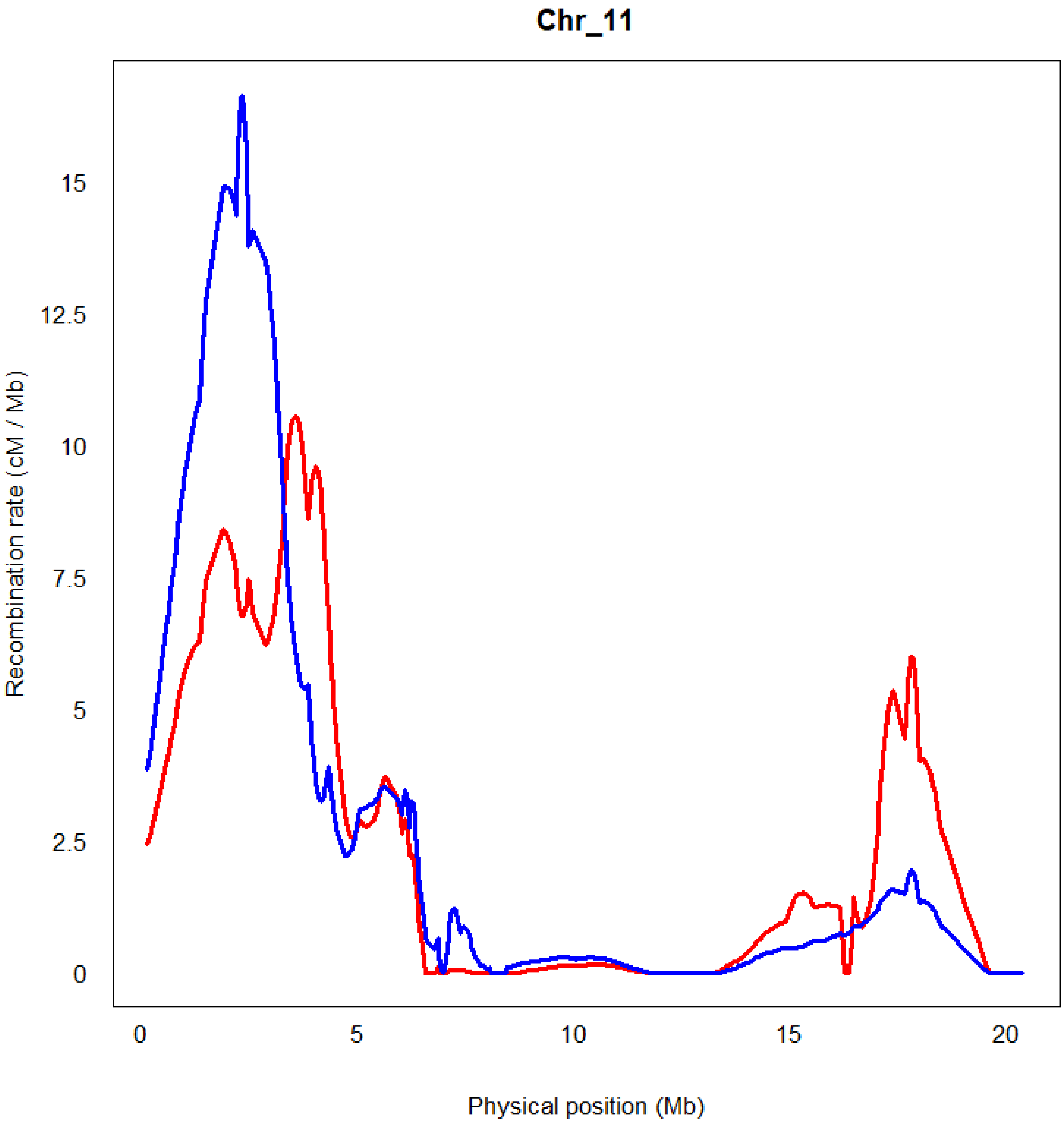

Chr_11
Recombination rate (cM / Mb)
0
2.5
5
7.5
10
12.5
15
0
5
10
15
20
Physical position (Mb)

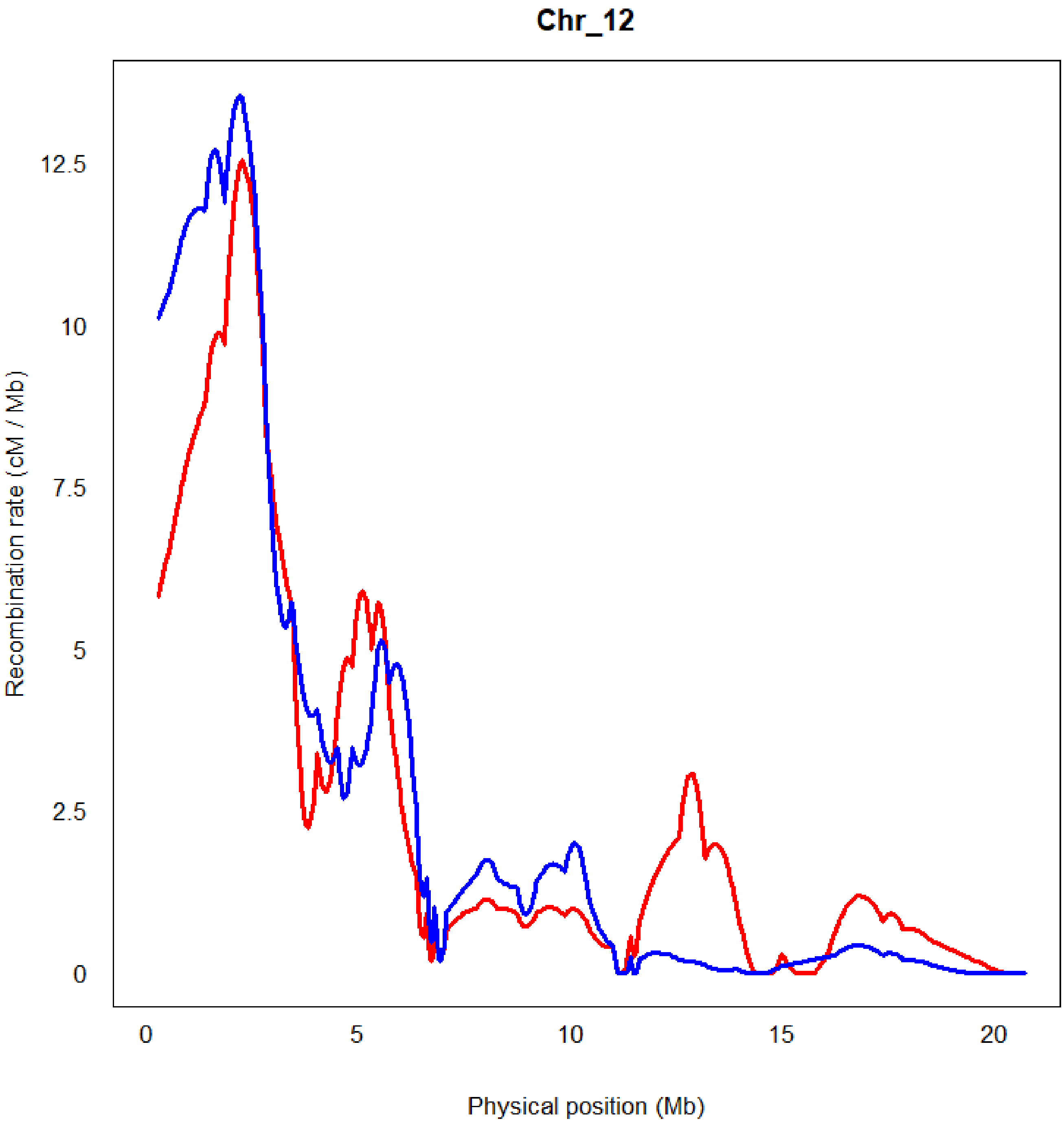
Chr_12
12.5
10
7.5
5
2.5
0
Recombination rate (cM / Mb)
0
5
10
15
20
Physical position (Mb)

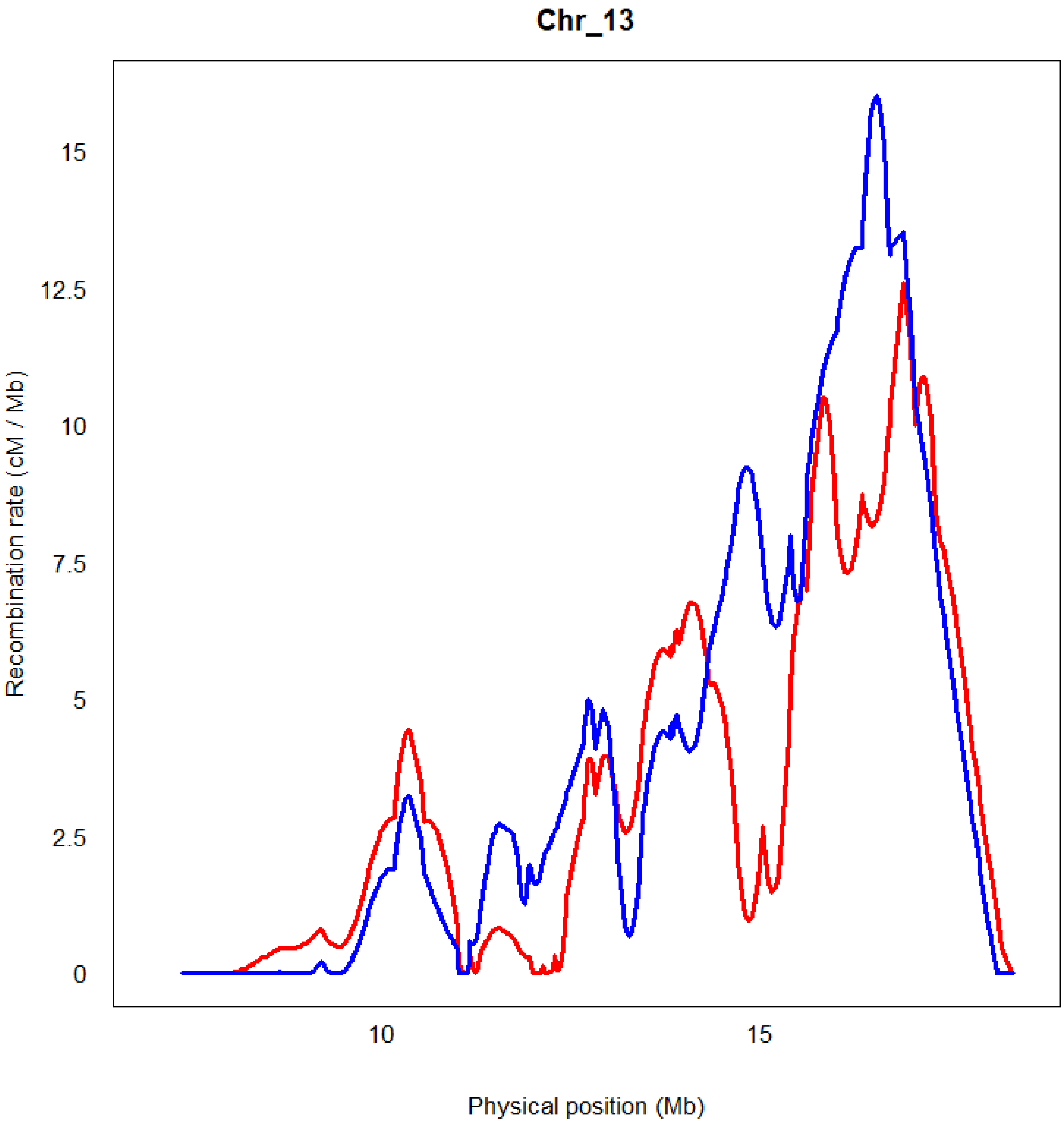
Chr_13
Recombination rate (cM / Mb)
0
2.5
5
7.5
10
12.5
15
10
15
Physical position (Mb)

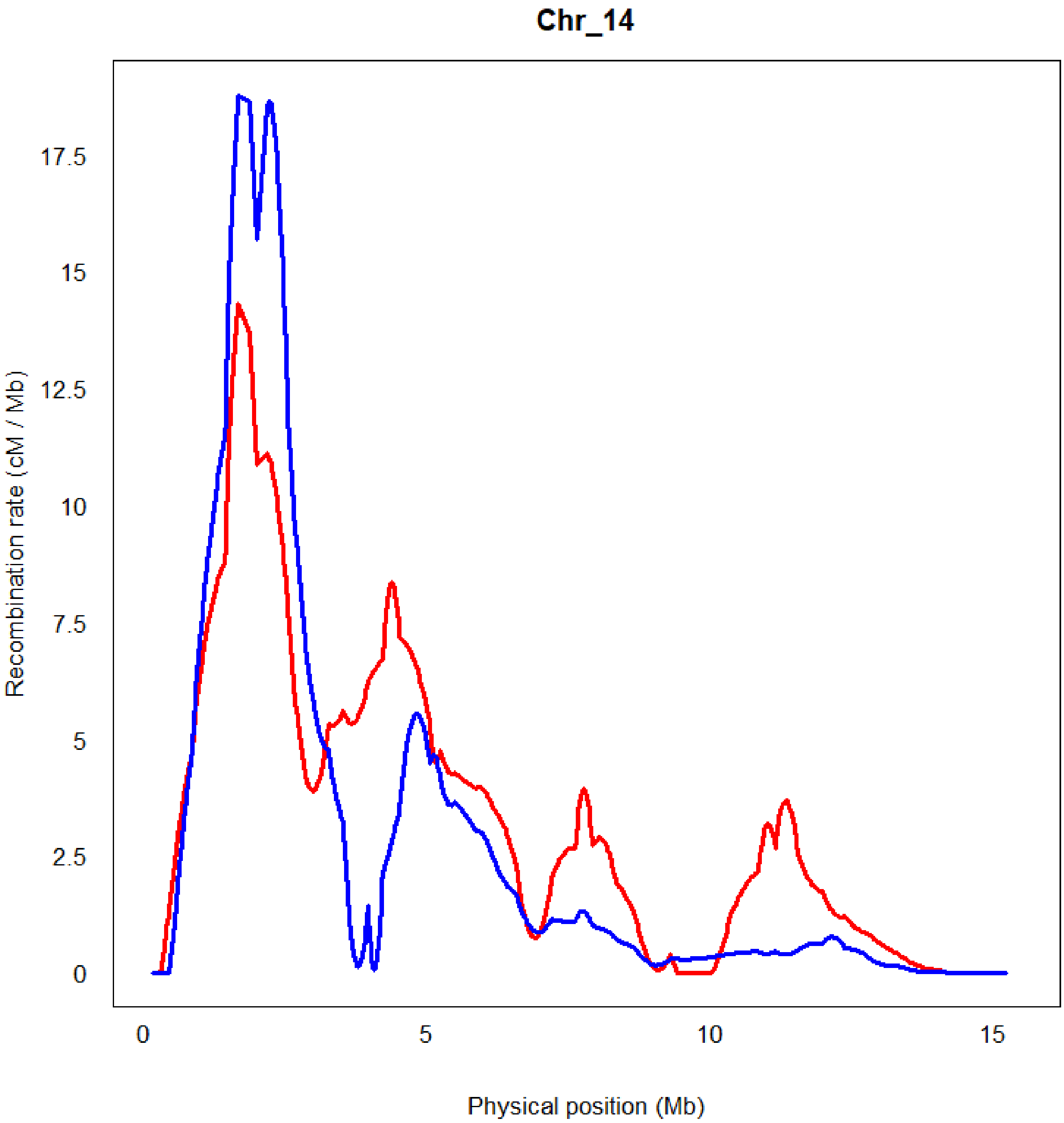
Chr_14
Recombination rate (cM / Mb)
0
2.5
5
7.5
10
12.5
15
17.5
0
5
10
15
Physical position (Mb)

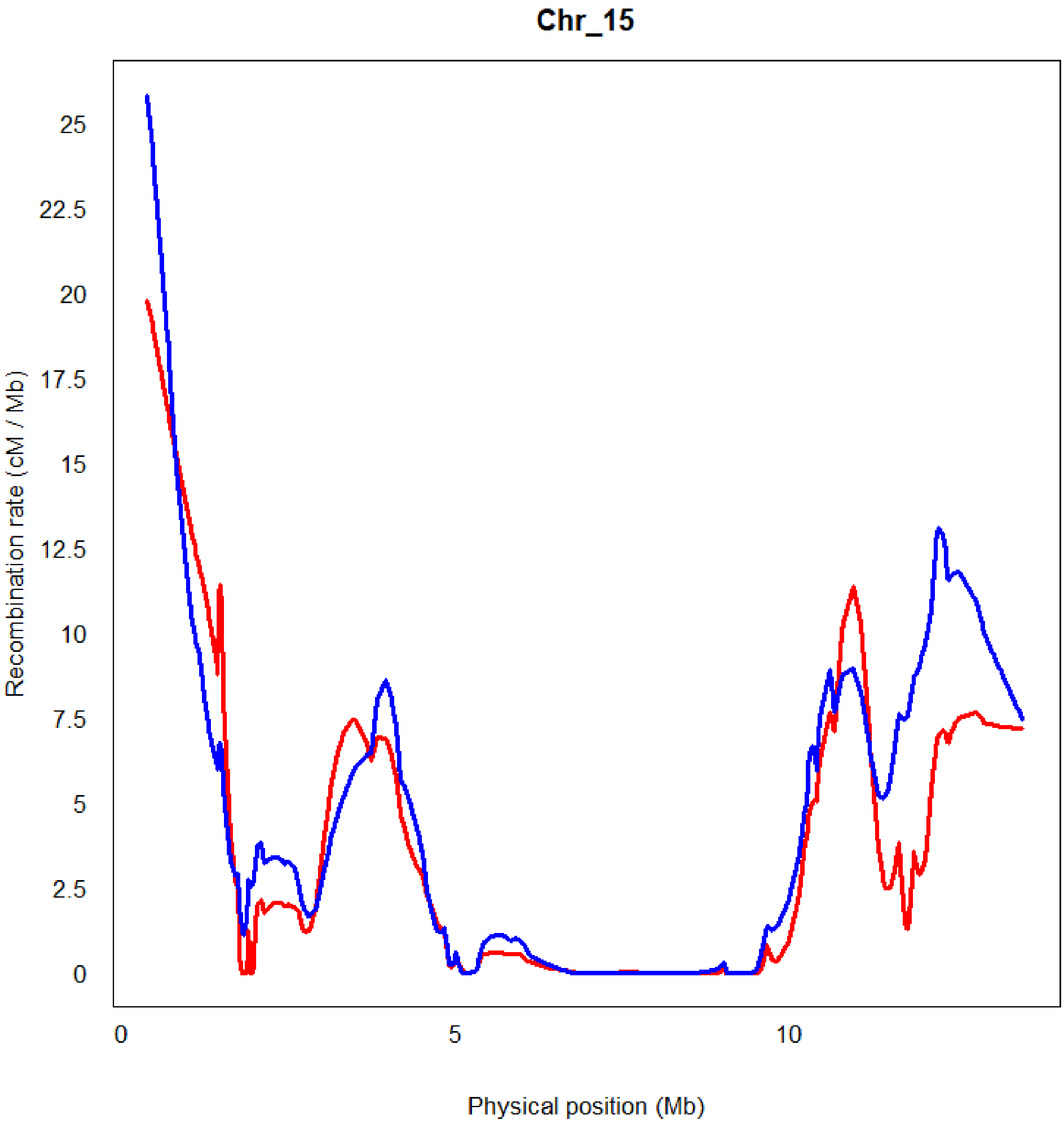
Chr_15
Recombination rate (cM / Mb)
25
22.5
20
17.5
15
12.5
10
7.5
5
2.5
0
0
5
10
Physical position (Mb)

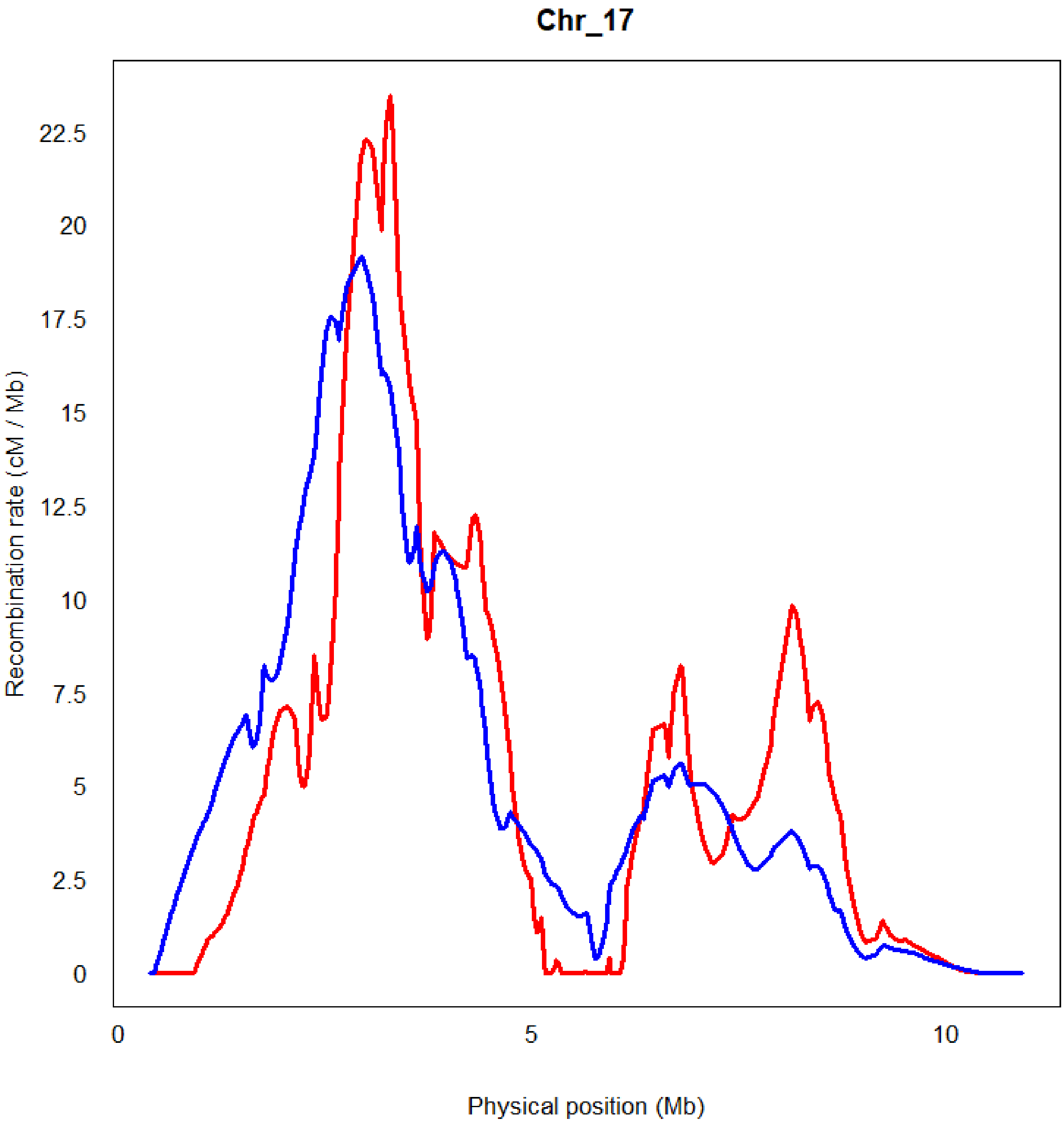

Chr_17
Recombination rate (cM / Mb)
Physical position (Mb)
0
2.5
5
7.5
10
12.5
15
17.5
20
22.5
0
5
10

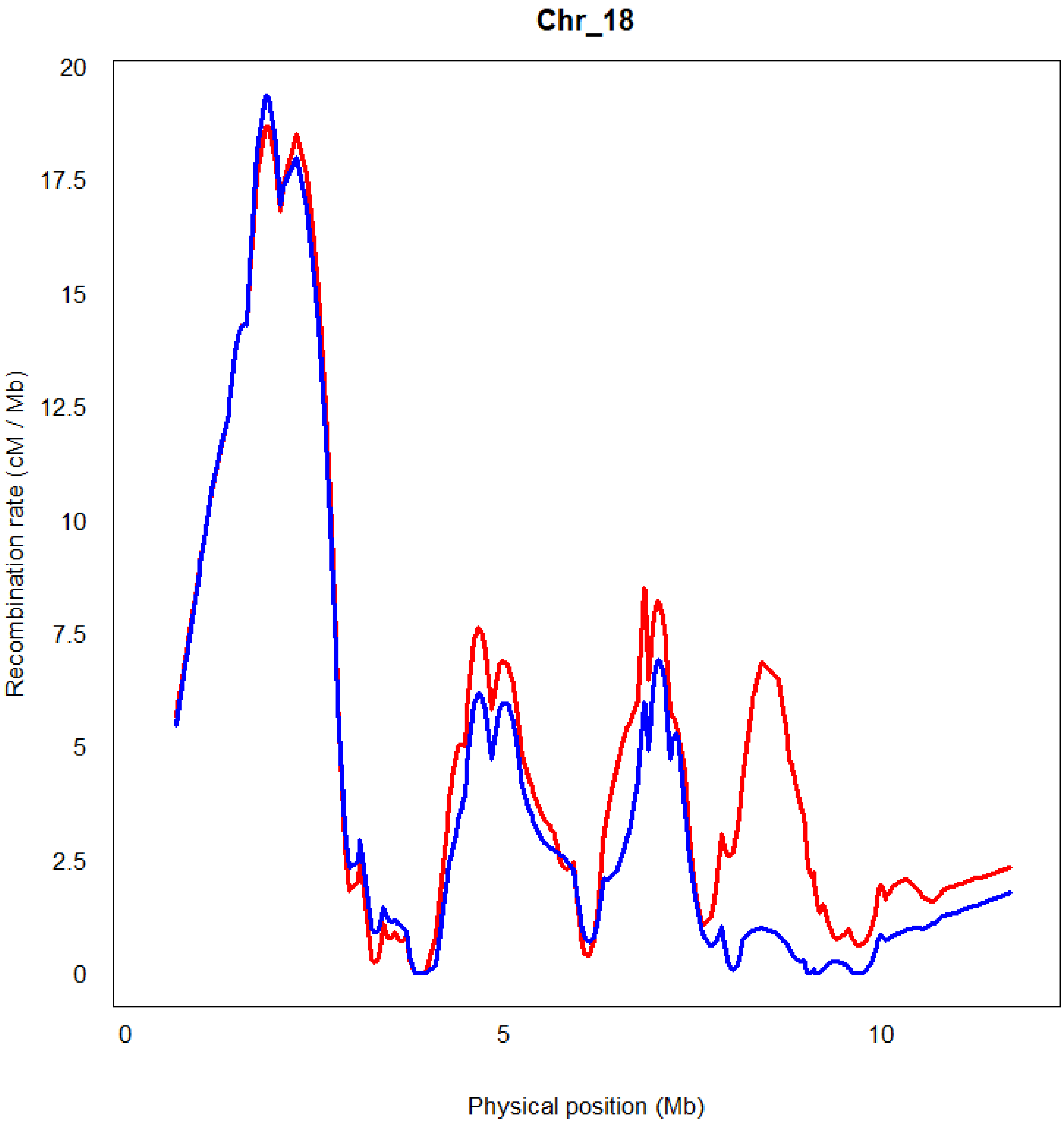

Chr_18
Recombination rate (cM / Mb)
20
17.5
15
12.5
10
7.5
5
2.5
0
0
5
10
Physical position (Mb)

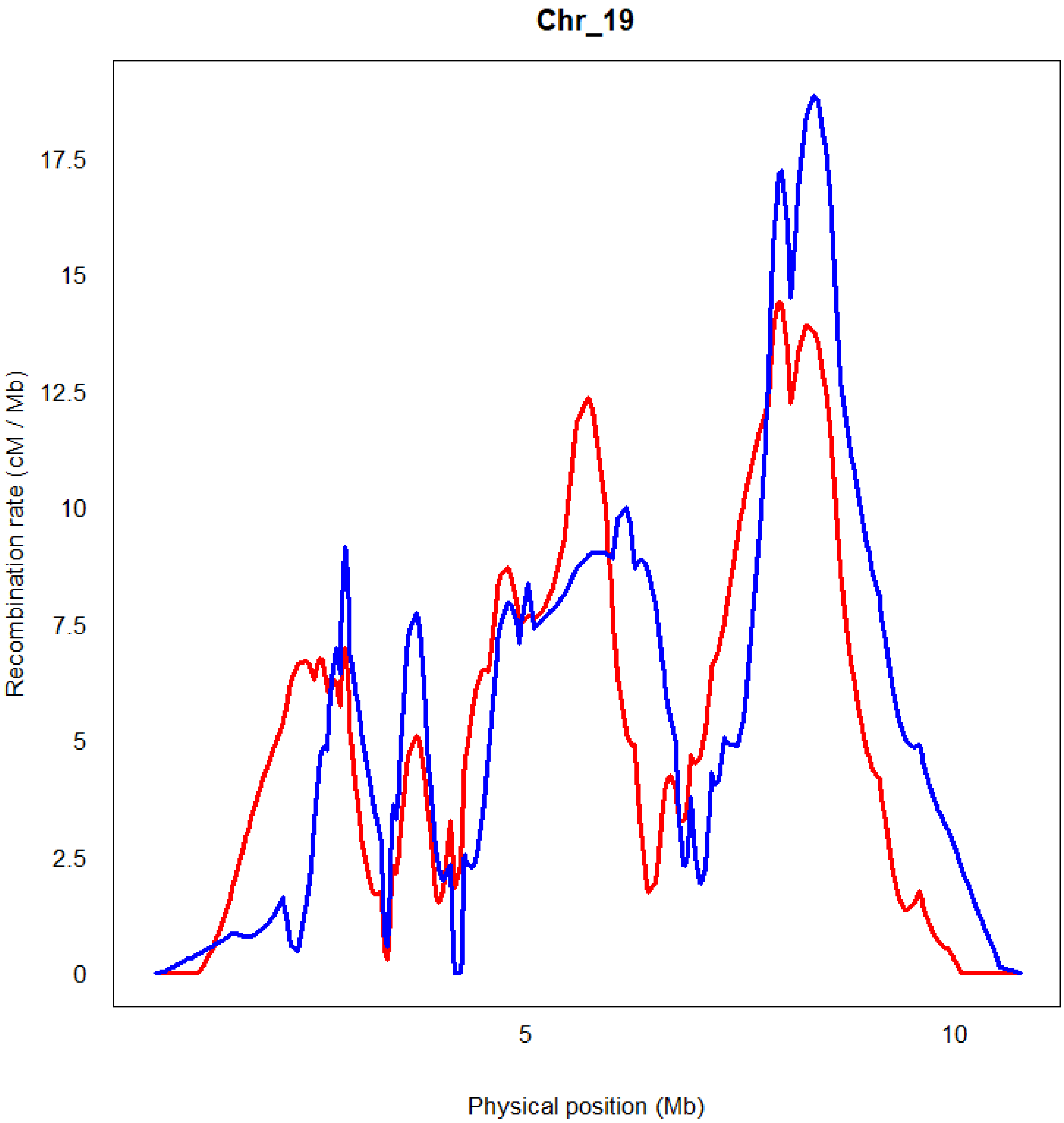

Chr_19
Recombination rate (cM / Mb)
0
2.5
5
7.5
10
12.5
15
17.5
5
10
Physical position (Mb)

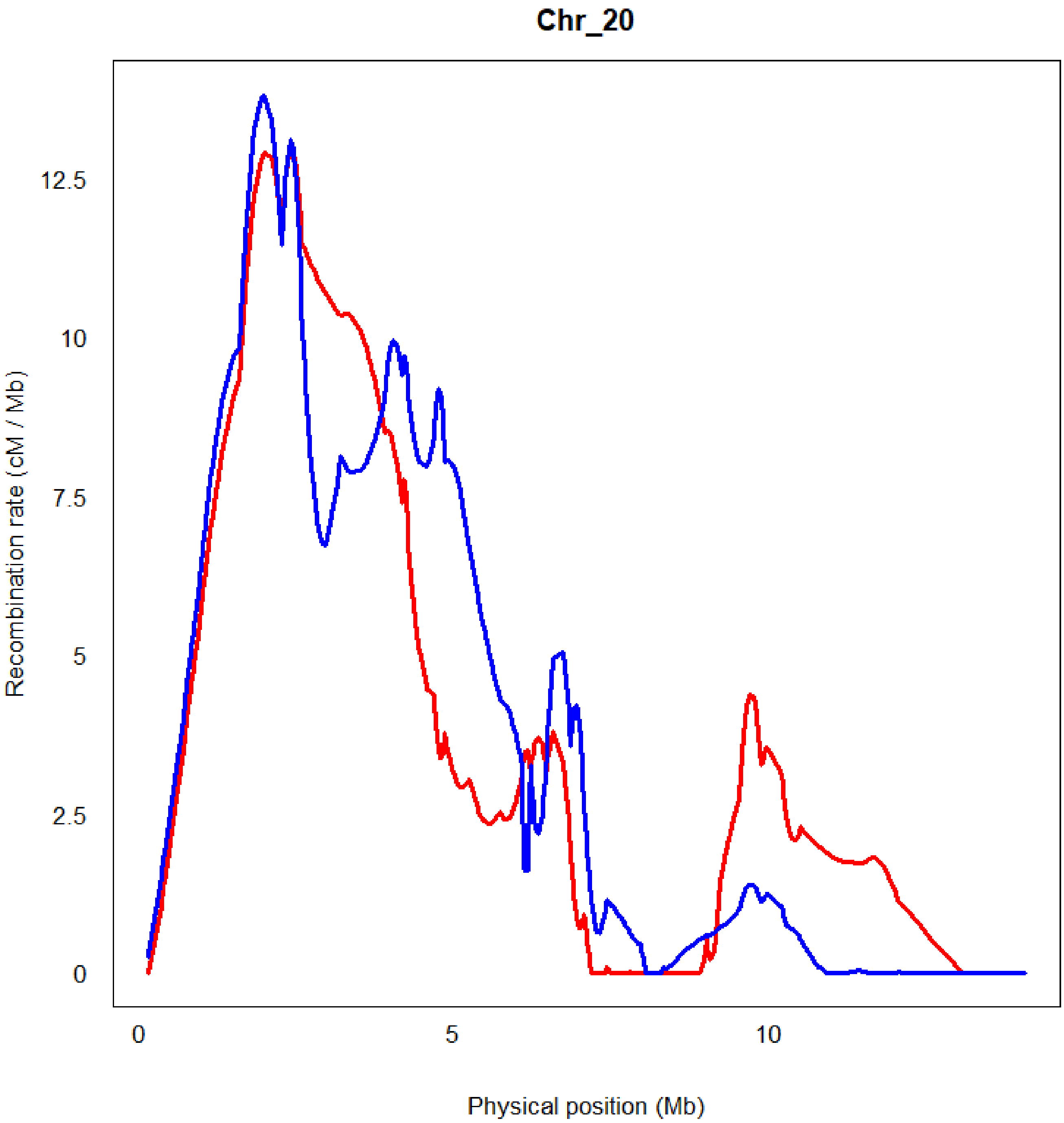

Chr_20
Recombination rate (cM / Mb)
12.5
10
7.5
5
2.5
0
0
5
10
Physical position (Mb)

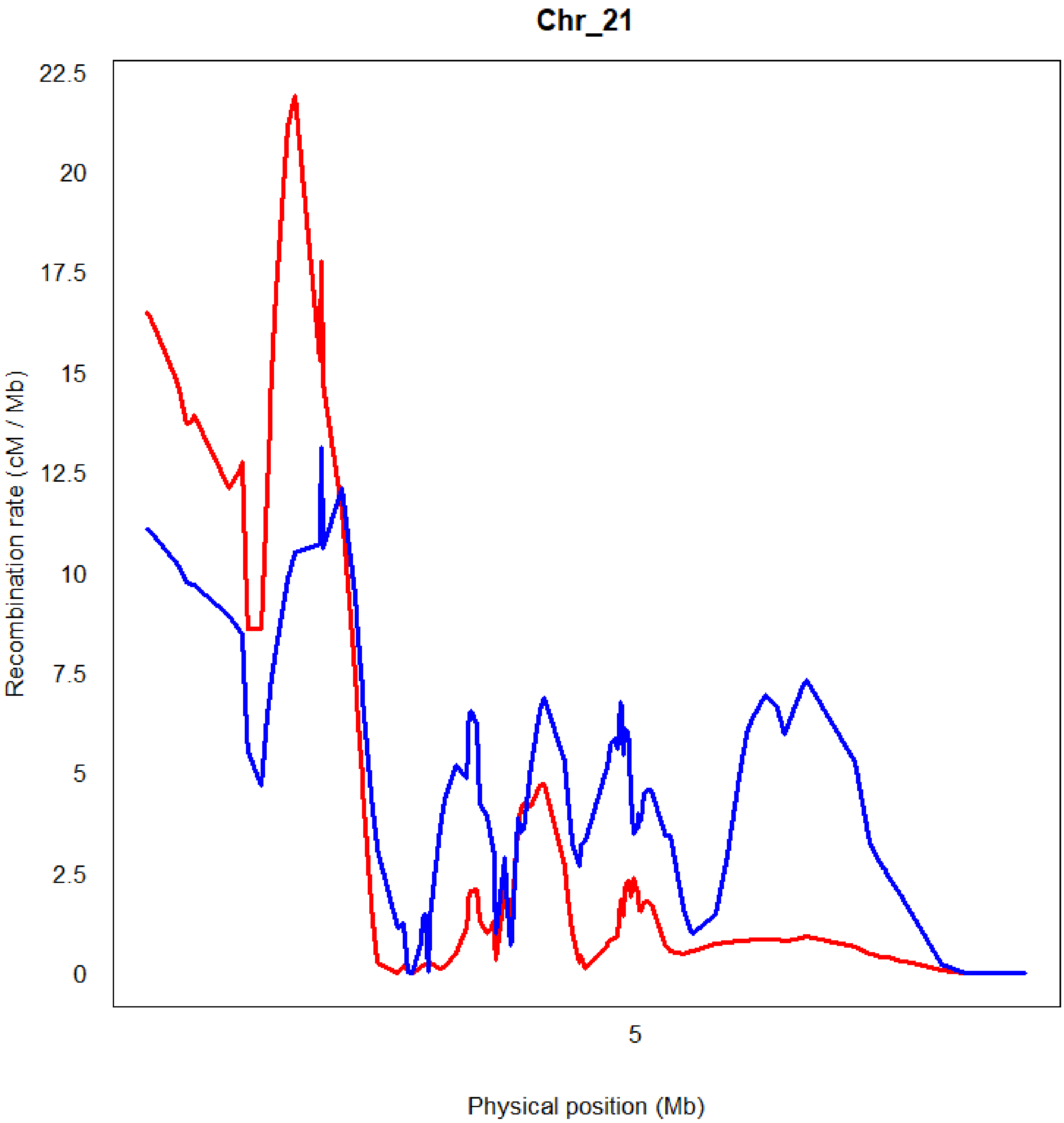
Chr_21
22.5
20
17.5
15
12.5
10
7.5
5
2.5
0
Recombination rate (cM / Mb)
5
Physical position (Mb)

Chr_23

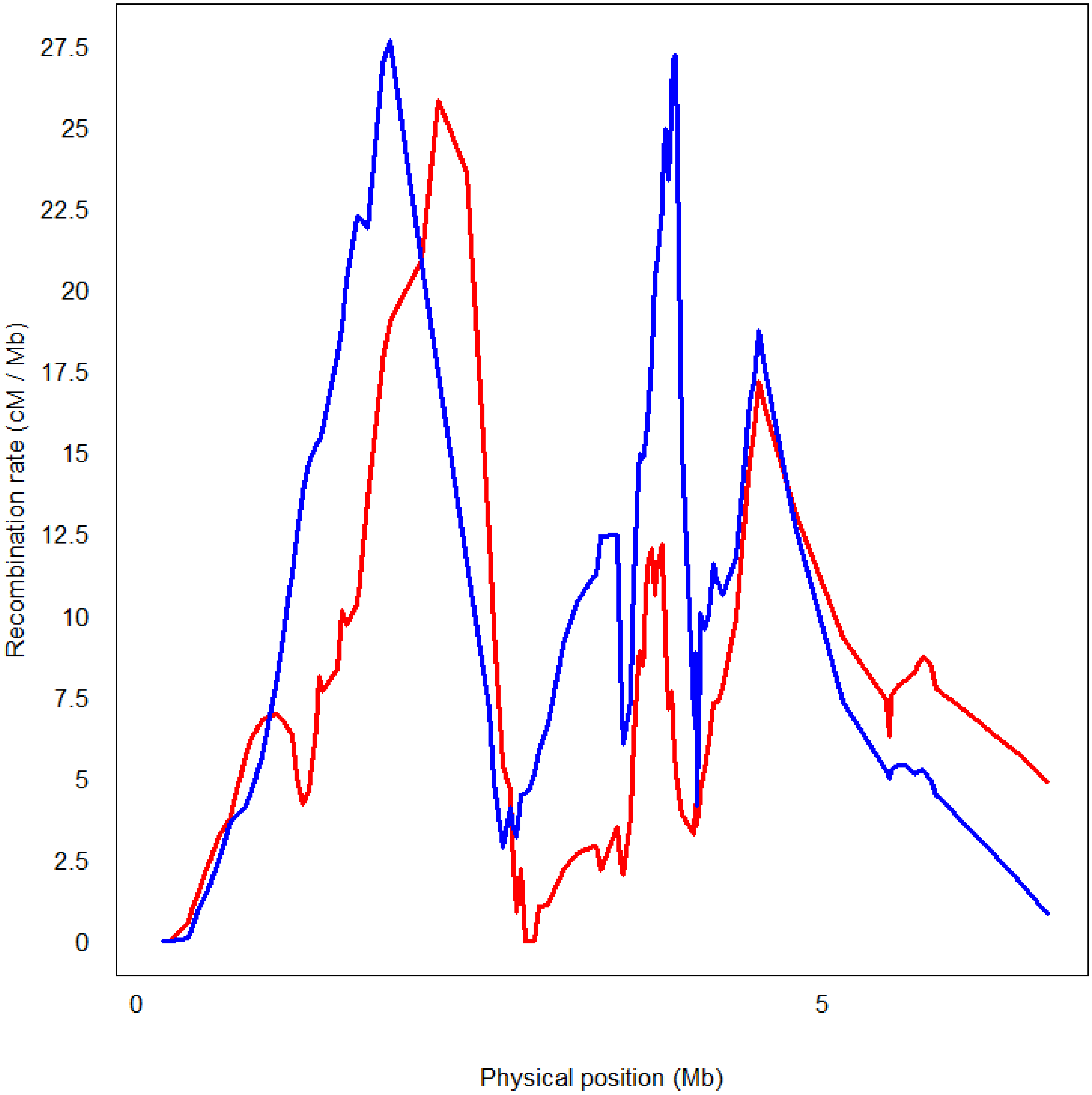

27.5
25
22.5
20
17.5
15
12.5
10
7.5
5
2.5
0
Recombination rate (cM / Mb)
0
5
Physical position (Mb)

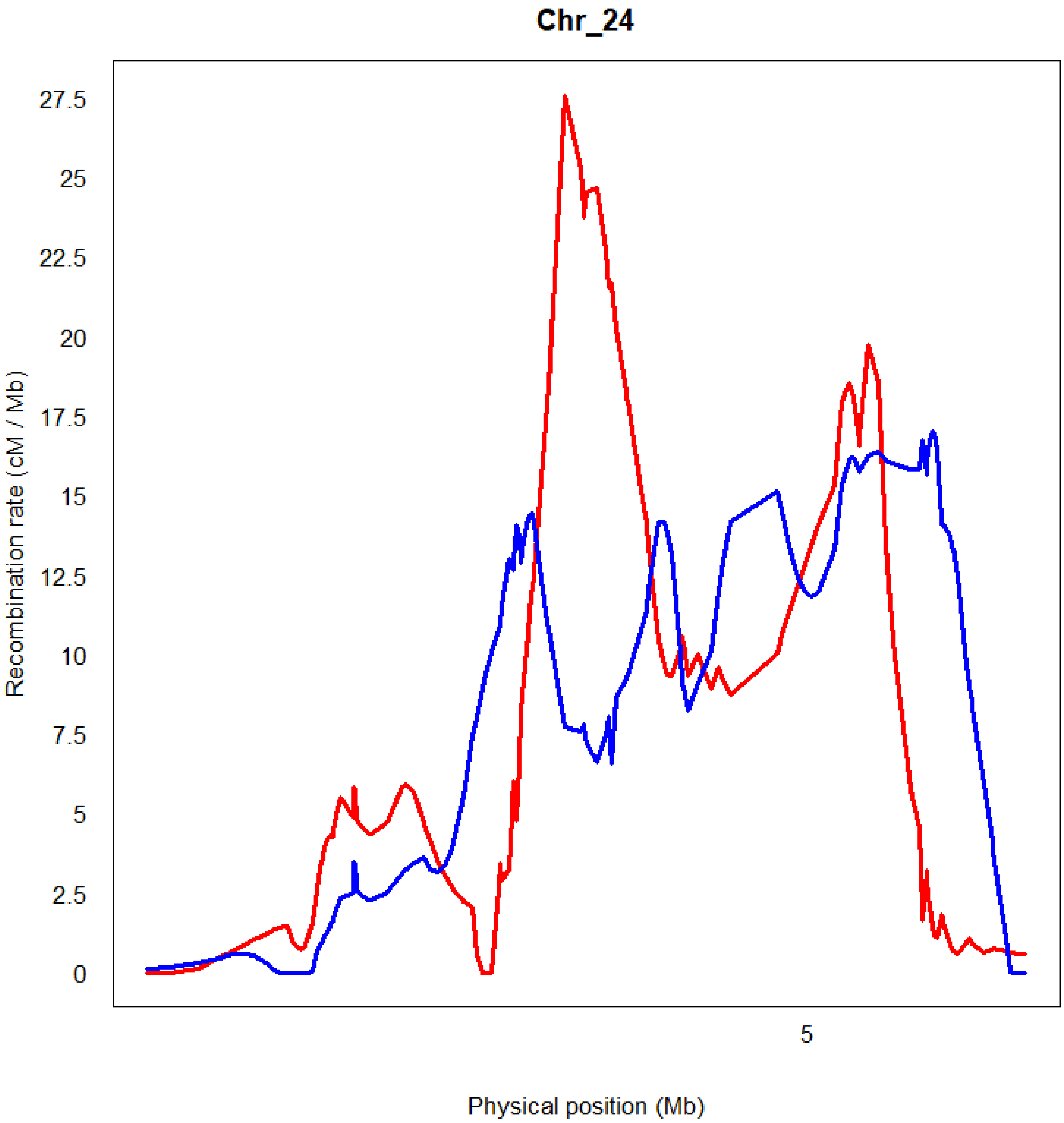

Chr_24
27.5
25
22.5
20
17.5
15
12.5
10
7.5
5
2.5
0
Recombination rate (cM / Mb)
5
Physical position (Mb)
