# Supplementary material for: Chromosome-length genome assembly and linkage map of a critically endangered Australian bird: the helmeted honeyeater
Source: Gigascience. 2022 Mar 29;11:giac025. doi: 10.1093/gigascience/giac025 (PMC8963300; doi:10.1093/gigascience/giac025)
Supplement: giac025_Supplemental_Files [file giac025_supplemental_files.zip › Supplementary_Material_S5.pdf]

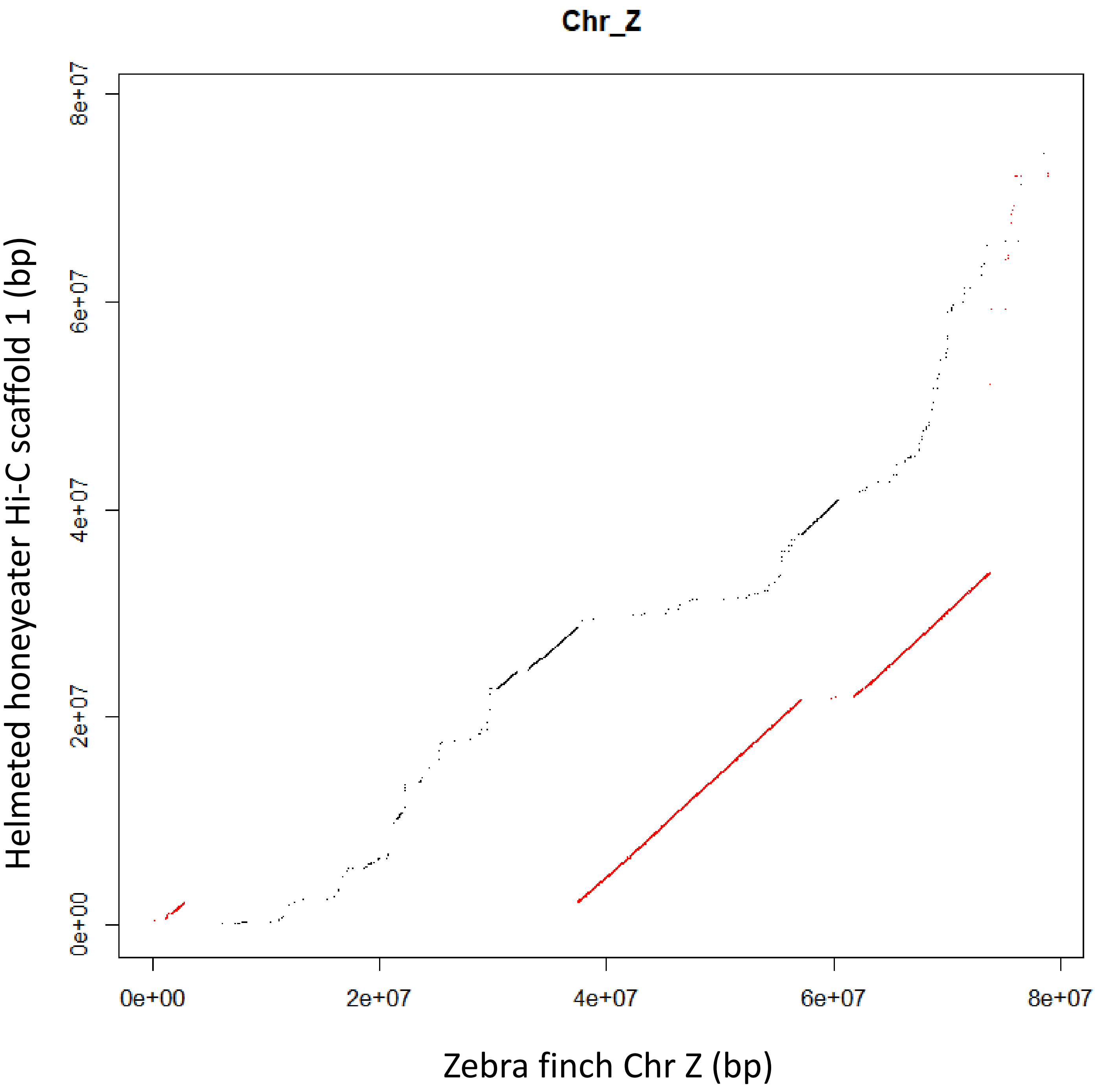
Chr_Z
Helmeted honeyeater Hi-C scaffold 1 (bp)
0e+00
2e+07
4e+07
6e+07
8e+07
Zebra finch Chr Z (bp)

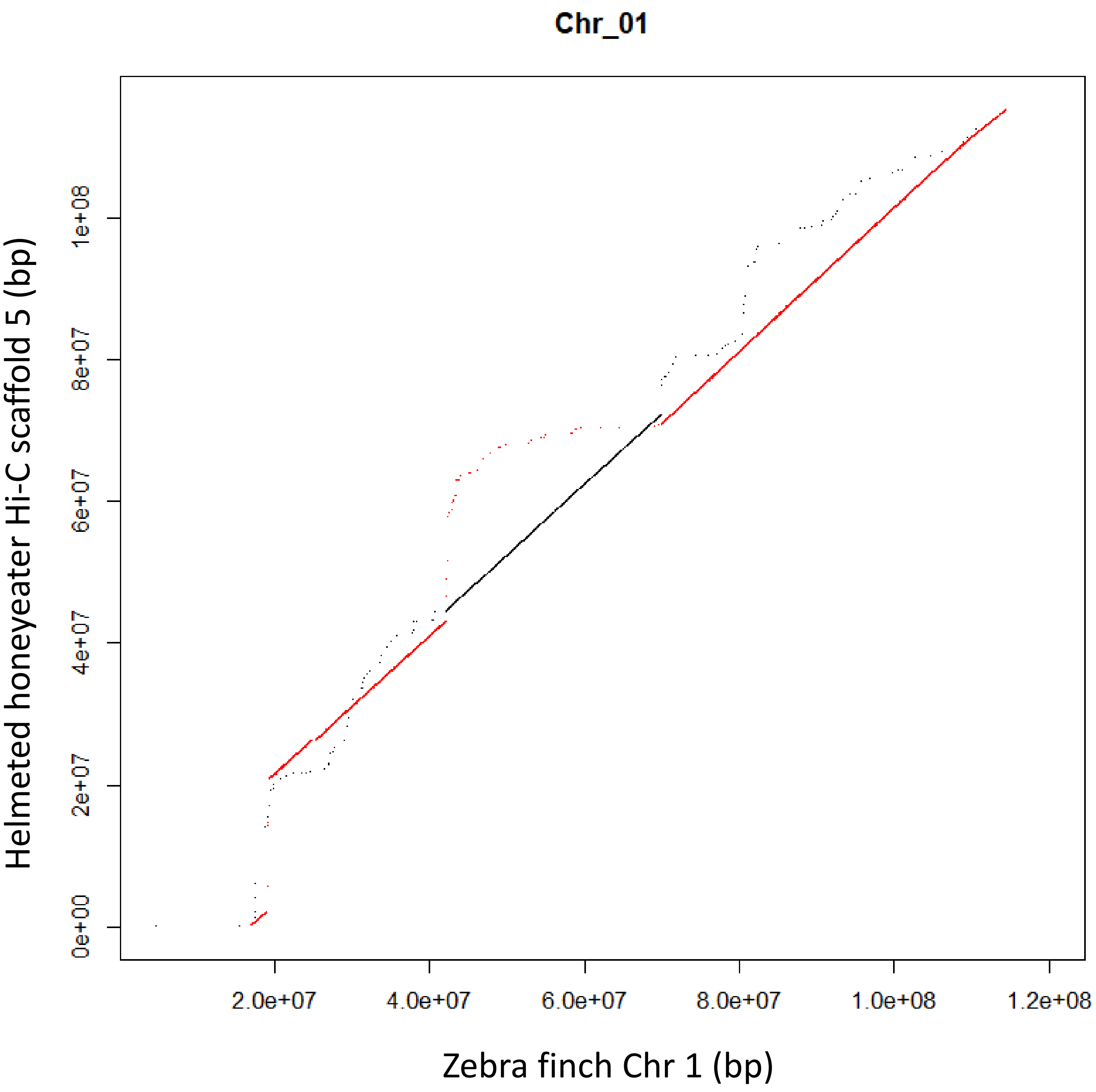

Chr_01
Helmeted honeyeater Hi-C scaffold 5 (bp)
0e+00
2e+07
4e+07
6e+07
8e+07
1e+08
2.0e+07
4.0e+07
6.0e+07
8.0e+07
1.0e+08
1.2e+08
Zebra finch Chr 1 (bp)

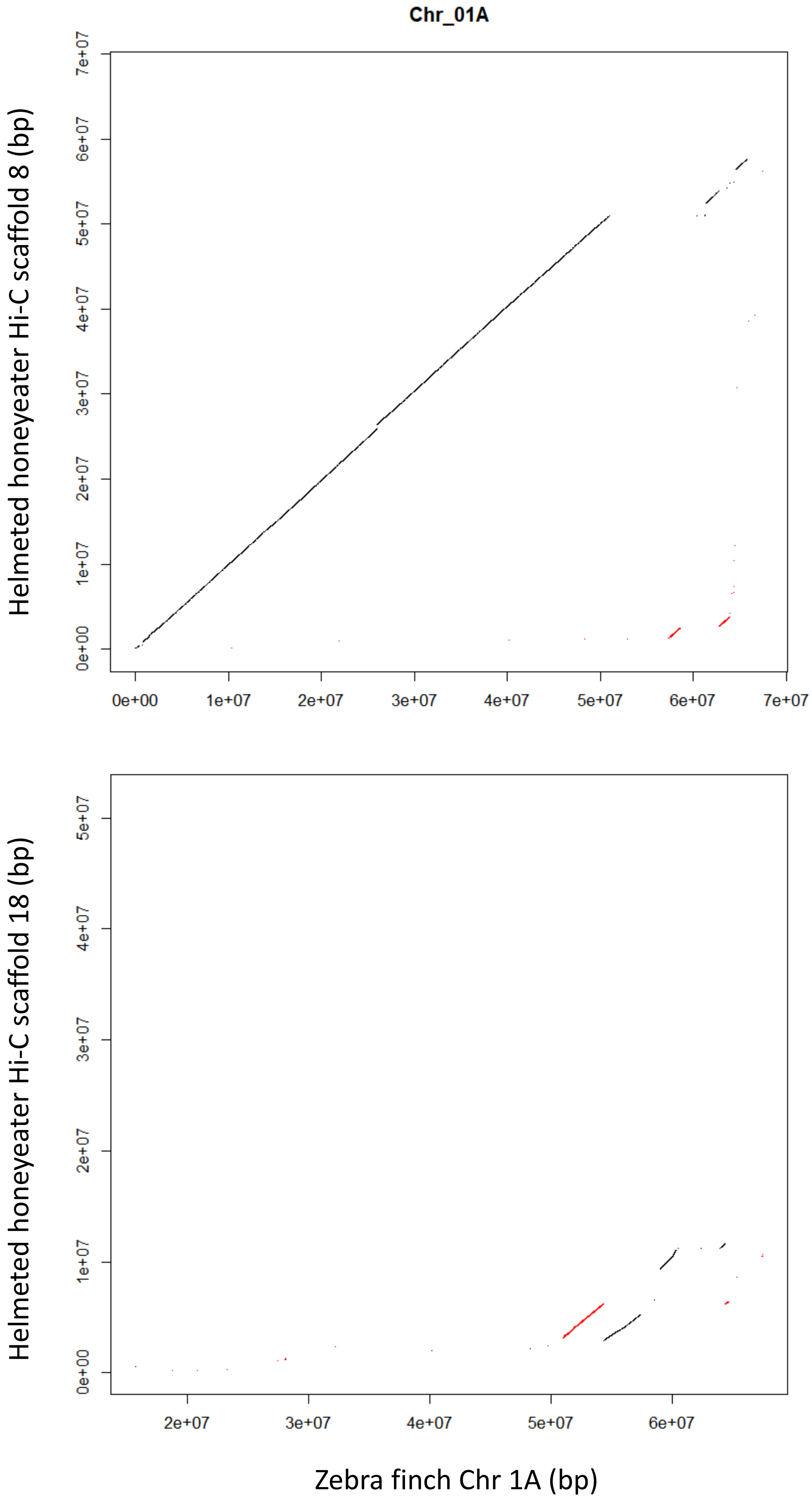

Chr_01A
Helmeted honeyeater Hi-C scaffold 8 (bp)
0e+00
1e+07
2e+07
3e+07
4e+07
5e+07
6e+07
7e+07
0e+00
1e+07
2e+07
3e+07
4e+07
5e+07
6e+07
7e+07
Helmeted honeyeater Hi-C scaffold 18 (bp)
0e+00
1e+07
2e+07
3e+07
4e+07
5e+07
2e+07
3e+07
4e+07
5e+07
6e+07
Zebra finch Chr 1A (bp)

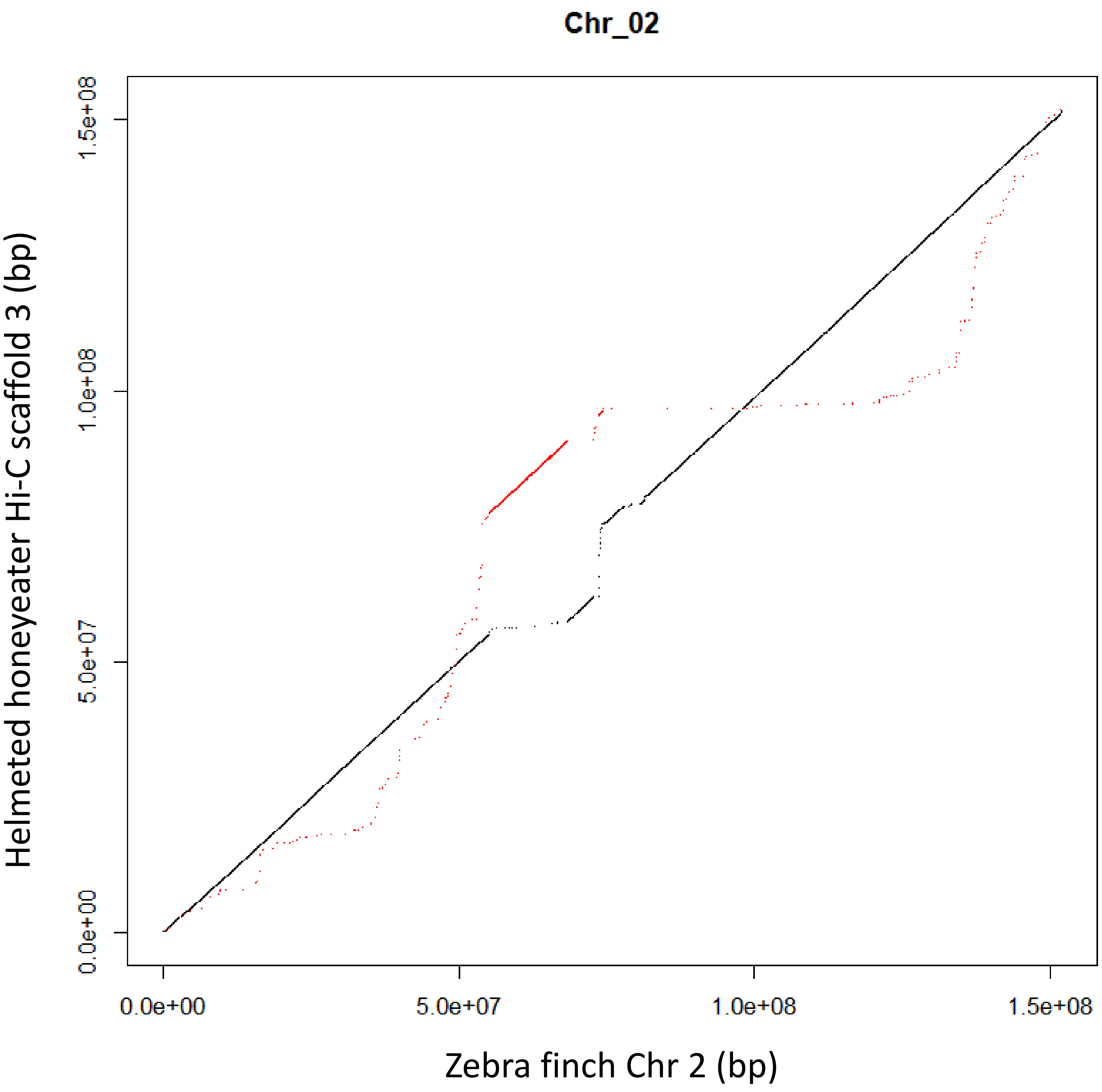
Chr_02
Helmeted honeyeater Hi-C scaffold 3 (bp)
0.0e+00
5.0e+07
1.0e+08
1.5e+08
Zebra finch Chr 2 (bp)
0.0e+00
5.0e+07
1.0e+08
1.5e+08

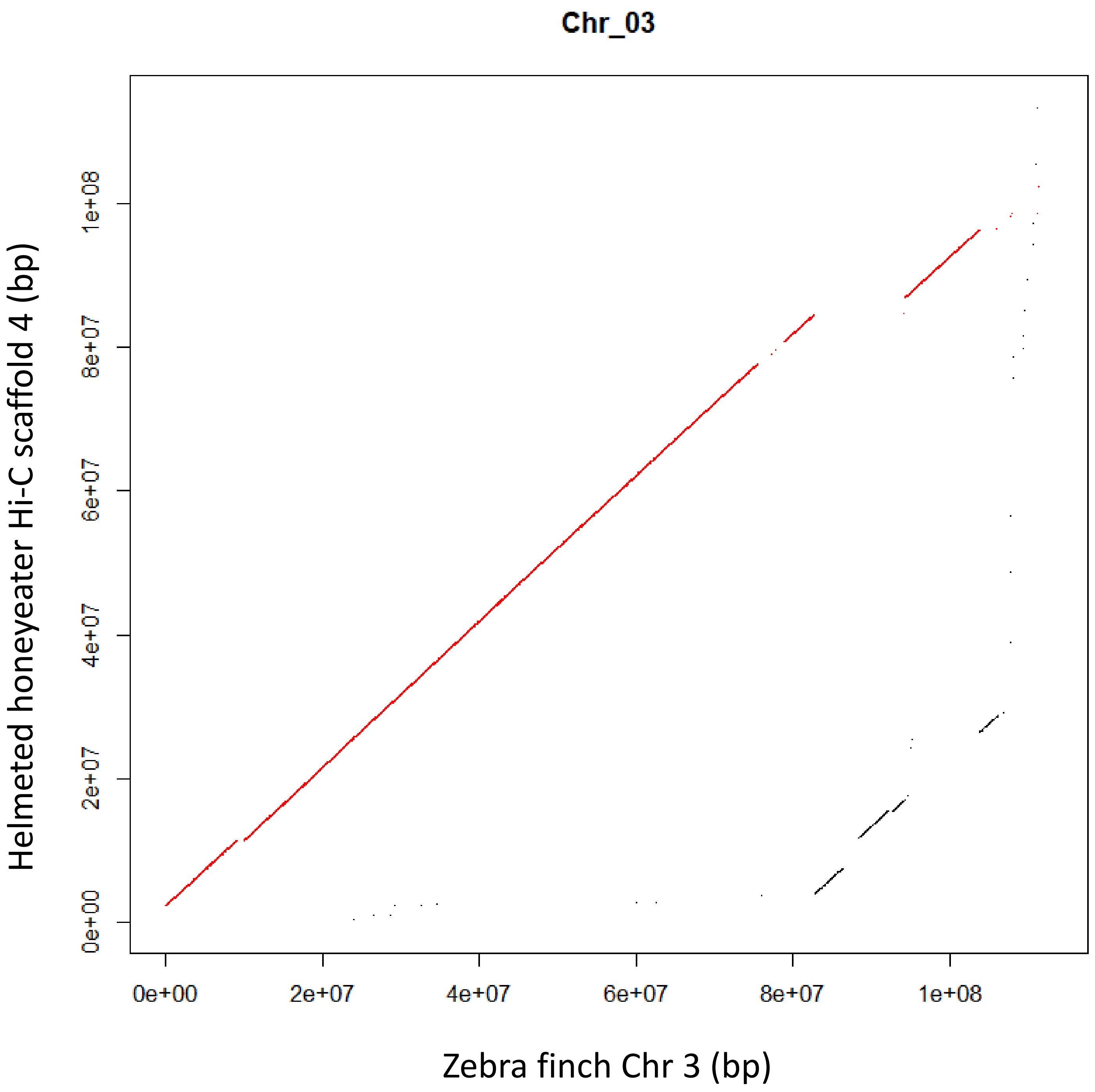
Chr_03
Helmeted honeyeater Hi-C scaffold 4 (bp)
0e+00
2e+07
4e+07
6e+07
8e+07
1e+08
0e+00
2e+07
4e+07
6e+07
8e+07
1e+08
Zebra finch Chr 3 (bp)

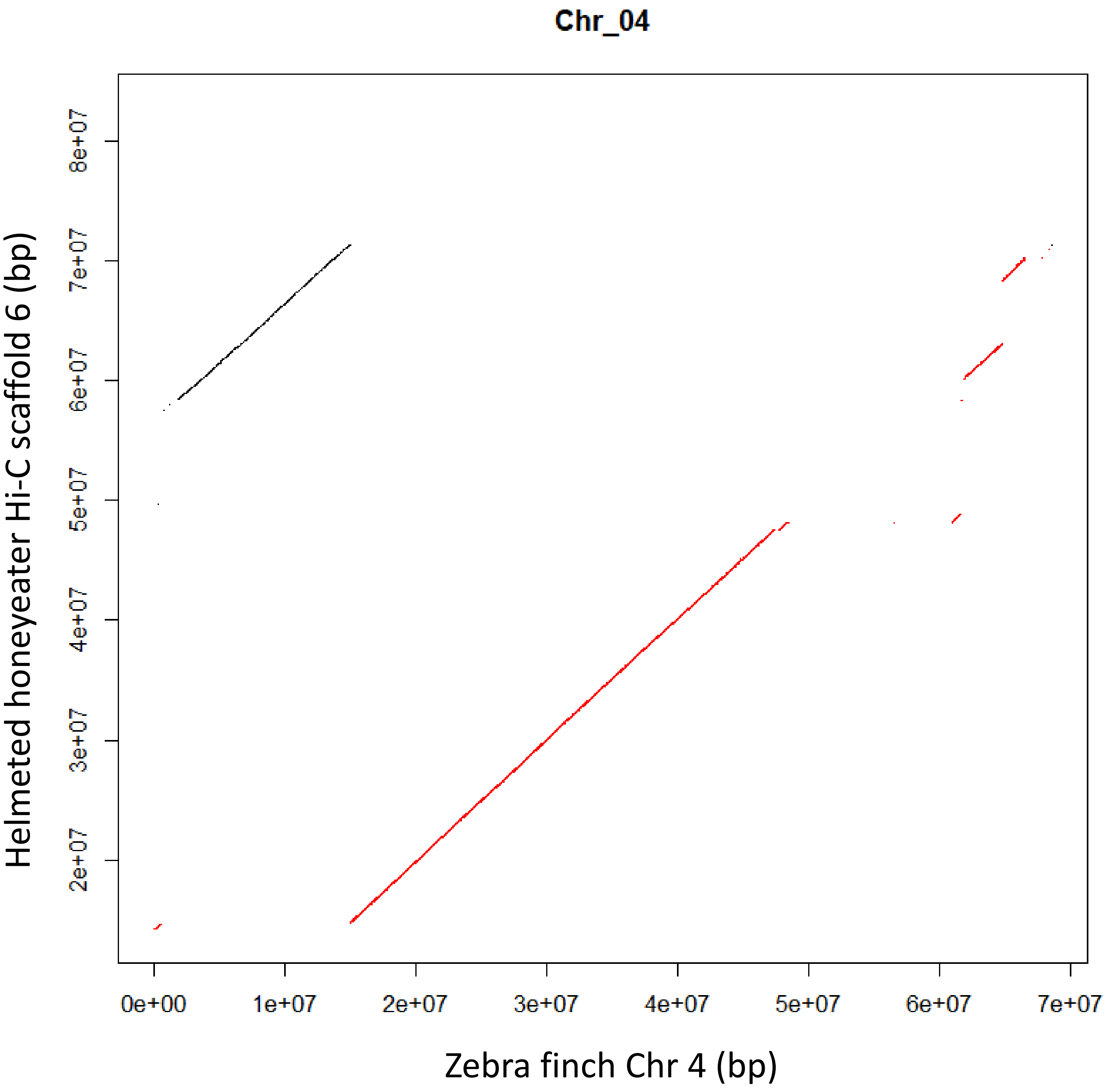

Chr_04
Helmeted honeyeater Hi-C scaffold 6 (bp)
8e+07
7e+07
6e+07
5e+07
4e+07
3e+07
2e+07
0e+00
1e+07
2e+07
3e+07
4e+07
5e+07
6e+07
7e+07
Zebra finch Chr 4 (bp)

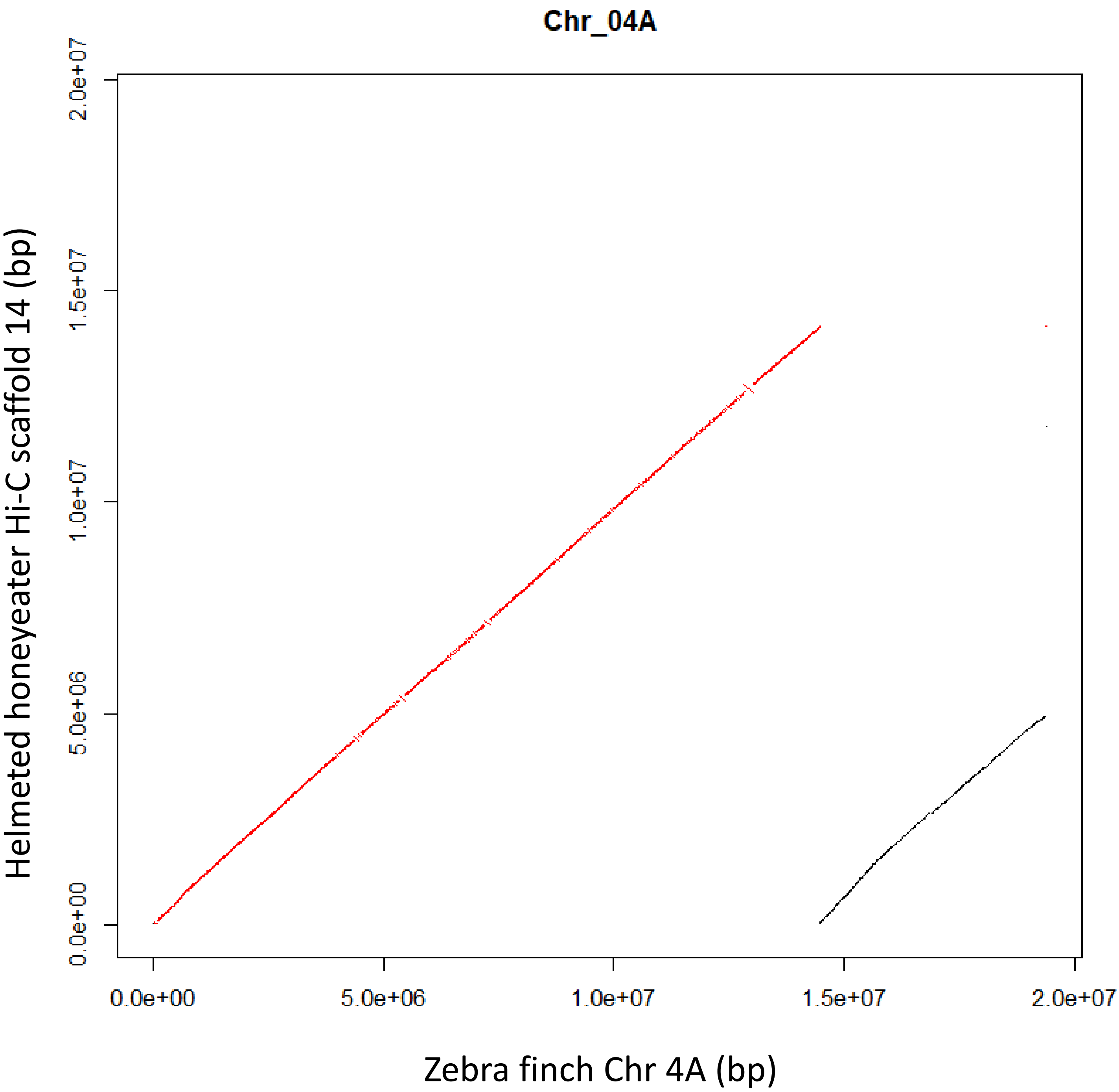
Chr_04A
Helmeted honeyeater Hi-C scaffold 14 (bp)
0.0e+00
5.0e+06
1.0e+07
1.5e+07
2.0e+07
0.0e+00
5.0e+06
1.0e+07
1.5e+07
2.0e+07
Zebra finch Chr 4A (bp)

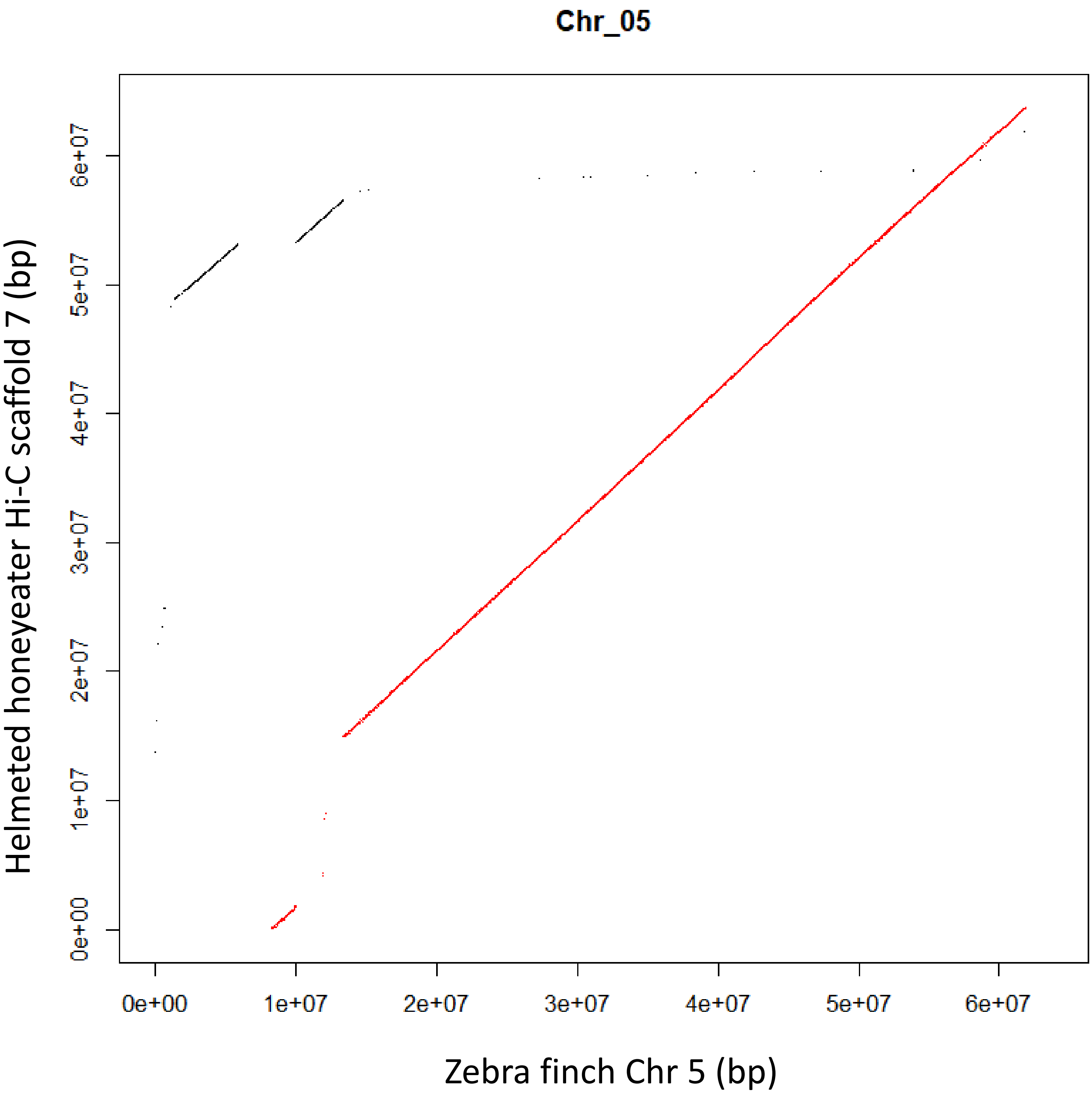
Chr_05
Helmeted honeyeater Hi-C scaffold 7 (bp)
0e+00
1e+07
2e+07
3e+07
4e+07
5e+07
6e+07
0e+00
1e+07
2e+07
3e+07
4e+07
5e+07
6e+07
Zebra finch Chr 5 (bp)

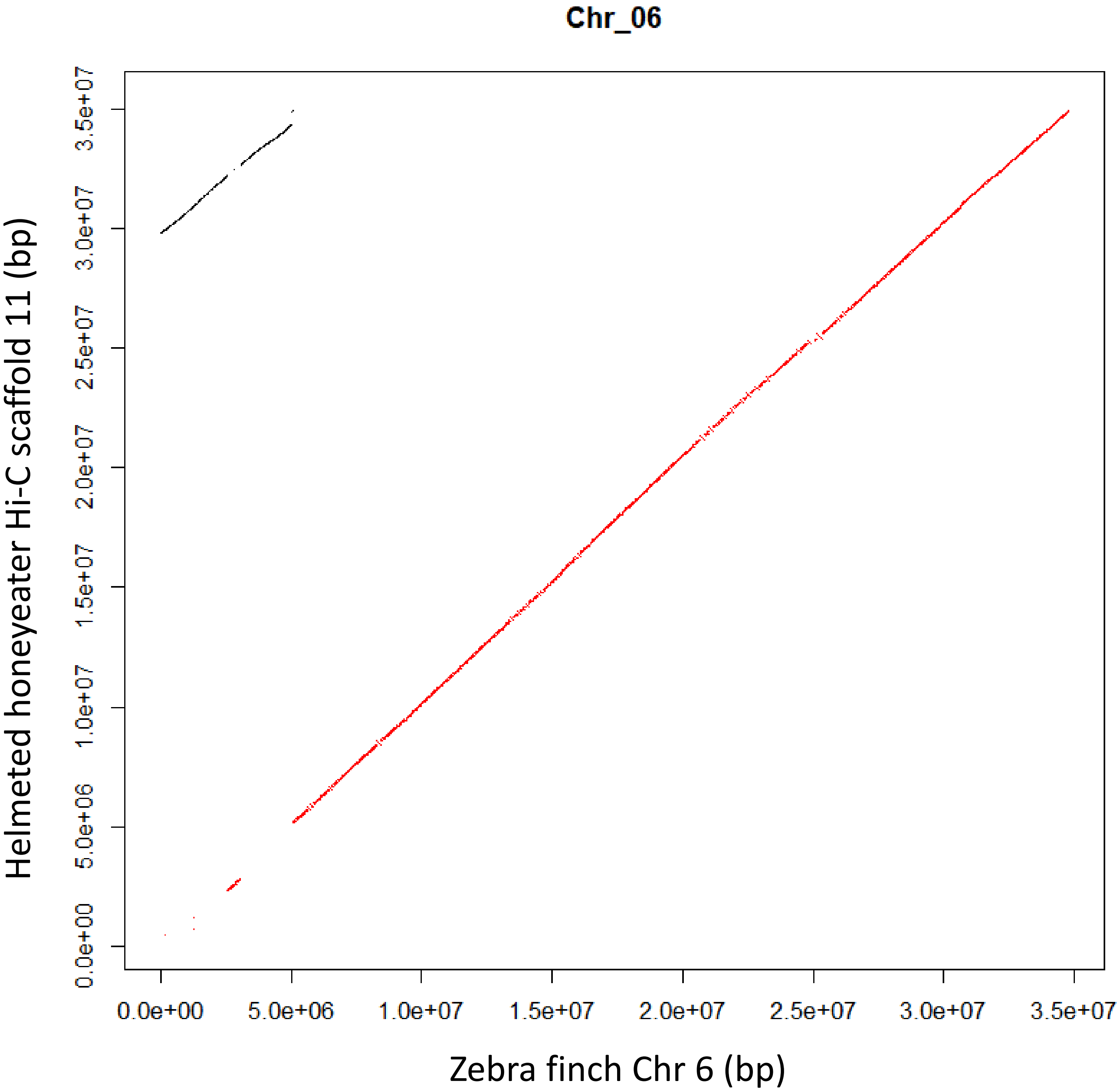

Chr_06
Helmeted honeyeater Hi-C scaffold 11 (bp)
0.0e+00
5.0e+06
1.0e+07
1.5e+07
2.0e+07
2.5e+07
3.0e+07
3.5e+07
Zebra finch Chr 6 (bp)
0.0e+00
5.0e+06
1.0e+07
1.5e+07
2.0e+07
2.5e+07
3.0e+07
3.5e+07

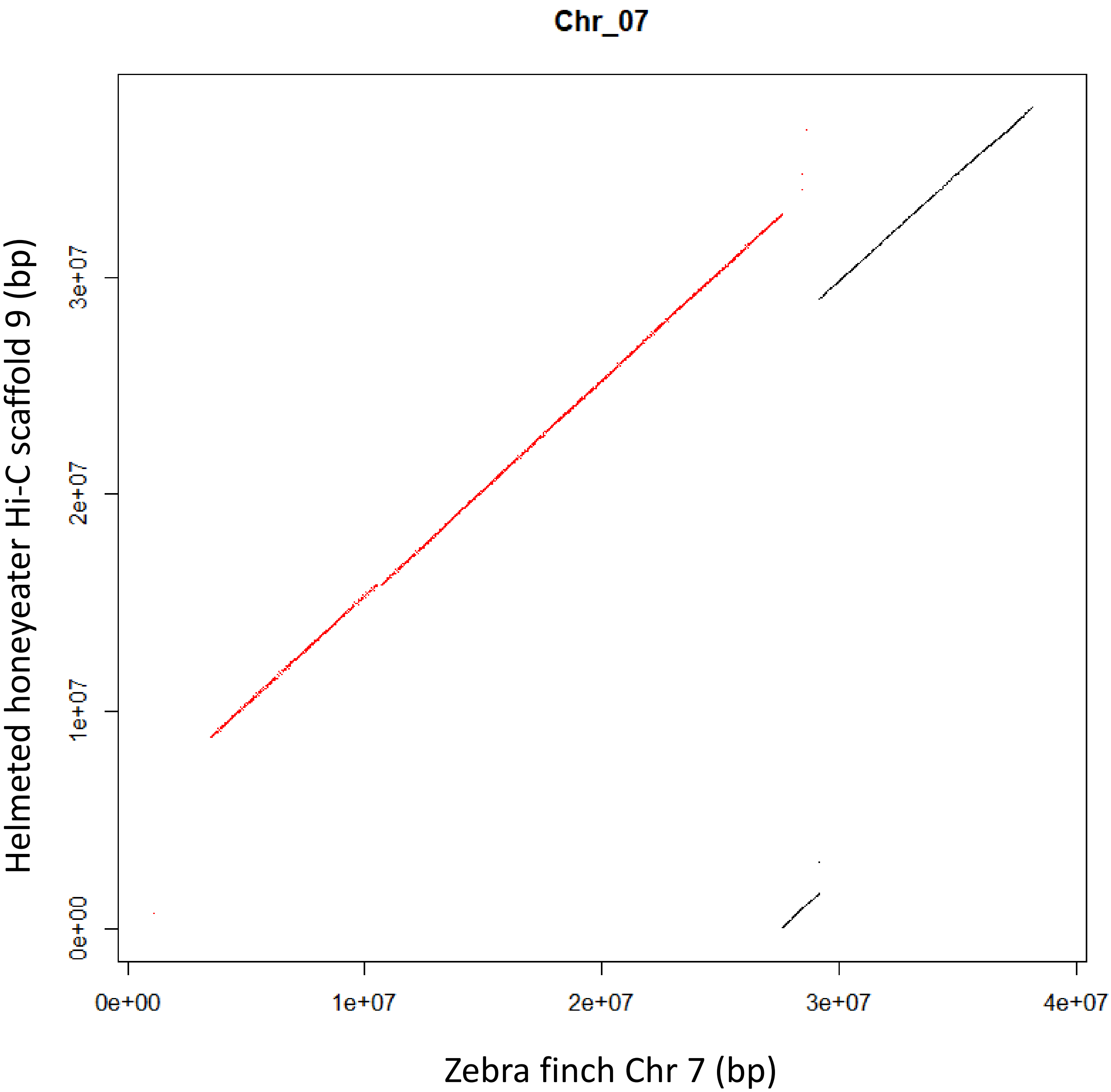

Chr_07
Helmeted honeyeater Hi-C scaffold 9 (bp)
0e+00
1e+07
2e+07
3e+07
0e+00
1e+07
2e+07
3e+07
4e+07
Zebra finch Chr 7 (bp)

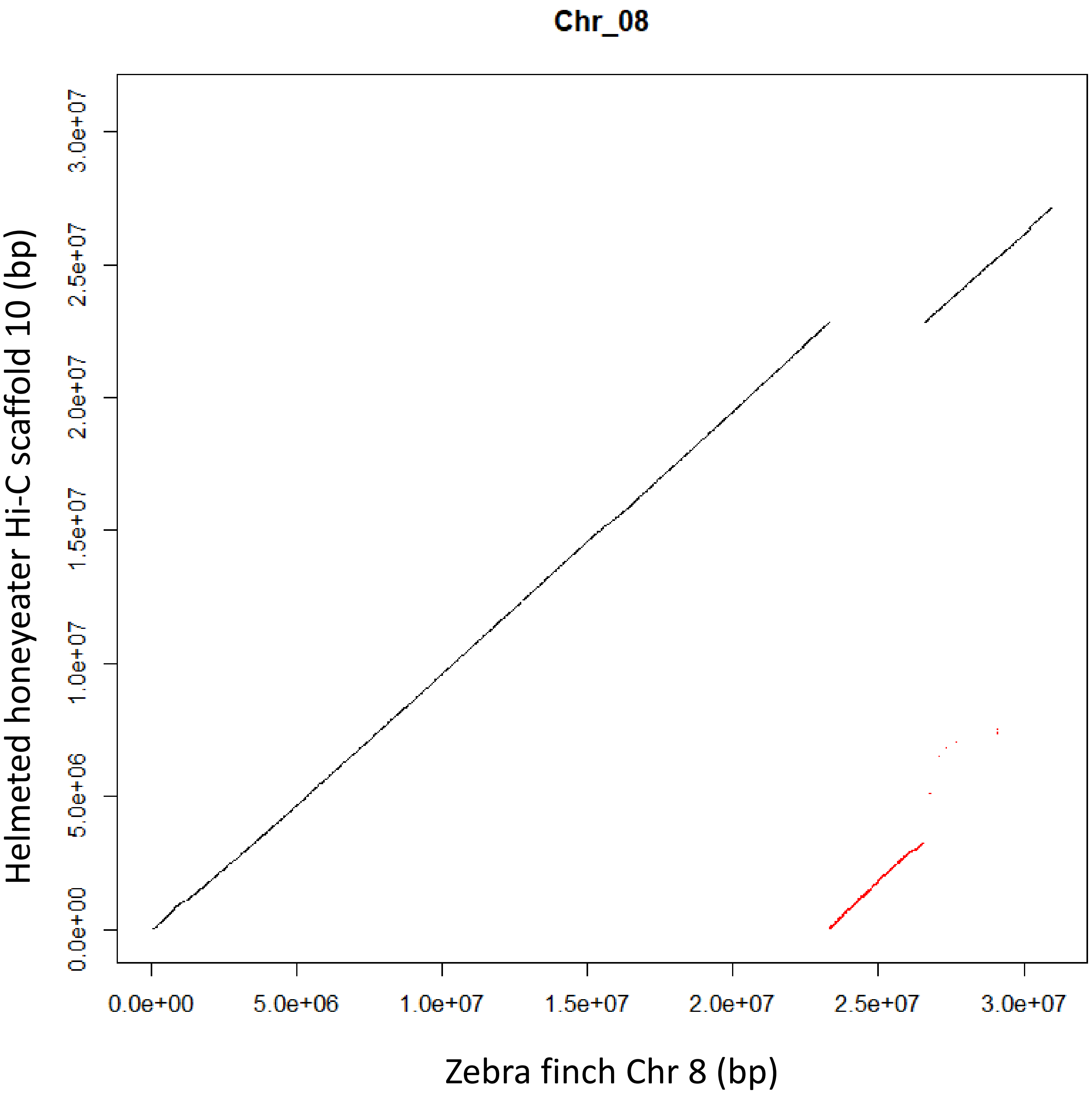
Chr_08
Helmeted honeyeater Hi-C scaffold 10 (bp)
0.0e+00
5.0e+06
1.0e+07
1.5e+07
2.0e+07
2.5e+07
3.0e+07
Zebra finch Chr 8 (bp)

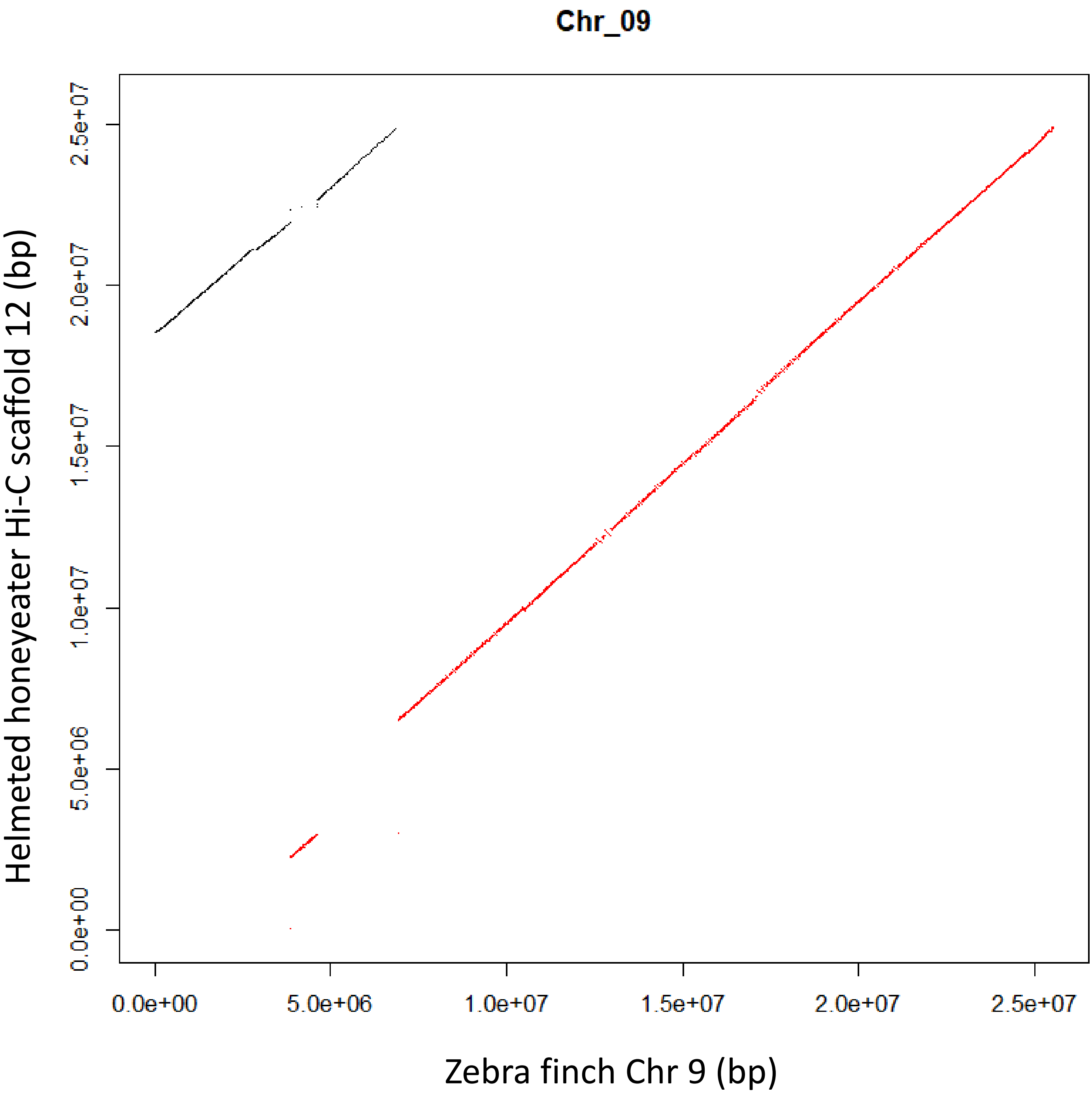
Chr_09
Helmeted honeyeater Hi-C scaffold 12 (bp)
0.0e+00
5.0e+06
1.0e+07
1.5e+07
2.0e+07
2.5e+07
Zebra finch Chr 9 (bp)

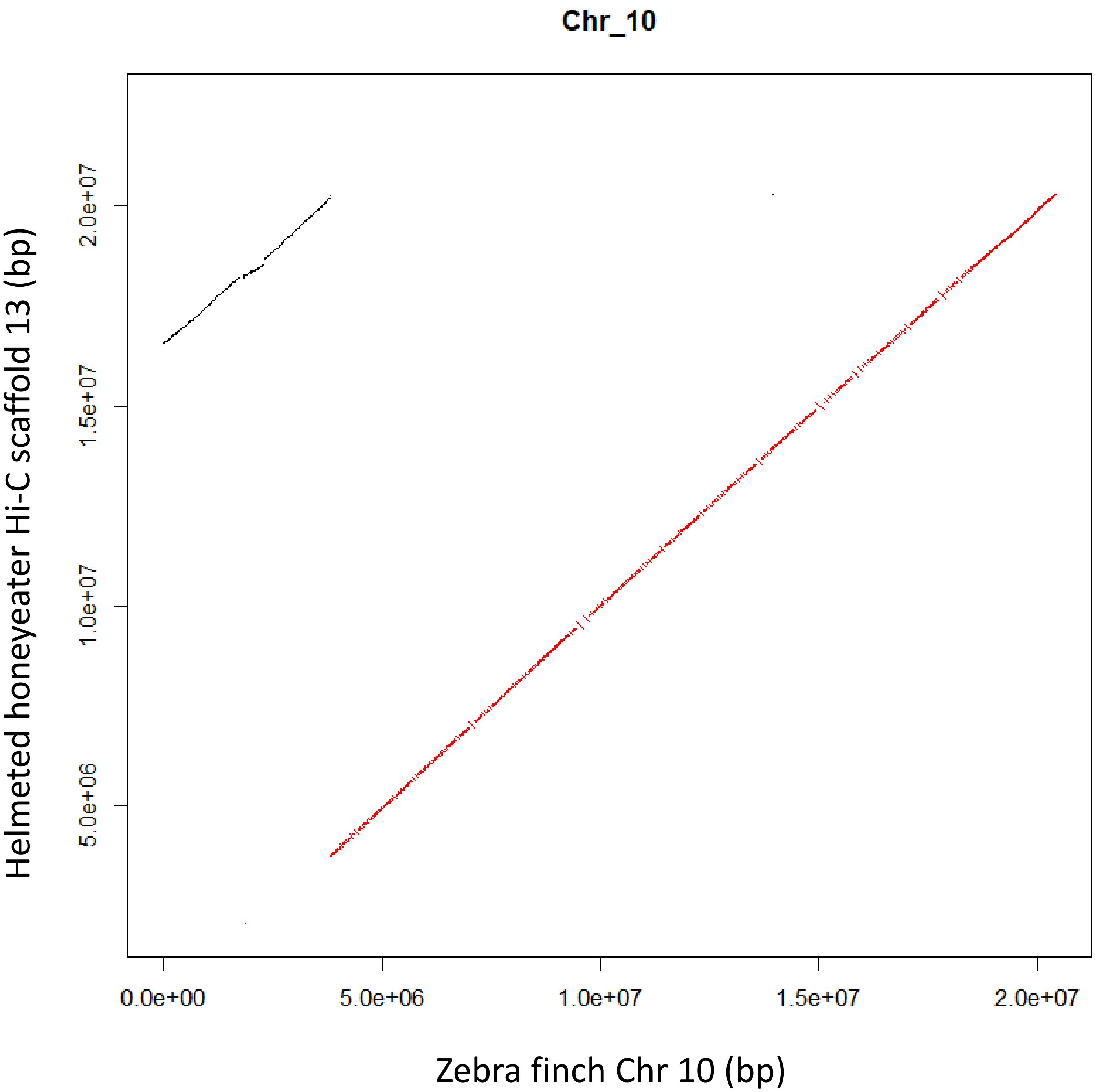

Chr_10
Helmeted honeyeater Hi-C scaffold 13 (bp)
2.0e+07
1.5e+07
1.0e+07
5.0e+06
0.0e+00
5.0e+06
1.0e+07
1.5e+07
2.0e+07
Zebra finch Chr 10 (bp)

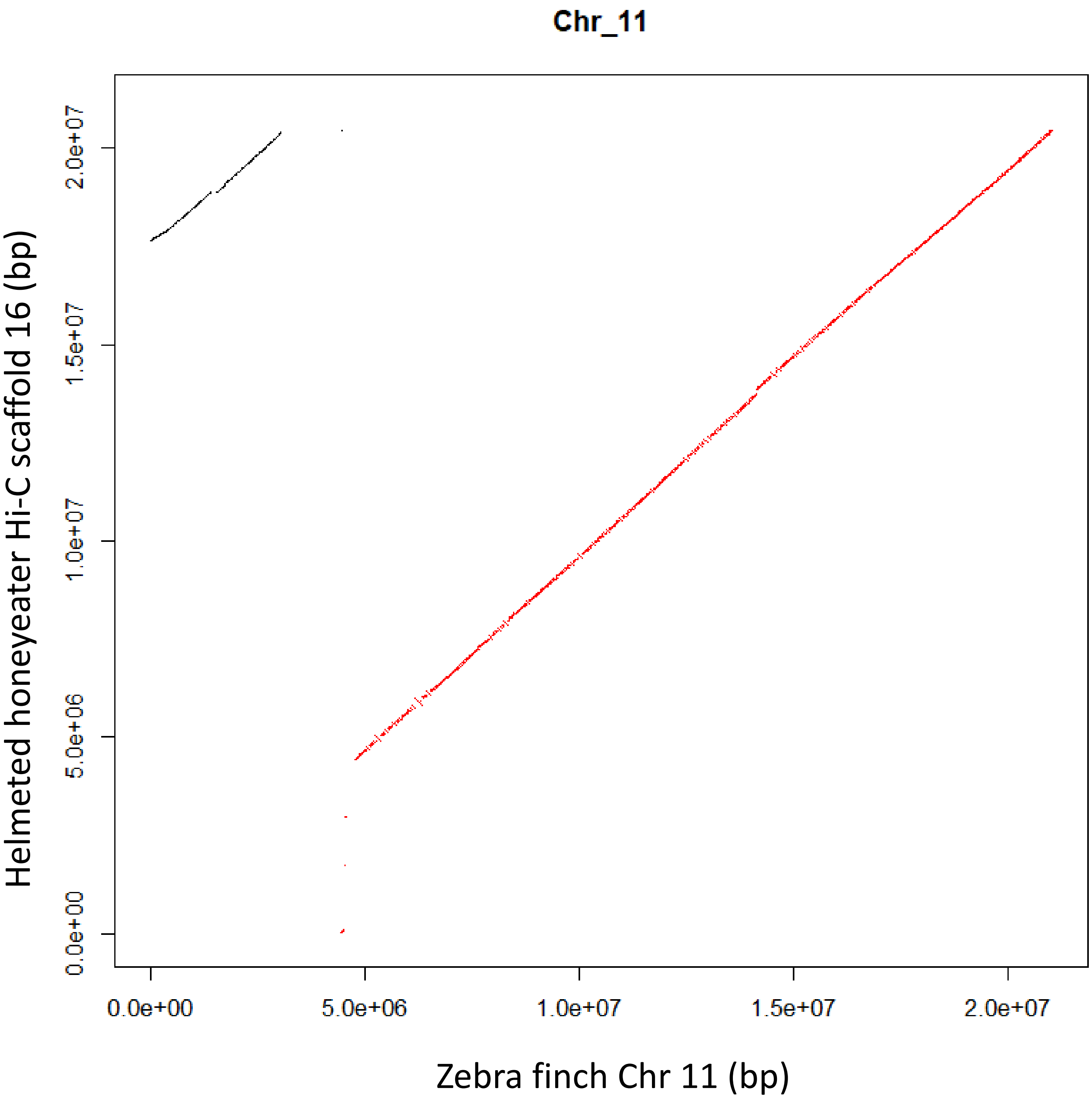
Chr_11
Helmeted honeyeater Hi-C scaffold 16 (bp)
0.0e+00
5.0e+06
1.0e+07
1.5e+07
2.0e+07
0.0e+00
5.0e+06
1.0e+07
1.5e+07
2.0e+07
Zebra finch Chr 11 (bp)

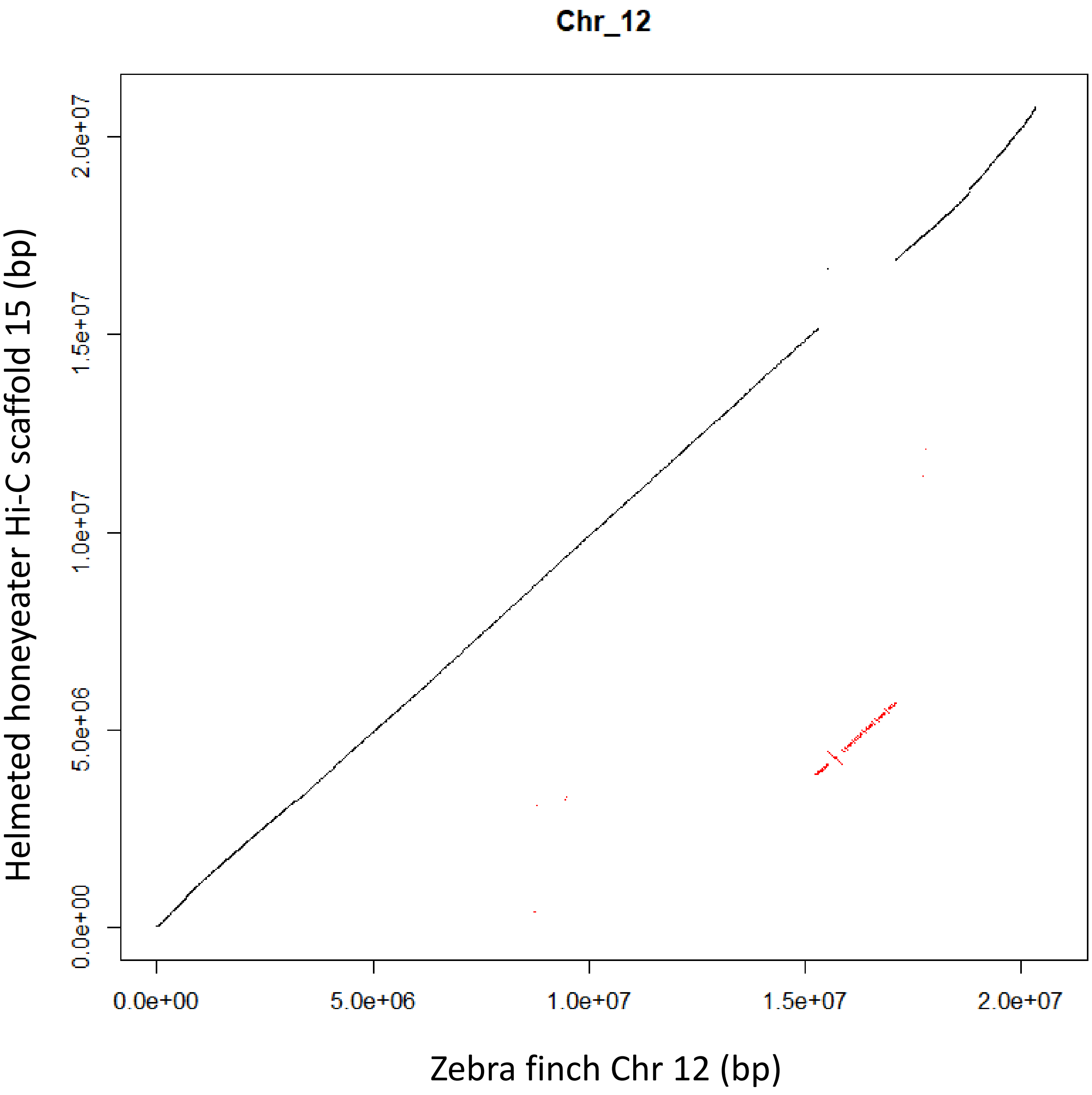

Chr_12
Helmeted honeyeater Hi-C scaffold 15 (bp)
0.0e+00
5.0e+06
1.0e+07
1.5e+07
2.0e+07
0.0e+00
5.0e+06
1.0e+07
1.5e+07
2.0e+07
Zebra finch Chr 12 (bp)

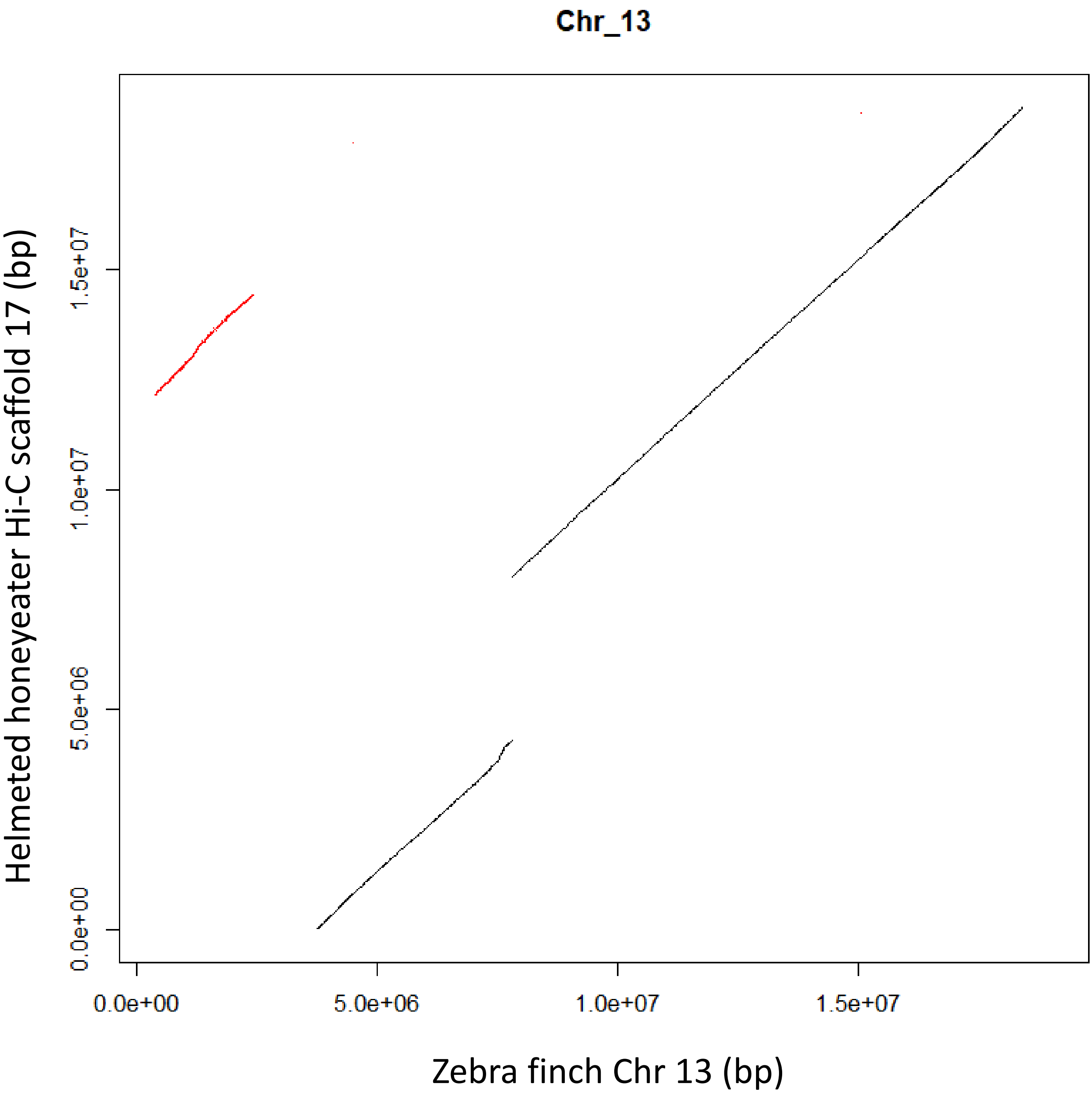
Chr_13
Helmeted honeyeater Hi-C scaffold 17 (bp)
0.0e+00
5.0e+06
1.0e+07
1.5e+07
0.0e+00
5.0e+06
1.0e+07
1.5e+07
Zebra finch Chr 13 (bp)

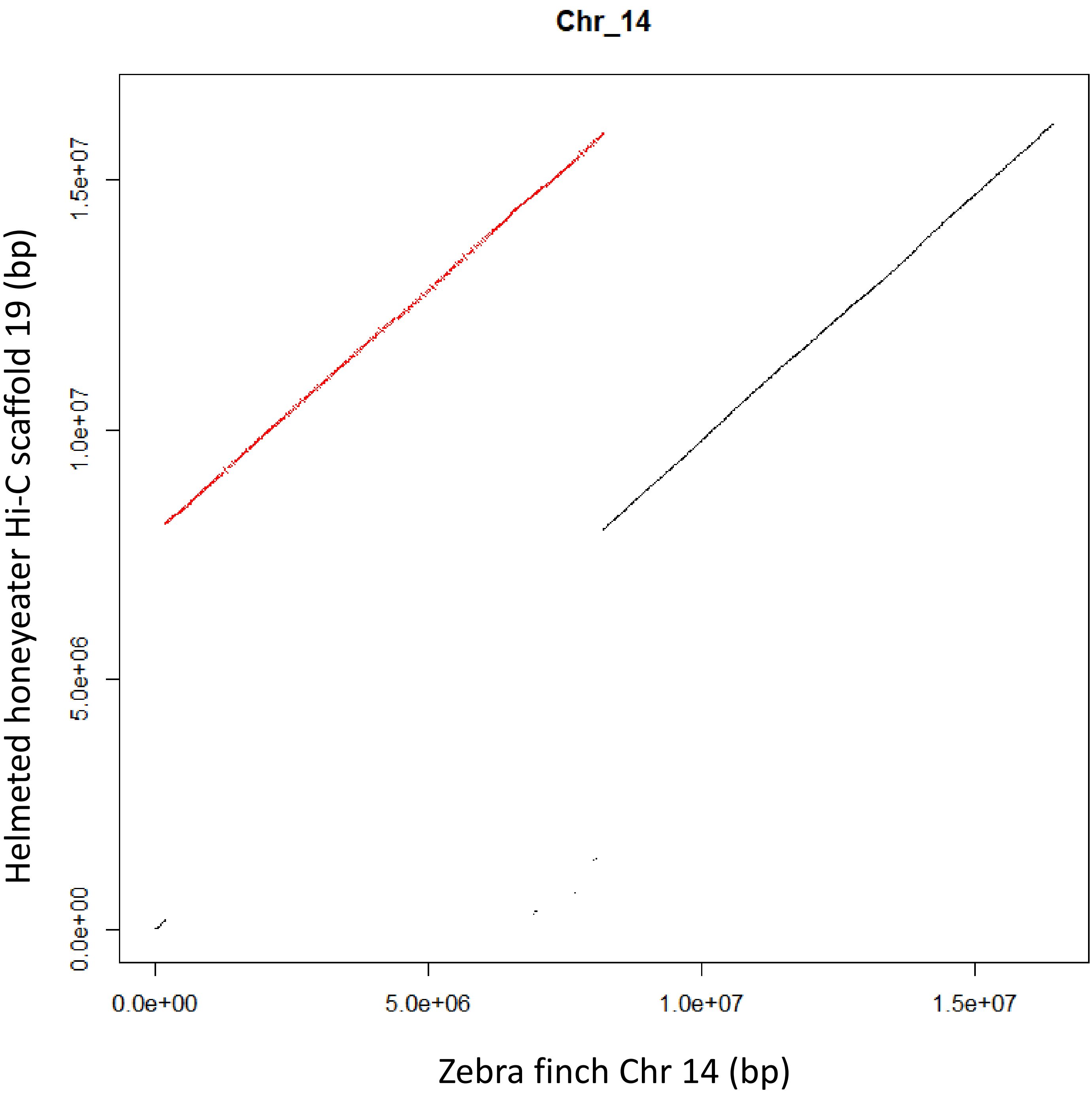
Chr_14
Helmeted honeyeater Hi-C scaffold 19 (bp)
0.0e+00
5.0e+06
1.0e+07
1.5e+07
0.0e+00
5.0e+06
1.0e+07
1.5e+07
Zebra finch Chr 14 (bp)

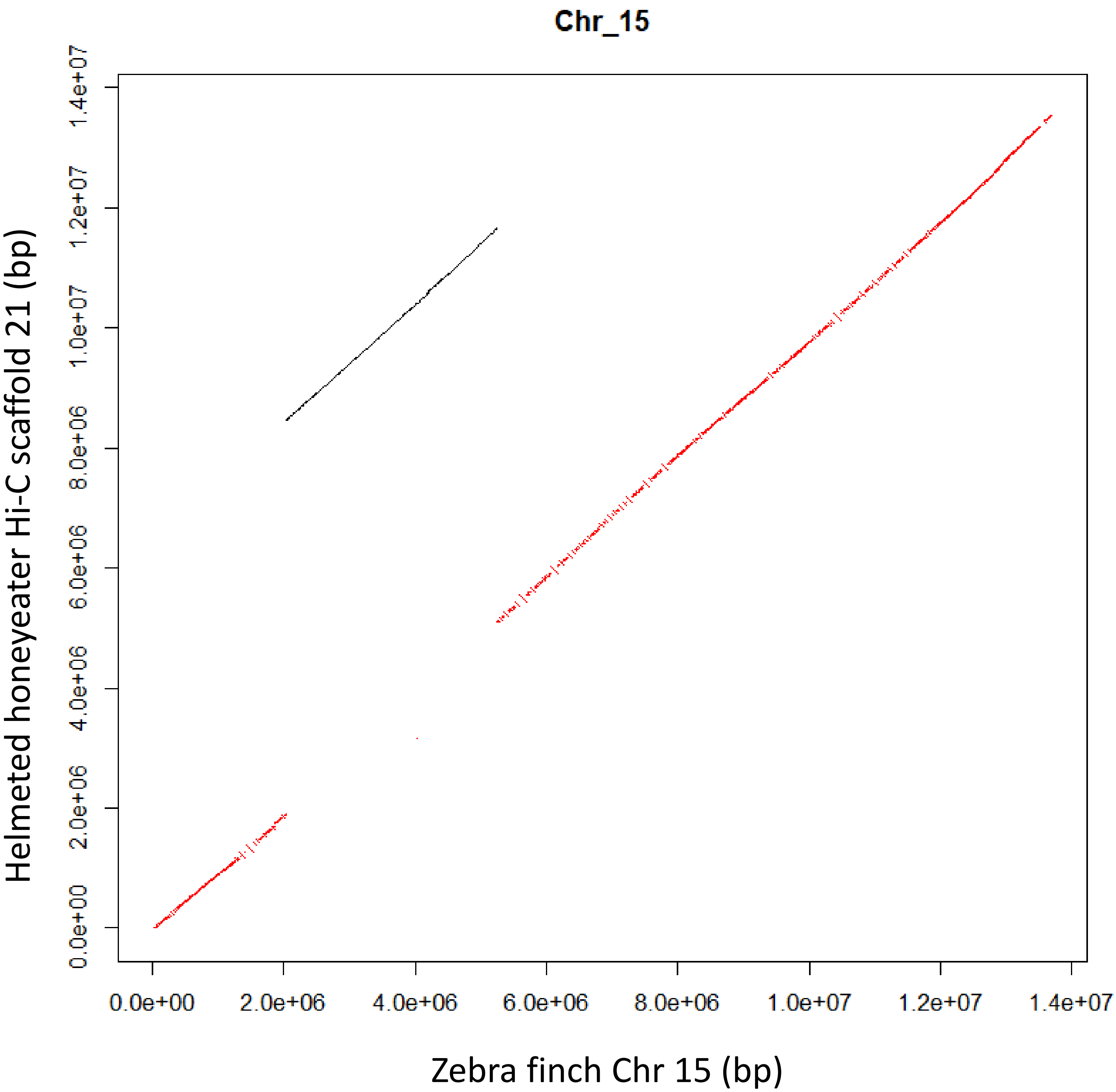

Chr_15
Helmeted honeyeater Hi-C scaffold 21 (bp)
0.0e+00
2.0e+06
4.0e+06
6.0e+06
8.0e+06
1.0e+07
1.2e+07
1.4e+07
Zebra finch Chr 15 (bp)

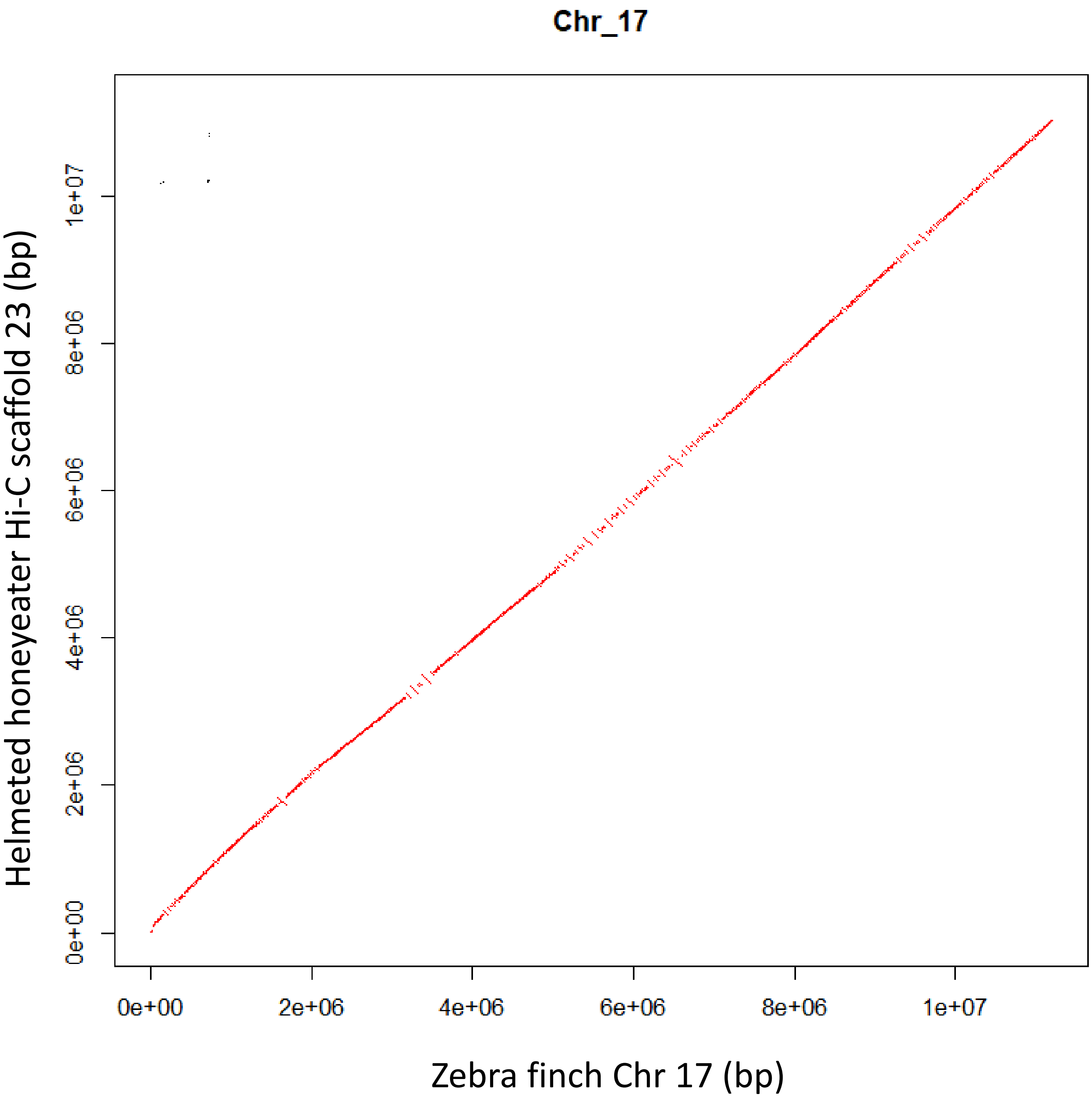

Chr_17
Helmeted honeyeater Hi-C scaffold 23 (bp)
0e+00
2e+06
4e+06
6e+06
8e+06
1e+07
0e+00
2e+06
4e+06
6e+06
8e+06
1e+07
Zebra finch Chr 17 (bp)

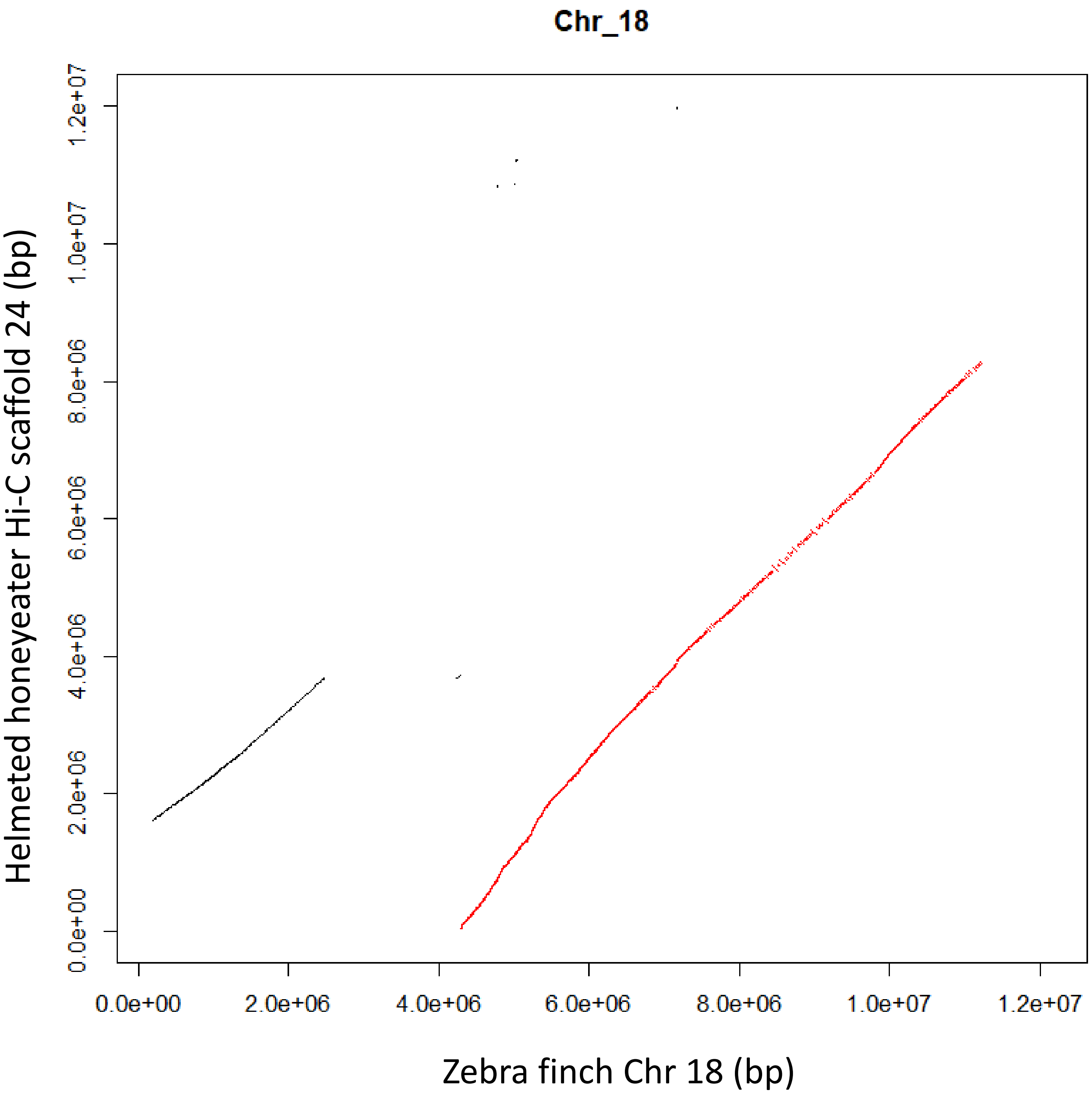

Chr_18
Helmeted honeyeater Hi-C scaffold 24 (bp)
0.0e+00
2.0e+06
4.0e+06
6.0e+06
8.0e+06
1.0e+07
1.2e+07
Zebra finch Chr 18 (bp)

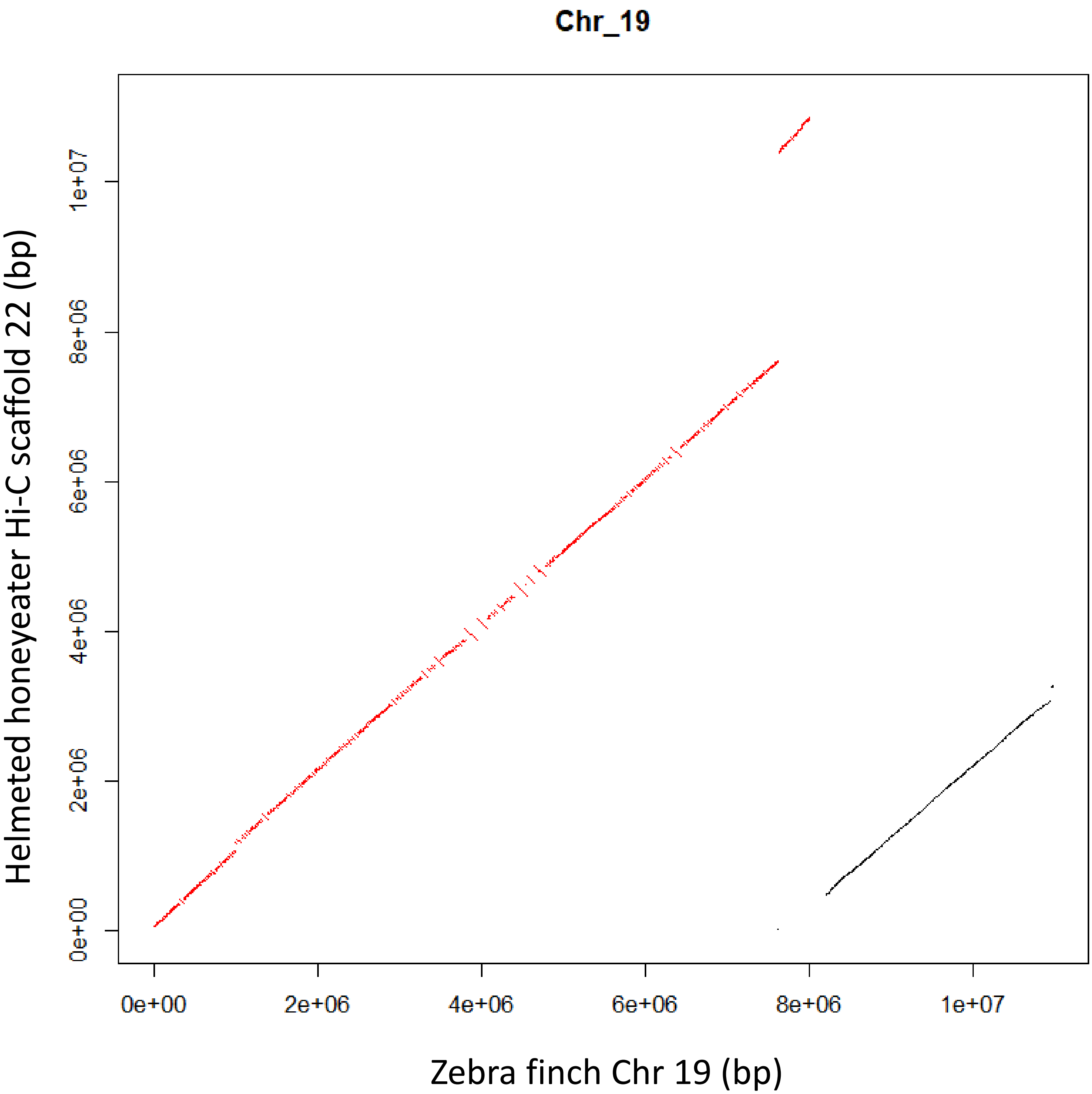
Chr_19
Helmeted honeyeater Hi-C scaffold 22 (bp)
0e+00
2e+06
4e+06
6e+06
8e+06
1e+07
Zebra finch Chr 19 (bp)

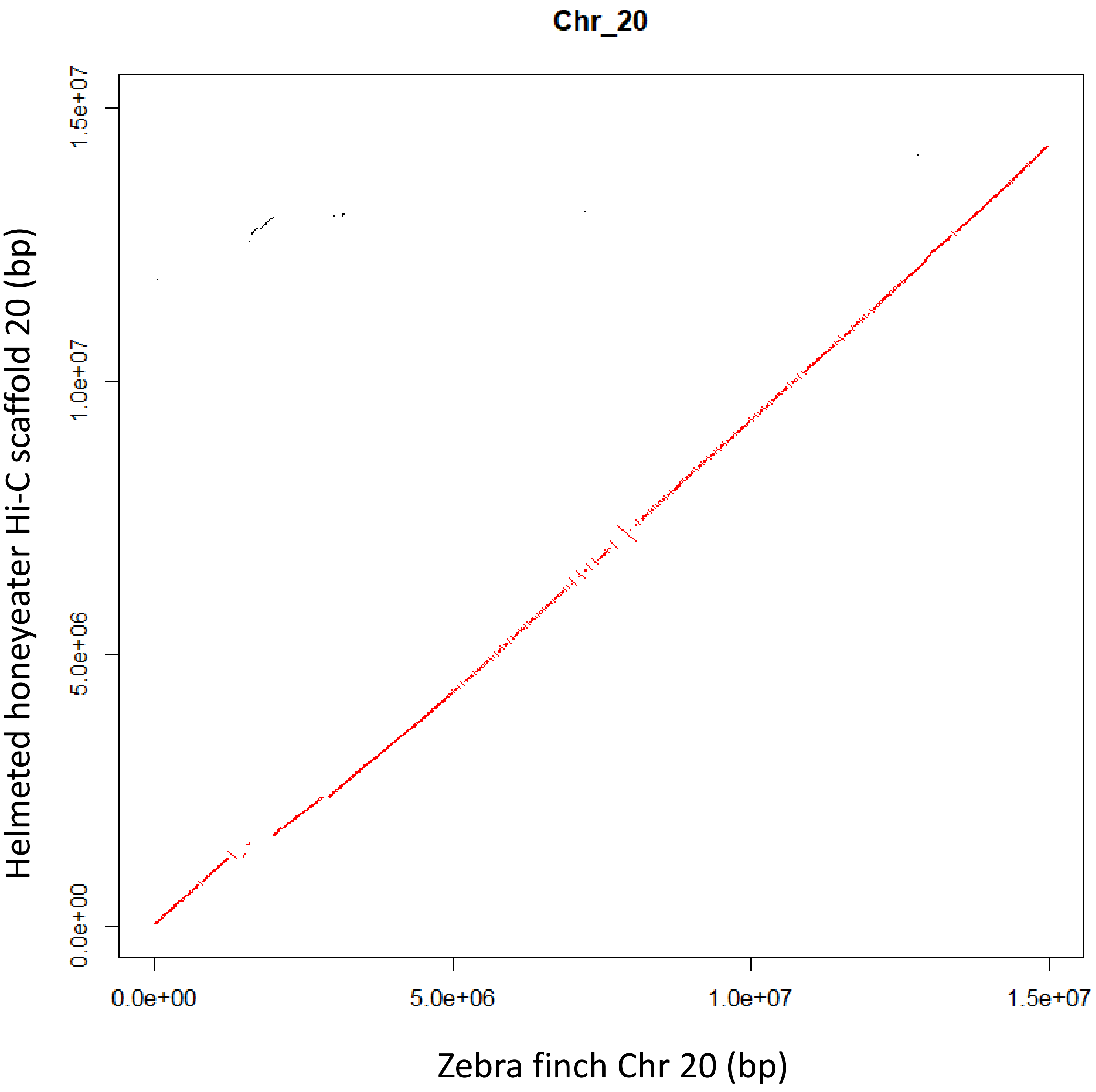

Chr_20
1.5e+07
1.0e+07
5.0e+06
0.0e+00
Helmeted honeyeater Hi-C scaffold 20 (bp)
0.0e+00
5.0e+06
1.0e+07
1.5e+07
Zebra finch Chr 20 (bp)

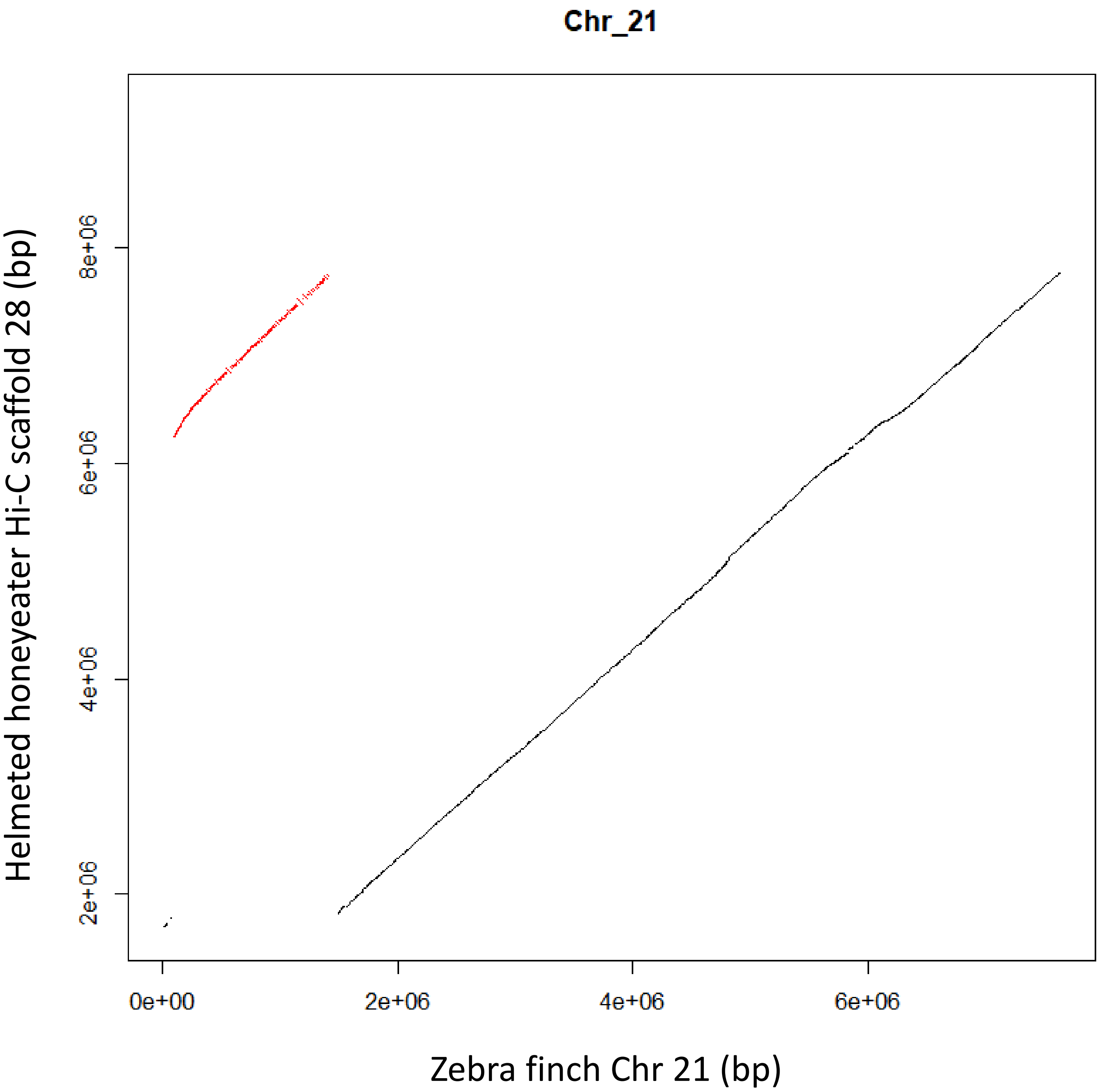

Chr_21
Helmeted honeyeater Hi-C scaffold 28 (bp)
2e+06
4e+06
6e+06
8e+06
0e+00
2e+06
4e+06
6e+06
Zebra finch Chr 21 (bp)

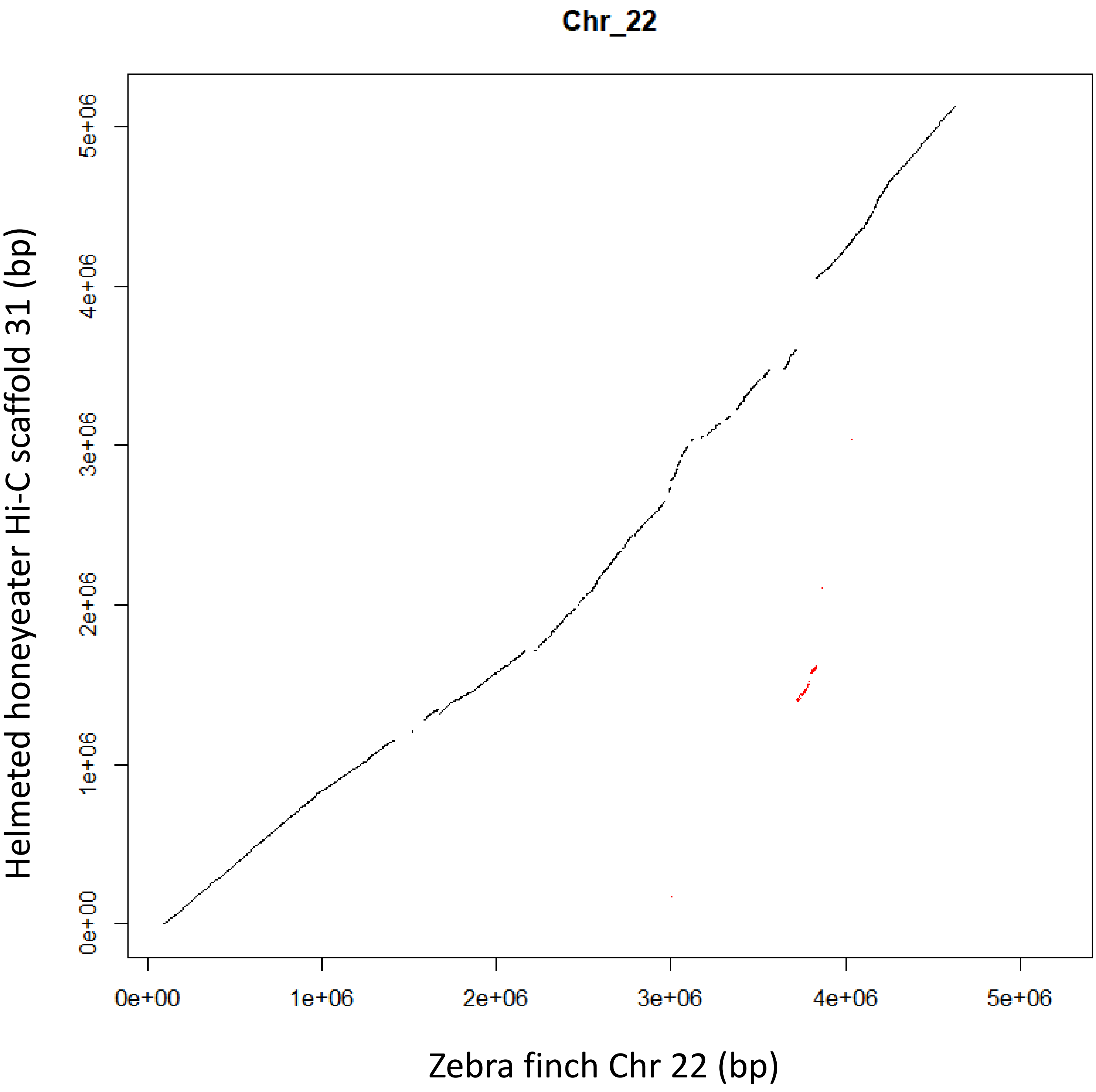
Chr_22
Helmeted honeyeater Hi-C scaffold 31 (bp)
0e+00
1e+06
2e+06
3e+06
4e+06
5e+06
0e+00
1e+06
2e+06
3e+06
4e+06
5e+06
Zebra finch Chr 22 (bp)

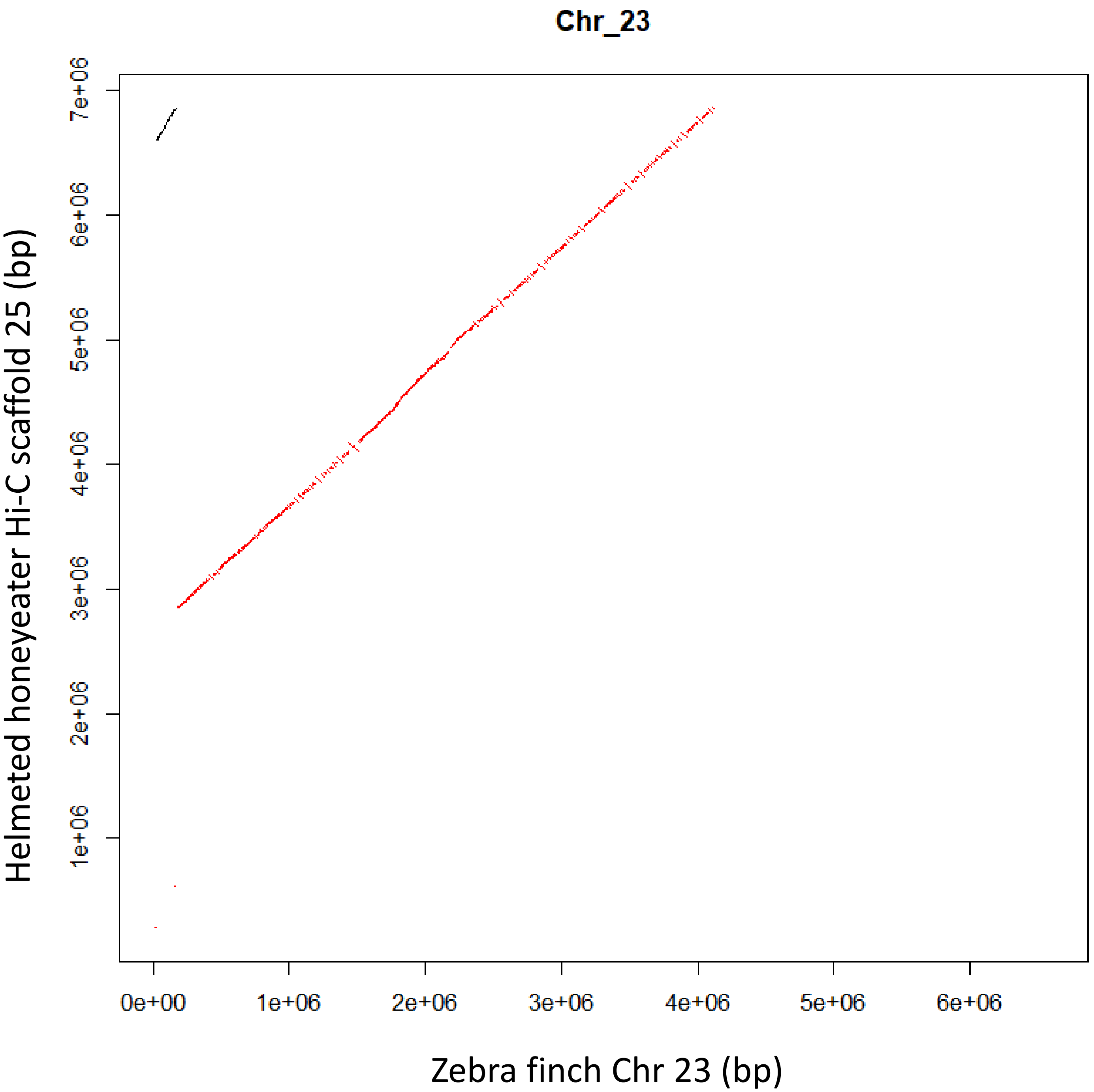

Chr_23
Helmeted honeyeater Hi-C scaffold 25 (bp)
1e+06
2e+06
3e+06
4e+06
5e+06
6e+06
7e+06
0e+00
1e+06
2e+06
3e+06
4e+06
5e+06
6e+06
Zebra finch Chr 23 (bp)

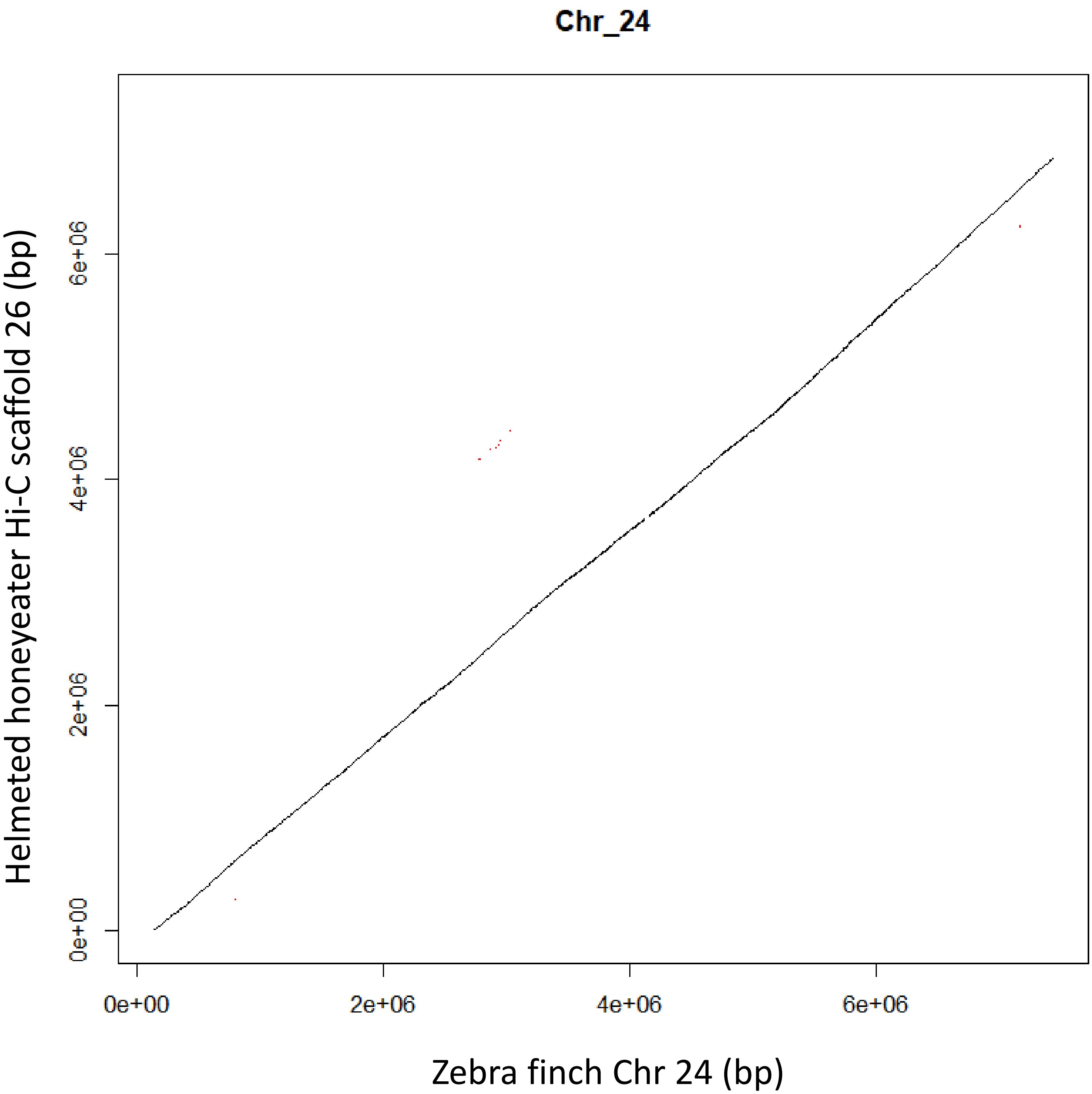

Chr_24
Helmeted honeyeater Hi-C scaffold 26 (bp)
0e+00
2e+06
4e+06
6e+06
0e+00
2e+06
4e+06
6e+06
Zebra finch Chr 24 (bp)

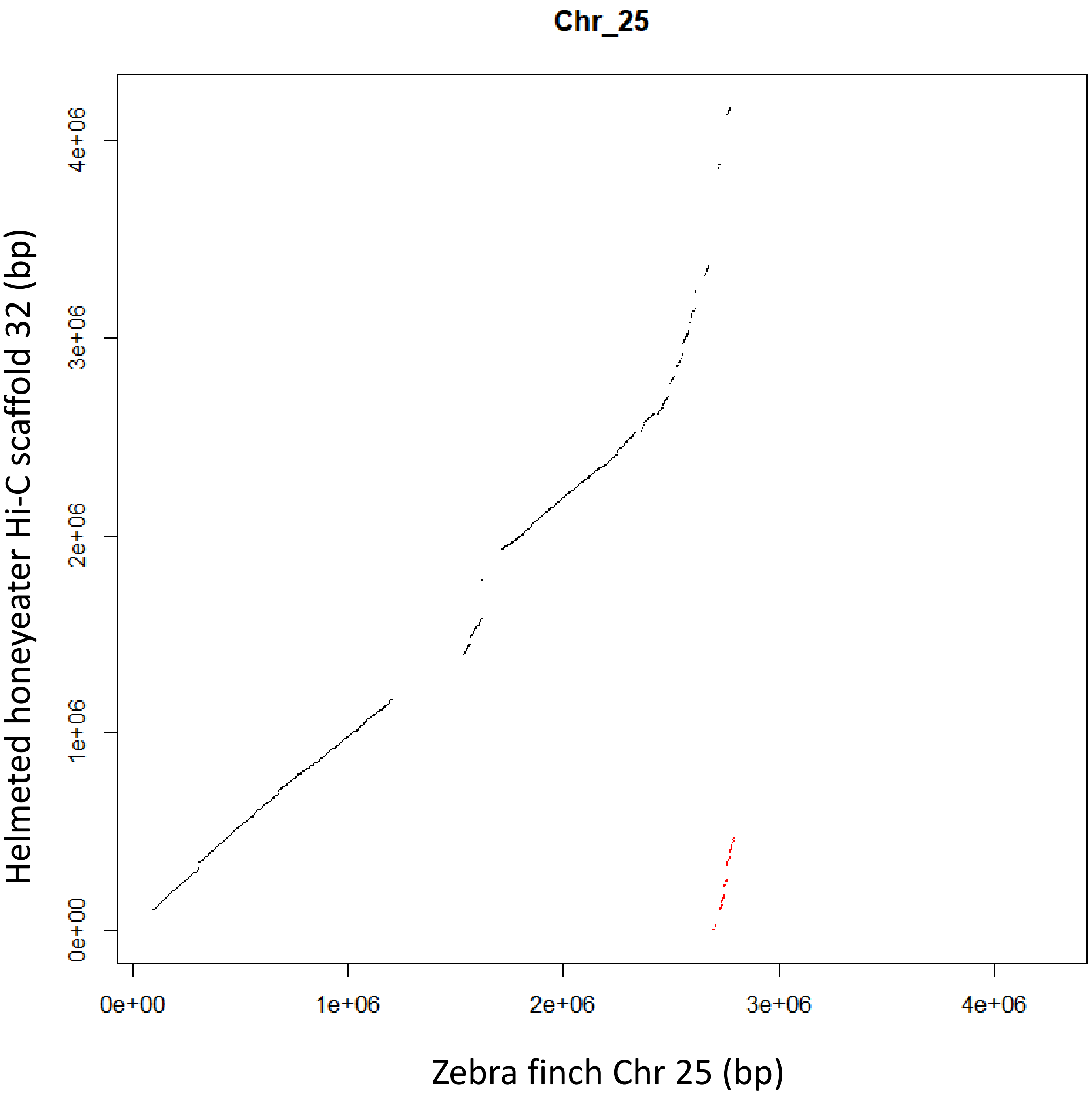

Chr_25
Helmeted honeyeater Hi-C scaffold 32 (bp)
0e+00
1e+06
2e+06
3e+06
4e+06
Zebra finch Chr 25 (bp)

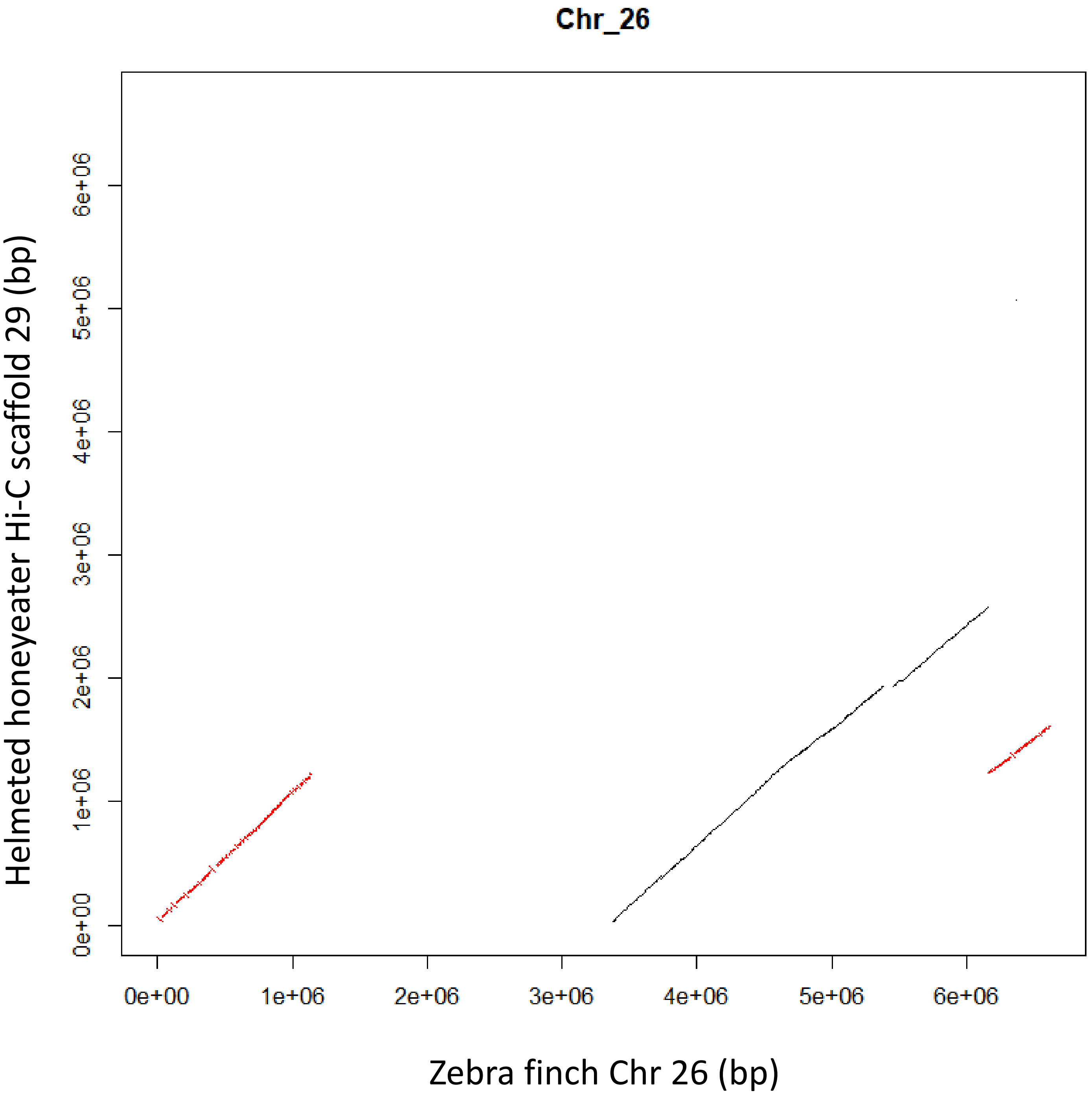
Chr_26
Helmeted honeyeater Hi-C scaffold 29 (bp)
0e+00
1e+06
2e+06
3e+06
4e+06
5e+06
6e+06
0e+00
1e+06
2e+06
3e+06
4e+06
5e+06
6e+06
Zebra finch Chr 26 (bp)

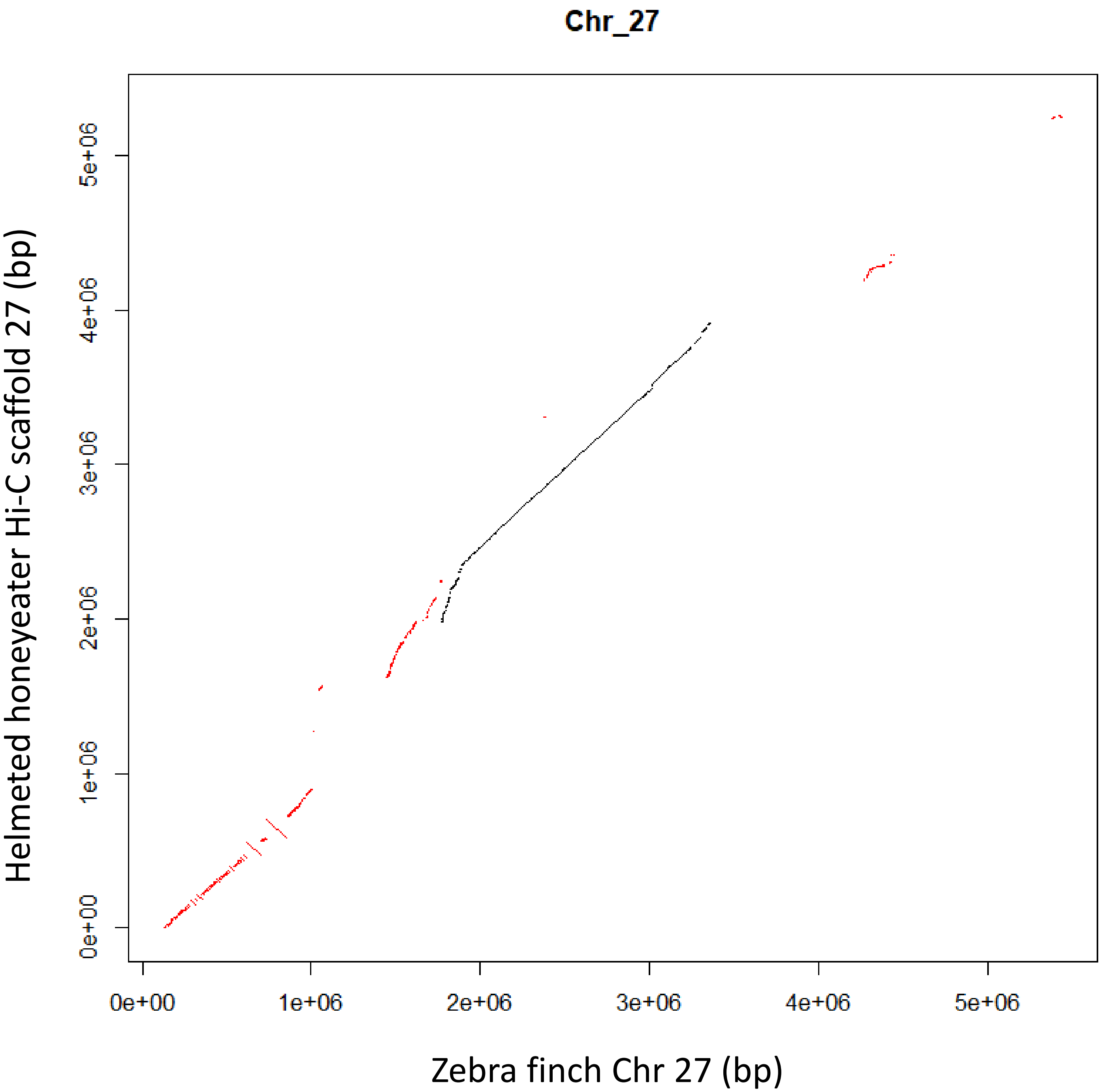
Chr_27
Helmeted honeyeater Hi-C scaffold 27 (bp)
0e+00
1e+06
2e+06
3e+06
4e+06
5e+06
Zebra finch Chr 27 (bp)
0e+00
1e+06
2e+06
3e+06
4e+06
5e+06

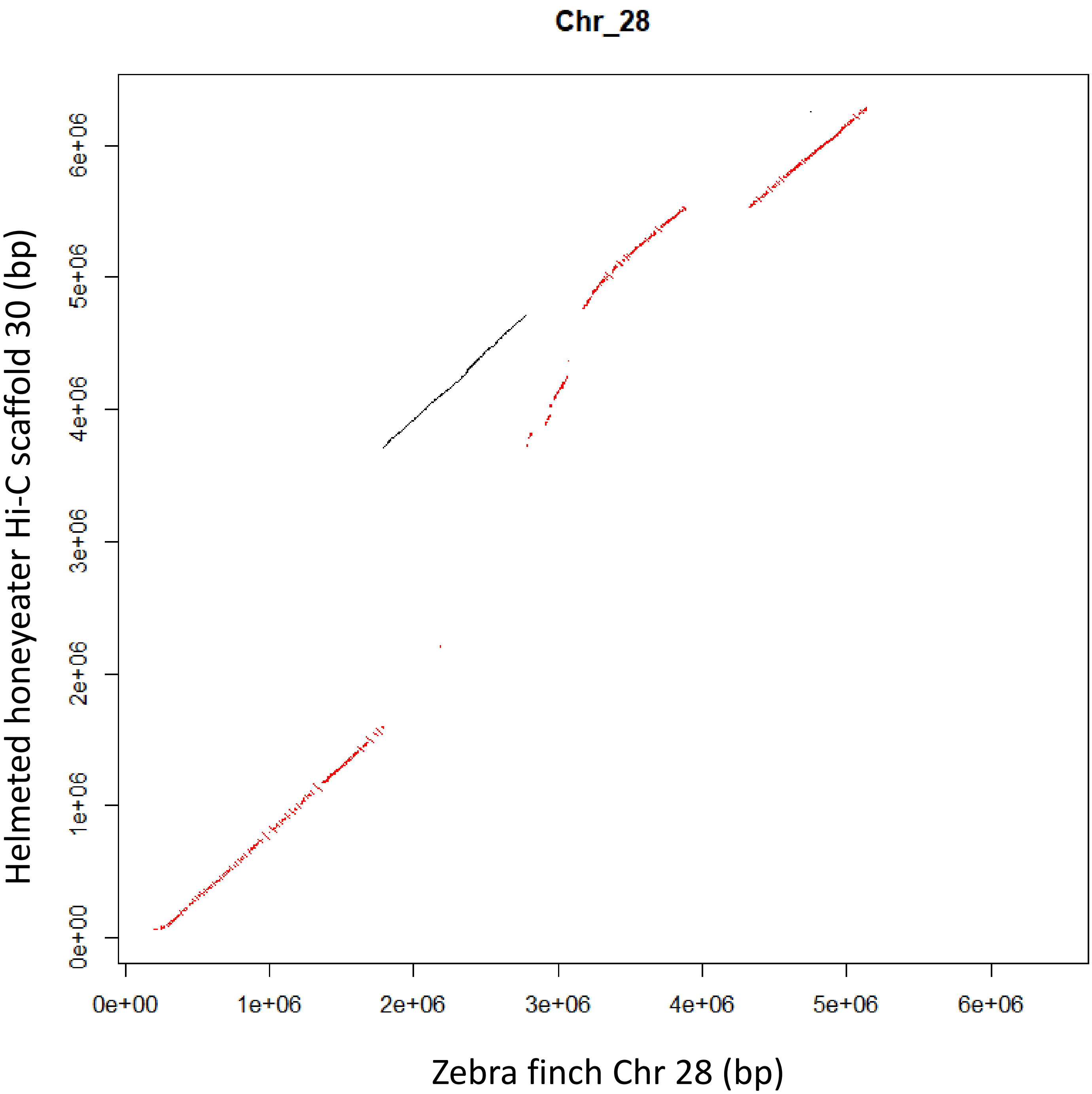
Chr_28
Helmeted honeyeater Hi-C scaffold 30 (bp)
0e+00
1e+06
2e+06
3e+06
4e+06
5e+06
6e+06
0e+00
1e+06
2e+06
3e+06
4e+06
5e+06
6e+06
Zebra finch Chr 28 (bp)

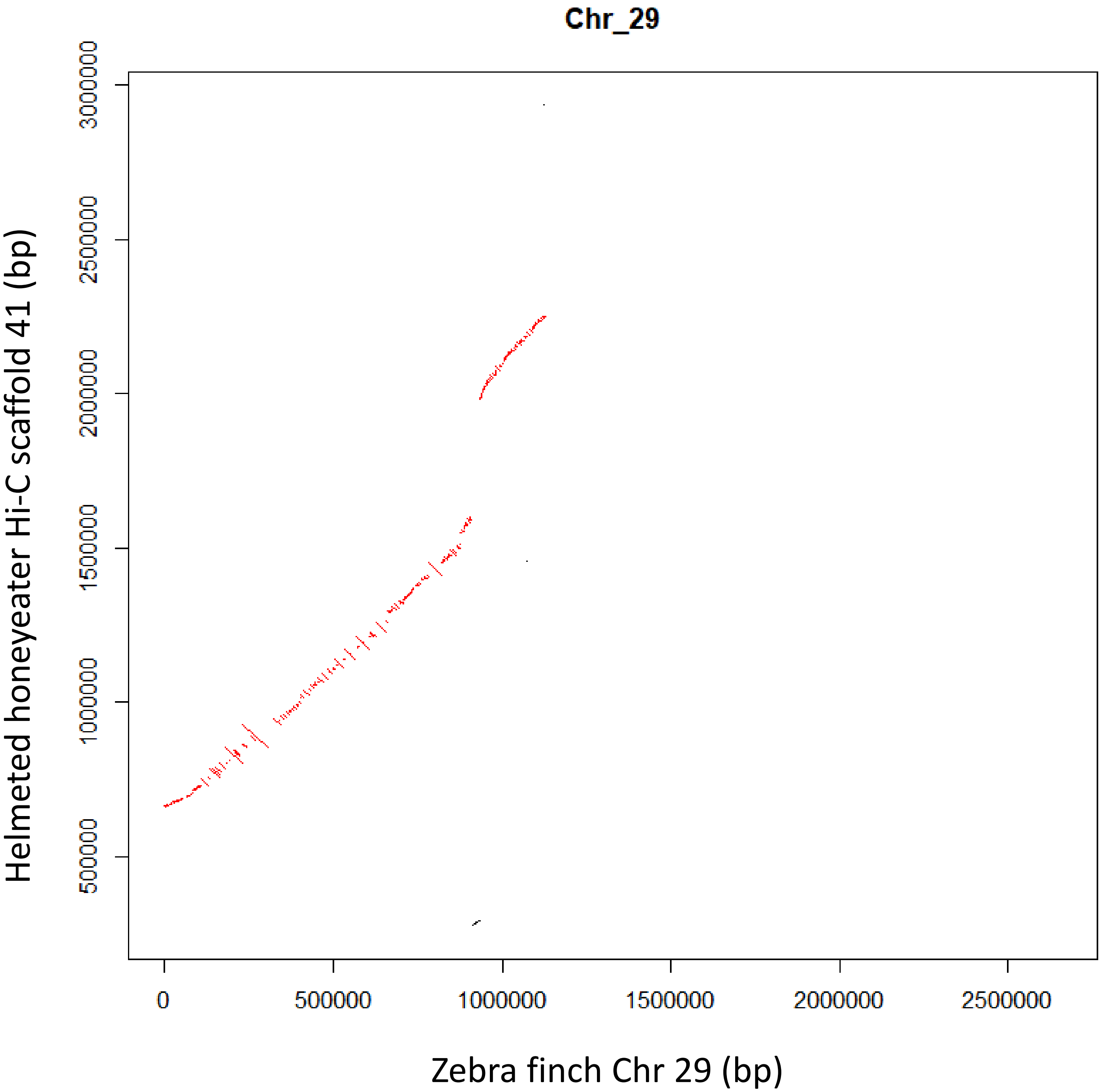

Chr_29
Helmeted honeyeater Hi-C scaffold 41 (bp)
3000000
2500000
2000000
1500000
1000000
500000
0
500000
1000000
1500000
2000000
2500000
Zebra finch Chr 29 (bp)
