# Supplementary figures and images for: Chromosome-length genome assembly and linkage map of a critically endangered Australian bird: the helmeted honeyeater
Source: Gigascience. 2022 Mar 29;11:giac025. doi: 10.1093/gigascience/giac025 (PMC8963300; doi:10.1093/gigascience/giac025)

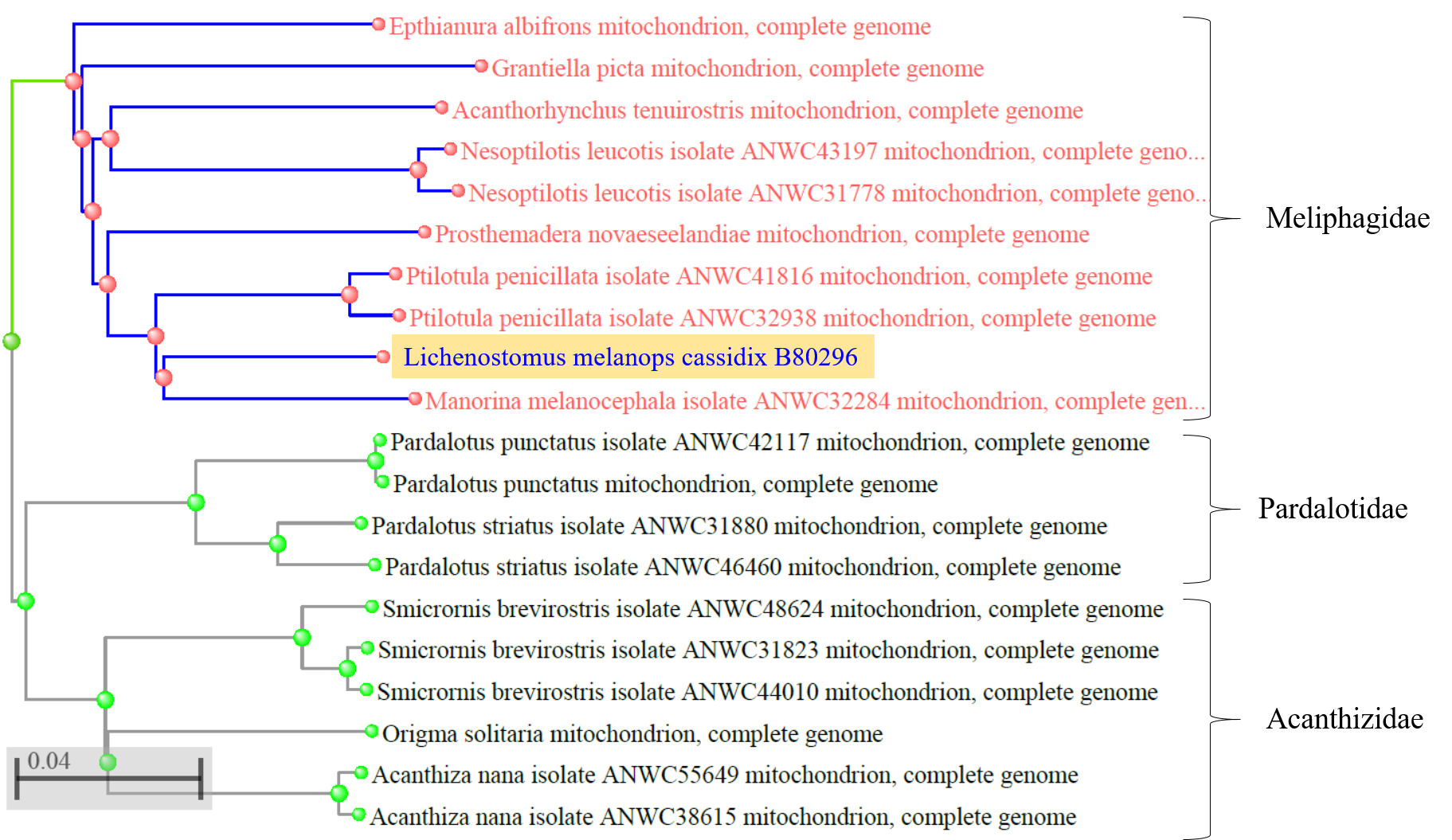

Supplement: giac025_Supplemental_Files [file giac025_supplemental_files.zip › Supplementary_Material_S1.png]

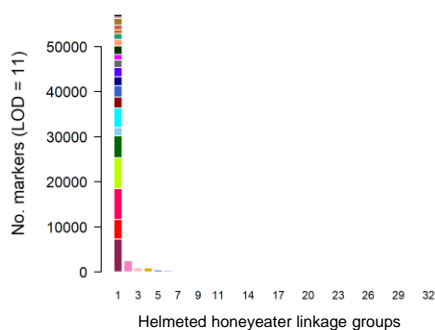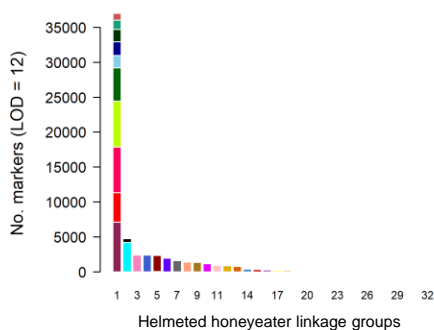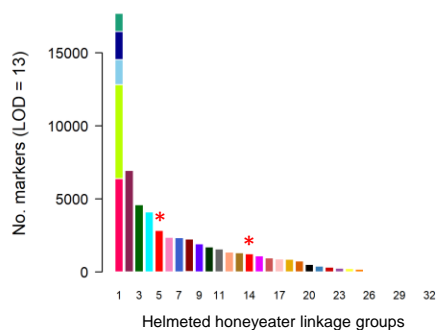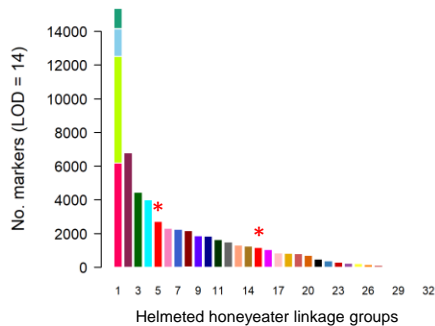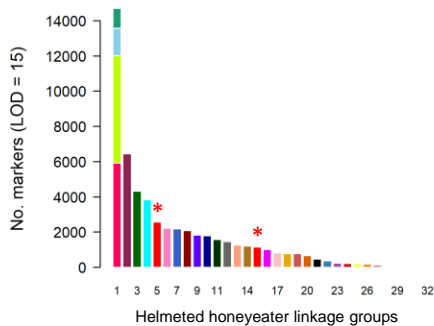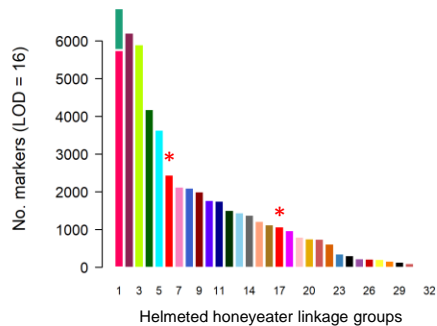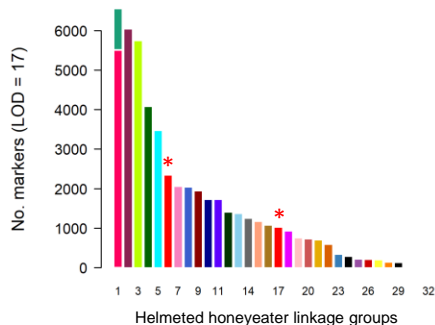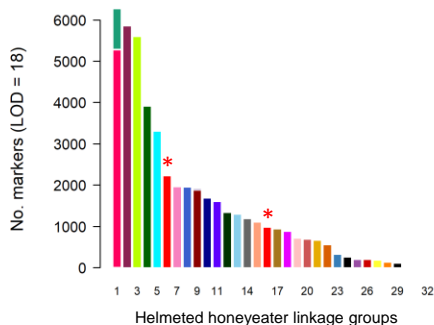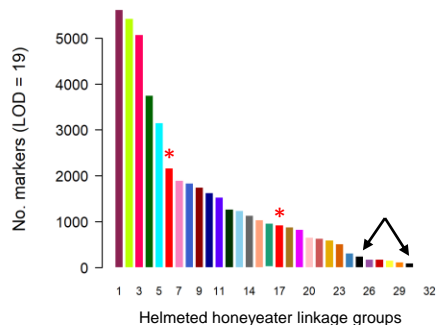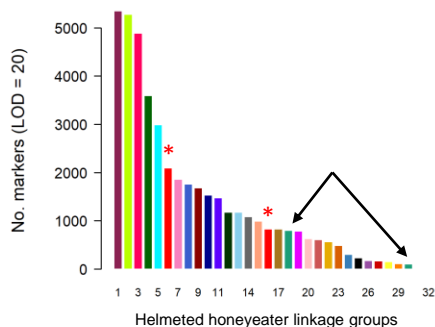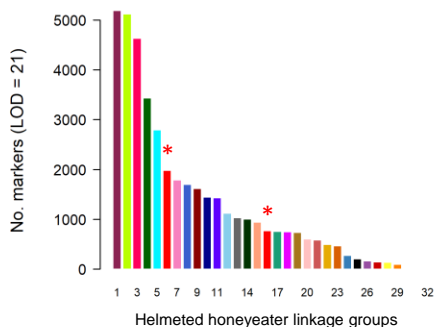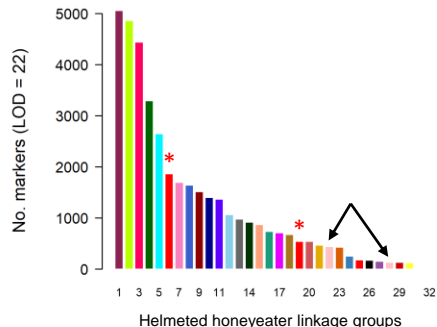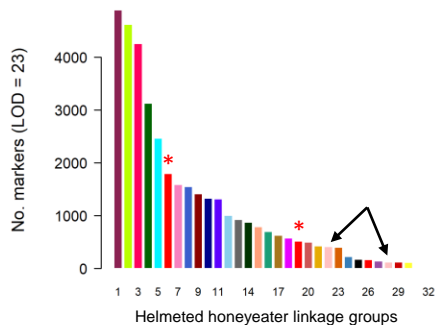

### Zebra finch chromosomes

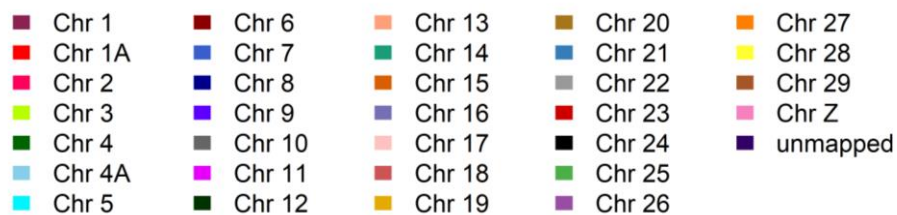

Supplement: giac025_Supplemental_Files [file giac025_supplemental_files.zip › Supplementary_Material_S10.pdf]

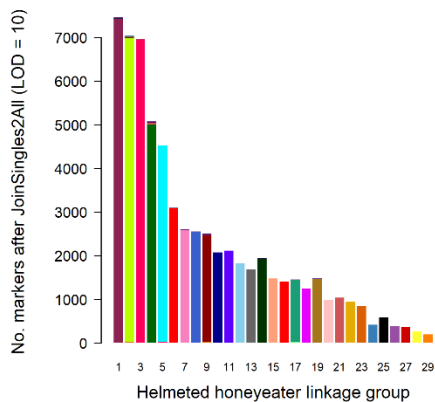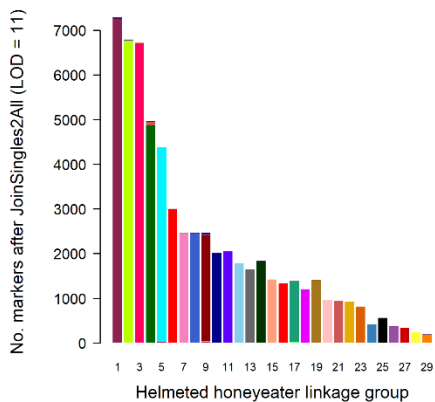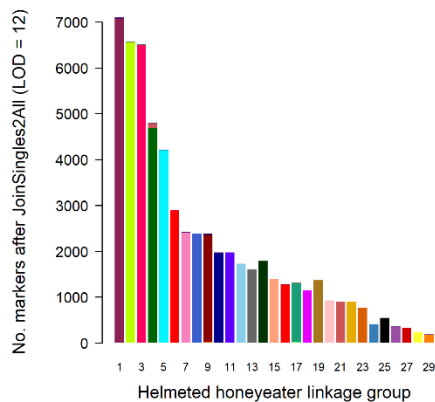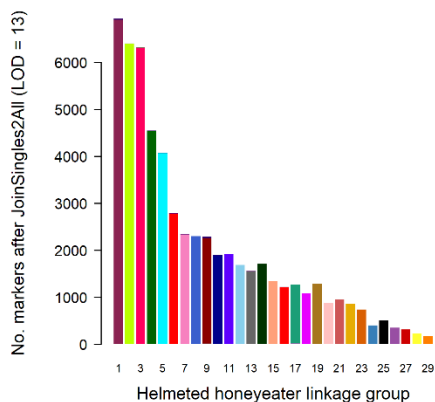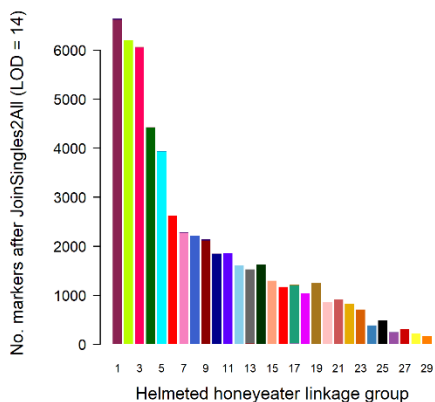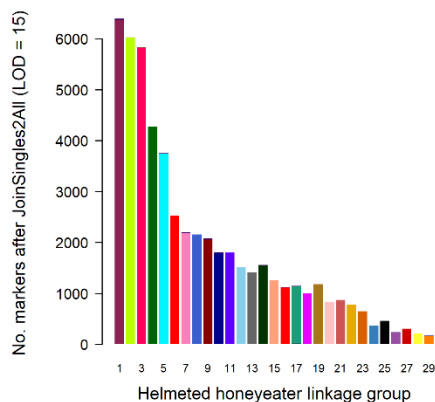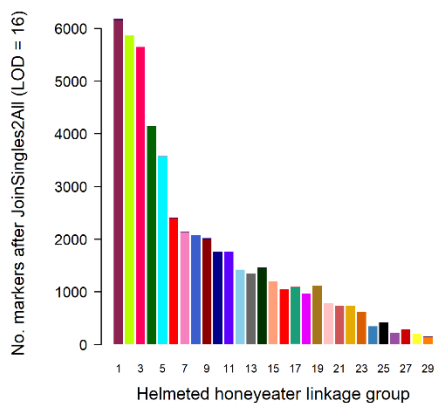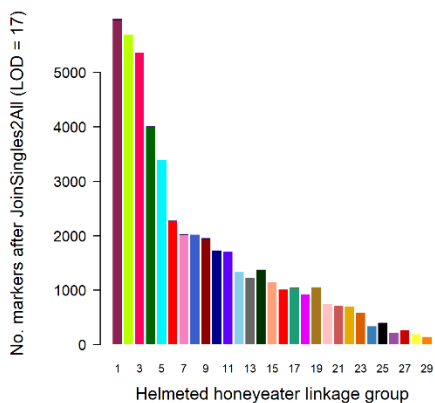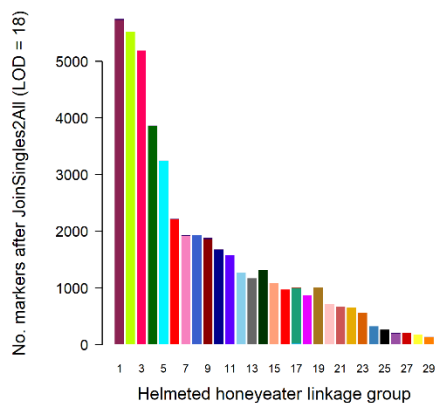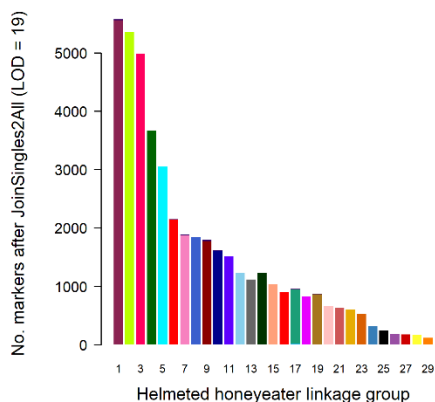

## Zebra finch chromosomes

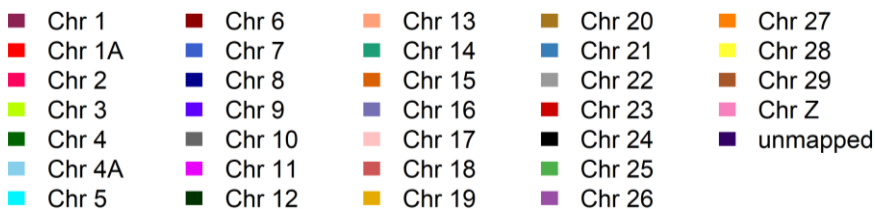

Supplement: giac025_Supplemental_Files [file giac025_supplemental_files.zip › Supplementary_Material_S11.pdf]

A

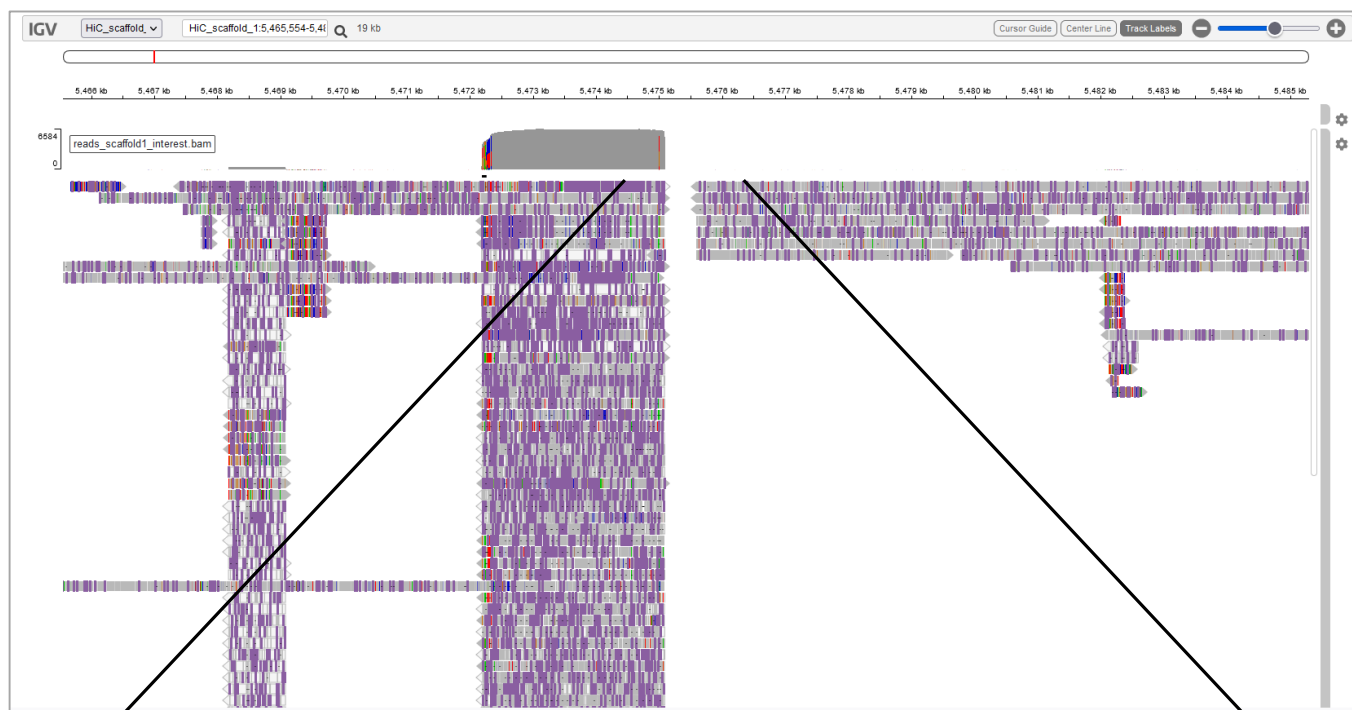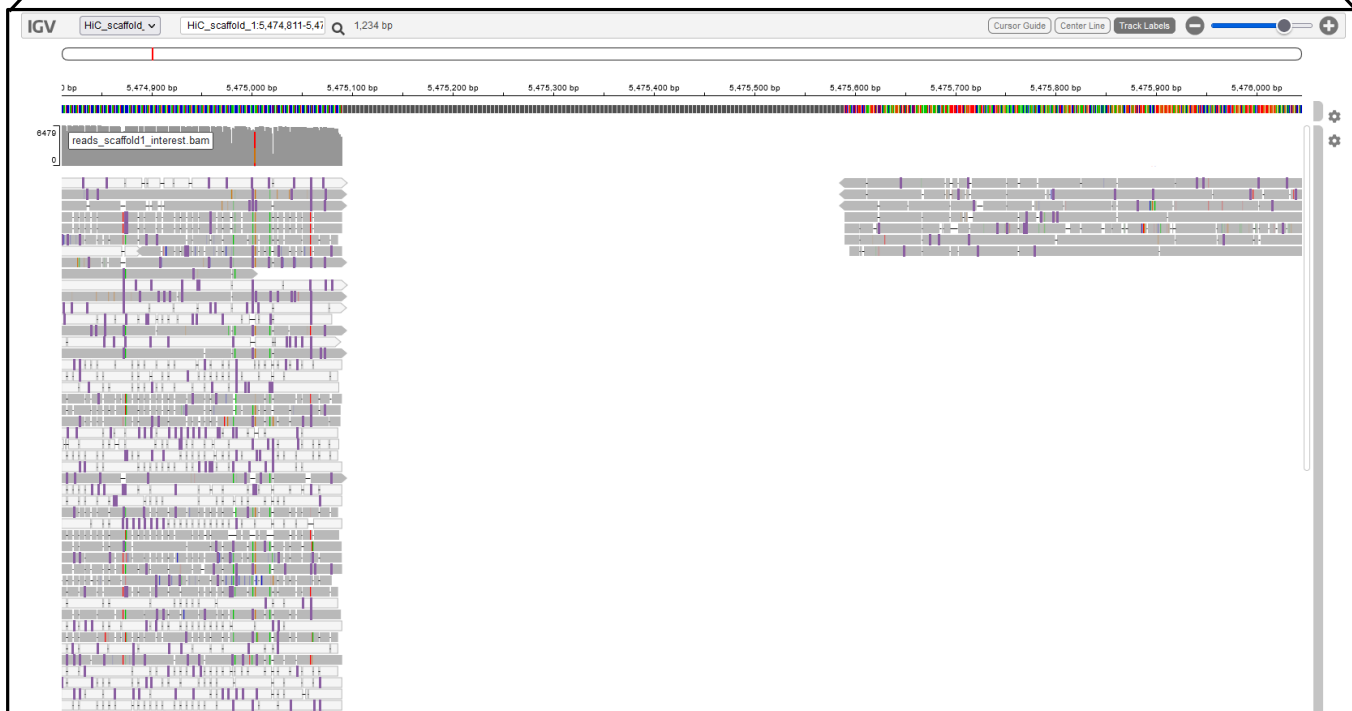

B

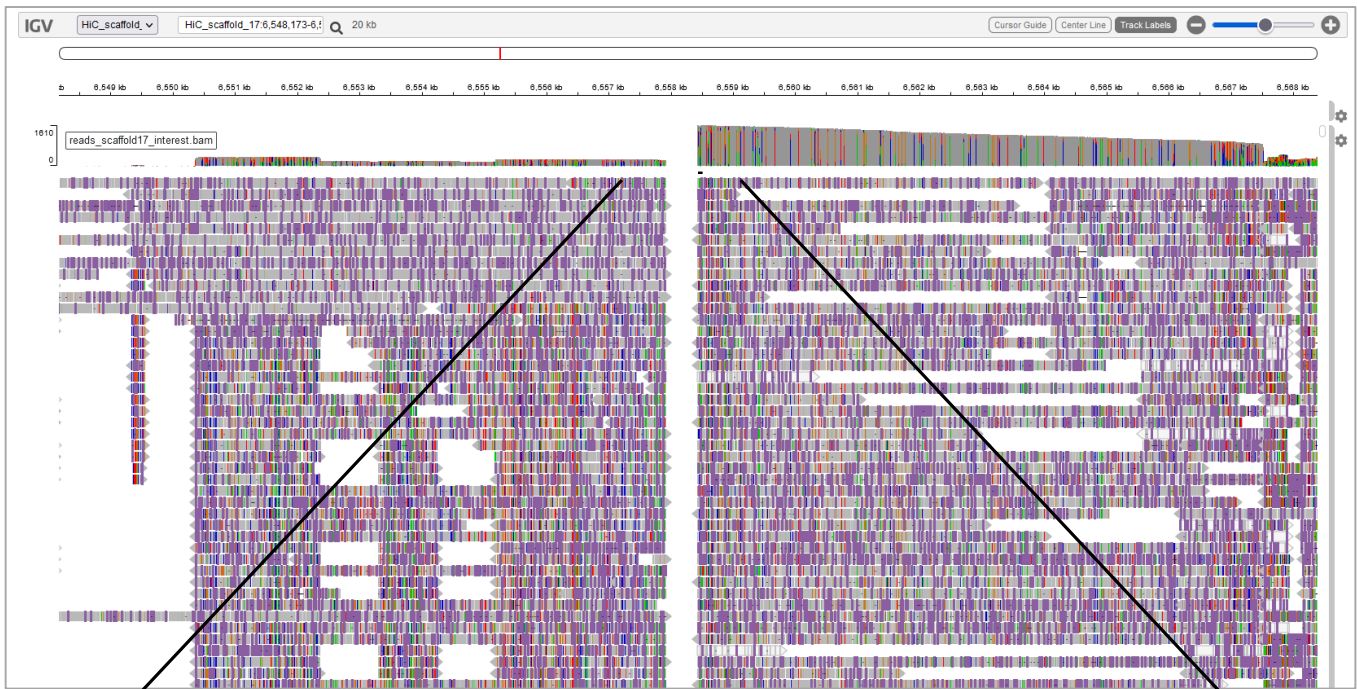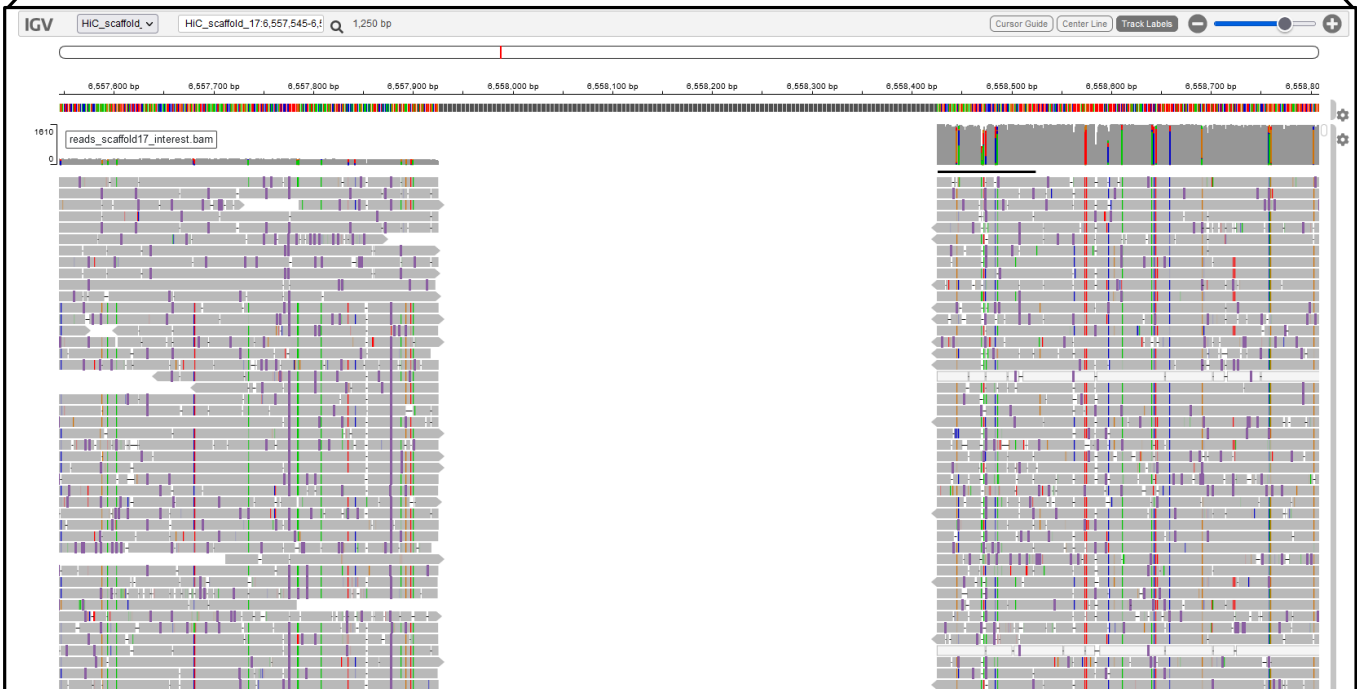

C

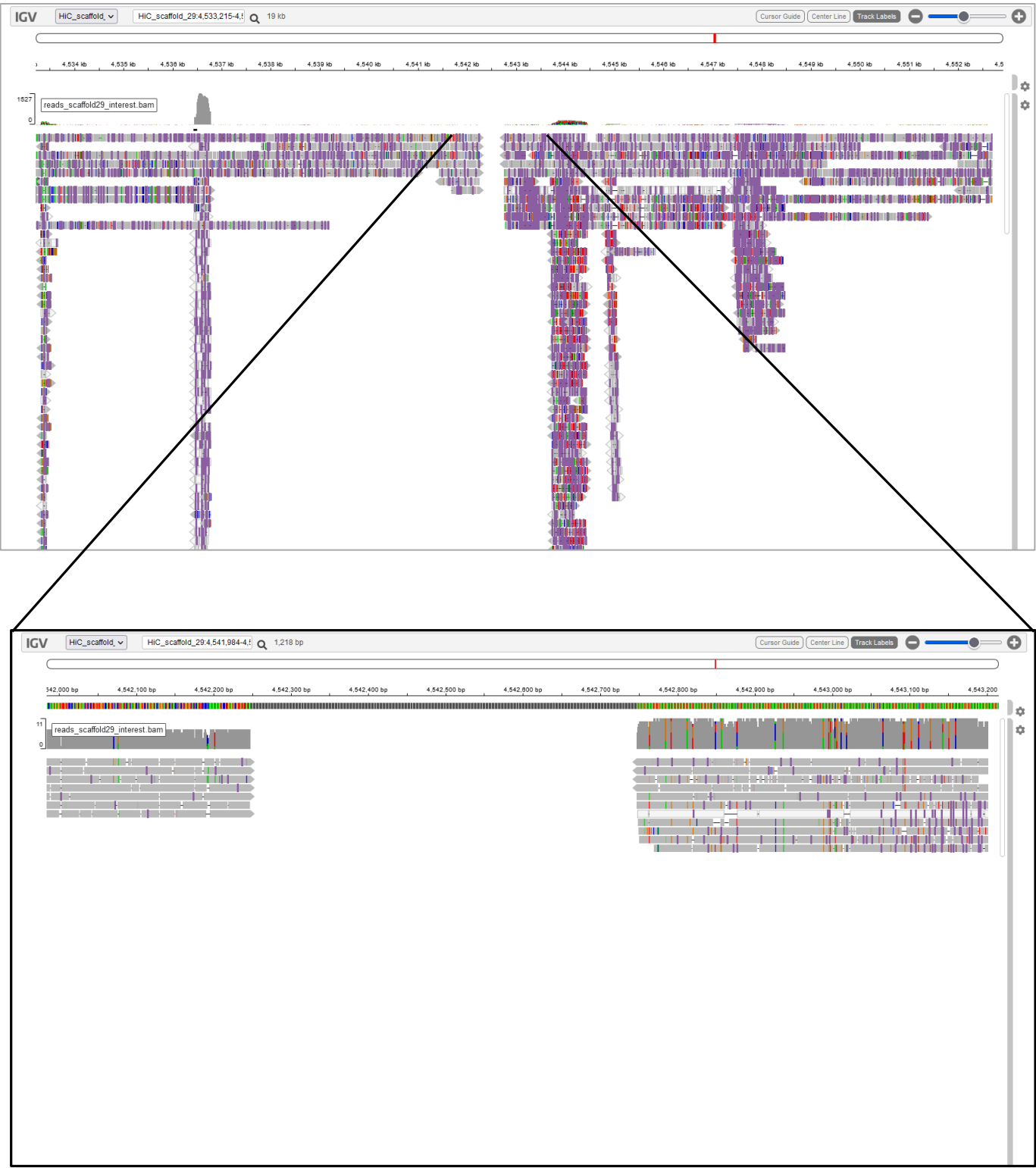

Supplement: giac025_Supplemental_Files [file giac025_supplemental_files.zip › Supplementary_Material_S13.pdf]

**Chr 26 Female**

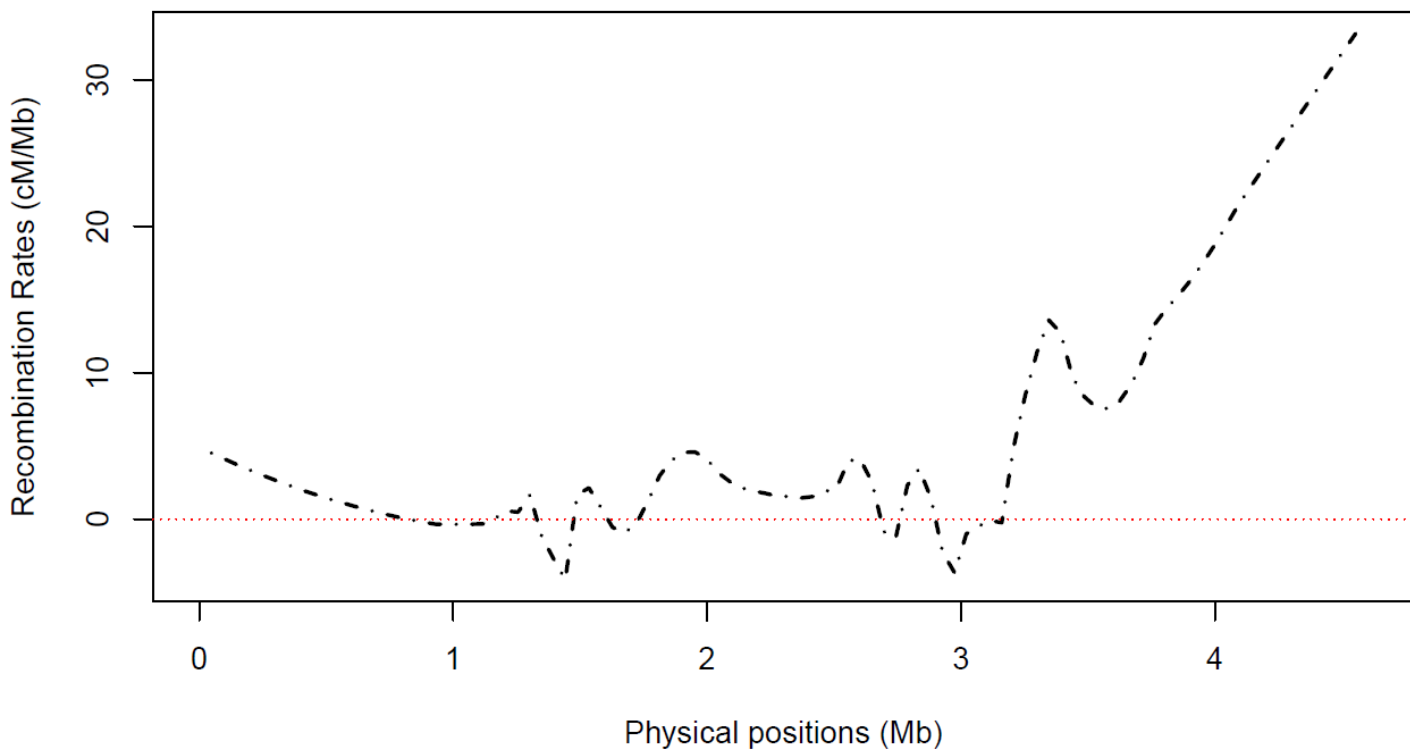

**Chr 26 Male**

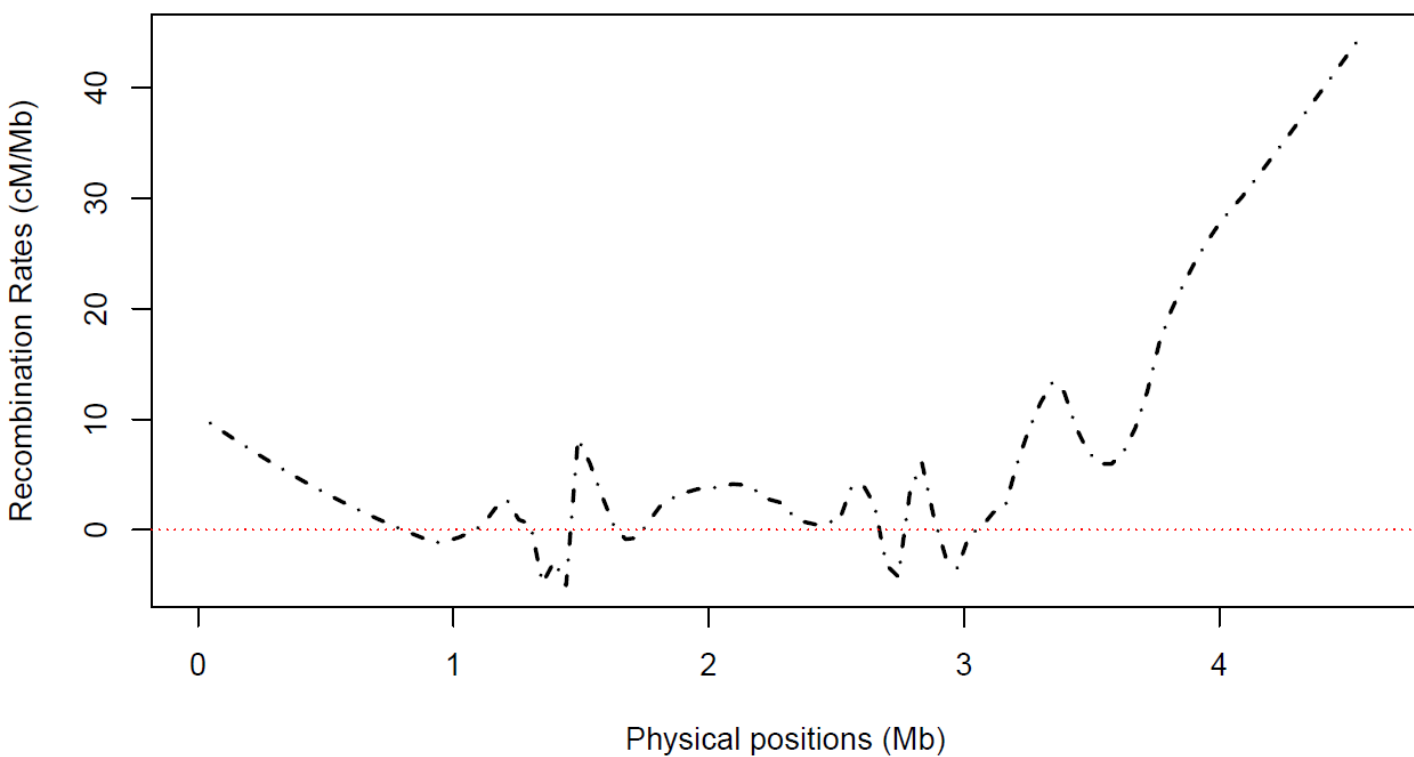

## Chr 27 Female

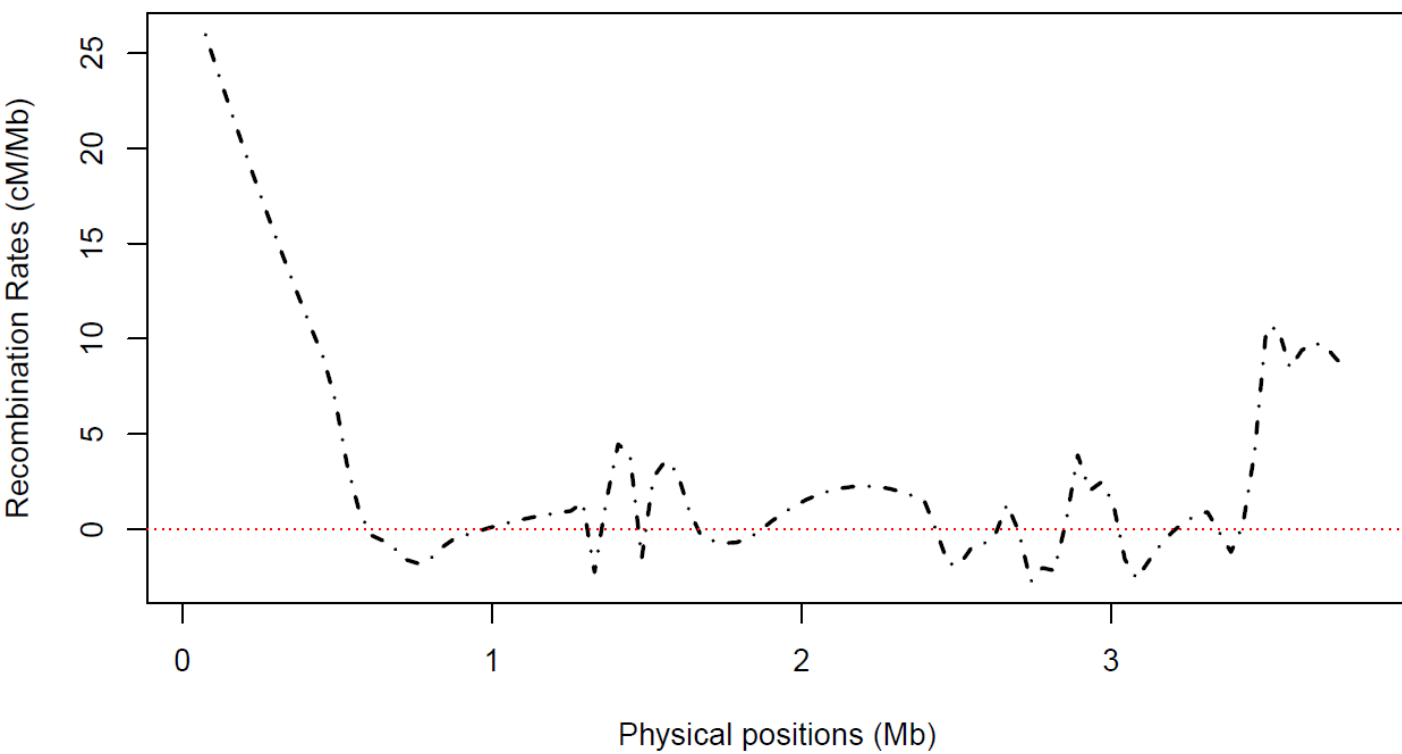

## Chr 27 Male

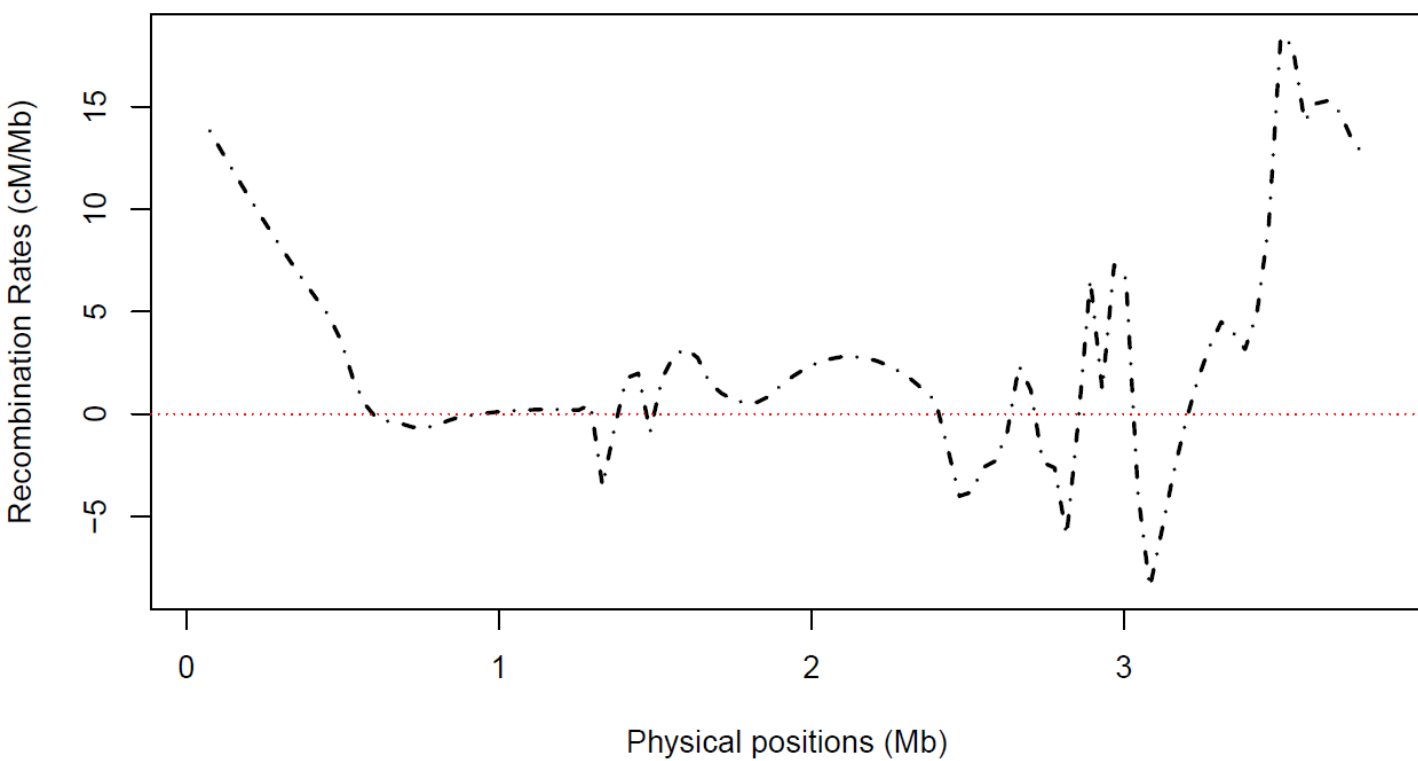

**Chr 28 Female**

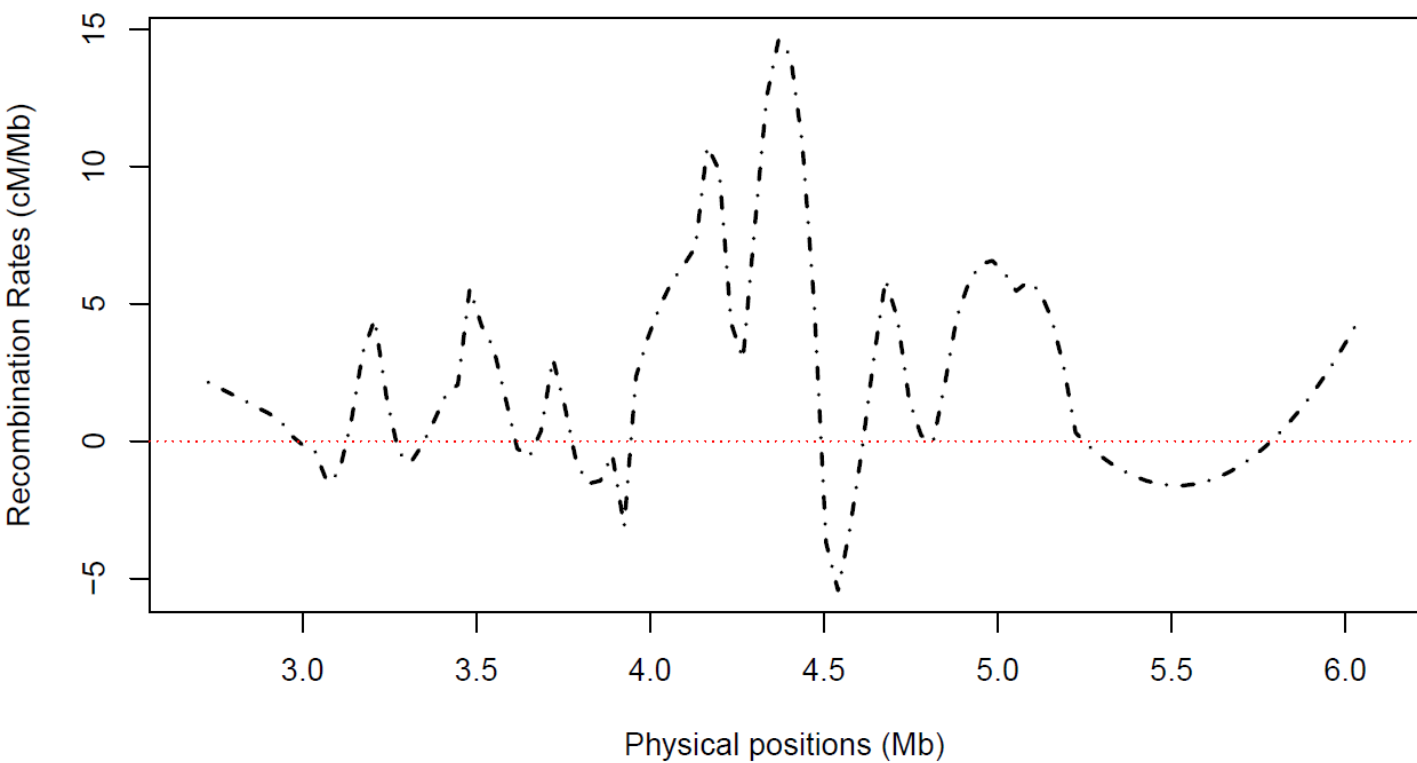

**Chr 28 Male**

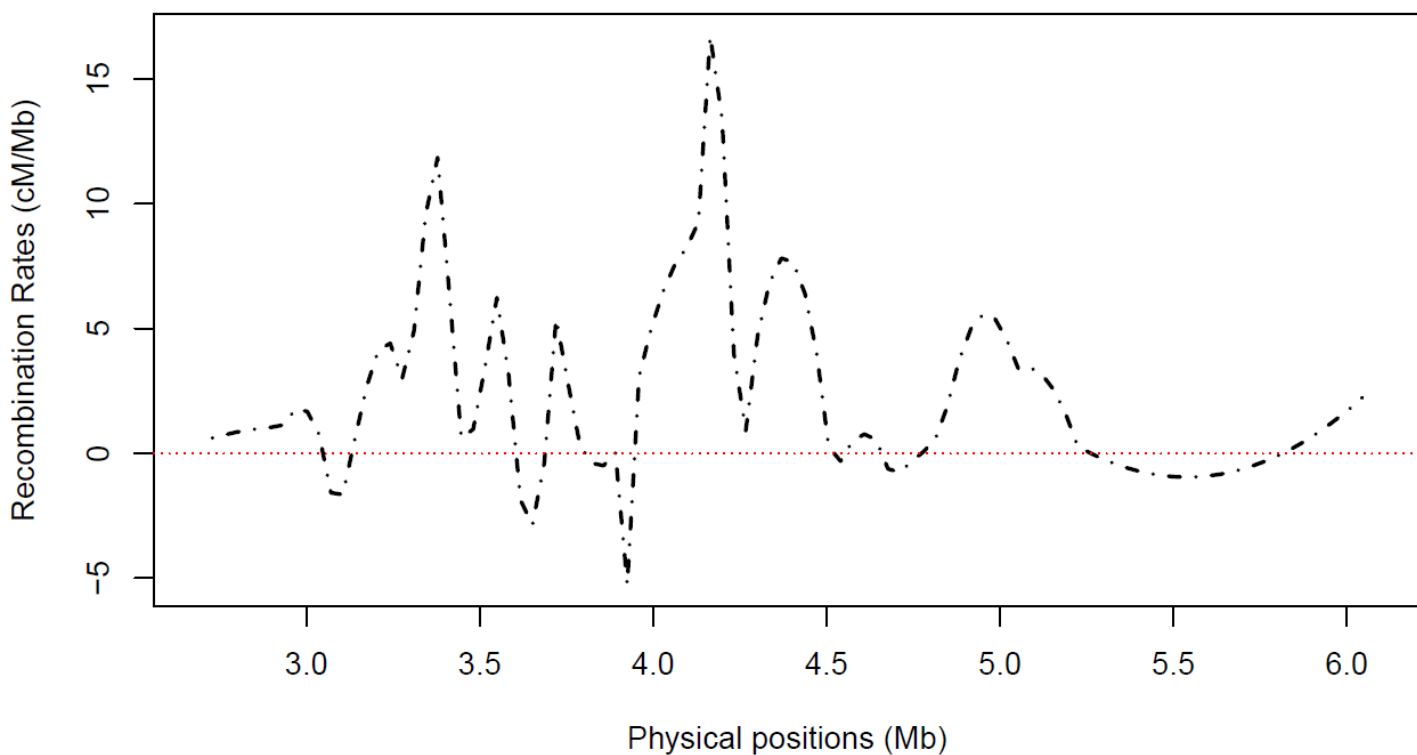

Supplement: giac025_Supplemental_Files [file giac025_supplemental_files.zip › Supplementary_Material_S14.pdf]

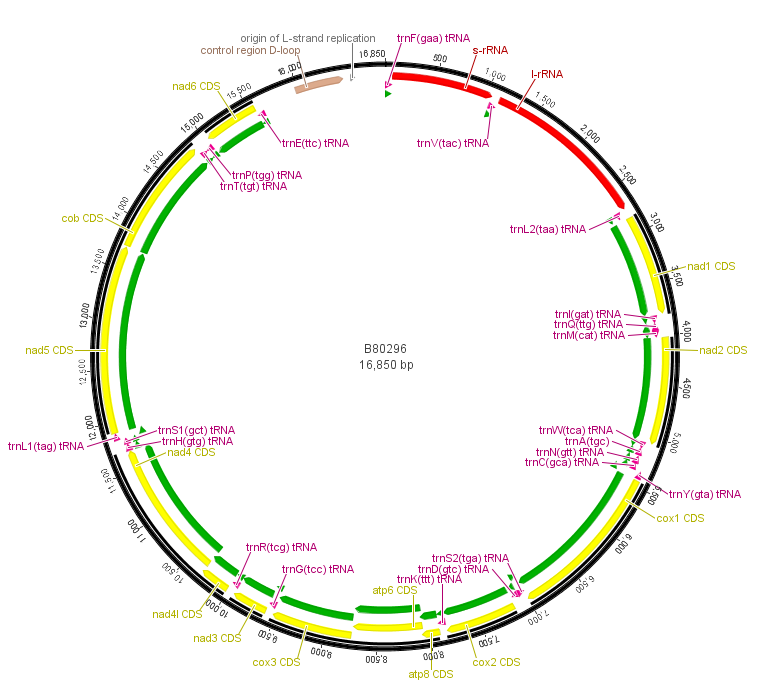

Supplement: giac025_Supplemental_Files [file giac025_supplemental_files.zip › Supplementary_Material_S2.png]

**A**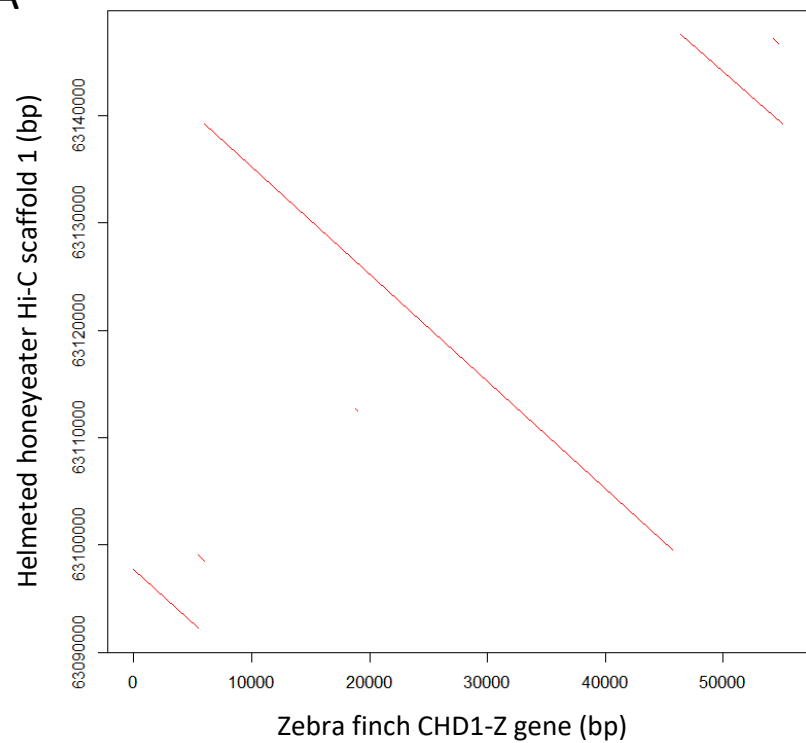**B**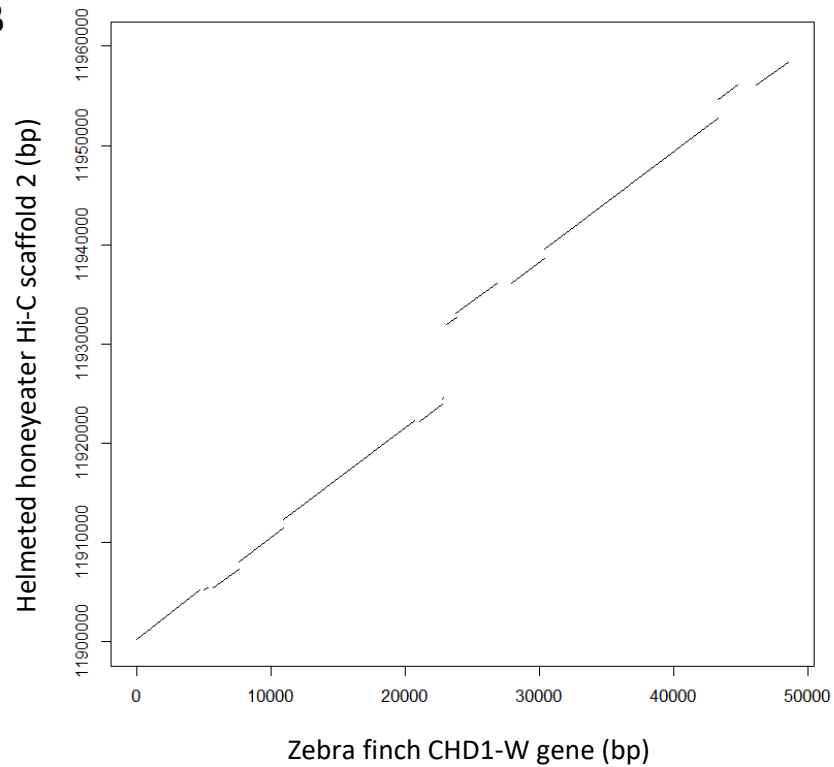

Supplement: giac025_Supplemental_Files [file giac025_supplemental_files.zip › Supplementary_Material_S7.pdf]

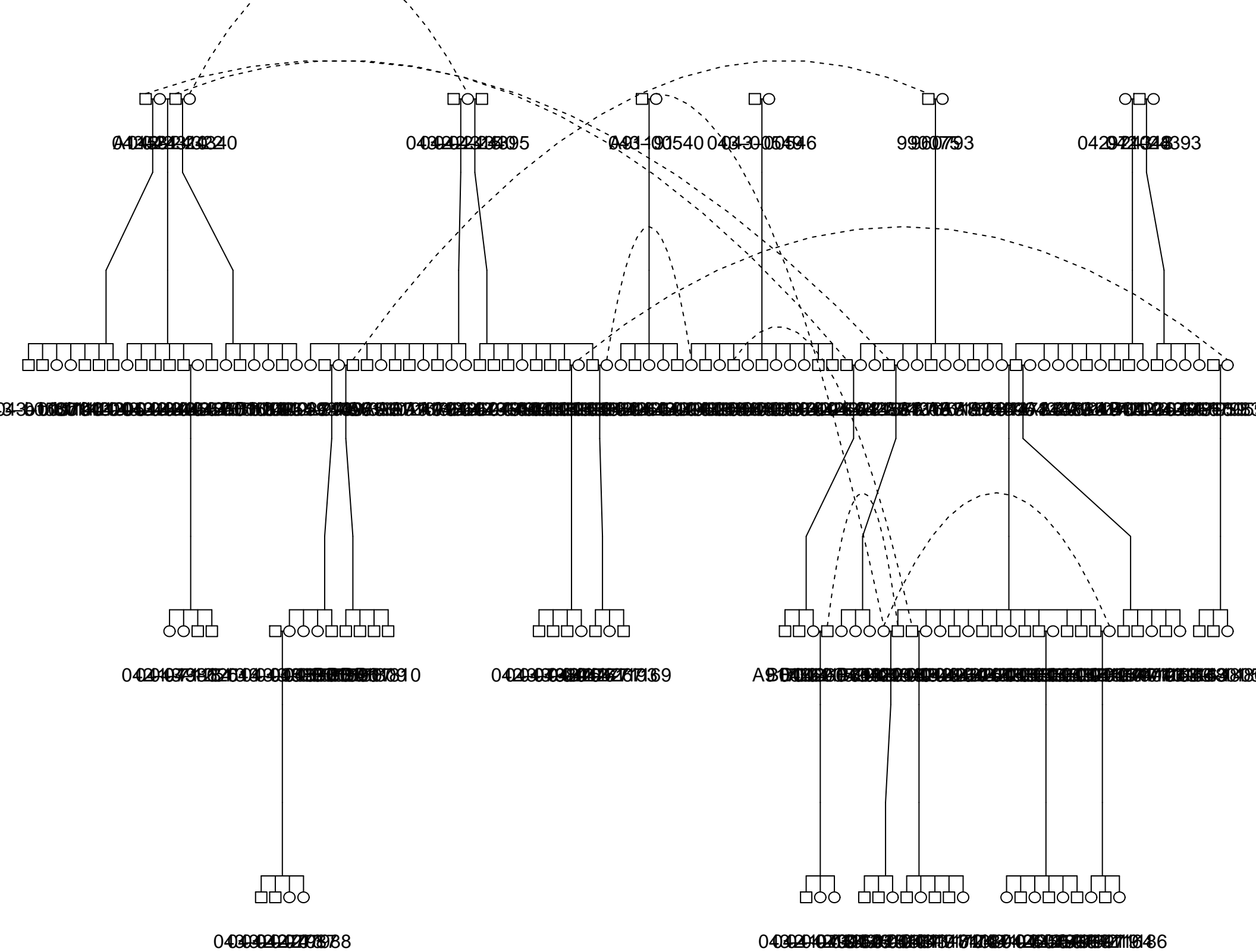

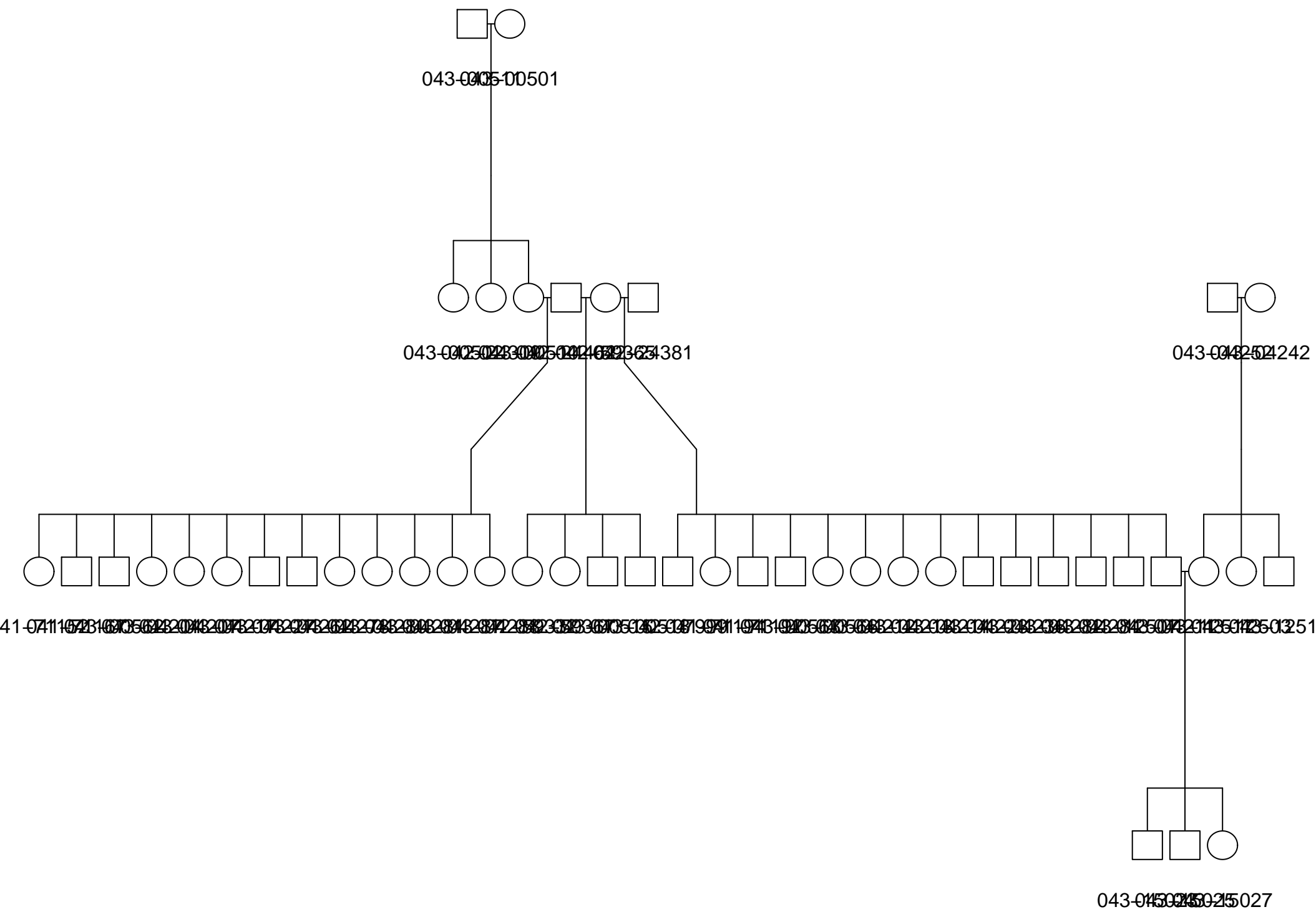

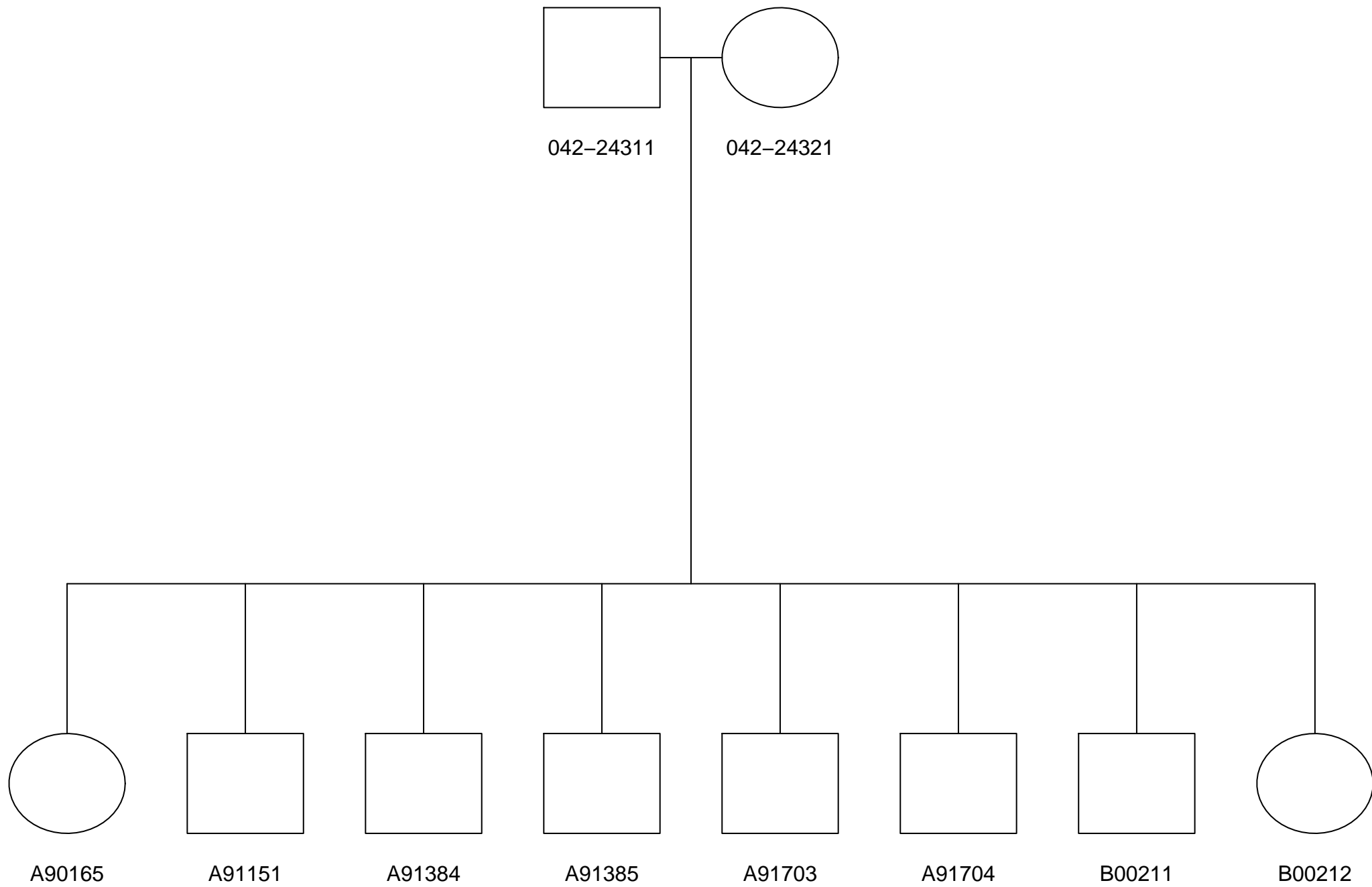

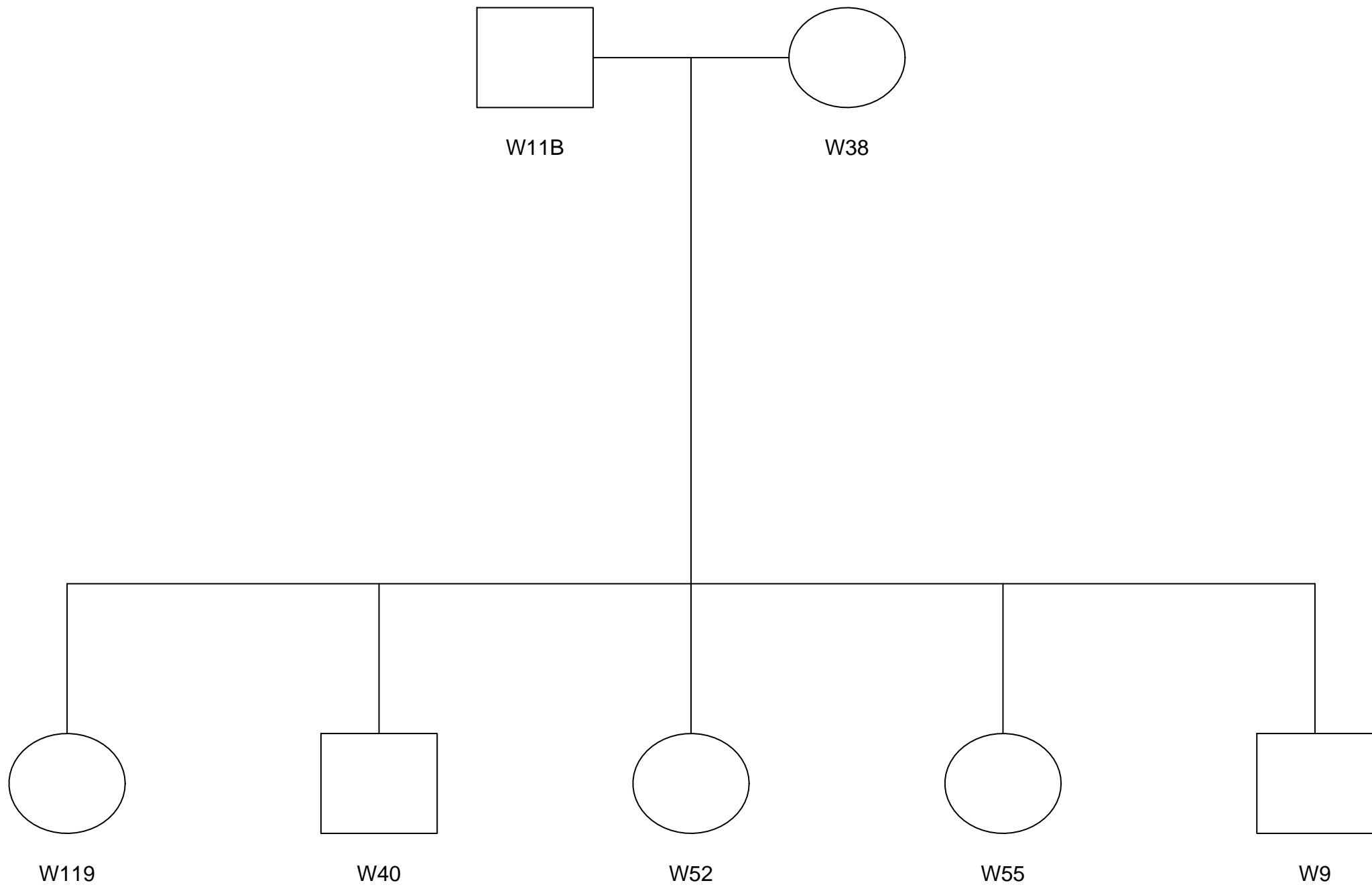

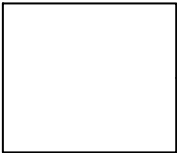

042-07967

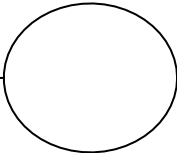

042-07955

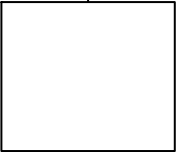

041-71195

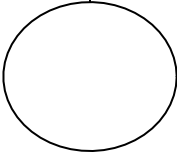

043-12504

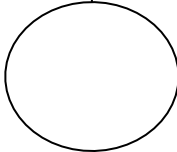

043-12505

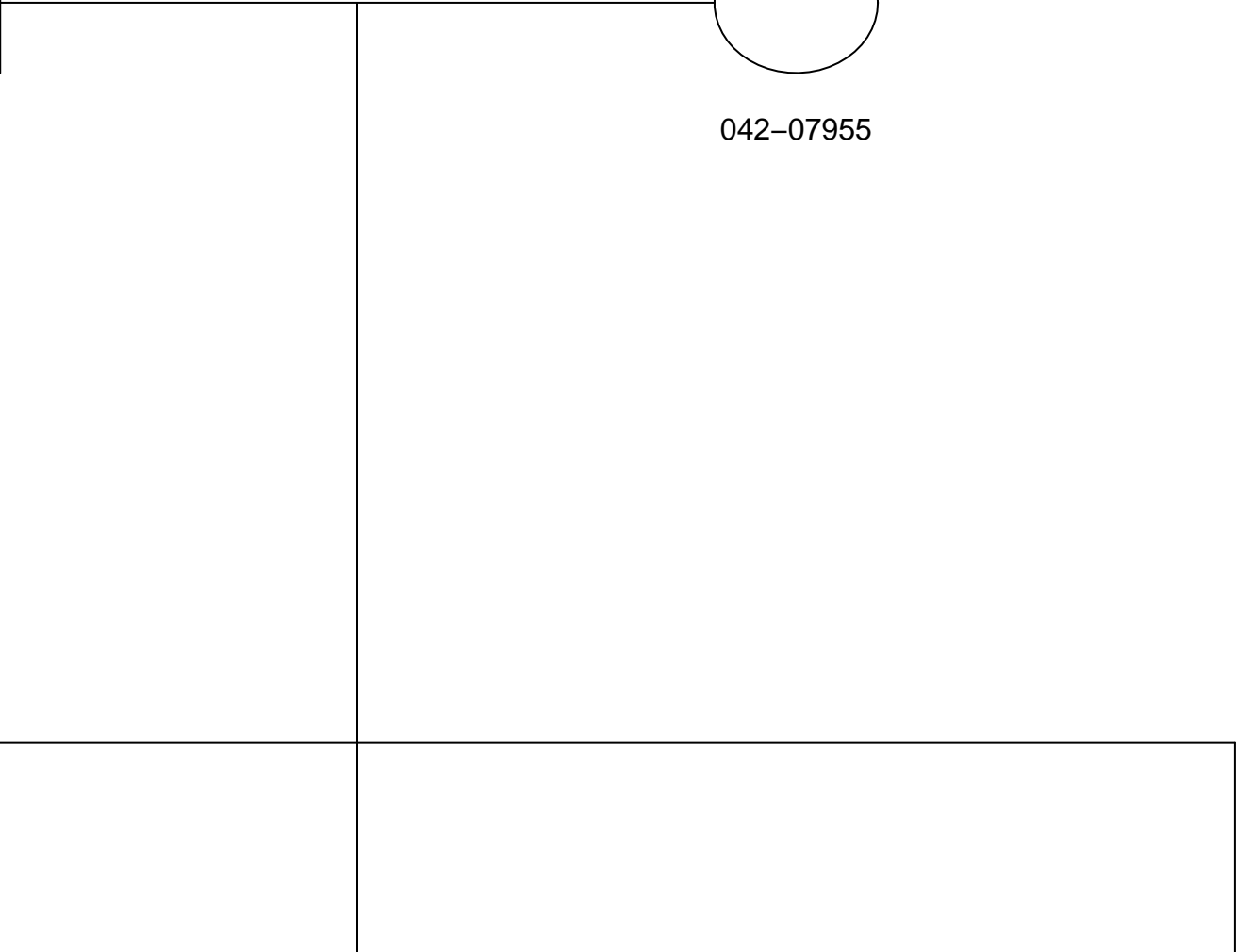

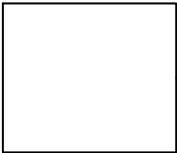

W58

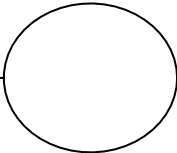

W57

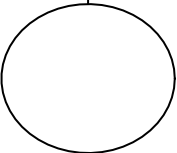

W56

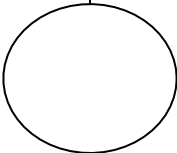

W24

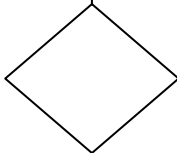

W79

Supplement: giac025_Supplemental_Files [file giac025_supplemental_files.zip › Supplementary_Material_S9.pdf]
