# Supplementary material for: Chromosome-length genome assembly and linkage map of a critically endangered Australian bird: the helmeted honeyeater
Source: Gigascience. 2022 Mar 29;11:giac025. doi: 10.1093/gigascience/giac025 (PMC8963300; doi:10.1093/gigascience/giac025)
Supplement: giac025_Supplemental_Files [file Supplementary_Material_S8.pdf › Supplementary_Material_S8.pdf]

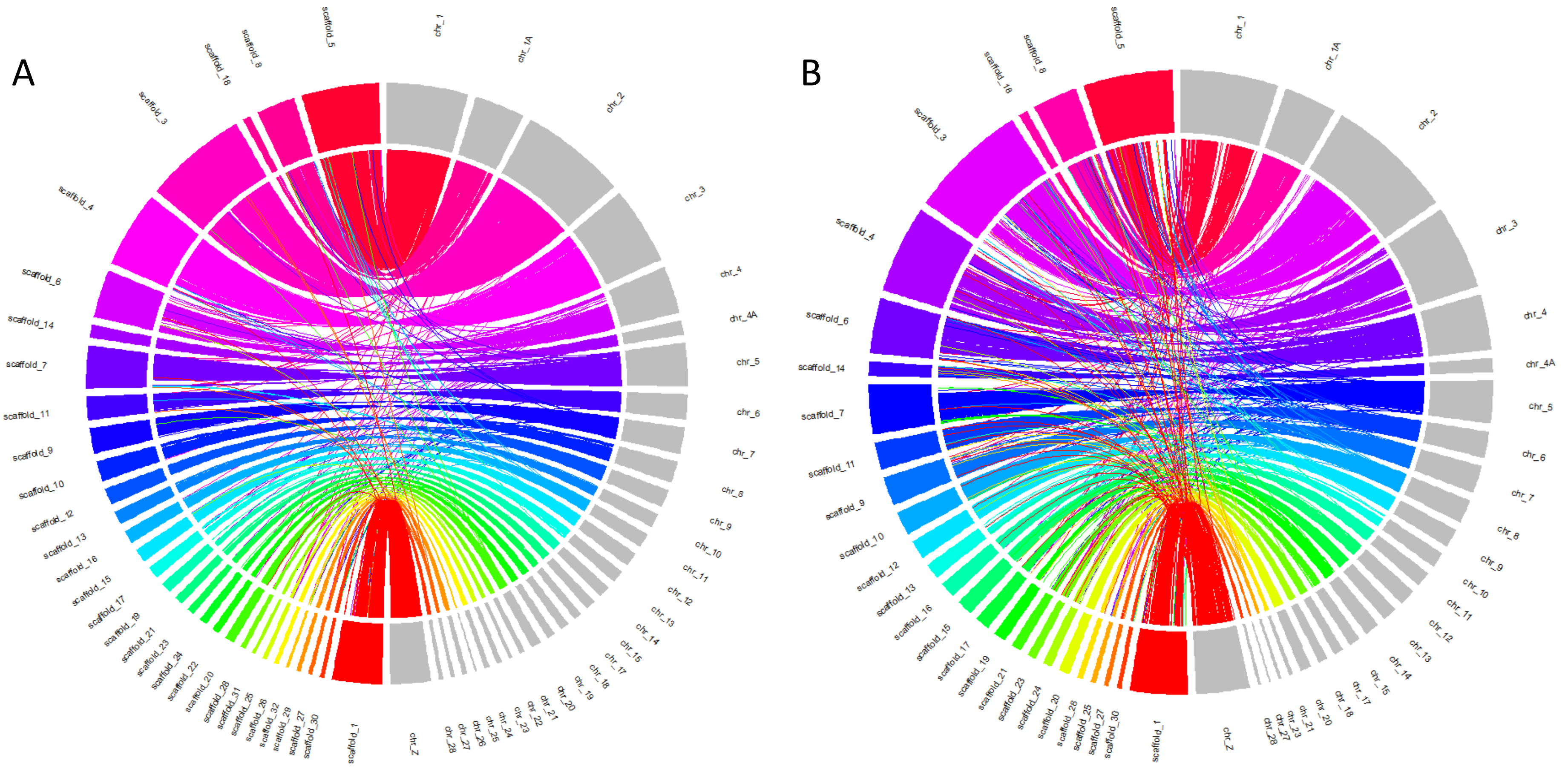
A
chr_1
chr_1A
chr_2
chr_3
chr_4
chr_4A
chr_5
chr_6
chr_7
chr_8
chr_9
chr_10
chr_11
chr_12
chr_13
chr_14
chr_15
chr_17
chr_18
chr_19
chr_20
chr_21
chr_22
chr_23
chr_24
chr_25
chr_26
chr_27
chr_28
chr_Z
scaffold_1
scaffold_30
scaffold_27
scaffold_29
scaffold_32
scaffold_26
scaffold_25
scaffold_31
scaffold_28
scaffold_20
scaffold_22
scaffold_24
scaffold_23
scaffold_21
scaffold_19
scaffold_17
scaffold_15
scaffold_16
scaffold_13
scaffold_12
scaffold_10
scaffold_9
scaffold_11
scaffold_7
scaffold_14
scaffold_6
scaffold_4
scaffold_3
scaffold_18
scaffold_8
scaffold_5
B
chr_1
chr_1A
chr_2
chr_3
chr_4
chr_4A
chr_5
chr_6
chr_7
chr_8
chr_9
chr_10
chr_11
chr_12
chr_13
chr_14
chr_15
chr_17
chr_18
chr_20
chr_21
chr_23
chr_27
chr_28
chr_Z
scaffold_1
scaffold_30
scaffold_27
scaffold_25
scaffold_28
scaffold_20
scaffold_24
scaffold_23
scaffold_21
scaffold_19
scaffold_17
scaffold_15
scaffold_16
scaffold_13
scaffold_12
scaffold_10
scaffold_9
scaffold_11
scaffold_7
scaffold_14
scaffold_6
scaffold_4
scaffold_3
scaffold_18
scaffold_8
scaffold_5
