# Supplementary material for: Chromosome-length genome assembly and linkage map of a critically endangered Australian bird: the helmeted honeyeater
Source: Gigascience. 2022 Mar 29;11:giac025. doi: 10.1093/gigascience/giac025 (PMC8963300; doi:10.1093/gigascience/giac025)
Supplement: giac025_Response_to_Reviewer_Comments_Revision_1 [file giac025_response_to_reviewer_comments_revision_1.pdf]

Author's Response To Reviewer Comments

Close

>>> We thank Dr Hongling Zhou for handling our manuscript and allowing us to improve it, and the reviewers for their useful comments and suggestions. We have addressed all the issues as outlined below. All changes to the manuscript are highlighted in yellow.

Reviewer #1: The authors present a high-quality chromosome length genome assembly of the helmeted honeyeater. The data presented is sound and the authors have used a range of complimentary approaches to obtain the assembly. The paper is written well and the experiments and procures are outlined very clearly. As the authors state in their introduction, a high-quality genome is a prerequisite for detailed population genetic studies of this endangered bird. I therefore am disappointed that they do not show any population genomic data in the manuscript. I understand that more detailed population genetic studies might be planned in other future manuscripts, but nevertheless some additional information should have been provided. With the data at hand (the chromosome level assembly and the high depth of Illumina data) it would have been rather straightforward to provide an overview (or plot) of the runs of homozygosity (ROHs) in the genome of the sequenced bird. The authors indicate that the effective population size of this species is very small and that the species suffers from inbreeding depression. A first view of the size distribution of ROHs in this bird would have been very informative in that respect. Likewise, the authors could have performed a PSMC analyses showing the effective population size over the past ~ 1 million years or so.
>>> We thank the reviewer for their helpful comments. As the reviewer correctly noted, we are preparing another manuscript that asks biological questions about the population genomics of the helmeted honeyeater population using ROHs, and focused on this manuscript being a “Data Note” according to GigaScience Author Guidelines (we contextualize this “exceptional dataset to encourage reuse” in order to “rapidly release data before subsequent detailed analysis has been carried out”). We have, however, followed the reviewer's suggestion to perform a PSMC analysis (lines 419-445, Figure 6) with which we exemplify the utility of our high-quality dataset and provide biological insight into the demographic history of the population.

There is a clear discrepancy between the assembled genome and the linkage maps for chromosomes Z, 13 and 26 (lines 350-356). The authors suggest that this might be due to genomic inversions in the genome of the sequenced bird. I find this highly unlikely. Did the authors carefully check reads crossing these potential breakpoints and can they rule out assembly artefacts? Likewise, if this indeed would be inversions, then it would be highly unlikely that both haplotypes of this bird would be affected, so I still would expect that conflicting reads should be present in the sequence data. The fact that the authors also do not include these regions in the recombination maps (see e.g. figure S11) already indicates that they have some doubts about these regions on these chromosomes.
>>> As per the reviewer’s recommendation we have mapped our long Nanopore reads to the three problematic chromosomes and visually checked the read coverage at the potential breakpoints. We found that in all three cases none of the read sequences span the potential breakpoints, which gives support to the possibility of a misassembly (Supplementary Material S13). These missasemblies will be corrected in the next version of the genome. We have added these findings to the manuscript in lines 358-365 and Supplementary Material S13, and we have removed the inversion hypothesis.

Minor comments:

Has the observed fision of chromosome 1A been observed in any other bird so far, or is this the first species that shows this fision?
>>> Neither the superb fairy-wren (close relative from the same superfamily), nor other passerine birds with chromosome-length genome and high-density linkage map, have reported this fission. Having both resources is necessary to be able to study synteny and linkage to be able to support the presence of a chromosomal fission/fusion. We have added this in lines 393-397.

The estimated distances in the linkage map are not precise given the small number of offs[ping in the

pedigree. Using decimals in the total size of the linkage maps, therefore does not make much sense and I would recommend to round these to cM's.
Line 53 and line 383 use 1347 rather than 1346.97
Line 384 use 1389 and 1302 instead of 1388.8 and 1301.9
>>> We have followed the reviewer's suggestions.

The scale of the y-axis in figure 4 should be changed. For many chromosomes the recombination rate does not exceed 5-10 cM/Mb. Using a scale that goes to 30 cM/Mb removes much of the information from these plots (e.g. compare figure 4 with figure S13).
>>> We have modified the y-axis of the first four rows in Figure 4 to scale to 15cM/Mb. We have also added a note on the figure legend for readers to notice the difference in scale in the fifth row.

Reviewer #2: The current manuscript presents a genome of the helmeted honeyeater assembled from Illumina, Oxford Nanopore, and Hi-C reads. This extensive data allowed authors to to assemble and annotated chromosome-length genome and high-density linkage map useful for follow up evolutionary studies and conservation efforts for the species. The paper contributes high quality data. It is technical and does not contain almost any biological or evolutionary context, but since it is in Data Note format, it is should be sufficient.
>>> We thank the reviewer for their helpful comments that helped us improve our manuscript.

Even though, it would benefit from putting observations in wider context by providing minor comments:
- Why was the synteny compared to zebra finch and not to collared flycatcher (even though the recombination was)
>>> We selected the zebra finch genome and not the collared flycatcher's for the synteny analysis and linkage mapping a few reasons:
1. The zebra finch was the second avian genome to be sequenced (only after the chicken genome) and as such has become a model passerine species genome.
2. The zebra finch genome GCF_008822105.2 is very recent (2020) and from a female individual which makes it more comparable to our female helmeted honeyeater genome. In comparison, the only chromosome-length genome for collared flycatcher in NCBI (GCA_000247815.2) was assembled in 2013 from a male individual.
3. The zebra finch genome used five sequencing technologies (including long reads and Hi-C sequencing) to produce a very complete assembly with scaffold N50 of 70.8Mb and coverage of 82.5x. On the other hand, the collared flycatcher genome was assembled using only two short-read sequencing technologies which translated in a scaffold N50 of 6.5Mb. Genome coverage was also lower (60x).
We have briefly clarified the relevance of comparing it to the zebra finch genome in lines 254-256.

- How much synteny is there compared with other bird genomes compared to the zebra finch
>>> We have added the analysis of the synteny between the helmeted honeyeater and the collared flycatcher and superb fairy-wren in lines 277-279, 286-288, and Supplementary Materials S8. Overall, the same pattern of highly conserved synteny was observed.

- Why is there so many more recombination hotspots on micro-chromosomes? Is this similar to other birds or unique?
>>> Thank you for the observation. We have added more detail on this in lines 411-414.

Close
