# Supplementary material for: Chromosome-length genome assembly and linkage map of a critically endangered Australian bird: the helmeted honeyeater
Source: Gigascience. 2022 Mar 29;11:giac025. doi: 10.1093/gigascience/giac025 (PMC8963300; doi:10.1093/gigascience/giac025)
Supplement: giac025_Reviewer_1_Report_Original_Submission — Martien Groenen -- 11/18/2021 Reviewed [file giac025_reviewer_1_report_original_submission.pdf]

Reviewer Report

Title: Chromosome-length genome assembly and linkage map of a Critically Endangered Australian bird: the helmeted honeyeater

Version: Original Submission **Date:** 11/18/2021

Reviewer name: Martien Groenen

Reviewer Comments to Author:

The authors present a high-quality chromosome length genome assembly of the helmeted honeyeater. The data presented is sound and the authors have used a range of complimentary approaches to obtain the assembly. The paper is written well and the experiments and procures are outlined very clearly. As the authors state in their introduction, a high-quality genome is a prerequisite for detailed population genetic studies of this endangered bird. I therefore am disappointed that they do not show any population genomic data in the manuscript. I understand that more detailed population genetic studies might be planned in other future manuscripts, but nevertheless some additional information should have been provided. With the data at hand (the chromosome level assembly and the high depth of Illumina data) it would have been rather straightforward to provide an overview (or plot) of the runs of homozygosity (ROHs) in the genome of the sequenced bird. The authors indicate that the effective population size of this species is very small and that the species suffers from inbreeding depression. A first view of the size distribution of ROHs in this bird would have been very informative in that respect. Likewise, the authors could have performed a PSMC analyses showing the effective population size over the past ~ 1 million years or so.

There is a clear discrepancy between the assembled genome and the linkage maps for chromosomes Z, 13 and 26 (lines 350-356). The authors suggest that this might be due to genomic inversions in the genome of the sequenced bird. I find this highly unlikely. Did the authors carefully check reads crossing these potential breakpoints and can they rule out assembly artefacts? Likewise, if this indeed would be inversions, then it would be highly unlikely that both haplotypes of this bird would be affected, so I still would expect that conflicting reads should be present in the sequence data. The fact that the authors also do not include these regions in the recombination maps (see e.g. figure S11) already indicates that they have some doubts about these regions on these chromosomes.

Minor comments:

Has the observed fision of chromosome 1A been observed in any other bird so far, or is this the first species that shows this fision?

The estimated distances in the linkage map are not precise given the small number of offs[ping in the pedigree. Using decimals in the total size of the linkage maps, therefore does not make much sense and I would recommend to round these to cM's.

Line 53 and line 383 use 1347 rather than 1346.97

Line 384 use 1389 and 1302 instead of 1388.8 and 1301.9

The scale of the y-axis in figure 4 should be changed. For many chromosomes the recombination rate does not exceed 5-10 cM/Mb. Using a scale that goes to 30 cM/Mb removes much of the information

from these plots (e.g. compare figure 4 with figure S13).

Level of Interest

Please indicate how interesting you found the manuscript: Choose an item.

Quality of Written English

Please indicate the quality of language in the manuscript: Choose an item.

Declaration of Competing Interests

Please complete a declaration of competing interests, considering the following questions:

- Have you in the past five years received reimbursements, fees, funding, or salary from an organisation that may in any way gain or lose financially from the publication of this manuscript, either now or in the future?
- Do you hold any stocks or shares in an organisation that may in any way gain or lose financially from the publication of this manuscript, either now or in the future?
- Do you hold or are you currently applying for any patents relating to the content of the manuscript?
- Have you received reimbursements, fees, funding, or salary from an organization that holds or has applied for patents relating to the content of the manuscript?
- Do you have any other financial competing interests?
- Do you have any non-financial competing interests in relation to this paper?

If you can answer no to all of the above, write 'I declare that I have no competing interests' below. If your reply is yes to any, please give details below.

I declare that I have no competing interests

I agree to the open peer review policy of the journal. I understand that my name will be included on my report to the authors and, if the manuscript is accepted for publication, my named report including any attachments I upload will be posted on the website along with the authors' responses. I agree for my report to be made available under an Open Access Creative Commons CC-BY license (http://creativecommons.org/licenses/by/4.0/). I understand that any comments which I do not wish to be included in my named report can be included as confidential comments to the editors, which will not be published.

Choose an item.

To further support our reviewers, we have joined with Publons, where you can gain additional credit to further highlight your hard work (see: https://publons.com/journal/530/gigascience). On publication of this paper, your review will be automatically added to Publons, you can then choose whether or not to claim your Publons credit. I understand this statement.

Yes Choose an item.
