# Supplementary material for: Chromosome-length genome assembly and linkage map of a critically endangered Australian bird: the helmeted honeyeater
Source: Gigascience. 2022 Mar 29;11:giac025. doi: 10.1093/gigascience/giac025 (PMC8963300; doi:10.1093/gigascience/giac025)
Supplement: giac025_Reviewer_2_Report_Original_Submission — Taras K Oleksyk, Ph.D. -- 11/24/2021 Reviewed [file giac025_reviewer_2_report_original_submission.pdf]

Reviewer Report

Title: Chromosome-length genome assembly and linkage map of a Critically Endangered Australian bird: the helmeted honeyeater

Version: Original Submission **Date:** 11/24/2021

Reviewer name: Taras K Oleksyk, Ph.D.

Reviewer Comments to Author:

The current manuscript presents a genome of the helmeted honeyeater assembled from Illumina, Oxford Nanopore, and Hi-C reads. This extensive data allowed authors to to assemble and annotated chromosome-length genome and high-density linkage map useful for follow up evolutionary studies and conservation efforts for the species. The paper contributes high quality data. It is technical and does not contain almost any biological or evolutionary context, but since it is in Data Note format, it is should be sufficient.

Even though, it would benefit from putting observations in wider context by providing minor comments:

- Why was the synteny compared to zebra finch and not to collared flycatcher (even though the recombination was)
- How much synteny is there compared with other bird genomes compared to the zebra finch
- Why is there so many more recombination hotspots on micro-chromosomes? Is this similar to other birds or unique?

Level of Interest

Please indicate how interesting you found the manuscript: Choose an item.

Quality of Written English

Please indicate the quality of language in the manuscript: Choose an item.

Declaration of Competing Interests

Please complete a declaration of competing interests, considering the following questions:

- Have you in the past five years received reimbursements, fees, funding, or salary from an organisation that may in any way gain or lose financially from the publication of this manuscript, either now or in the future?
- Do you hold any stocks or shares in an organisation that may in any way gain or lose financially from the publication of this manuscript, either now or in the future?
- Do you hold or are you currently applying for any patents relating to the content of the manuscript?

- Have you received reimbursements, fees, funding, or salary from an organization that holds or has applied for patents relating to the content of the manuscript?
- Do you have any other financial competing interests?
- Do you have any non-financial competing interests in relation to this paper?

If you can answer no to all of the above, write 'I declare that I have no competing interests' below. If your reply is yes to any, please give details below.

I do not have any competing interests

I agree to the open peer review policy of the journal. I understand that my name will be included on my report to the authors and, if the manuscript is accepted for publication, my named report including any attachments I upload will be posted on the website along with the authors' responses. I agree for my report to be made available under an Open Access Creative Commons CC-BY license (http://creativecommons.org/licenses/by/4.0/). I understand that any comments which I do not wish to be included in my named report can be included as confidential comments to the editors, which will not be published.

Choose an item.

To further support our reviewers, we have joined with Publons, where you can gain additional credit to further highlight your hard work (see: https://publons.com/journal/530/gigascience). On publication of this paper, your review will be automatically added to Publons, you can then choose whether or not to claim your Publons credit. I understand this statement.

Yes Choose an item.
